# A structure-based approach towards the identification of novel antichagasic compounds: *Trypanosoma cruzi* carbonic anhydrase inhibitors

**DOI:** 10.1080/14756366.2019.1677638

**Published:** 2019-10-16

**Authors:** Manuel A. Llanos, María L. Sbaraglini, María L. Villalba, María D. Ruiz, Carolina Carrillo, Catalina Alba Soto, Alan Talevi, Andrea Angeli, Seppo Parkkila, Claudiu T. Supuran, Luciana Gavernet

**Affiliations:** a Laboratory of Bioactive Research and Development (LIDeB), Medicinal Chemistry, Department of Biological Sciences, Faculty of Exact Sciences, National University of La Plata, Buenos Aires, Argentina; b Instituto de Ciencias y Tecnología Dr. Cesar Milstein (ICT Milstein), Argentinean National Council of Scientific and Technical Research (CONICET), Buenos Aires, Argentina; c Departamento de Microbiología, Parasitología e Inmunología, Facultad de Medicina, Universidad de Buenos Aires, Instituto de Investigaciones en Microbiología y Parasitología Médica (IMPaM), UBA-CONICET, Buenos Aires, Argentina; d Neurofarba Department, Sezione di Scienze Farmaceutiche e Nutraceutiche, Universita degli Studi di Firenze, Sesto Fiorentino, Florence, Italy; e Faculty of Medicine and Health Technology, University of Tampere, Tampere, Finland

**Keywords:** Chagas, carbonic anhydrase, virtual screening, sulphamides, sodium cyclamate, acesulphame

## Abstract

*Trypanosoma cruzi* carbonic anhydrase (*Tc*CA) has recently emerged as an interesting target for the design of new compounds to treat Chagas disease. In this study we report the results of a structure-based virtual screening campaign to identify novel and selective *Tc*CA inhibitors. The combination of properly validated computational methodologies such as comparative modelling, molecular dynamics and docking simulations allowed us to find high potency hits, with K_I_ values in the nanomolar range. The compounds also showed trypanocidal effects against *T. cruzi* epimastigotes and trypomastigotes. All the candidates are selective for inhibiting *Tc*CA over the human isoform CA II, which is encouraging in terms of possible therapeutic safety and efficacy.

## Introduction

Carbon dioxide (CO_2_) is a product of many metabolic aerobic processes in different organisms. It has a relatively low solubility in water, but it reacts with this solvent (at the neutral pH) to produce bicarbonate anion and protons. These two ions play a crucial role in maintaining pH homeostasis in living systems and their equilibration with CO_2_ is controlled by the activity of the enzymes Carbonic Anhydrase (CA, EC 4.2.1.1)^1–5^. CAs are extremely efficient catalysts, and they are widespread in organisms all over the phylogenetic tree. So far, there are eight genetically distinct families categorised as α-, β-, γ-, δ-, ζ-, η-, Θ- and ⌈-CAs^6–12^. In mammals, there are 16 isoforms known to date, that belong to the α-CA family and some of them have been studied as molecular targets to treat human diseases^8–12^. Traditionally, CA inhibitors are employed as diuretic and antiglaucoma drugs, but other clinical applications have been proposed, including the treatment of cancer, obesity and epilepsy^13–19^. Regarding neglected protozoan diseases, such as the American trypanosomiasis, CA is emerging as a new therapeutic target, with its inhibitors being probably able to provide an increased efficacy and safety in comparison with the current treatments^20^. American trypanosomiasis, also known as Chagas disease, is caused by the parasite *Trypanosoma cruzi*^21^. It was first described by Carlos Chagas more than a century ago and it affects about 8 million people worldwide^22^. The disease is characterised by two phases: an initial acute stage which usually goes undetected because it lacks specific symptoms and; a chronic stage that may remain asymptomatic but evolves to clinical manifestations in specific organs (heart, digestive system and/or nervous system) in about 30% of the patients^23^. The therapeutic arsenal to treat Chagas is composed of only two drugs, benznidazole and nifurtimox^23^. They were discovered more than 40 years ago, and they are mostly active in the acute phase of the disease. Their limited efficacy in the chronic phase as well as their considerable toxicity fostered the efforts to explore the molecular biology of *T. cruzi* aiming to discover new therapeutic targets. In this scenario, the inhibition of protozoan CAs showed up recently as an interesting option for the future treatment of Chagas disease, as well as other neglected pathologies like Leishmaniasis^20^.

At present, only one *T. cruzi* carbonic anhydrase (*Tc*CA) has been described in the genome of the parasite. *Tc*CA has recently been cloned and characterised by Pan et al.^24^ It belongs to the α-CA family and shares the active site topology with the catalytic α-isoforms found in mammals: the three His residues coordinating with one Zinc ion, and a fourth coordination position occupied by a water molecule at acidic pH (<7) or by a hydroxide ion at higher pH values^20^^,^^24^. The role of the enzyme in the pathogen is still poorly understood, but it is known that *Tc*CA shows a very high catalytic activity^24^^,^^25–29^. It has been hypothesised that *Tc*CA is involved in the proliferation of the parasite when it is in the epimastigote stage^20^. This assumption was supported by some *in vitro and ex vivo* studies that demonstrated the ability of *Tc*CA inhibitors to inhibit epimastigote proliferation.

Regarding the chemical structure of CA inhibitors, sulphonamides are the main class of compounds that interacts with CAs in mammals, especially with CA II (which is, by far, the most studied isoform)^13–19^. The classical mechanism of inhibition involves the interaction of the sulphonamide nitrogen atom with the Zinc ion, which places the inhibitors in the position originally occupied by water (or the hydroxide ion)^13–19^^,^^30^. *Tc*CA is also inhibited by other compound families, such as thiols and hydroxamates^20^. Moreover, molecules from these two classes showed better potency and selectivity *in vitro* compared to sulphonamides, as well as promising *in vivo* profiles, by inhibiting the three phases of the pathogen’s life cycle (trypomastigote, amastigote and epimastigote)^24^. Following these previous investigations, we investigate here the capacity of a new set of compounds to inhibit *Tc*CA. One of the main challenges in the development of new CA inhibitors is to find selectivity against the specific isoforms involved in the disease, to avoid tolerance problems and to improve the therapeutic safety. Therefore, the selection of the candidates for biological assays was guided by the results of a virtual screening protocol applied to a set of compounds that share a zinc binding function (sulphamides, and their bioisosters sulphamates and sulphonamides) and have low inhibitory activity against the ubiquitous human isoform CA II, which could be considered as an anti-target.

Our investigation initiated with the construction of three-dimensional models of *Tc*CA, since the experimental structure of the target is not yet available. A test set of 87 known *Tc*CA inhibitors was then compiled from literature and used to select the best docking model for the virtual screening, based on the correlation between the docking score and the experimentally determined pK_I_. Finally, an in-house dataset of 255 compounds was screened and 10 compounds were tested against *Tc*CA. Anti-trypanosomal effects were also investigated for the selected candidates. Information about the identity of the structures of the test set (Table S1) and the dataset for screening is given as supporting information (Table S2) (see Supplementary information for details)

## Materials and methods

### Protein structure modelling

We applied the Meier et al.^31^ algorithm as implemented in the HHPred Server^32^ to construct our initial model of the enzyme. The sequence of *Tc*CA was retrieved from Uniprot (Q4CVY4)^33^. A multi-template model was generated combining the top 10 templates ranked by its alignment score. The quality of the model was evaluated with the metrics provided by the server, Molprobity and the QMEAN scoring functions^32^. Then, the macrostructure was submitted to an iterative refinement process, combining Rosetta and Molecular Dynamics (MD) simulations. For that purpose, we used Rosetta’s Fast Relax algorithm with the metalloproteins modification, which imposes constraints on the Zinc ion and its coordinating residues to preserve the coordination geometry during the simulation^34–37^. Briefly, the protocol consists of alternating cycles of rotamer repacking and gradient-based energy minimisation, increasing the repulsive contributions within each cycle. After generating 100 new refined models, the top scoring decoy was selected as the starting point for MD simulations.

MD simulations were carried out with the Amber16 package^38^, using the FF14SB force field and the Zinc AMBER force field (ZAFF) modification designed for 4-coordinated Zinc metal centres^39^. Because the three His residues that coordinate with the zinc ion in human CA are also conserved in *Tc*CA, we assumed a tetrahedral coordination for the Zinc atom and a water molecule was manually placed in the fourth vertex to fulfil the coordination geometry. Thus, the centre ID No. 6 in the ZAFF force field was selected to set the parameters, which were derived by Mertz et al. from human CA-II crystal structure 1CA2 using the MCPB program^39^.

The structure was solvated in an octahedral box of TIP3P water molecules, and Cl^−^ ions were added to neutralise the system. Minimisation of the system was performed in four sequential steps. First, only hydrogen atoms were minimised. Second, only the solvent around the protein was allowed to move. Third, only protein side chains and solvent were minimised, fixing the position of the protein backbone atoms. Finally, the whole system was minimised with no constraints. Every minimisation stage involved 2500 steepest descent steps, followed by 7500 steps of conjugate gradient. Then, the system was carefully heated to 298.15 K and equilibrated in six consecutive steps, starting in NVT conditions and then switching to an NPT ensemble to equilibrate the system density, progressively relieving constraints to achieve a proper equilibration. A total of 50 ns was simulated, monitoring the protein backbone RMSD as a simulation convergence criterion, and 10 representative snapshots were extracted by clustering analysis. For that purpose, the whole trajectory was clustered using the *K*-means algorithm implemented in cpptraj, fixing the number of clusters (*K* = 10) and choosing the cluster centroid as the representative structure in each case. Finally, all the selected snapshots were subjected to a later refinement step using Rosetta’s Fast Relax protocol described before^34–37^, to sample high-quality decoys around the starting conformation. These 10 final *Tc*CA structural models were used for docking simulation.

### Construction of the dataset and validation of the docking models

A validation set of 87 molecules tested against the *Tc*CA was compiled from literature^20^. It includes compounds with different chemical structures but mostly presenting sulphonamides, thiols and hydroxamates as Zinc binding functions. The activity values (K_I_) of the compounds range between 0.51 and 84,000 nM. These reported activities were transformed to pK_I_ values (Table S1). Benzoxaboroles described by Nocentini et al.^40^ were excluded from the dataset because the docking forcefield does not include parameters for boron atoms. For each molecule, the most abundant species at pH 7.4 was calculated with ChemAxon^41^, considering tautomerization and resonance effects. Finally, 5000 steps of geometry optimisation were carried out with the MMFF94S force field, to get reasonable starting geometries. It is worth noticing that thiol compounds were predicted to be deprotonated at the given pH, with the negative charge stabilised by a nitrogen atom from an adjacent heteroaromatic ring that is present in these molecules. The docking calculations were run with AutoDockZN^42^, a software that includes modifications of the classic AutoDock4 force field with specific parameters for the zinc ion. This forcefield has proven to overcome the performance of the original AutoDock4^43^ for docking small molecules into Zinc metalloproteins, in both binding energy estimation and pose prediction. The grid maps were constructed centred on the Zinc atom for every *Tc*CA model with Autogrid 4.2.6^43^. They covered 60 points in each dimension from the Zinc centre, with a spacing of 0.375 Å. The docking simulations were run by considering a population size of 300 and an elitism of 1. We performed 100 runs of the Lamarckian Genetic Algorithm with AutoDock4.2^43^. The scoring power of every docking model (combination of docking programme and *Tc*CA structural model) was evaluated by calculating the Pearson correlation coefficient (Pearson’s *r*) between the docking scores and the p*K*_I_ values for all the compounds in the validation set. Additionally, we evaluated the ranking power of the models by calculating Kendall’s rank correlation coefficient (Tau B) and Spearman’s rank correlation coefficient (Rho).

**Figure 1.**
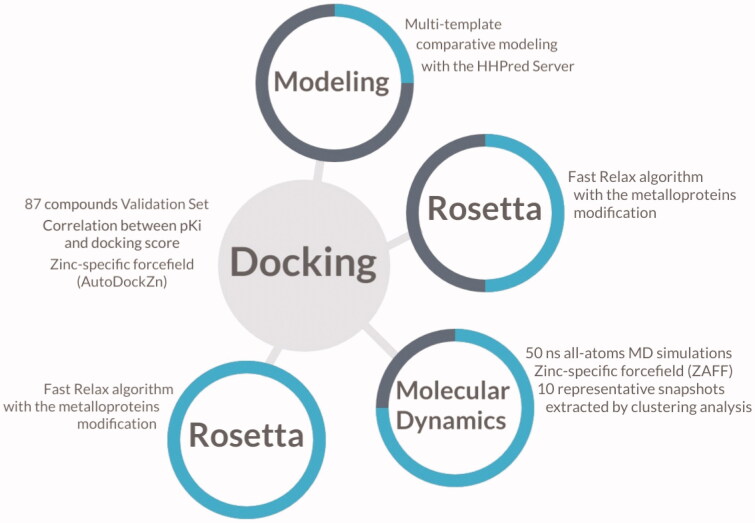
Schematic illustration of the methods involved in the construction of the model for the virtual screening. The progress of the investigation is described as a cyan circle in each step of the molecular modelling.

In order to evaluate the model’s performance as a classifier, compounds were defined either as active or inactive based on the reported *K*_I_ value. To this end, compounds with a *K*_I_ value lower than 1 μM were considered as actives. Then, the Area Under the Receiver Operating Characteristic Curve (ROC-AUC) was calculated as a metric to evaluate the discriminating power of the model. Figure 1 shows a schematic representation of the methods used in this investigation to create and validate the structure-based predictive model of *Tc*CA for the virtual screening campaign.

### Virtual screening

The previously described docking protocol was selected to screen an inhouse library of 255 compounds (Table S2). Some of these compounds were synthesised in our lab^44–46^, whilst others were commercial compounds like the artificial sweeteners cyclamate and acesulphame. Among top scoring hits, 10 structures were selected for biological evaluation. All the candidates were tested before as human CA II inhibitors and they showed poor inhibitory potency in this mammalian isoform^46–48^. As previously mentioned, CA II is ubiquitous in humans and it was considered as an anti-target in this investigation, since we are looking for selective inhibitors of the parasite isoform.

### Biological assays

#### TcCA inhibition studies

An Sx.18Mv-R Applied Photophysics (Oxford, UK) stopped-flow instrument has been used to assay the catalytic activity of various CA isozymes for CO_2_ hydration reaction^49^. Phenol red (at a concentration of 0.2 mM) was used as indicator, working at the absorbance maximum of 557 nm, with 10 mM Hepes (pH 7.5) as buffer, 0.1 M Na_2_SO_4_ (for maintaining constant ionic strength), following the CA-catalyzed CO_2_ hydration reaction for a period of 10 s at 25 °C. The CO_2_ concentrations ranged from 1.7 to 17 mM for the determination of the kinetic parameters and inhibition constants. For each inhibitor at least six traces of the initial 5–10% of the reaction have been used for determining the initial velocity. The uncatalyzed rates were determined in the same manner and subtracted from the total observed rates. Stock solutions of inhibitors (10 mM) were prepared in distilled-deionised water and dilutions up to 1 nM were done thereafter with the assay buffer. Enzyme and inhibitor solutions were pre-incubated together for 15 min (standard assay at room temperature) prior to assay, to allow for the formation of the enzyme–inhibitor complex. The inhibition constants were obtained by non-linear least-squares methods using PRISM 3 and the Cheng–Prusoff equation, as reported earlier^40^^,^^50–72^. All CAs were recombinant proteins produced as reported earlier by our group^24^.

#### Biological activity against *T. cruzi* epimastigotes

For all biological assays, stock and working solutions of the candidate drugs were prepared using DMSO as solvent and all conditions were tested in triplicate.

Epimastigotes of the Y strain of *T. cruzi* were cultured at 28 °C in BHT medium supplemented with 20 μg/ml haemin, 10% heat-inactivated foetal bovine serum (FBS), 100 µg/ml streptomycin and 100 U/ml penicillin. The antiproliferative activity of the candidates was tested at 50 µM concentration in cultures initiated at 10^7^ cells/ml. Untreated controls were performed under the same culture conditions with equal concentrations of DMSO as for candidate drugs. After 3 and 7 d, the number of viable parasites was counted using a haemocytometer chamber under light microscope and results were expressed as percentage respect of the untreated controls^73^.

#### Biological activity against *T. cruzi* trypomastigotes

Trypomastigotes of *T. cruzi* were purified at the parasitemia peak from peripheral blood of mice infected with the RA strain. Trypomastigotes (1 × 10^5^ per well) were cultured in a 96 well-plate (final volume 200 μl) in RPMI medium supplemented with 10% FBS at 37 °C in 5% CO_2_ atmosphere. After 24 h motile parasites were counted in haemocytometer chamber under the light microscope^74^. Results were expressed as % viability of trypomastigotes (respect to control) at 20 µM of the hits. Controls were performed under the same culture conditions with equal concentrations of DMSO as for candidate drugs. The negative control was cultured with PBS and the positive control was cultured with Benznidazole (20 µM). The animal care for the experimental protocols was conducted in accordance with the guidelines for the care and use of laboratory animals approved by the Ethical Committee of the Faculty of Medicine of the University of Buenos Aires.

## Results and discussion

### Construction of TcCA models

*Tc*CA has been recently cloned and characterised by Pan et al.^24^ As mentioned before, the three His residues coordinating the Zinc ion are conserved in the active site of *Tc*CA, with the fourth coordination position occupied by a water molecule (or by a hydroxide ion, depending on the pH). Like human CA II, *Tc*CA exerts a high catalytic activity, and the gatekeeping residues Glu106 and Thr199 are well conserved^8–12^. However, the proton shuttle His64 is absent in *Tc*CA isoform. Despite several CA structures of many organisms have been crystallised so far, there is not a good template to perform a comparative modelling of *Tc*CA. There are 924 structures of CAs deposited in the Protein Data Bank (PDB)^75^, but all of them have sequence identities lower than 30% with *Tc*CA. In this situation, the combination of multiple templates generally increases the quality of the resultant model, but only when the correct templates are combined since there is a trade-off between the number of sequences included and the noise introduced in the restraints. One possible approach to address this challenge was recently proposed by Meier et al.^31^ and used in this investigation. They introduced a modification in the MODELLER algorithm, using probability theory to combine the density functions of individual template restraints^76^.

The multiple sequence alignment is provided as Supporting Information (Figure S1), including the PDB ID and UNIPROT ID of each template selected. The final model is 261 amino acids long, spanning from residue 56 to 316 of the target sequence. The structural quality metrics are summarised in Table 1 for every intermediate structure of the modelling pipeline, from the raw model generated by the HHPred server to the refined MD snapshot selected as the best *Tc*CA model for docking (the criteria for this selection was included in the next section). The quality of the models was estimated with the QMEAN scoring function, and its stereochemical correctness and all-atoms contacts were evaluated with the Molprobity software.

**Table 1.** Values of the parameters achieved in the structure quality evaluation of the different models of *Tc*CA.

Metric/model	HHPred raw	HHPred refined	Snap 17423	Snap 17423_refined
QMEAN4	−6.04	−1.60	−4.06	−0.96
Molprobity	3.57	1.83	2.04	1.81
Ramachandran allowed	95.4	98.1	95.8	98.1
Clash score	136.37	5.43	2.71	4.93

**Figure 2.**
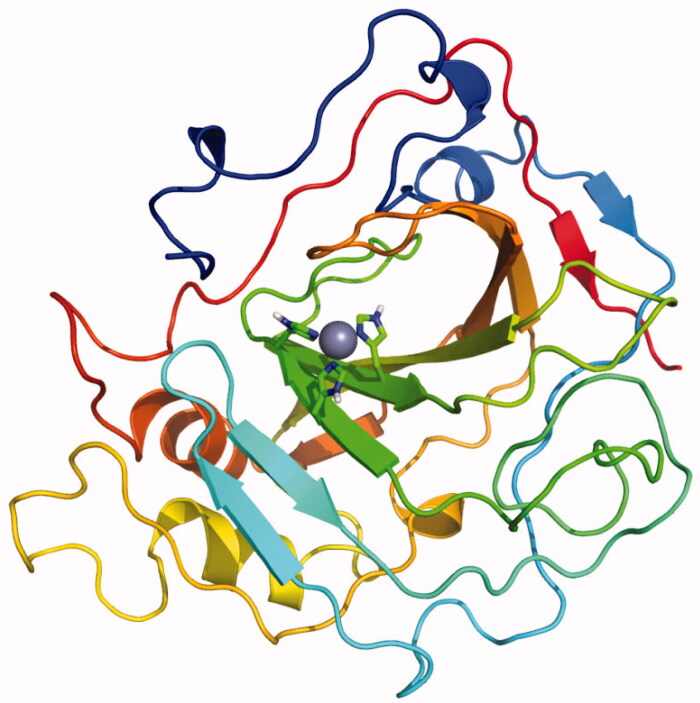
Final model of *Tc*CA achieved from multi-template comparative modelling and subsequent iterative Rosetta-MD refinement. Histidine residues of the active site are highlighted in green for carbon atoms and in blue for nitrogen atoms. The zinc ion is represented as a non-bonded sphere in grey.

The raw model produced by Modeller shows the poorest quality, whereas Rosettàs refined model of the best snapshot (17423_refined in Table 1) is the most correct in both geometric and energetic evaluations. The normalised QMEAN4 value for the final model is similar to the scores achieved in high resolution X-ray structures of comparable size, and the per-residue energy analysis shows that the problematic portions of the model are beyond the defined docking site (Figure S2). Figure 2 shows the overall architecture of the final *Tc*CA model, which was constructed based on the multi-template strategy and subsequently refined by iterative Rosetta-MD simulations. Coordinates of the model have been included as Supporting Information.

### Validation of the docking protocol for TcCA virtual screening

Once we obtained an ensemble of *Tc*CA models, the next step was the selection of the best docking conditions for the virtual screening. For that purpose, we analysed the ability of the software to correlate the docking scores with the p*K*_I_ values of the compounds of the validation set. We docked the validation set into the final Rosetta’s refined models of the target. Bootstrap sampling was applied to the data to calculate median values and its associated confidence intervals to compare between different models. The lowest energy pose from all the runs was chosen as the binding mode for each structure. Other criteria would be to consider the lowest energy pose from the most populated cluster, but we found that these conformations oriented the zinc-binding function outside the binding site for some compounds of the dataset.

We did not expect a perfect correlation between the *K*i values and the scores due to the known limitations of docking scoring functions to predict absolute binding energies, especially when using protein models instead of crystal structures. Moreover, there are experimental errors and biological variability inherent to all bioactivity data that impacts on the final correlations. Nevertheless, these metrics can be applied to compare between different protein/docking models to select the best one under different conditions.

**Figure 3.**
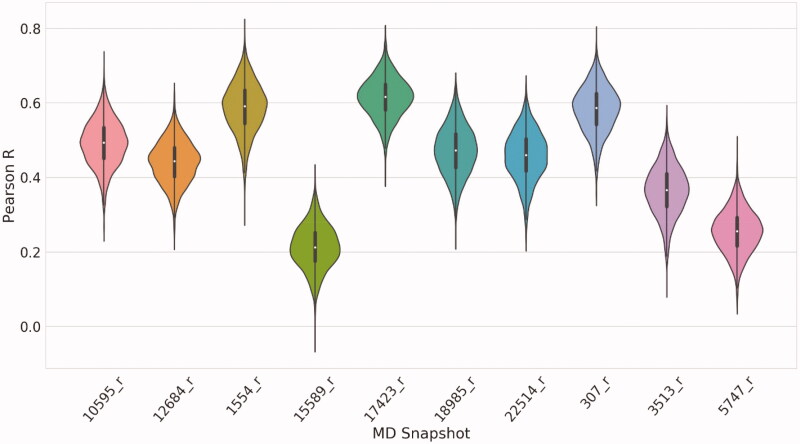
Pearson’s *R* obtained from docking the validation set on every refined MD snapshot. The white dot represents the median value, and the black line height represents the distance between the lower quartile (Q1) to the upper quartile (Q3), which is called the interquartile range (IQR). Violin plots show the probability density of the data at different values as a kernel density estimation.

Figure 3 shows the statistical parameters in a conditional violin plot for every protein model, considering Pearson’s *R* between the docking scores and the p*K*_I_ values. According to the results, we decided to use as target model the snapshot 17423_r, since it achieved the best correlation metrics for the structures of the validation set. All the metrics calculated for every protein model are summarised in Table S3.

**Figure 4.**
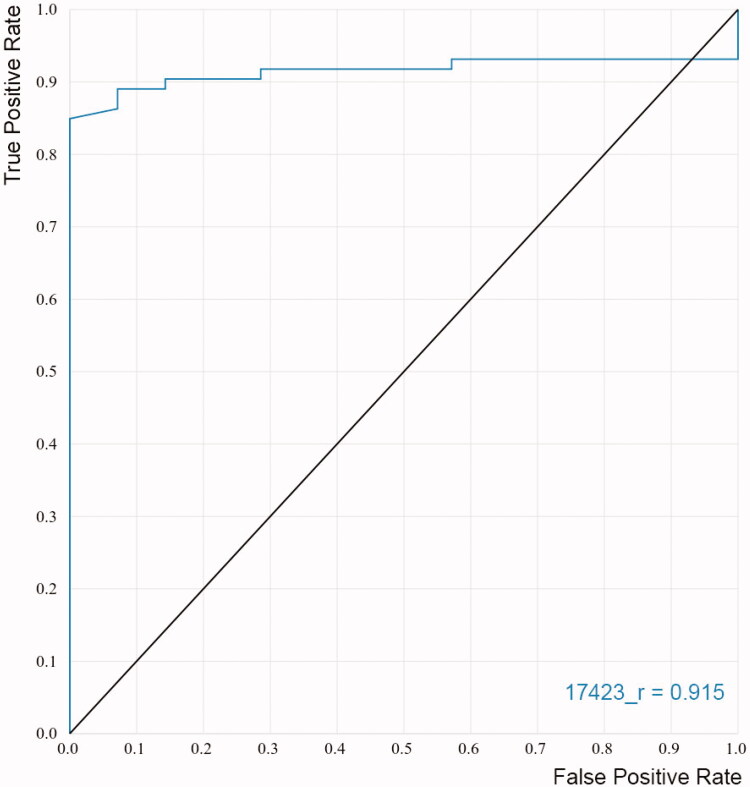
ROC curve obtained for the final model applied in the virtual screening.

Additionally, ROC curves were constructed to evaluate the capacity of the models to discriminate known inhibitors from non-inhibitors. The ROC curves plot the sensitivity of the model (true positive rate) as a function of the false positive rate (1 − specificity) at various threshold settings. It could be interpreted as the probability that a classifier (like the docking score) ranks a randomly chosen active compound higher than a randomly chosen inactive one^77^^,^^78^. Accordingly, a perfect classification of compounds would be represented in the graph by a line that starts from the origin, reaches vertically the upper left corner, and then goes to the upper right corner^78^. The area under the curve (AUC) will be equal to one for this ideal performance while an AUC of 0.5 will represent a random selection of active compounds. Figure 4 shows the ROC curve obtained for the best docking model selected for the virtual screening. The black line represents a random classifier whose AUC-ROC is 0.5, whereas the AUC-ROC for our model is 0.915, which is very close to the optimal value.

**Figure 5.**
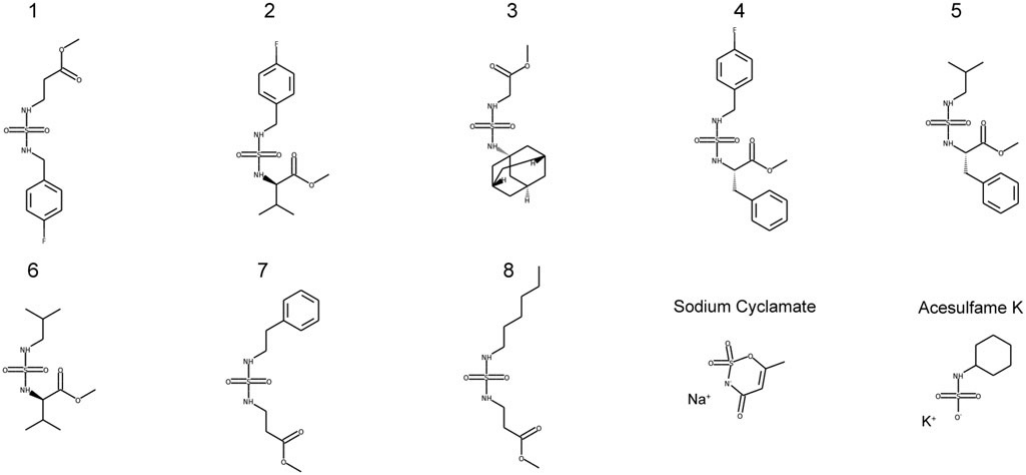
Candidates selected from the virtual screening.

The virtual screening of our database with the refined model 17423_r identified 42 structures as possible *Tc*CA inhibitors. Among them, 10 compounds were selected for biological evaluation (Figure 5). The criteria of choice were the docking scores and the chemical structure of the candidates. It is important to note that human examination of the hits is highly recommended for the selection of the candidates, since it helps to reduce false positives due to docking score artefacts and can lead to the discovery of more potent hits than score prioritisation alone. Compounds 1–8 are aminoester-derived sulphamides with different substituents in the second nitrogen atom of the sulphamide function. These compounds were tested before as human CA II inhibitors, with poor results^50–54^. The biological results against *Tc*CA of this family would give us the opportunity to get information about the structure-activity relationships. The set was completed with two artificial sweeteners, acesulphame and sodium cyclamate, which also have low potency as human CA II inhibitors and different scaffolds than the other selected candidates^47^^,^^48^. Additionally, they are commercially available compounds approved for human consumption.

### Biological assays

Table 2 shows the results of the biological assays against the enzyme and the parasite. Inhibition values against *Tc*CA were reported as inhibition constants (*K*_I_) and the corresponding values against human CA II were also included for comparison. The effects of the candidates with respect to the untreated controls against *T. cruzi* epimastigotes (Y-strain) and trypomastigotes (RA-strain) were also measured and included in the table.

**Table 2.** Biological data and docking scores achieved for compounds of the set.

Compound	*K*_I_*Tc*CA (nM)	K_I_ hCA II (nM)^a^	Selectivity ratio	Docking score	% Proliferation. Epimastigotes^b^		% Viability. Trypomastigotes 24 h^c^
					3rd day of growth	7th day of growth	
**1**	594.4	>10,000	>16.8	−8.38	86	92	73
**2**	604.6	>10,000	>16.5	−9.16	79	102	66
**3**	752.5	4957	>6.6	−9.30	107	94	91
**4**	950.5	>10,000	>10.5	−8.48	57	55	72
**5**	261.4	8528	>32.6	−9.13	73	96	105
**6**	951.8	>10,000	>10.5	−8.14	103	104	65
**7**	7250	8884	1.22	−8.79	103	121	76
**8**	448.8	>10,000	>22.3	−7.97	72	114	98
Acesulphame	2242	>20,000	>8.9	−7.05	92	89	101
Sodium cyclamate	348.1	>10,000	>28.7	−7.46	89	78	52

^a^Inhibition constants against hCAII were taken from literature^28–30^.

^b^Epimastigotes of the Y strain of *T. cruzi* (candidates tested at 50 µM).

^c^Trypomastigotes of the RA strain of *T. cruzi* (candidates tested at 20 µM).

Regarding the interaction with *Tc*CA, most of the structures showed interesting inhibitory effect against the enzyme, with K_I_ values in the nanomolar range. These results evidence the predictive capacity of the docking model to identify *Tc*CA inhibitors. Particularly, sulphamide **5** is the most potent inhibitor of the set, with a *K*_I_ value of 0.26 μM. Sodium cyclamate, a widely used sweetener, showed to be a potent *Tc*CA inhibitor too. Concerning selectivity, both compounds are about 30 times more active against the parasite target than the human CA II, which represent an important aspect to be considered in future investigations.

**Figure 6.**
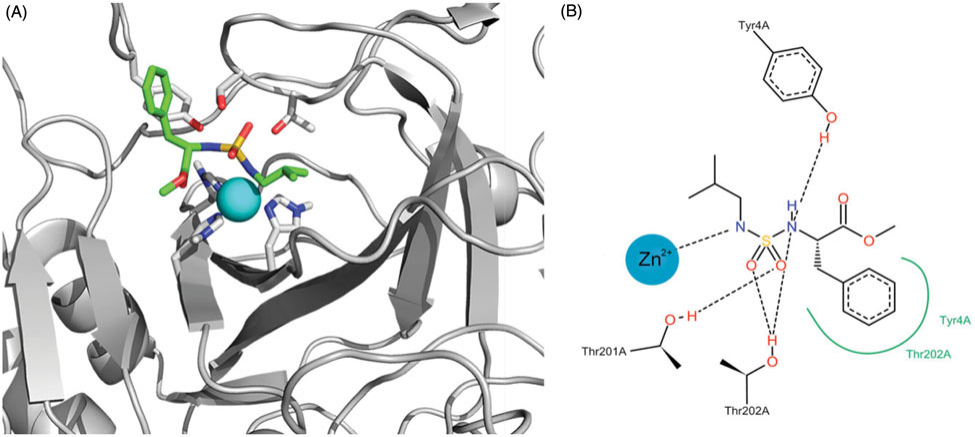
(A) Best docking solution for the interaction of compound **5** within the active site of *Tc*CA. Histidine and important residues of the active site are highlighted in grey for carbon atoms, in red for oxygen atoms and in blue for nitrogen atoms. The zinc ion is represented as a non-bonded sphere in cyan. Colour code for compound **5**: Carbon atoms in green, Nitrogen atoms in blue, sulphur atoms in yellow and oxygen atoms in red. (B) Schematic representation of the interactions between compound **5** and the active site of the *Tc*CA model.

Figure 6 shows the representation of the binding interaction of compound **5** with the active site. The docking programme orients one nitrogen atom of the sulphamide group towards the zinc ion, whereas oxygen atoms acts as hydrogen bond acceptors for THR201 and THR202 sidechain hydroxyl groups. Similar interactions were achieved between the second nitrogen atom of the sulphamide and the Tyr4 side chain. This residue is also involved in positive aromatic interactions with the inhibitor.

**Figure 7.**
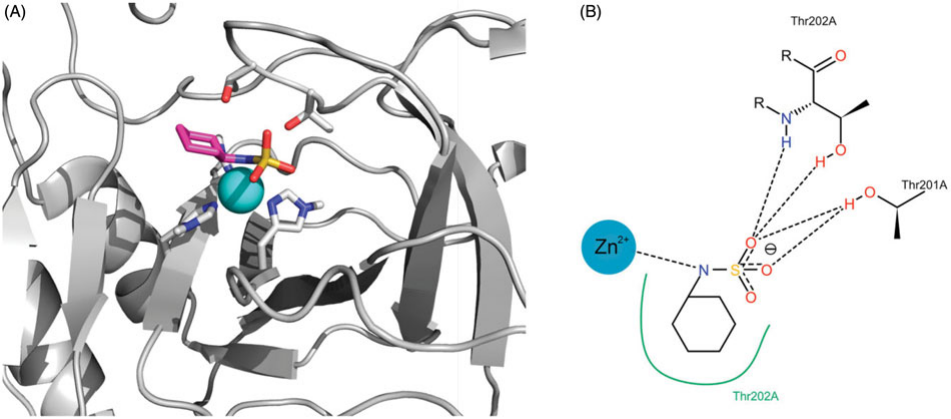
(A) Best docking solution for the interaction of cyclamate with the active site of *Tc*CA. Histidine and important residues of the active site are highlighted in grey for carbon atoms, in red for oxygen atoms and in blue for nitrogen atoms. The zinc ion is represented as a non-bonded sphere in cyan. Colour code for cyclamate: Carbon atoms in violet, Nitrogen atoms in blue, sulphur atoms in yellow and oxygen atoms in red. (B) Schematic representation of the interactions between cyclamate and the active site of the *Tc*CA model.

Similarly, Figure 7 shows the representation of the interaction proposed by docking between cyclamate and the active site of *Tc*CA. The nitrogen atom of the sulphamate function interacts with the Zinc ion and their oxygen atoms behave as hydrogen bonding acceptors from the Thr residues of the active site. The cyclohexyl substituent attached to the N atom contributes to the binding through hydrophobic interactions.

The effect of the candidates against *T. cruzi* epimastigotes, the non-infective and proliferative stage, was tested at 50 µM (Table 2). Among the best two *Tc*CA inhibitors of the set, sodium cyclamate showed 20% of inhibition on the proliferation of epimastigotes at the tested concentration (7th day). When sodium cyclamate was tested against trypomastigote, one of the clinically relevant stages of *T. cruzi*, this candidate inhibited parasite viability by 50% at 20 µM.

Other compounds also showed good inhibition profiles against both forms of the parasite, but they were poorer *Tc*CA inhibitors. The lack of correlation between the inhibitory effects on the enzyme and the trypanocidal effects on the parasite may suggest that other mechanisms of action besides CA inhibition are present. Other possible explanations to the discrepancy may relate with the drugs uptake kinetics by the parasite and/or biotransformation/inactivation of the candidates.

## Conclusions

We report the results of a target-based virtual screening for the discovery of new inhibitors of *Tc*CA with poor interaction with hCA II. As the experimental structure of the target is not available, we turned to the careful construction of multi-template three-dimensional models, to select the best macrostructure for docking simulation. The iterative combination of Rosetta and MD simulations allowed us to significantly improve the quality of the starting model, as reflected by the reported metrics. It is important to note that we have achieved target models with high quality despite the low sequence identity of the templates found for their construction.

The docking calculations were fully validated through a test set of compounds with reported activity against *Tc*CA, which led us to find a structural model with the best scoring power. By the application of the model in a virtual screening campaign we identified sulphamides with high potency and selectivity against the ubiquitous human CAII isoform. Additionally, the model selected two commercial and widely used artificial sweeteners with abundant toxicological data available. They share the sulphamate function, a bioisosteric partner of sulphamide as zinc-binding function. The assays in parasites identified sodium cyclamate as the most promising structure in terms of trypanocidal activity, reducing trypomastigote viability by 48% at 20 µM. Further molecular modelling and SAR studies will be performed in future investigations, to achieve a deeper knowledge about the molecular determinants of the potency and selectivity against *Tc*CA.

## Supplementary Material

Supplemental Material
