# Supplementary material for: A structure-based approach towards the identification of novel antichagasic compounds: Trypanosoma cruzi carbonic anhydrase inhibitors
Source: J Enzyme Inhib Med Chem. 2019 Oct 16;35(1):21–30. doi: 10.1080/14756366.2019.1677638 (PMC6807911; doi:10.1080/14756366.2019.1677638)
Supplement: Supplemental Material [file IENZ_A_1677638_SM5177.pdf]

Figure S1

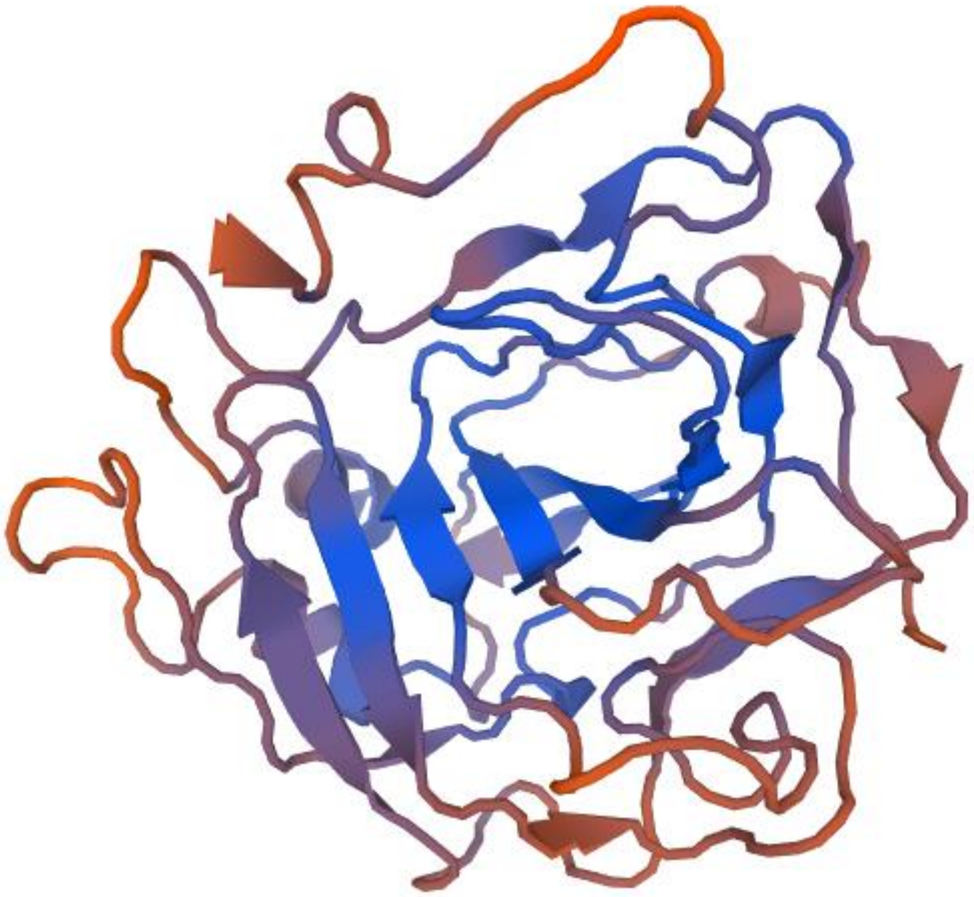

Table S1

| SMILES | pKi | Docking Score |
|---|---|---|
| COc1ccc(CC(=O)[N-]c2nnc(S([NH-])(=O)=O)s2)cc1 | 9.29 | -9.25 |
| [NH-]S(=O)(=O)c1nnc([N-]C(=O)Cc2ccccc2Br)s1 | 9.08 | -10.49 |
| [NH-]S(=O)(=O)c1nnc([N-]C(=O)Cc2ccc(Cl)cc2)s1 | 9.02 | -13.41 |
| NS(=O)(=O)c1ccc(NC(=O)Cc2ccc(Cl)cc2)c(F)c1 | 8.92 | -9.58 |
| NS(=O)(=O)c1ccc(NC(=O)Cc2ccccc2Br)c(F)c1 | 8.80 | -10.47 |
| NS(=O)(=O)c1ccc(NC(=O)Cc2ccc(Cl)cc2)cc1 | 8.57 | -9.63 |
| NS(=O)(=O)c1ccc(CCNC(=O)Cc2ccccc2Br)cc1 | 8.48 | -10.98 |
| NS(=O)(=O)c1ccc(CNC(=O)Cc2ccc(F)cc2)cc1 | 8.43 | -10.32 |
| COc1ccc(CC(=O)Nc2ccc(S(N)(=O)=O)cc2F)cc1 | 8.39 | -9.83 |
| NS(=O)(=O)c1ccc(NC(=O)Cc2ccc(F)cc2)c(Cl)c1 | 8.15 | -9.35 |
| NS(=O)(=O)c1ccc(NC(=O)Cc2ccc(Cl)cc2)c(Br)c1 | 8.15 | -9.74 |
| COc1ccc(CC(=O)Nc2ccc(S(N)(=O)=O)cc2Br)cc1 | 8.14 | -9.99 |
| COc1ccc(CC(=O)NCc2ccc(S(N)(=O)=O)cc2)cc1 | 8.13 | -9.93 |
| COc1ccc(CC(=O)NCCc2ccc(S(N)(=O)=O)cc2)cc1 | 8.12 | -9.57 |
| NS(=O)(=O)c1nnc(NC(=O)Cc2ccc(F)cc2)s1 | 8.12 | -11.81 |
| NS(=O)(=O)c1ccc(NC(=O)Cc2ccc(Cl)cc2)c(Cl)c1 | 8.11 | -10.68 |
| NS(=O)(=O)c1ccc(NC(=O)Cc2ccccc2Br)c(Cl)c1 | 8.11 | -9.57 |
| COc1ccc(CC(=O)Nc2ccc(S(N)(=O)=O)cc2Cl)cc1 | 8.10 | -10.06 |
| COc1ccc(CC(=O)Nc2ccc(S(N)(=O)=O)cc2)cc1 | 8.10 | -9.97 |
| NS(=O)(=O)c1ccc(NC(=O)Cc2ccc(F)cc2)c(F)c1 | 8.08 | -9.32 |
| NS(=O)(=O)c1ccc(NC(=O)Cc2ccc(F)cc2)cc1 | 8.07 | -9.35 |
| NS(=O)(=O)c1ccc(NC(=O)Cc2ccccc2Br)cc1 | 8.05 | -9.76 |
| NS(=O)(=O)c1ccc(NC(=O)Cc2ccc(F)cc2)c(Br)c1 | 8.05 | -9.49 |
| NS(=O)(=O)c1ccc(CNC(=O)Cc2ccccc2Br)cc1 | 8.05 | -10.81 |
| NS(=O)(=O)c1ccc(CCNC(=O)Cc2ccc(F)cc2)cc1 | 8.04 | -10.36 |
| NS(=O)(=O)c1ccc(NC(=O)Cc2ccccc2Br)c(Br)c1 | 7.96 | -9.64 |
| NS(=O)(=O)c1ccc(CCNC(=O)Cc2ccc(Cl)cc2)cc1 | 7.93 | -10.29 |
| NS(=O)(=O)c1ccc(CNC(=O)Cc2ccc(Cl)cc2)cc1 | 7.90 | -10.63 |
| O=C(N/N=C/c1ccccc1)Nc1n[n-]c(=S)s1 | 7.68 | -9.96 |
| COc1cccc(/C=N/NC(=O)Nc2n[n-]c(=S)s2)c1 | 7.46 | -9.99 |
| O=C(NO)[C@H]1CC(c2ccc(OCc3ccccc3)cc2)=NO1 | 7.40 | -11.43 |
| O=C(N/N=C/c1cccc(Br)c1)Nc1n[n-]c(=S)s1 | 7.37 | -10.48 |
| CC(C)C/C=N\NC(=O)Nc1n[n-]c(=S)s1 | 7.28 | -8.6 |
| CC(=O)Nc1nnc(S(N)(=O)=O)s1 | 7.21 | -9.41 |
| O=C(N/N=C/c1ccc(Cl)cc1)Nc1n[n-]c(=S)s1 | 7.19 | -8.99 |
| CCOc1ccc(C2=NO[C@@H](C(=O)NO)C2)cc1 | 7.15 | -9.5 |
| NS(=O)(=O)c1ccc(N2CCCS2(=O)=O)cc1 | 7.14 | -9.08 |
| O=C(N/N=C1/C(=O)Nc2ccc(Br)cc21)Nc1n[n-]c(=S)s1 | 7.14 | -9.94 |
| CC(=O)/N=c1\sc(S(N)(=O)=O)nn1C | 7.13 | -7.8 |
| O=C(N/N=C1/C(=O)Nc2ccccc21)Nc1n[n-]c(=S)s1 | 7.10 | -9.97 |
| Cc1onc(-c2ccccc2)c1-c1ccc(S(N)(=O)=O)cc1 | 7.08 | -10.16 |
| NS(=O)(=O)c1ccc(S(=O)(=O)Nc2cccc3c(Cl)c[nH]c23)cc1 | 7.07 | -11.22 |
| CC1(C)O[C@@H]2CO[C@@]3(COS(N)(=O)=O)OC(C)(C)O[C@H]3[C@@H]2O1 | 7.07 | -8.76 |

| | | |
|---|---|---|
| CCN[C@H]1CN(CCCOC)S(=O)(=O)c2sc(S(N)(=O)=O)cc21 | 7.06 | -8.82 |
| CCN1CCC[C@H]1CNC(=O)c1cc(S(N)(=O)=O)ccc1OC | 7.06 | -10.26 |
| CCOc1ccc2nc(S(N)(=O)=O)sc2c1 | 7.05 | -8.94 |
| Nc1ccc(S(=O)(=O)Nc2nnc(S(N)(=O)=O)s2)cc1 | 7.05 | -10.47 |
| Cc1ccc(-c2cc(C(F)(F)F)nn2-c2ccc(S(N)(=O)=O)cc2)cc1 | 7.04 | -10.03 |
| CCN[C@H]1C[C@H](C)S(=O)(=O)c2sc(S(N)(=O)=O)cc21 | 7.03 | -10.19 |
| NS(=O)(=O)Cc1nnc(NS(=O)(=O)c2ccccc2)s1 | 7.03 | -11.63 |
| CCOc1ccccc1C1=NO[C@@H](C(=O)NO)C1 | 7.03 | -8.96 |
| C/C(=N\NC(=O)Nc1n[n-]c(=S)s1)c1ccc(Cl)cc1 | 7.02 | -9.54 |
| NNC(=O)Nc1n[n-]c(=S)s1 | 6.90 | -6.93 |
| NS(=O)(=O)c1cc(Cl)c(Cl)c(S(N)(=O)=O)c1 | 6.89 | -9.18 |
| NS(=O)(=O)c1ccc(CCNS(=O)(=O)c2ccc([N+](=O)[O-])cc2CO)cc1 | 6.87 | -10.66 |
| NS(=O)(=O)c1cc2c(cc1Cl)NCNS2(=O)=O | 6.87 | -9.19 |
| C[C@@]1(C(=O)NO)CC(c2ccccc2F)=NO1 | 6.85 | -7.97 |
| C[C@@]1(C(=O)NO)CC(c2cccc(F)c2)=NO1 | 6.74 | -7.92 |
| O=C(NO)[C@H]1CC(c2ccc(Cl)cc2)=NO1 | 6.72 | -9.66 |
| Nc1ccc(S(=O)(=O)NCCc2ccc(S(N)(=O)=O)cc2)cc1 | 6.72 | -11.65 |
| Nc1ccc(S(=O)(=O)NCc2ccc(S(N)(=O)=O)cc2)cc1 | 6.61 | -10.11 |
| O=C(NO)[C@H]1CC(c2ccccc2Cl)=NO1 | 6.58 | -9.39 |
| O=C(NO)[C@H]1CC(c2cccc(Cl)c2)=NO1 | 6.57 | -9.36 |
| Nc1ccc(S(=O)(=O)Nc2ccc(S(N)(=O)=O)cc2)cc1 | 6.44 | -10.01 |
| C[C@@]1(C(=O)NO)CC(c2cccc(Cl)c2)=NO1 | 6.44 | -8.17 |
| O=C(NO)[C@H]1CC(c2ccccc2)=NO1 | 6.37 | -9.17 |
| Nc1nnc(S(N)(=O)=O)s1 | 6.27 | -8.77 |
| C[C@@]1(C(=O)NO)CC(c2cccc(Br)c2)=NO1 | 6.21 | -8.29 |
| Cn1nc(S(N)(=O)=O)sc1=N | 6.19 | -7.93 |
| Nc1nccc(Nc2ccc(S(N)(=O)=O)cc2)n1 | 6.09 | -8.79 |
| Nc1cc(Cl)c(S(N)(=O)=O)cc1S(N)(=O)=O | 6.09 | -8.9 |
| Nc1cc(C(F)(F)F)c(S(N)(=O)=O)cc1S(N)(=O)=O | 6.07 | -8.52 |
| NS(=O)(=O)Cc1noc2ccccc12 | 6.06 | -9.89 |
| Nc1ccc(S(N)(=O)=O)cc1Cl | 5.16 | -8.18 |
| NCc1ccc(S(N)(=O)=O)cc1 | 5.14 | -8.46 |
| Nc1ccc(S(N)(=O)=O)cc1F | 5.09 | -8 |
| Nc1ccc(S(N)(=O)=O)cc1Br | 5.07 | -8.27 |
| NCCc1ccc(S(N)(=O)=O)cc1 | 5.03 | -8.51 |
| NS(=O)(=O)c1cc(Cl)cc(Cl)c1O | 5.03 | -8.53 |
| Nc1cccc(S(N)(=O)=O)c1 | 4.59 | -8.22 |
| Cc1ccc(S(N)(=O)=O)cc1 | 4.35 | -8.14 |
| Nc1ccc(S(N)(=O)=O)cc1 | 4.24 | -8.12 |
| NS(=O)(=O)c1cccc(S(N)(=O)=O)c1 | 4.20 | -8.77 |
| NS(=O)(=O)c1ccc(CCO)cc1 | 4.18 | -8.02 |
| NS(=O)(=O)c1ccc(CO)cc1 | 4.14 | -8.16 |
| NS(=O)(=O)c1ccc(O)cc1 | 4.13 | -8.01 |
| NS(=O)(=O)c1ccc(C(=O)O)cc1 | 4.08 | -8.19 |

Table S2

| SMILES | Docking Score |
|---|---|
| O=C(Nc1ccc(S(=O)(=O)[N-]c2nccs2)cc1)c1ccccc1C(=O)c1ccccc1 | -11.1 |
| C(=Cc1ccccc1)C[N@H+]1CC[N@@H+](C(c2ccccc2)c2ccccc2)CC1 | -11.01 |
| Cc1cn(-c2cc(NC(=O)c3ccc(C)c(Nc4nccc(-c5cccnc5)n4)c3)cc(C(F)(F)F)c2)cn1 | -10.81 |
| c1ccc(-c2ccc([C@H](c3ccccc3)n3ccnc3)cc2)cc1 | -10.76 |
| CCCc1nn(C)c2c(=O)nc(-c3cc(S(=O)(=O)N4CC[NH+](C)CC4)ccc3OCC)[nH]c12 | -10.43 |
| O=C(Nc1ccc(S(=O)(=O)[N-]c2ncccn2)cc1)C(c1ccccc1)c1ccccc1 | -10.41 |
| O=C([O-])c1cc(/N=N/c2ccc(S(=O)(=O)Nc3ccccn3)cc2)ccc1O | -10.32 |
| Cc1ccc(C)c(OCCCC(C)(C)C(=O)Nc2ccc(S(=O)(=O)[N-]c3ncccn3)cc2)c1 | -10.29 |
| O=C(Nc1ccc(S(=O)(=O)[N-]c2ncccn2)cc1)c1ccccc1C(=O)c1ccccc1 | -10.29 |
| Cc1ccc(-c2cc(C(F)(F)F)nn2-c2ccc(S(N)(=O)=O)cc2)cc1 | -10.18 |
| CC(C)(O)c1ccccc1CC[C@@H](SCC1(CC(=O)[O-])CC1)c1cccc(/C=C/c2ccc3ccc(Cl)cc3n2)c1 | -9.94 |
| Clc1ccc(CO[C@H](Cn2ccnc2)c2ccc(Cl)cc2Cl)cc1 | -9.86 |
| C#C[C@]1(O)CC[C@H]2[C@@H]3CCC4=Cc5oncc5C[C@]4(C)[C@H]3CC[C@@]21C | -9.83 |
| Clc1ccc(CO[C@H](Cn2ccnc2)c2ccc(Cl)cc2Cl)c(Cl)c1 | -9.83 |
| CC[C@@H](C)n1ncn(-c2ccc(N3CCN(c4ccc(OC[C@H]5CO[C@](Cn6cncn6)(c6ccc(Cl)cc6Cl)O5)cc4)CC3)cc2)c1=O | -9.81 |
| Nc1ccccc1C(=O)Nc1ccc(S(=O)(=O)[N-]c2nccs2)cc1 | -9.69 |
| O=C(NC1CCCCC1)c1cccnc1 | -9.65 |
| O=S(=O)(NCCc1ccccc1)NCCc1ccccc1 | -9.64 |
| O=C(Nc1ccc(S(=O)(=O)[N-]c2nccs2)cc1)c1cccc([N+](=O)[O-])c1 | -9.64 |
| CC(C)C[C@H]1C(=O)N2CCC[C@H]2[C@]2(O)O[C@](NC(=O)[C@@H]3C=C4c5cccc6[nH]c(Br)c(c56)C[C@H]4[N@@H+](C)C3)(C(C)C)C(=O)N12 | -9.59 |
| COc1ccc(CC[N@@H+](C)CCC[C@](C#N)(c2ccc(OC)c(OC)c2)C(C)C)cc1OC | -9.57 |
| Clc1ccccc1C(c1ccccc1)(c1ccccc1)n1ccnc1 | -9.56 |
| O=C(Nc1ccc(S(=O)(=O)[N-]c2ncccn2)cc1)c1ccccc1Cl | -9.54 |
| Nc1ccc(S(=O)(=O)Nc2ncccn2)cc1 | -9.53 |
| Nc1ccccc1C(=O)Nc1ccc(S(=O)(=O)[N-]c2ncccn2)cc1 | -9.53 |
| Nc1cccc(C(=O)Nc2ccc(S(=O)(=O)[N-]c3nccs3)cc2)c1 | -9.51 |
| CSc1nc2cc(Oc3cccc(Cl)c3Cl)c(Cl)cc2[nH]1 | -9.49 |
| O=S(=O)(NC1CCCCC1)NC1CCCCC1 | -9.47 |
| CC(C)(C)C(=O)Nc1ccc(S(=O)(=O)[N-]c2ncccn2)cc1 | -9.45 |
| O=C(Nc1ccc(S(=O)(=O)[N-]c2nccs2)cc1)C(c1ccccc1)c1ccccc1 | -9.43 |
| CC(=O)N1CCN(c2ccc(OC[C@H]3CO[C@](Cn4ccnc4)(c4ccc(Cl)cc4Cl)O3)cc2)CC1 | -9.41 |
| O=C(Nc1ccc(S(=O)(=O)[N-]c2nccs2)cc1)c1ccccc1 | -9.38 |
| NS(=O)(=O)c1cc2c(cc1Cl)NCNS2(=O)=O | -9.31 |

| | |
|---|---|
| O=C(Nc1ccc(S(=O)(=O)[N-]c2ncccn2)cc1)c1ccccc1 | -9.3 |
| O=C(Nc1ccc(S(=O)(=O)[N-]c2nccs2)cc1)c1ccccc1Cl | -9.29 |
| O=C(Nc1ccc(S(=O)(=O)[N-]c2ncccn2)cc1)c1cccc([N+](=O)[O-])c1 | -9.28 |
| O=C(Nc1ccc(S(=O)(=O)[N-]c2nccs2)cc1)c1ccc(Cl)cc1 | -9.22 |
| CCCC(=O)Nc1ccc(S(=O)(=O)[N-]c2ncccn2)cc1 | -9.2 |
| Nc1cccc(C(=O)Nc2ccc(S(=O)(=O)[N-]c3ncccn3)cc2)c1 | -9.18 |
| CC(C)C(=O)Nc1ccc(S(=O)(=O)[N-]c2ncccn2)cc1 | -9.16 |
| CC(C)(C)C(=O)Nc1ccc(S(=O)(=O)[N-]c2nccs2)cc1 | -9.14 |
| COC(=O)CCCNS(=O)(=O)NCc1ccccc1 | -9.12 |
| O=S(=O)(NCCc1ccccc1)Oc1ccccc1O | -9.1 |
| COC(=O)CCNS(=O)(=O)NCCc1ccccc1 | -9.1 |
| CCCSc1ccc2nc(NC(=O)OC)[nH]c2c1 | -9.09 |
| CCC(=O)Nc1ccc(S(=O)(=O)[N-]c2nccs2)cc1 | -9.07 |
| Cc1ccc(C)c(OCCCC(C)(C)C(=O)Nc2ccc(S(=O)(=O)[N-]c3nccs3)cc2)c1 | -9.07 |
| CCCC(CCC)C(=O)Nc1ccc(S(=O)(=O)[N-]c2nccs2)cc1 | -9.07 |
| O=[N+]([O-])c1nccn1CC(O)=NCc1ccccc1 | -9.02 |
| CCC(=O)Nc1ccc(S(=O)(=O)[N-]c2ncccn2)cc1 | -9.01 |
| CCCCC(=O)Nc1ccc(S(=O)(=O)[N-]c2ncccn2)cc1 | -9 |
| CC(=O)Nc1ccc(S(=O)(=O)[N-]c2nccs2)cc1 | -8.99 |
| COC(=O)CCNS(=O)(=O)NCC12C[C@H]3C[C@@H](C1)C[C@@H](C 2)C3 | -8.97 |
| CC(=O)Nc1ccc(S(=O)(=O)[N-]c2ncccn2)cc1 | -8.91 |
| CCCC(=O)Nc1ccc(S(=O)(=O)[N-]c2nccs2)cc1 | -8.86 |
| O=C(Nc1ccc(S(=O)(=O)[N-]c2ncccn2)cc1)c1ccc(Cl)cc1 | -8.85 |
| CCCCC(=O)Nc1ccc(S(=O)(=O)[N-]c2nccs2)cc1 | -8.84 |
| CCCCc1ncc(/C=C(\Cc2cccs2)C(=O)[O-])n1Cc1ccc(C(=O)[O-])cc1 | -8.81 |
| O=C(Nc1ccc(S(=O)(=O)[N-]c2ncccn2)cc1)c1cc([N+](=O)[O- ])cc([N+](=O)[O-])c1 | -8.81 |
| O=C(Nc1ccc(S(=O)(=O)[N-]c2nccs2)cc1)c1cc([N+](=O)[O- ])cc([N+](=O)[O-])c1 | -8.8 |
| CCCC(=O)Nc1ccc(S(=O)(=O)[N-]c2nccs2)cc1 | -8.75 |
| CCCC(CCC)C(=O)Nc1ccc(S(=O)(=O)[N-]c2ncccn2)cc1 | -8.68 |
| Nc1ccc(S(=O)(=O)[N-]c2nccs2)cc1 | -8.68 |
| O=C(Nc1ccc(S(=O)(=O)[N-]c2ncccn2)cc1)c1ccc([N+](=O)[O-])cc1 | -8.6 |
| Fc1ccc([C@@H]2CC[NH2+]C[C@H]2COc2ccc3c(c2)OCO3)cc1 | -8.56 |
| COc1ccc(C(=O)Nc2ccc(S(=O)(=O)[N-]c3nccs3)cc2)cc1 | -8.56 |
| COC(=O)CCCNS(=O)(=O)N[C@@H](C)c1ccccc1 | -8.54 |
| C[C@H](NC(=O)C(C)(C)OC(C)(C)C(=O)N[C@@H](C)c1ccccc1)c1cccc c1 | -8.53 |
| COC(=O)CC[C@H](NS(=O)(=O)NCCc1ccccc1)C(=O)OC | -8.5 |
| C=C1[C@@H](n2cnc3c(=O)nc(N)[nH]c32)C[C@H](O)[C@H]1CO | -8.46 |
| CC(C)CNS(=O)(=O)NCC(C)C | -8.45 |
| O=C(Nc1ccc(S(=O)(=O)[N-]c2ncccn2)cc1)c1ccc(O)cc1 | -8.43 |
| CCCCCCNS(=O)(=O)NCCCCCC | -8.41 |
| COC(=O)CCCNS(=O)(=O)NCCc1ccccc1 | -8.39 |
| O=S(=O)(NCc1ccc(F)cc1)Oc1ccccc1O | -8.36 |
| Cc1ccc(C)c(OCCCC(C)(C)C(=O)Nc2c(C)cccc2C)c1 | -8.33 |

| | |
|---|---|
| C#C[C@]1(O)CC[C@H]2[C@@H]3CCc4cc(OC5CCCC5)ccc4[C@H]3CC[C@@]21C | -8.32 |
| COc1ccc(C(=O)Nc2ccc(S(=O)(=O)[N-]c3ncccn3)cc2)cc1 | -8.24 |
| COC(=O)[C@H](Cc1ccccc1)NS(=O)(=O)NCC(C)C | -8.23 |
| COC(=O)CCNS(=O)(=O)NCc1ccc(F)cc1 | -8.23 |
| CCCCc1oc2ccccc2c1C(=O)c1cc(I)c(OCC[NH+](CC)CC)c(I)c1 | -8.2 |
| O=C(Nc1ccc(S(=O)(=O)[N-]c2nccs2)cc1)c1ccc(O)cc1 | -8.19 |
| CCOC(=O)[C@H](CCc1ccccc1)[NH2+][C@@H](C)C(=O)N1CCC[C@H]1C(=O)[O-] | -8.18 |
| Cc1cccc(C)c1OC[C@H](C)NC(=O)[C@@H]1CCCN1C(=O)[C@H](C)[NH2+][C@@H](CCc1ccccc1)C(=O)[O-] | -8.18 |
| Nc1ccc(S(N)(=O)=O)cc1 | -8.17 |
| O=C(/C=C/c1ccc(O)c(O)c1)Nc1ccc(S(=O)(=O)[N-]c2ncccn2)cc1 | -8.14 |
| COC(=O)CCCNS(=O)(=O)NC(=O)[C@H]([NH3+])CC(C)C | -8.13 |
| Cn1cc(NC(=O)C(O)(c2ccccc2)c2ccccc2)c(=O)n1-c1ccccc1 | -8.12 |
| CCCNC(=O)c1cccnc1 | -8.08 |
| COC(=O)CC[C@@H](NS(=O)(=O)NCc1ccccc1)C(=O)OC | -8.07 |
| CCOC(=O)[C@H](CCc1ccccc1)[NH2+][C@H]1CCc2ccccc2N(CC(=O)[O-])C1=O | -8.07 |
| COC(=O)CCNS(=O)(=O)N[C@@H](C)c1ccccc1 | -8.07 |
| COC(=O)CCCNS(=O)(=O)N[C@H](C(=O)OC)C(C)C | -8.05 |
| Cc1ccccc1-c1cc(N2CC[NH+](C)CC2)ncc1N(C)C(=O)C(C)(C)c1cc(C(F)(F)F)cc(C(F)(F)F)c1 | -8.05 |
| C[C@@H](NC(=O)C(C)(C)OC(C)(C)C(=O)N[C@H](C)c1ccccc1)c1ccccc1 | -8.04 |
| Cc1cccc(C)c1NC(=O)[C@@H]1CCCN1C(=O)[C@H](C)[NH2+][C@@H](CCc1ccccc1)C(=O)[O-] | -8.03 |
| CCCCN(CCCC)C(=O)c1cccnc1 | -8.03 |
| CC(C)NS(=O)(=O)NC(C)C | -8.02 |
| CCCCNS(=O)(=O)NCCCC | -8.01 |
| C[C@H](NC(=O)C(C)(C)OC(C)(C)C(=O)N[C@H](C)c1ccccc1)c1ccccc1 | -8 |
| COC(=O)[C@H](NS(=O)(=O)NCC(C)C)C(C)C | -7.97 |
| O=C(Nc1ccc(S(=O)(=O)[N-]c2nccs2)cc1)c1ccc([N+](=O)[O-])cc1 | -7.96 |
| O=C(Nc1ccc(S(=O)(=O)[N-]c2nccs2)cc1)c1cc(O)c(O)c(O)c1 | -7.93 |
| CCCNS(=O)(=O)NCCC | -7.92 |
| O=C(Nc1ccc(S(=O)(=O)[N-]c2ncccn2)cc1)c1cc(O)c(O)c(O)c1 | -7.91 |
| CCCCCCNS(=O)(=O)NCCCC(=O)OC | -7.9 |
| CCCCCCNS(=O)(=O)NCCC(=O)OC | -7.85 |
| Cc1ccc(C)c(OCCCC(C)(C)C(=O)NCC2CCCCC2)c1 | -7.81 |
| CC(C)N=c1cc2n(-c3ccc(Cl)cc3)c3ccccc3nc-2cc1Nc1ccc(Cl)cc1 | -7.74 |
| COC(=O)[C@H](Cc1ccccc1)NS(=O)(=O)NCc1ccc(F)cc1 | -7.74 |
| CCCCNS(=O)(=O)NCCCC(=O)OC | -7.69 |
| COC(=O)CCCNS(=O)(=O)N[C@@H](Cc1ccccc1)C(=O)OC | -7.67 |
| OC(Cn1cncn1)(Cn1cncn1)c1ccc(F)cc1F | -7.66 |
| COC(=O)[C@@H](NS(=O)(=O)NCc1ccc(F)cc1)C(C)C | -7.64 |
| COC(=O)CCCNS(=O)(=O)NCC(C)C | -7.63 |
| Cc1ccc(C)c(OCCCC(C)(C)C(=O)N[C@@H](C)COc2c(C)cccc2C)c1 | -7.63 |

| | |
|---|---|
| C[C@H](NC(=O)COCC(=O)N[C@H](C)c1ccccc1)c1ccccc1 | -7.59 |
| Cc1ccccc1CNC(=O)C(C)(C)OC(C)(C)C(=O)NCc1ccccc1C | -7.58 |
| Cc1ccc(C)c(OCCCC(C)(C)C(=O)NCc2ccccc2)c1 | -7.58 |
| COC(=O)CCNS(=O)(=O)NCC(C)C | -7.57 |
| COC(=O)CCCNS(=O)(=O)NCC(N)=O | -7.55 |
| COC(=O)CC[C@@H](NS(=O)(=O)NCC(C)C)C(=O)OC | -7.55 |
| CC(C)(OC(C)(C)C(=O)NCc1ccccc1)C(=O)NCc1ccccc1 | -7.52 |
| CC(C)(OC(C)(C)C(=O)NCCc1ccccc1)C(=O)NCCc1ccccc1 | -7.46 |
| C[NH2+][C@@]1(c2ccccc2Cl)CCCCC1=O | -7.45 |
| CCCC(CCC)C(=O)OC(C)(C)C(=O)Nc1c(C)cccc1C | -7.44 |
| Cc1ccccc1CNC(=O)COCC(=O)NCc1ccccc1C | -7.4 |
| C[C@H]([NH2+][C@@H](CCc1ccccc1)C(=O)[O-])C(=O)N1CCC[C@H]1C(=O)[O-] | -7.36 |
| CC(=O)NCCc1c[nH]cn1 | -7.36 |
| C[C@H](NC(=O)C(C)(C)OC(=O)c1ccc(O)cc1)c1ccccc1 | -7.29 |
| C[C@@H](NC(=O)/C=C/c1ccc(O)c(O)c1)c1ccccc1 | -7.27 |
| C[C@H](NC(=O)/C=C/c1ccc(O)c(O)c1)c1ccccc1 | -7.27 |
| COc1cc(N)c(Cl)cc1C(=O)N[C@@H]1CC[N@H+](CCCOc2ccc(F)cc2)C[C@@H]1OC | -7.26 |
| COC(=O)CC[C@@H](NS(=O)(=O)NCC(N)=O)C(=O)OC | -7.25 |
| Cc1ccc(C)c(OCCCC(C)(C)C(=O)NCCc2ccccc2)c1 | -7.25 |
| C[C@H](NC(=O)C(C)(C)OC(=O)c1ccc(O)cc1)c1ccccc1 | -7.25 |
| CCCC(CCC)C(=O)OC(C)(C)C(=O)N[C@H](C)c1ccccc1 | -7.24 |
| CC[NH+](CC)CCOC(=O)C1(C2CCCCC2)CCCCC1 | -7.22 |
| CCCC(CCC)C(=O)OC(C)(C)C(=O)NCC1CCCCC1 | -7.21 |
| CCCCCCNS(=O)(=O)N[C@H](CCC(=O)OC)C(=O)OC | -7.19 |
| COc1ccc2c(c1)c(CC(=O)[O-])c(C)n2C(=O)c1ccc(Cl)cc1 | -7.17 |
| Nc1nc(=O)c2ncn(COCCO)c2[nH]1 | -7.16 |
| COC(=O)CC[C@@H](NS(=O)(=O)N[C@H](C)c1ccccc1)C(=O)OC | -7.15 |
| CN1C(=O)[C@]2(NC(=O)c3ccccc3N2)c2ccccc21 | -7.15 |
| COC(=O)CCCNS(=O)(=O)NCC(N)=O | -7.14 |
| Cc1cccc(C)c1OC[C@H](C)NC(=O)/C=C/c1ccc(O)c(O)c1 | -7.14 |
| CC[NH+](CC)CC(=O)Nc1c(C)cccc1C | -7.13 |
| C[C@@H](NC(=O)c1ccccc1)c1ccccc1 | -7.11 |
| O=C(NCCc1ccccc1)c1ccc(F)cc1 | -7.09 |
| CC(C)[NH2+]C[C@H](O)COc1cccc2ccccc12 | -7.09 |
| C[C@H](NC(=O)c1ccc(F)cc1)c1ccccc1 | -7.07 |
| O=C(/C=C/c1ccc(O)c(O)c1)NCCc1ccccc1 | -7.05 |
| O=C(COCC(=O)NCc1ccccc1)NCc1ccccc1 | -7.04 |
| CC(C)(OC(=O)c1ccc(O)cc1)C(=O)NCc1ccccc1 | -7.04 |
| O=C(NCCc1ccccc1)c1ccccc1 | -7.03 |
| CCCC(CCC)C(=O)OC(C)(C)C(=O)NCc1ccccc1 | -7.03 |
| COCCOC(=O)C1=C(C)NC(C)=C(C(=O)OC(C)C)[C@H]1c1cccc([N+](=O)[O-])c1 | -7.02 |
| COC(=O)CCCNS(=O)(=O)Oc1ccccc1O | -7.01 |
| COC(=O)CCCNS(=O)(=O)N[C@@H](C)C(=O)OC | -6.98 |
| O=C(COCC(=O)NCCc1ccccc1)NCCc1ccccc1 | -6.98 |

| | |
|---|---|
| CCCC(CCC)C(=O)OC(C)(C)C(=O)NCCc1ccccc1 | -6.94 |
| CCCCNC(=O)NS(=O)(=O)c1ccc(C)cc1 | -6.92 |
| CN1C(=O)CN=C(c2ccccc2)c2cc(Cl)ccc21 | -6.91 |
| CC(C)(OC(C)(C)C(=O)NCc1ccc(F)cc1)C(=O)NCc1ccc(F)cc1 | -6.9 |
| COC(=O)CCCNS(=O)(=O)NCC(=O)OC | -6.9 |
| COC(=O)CC[C@H](NS(=O)(=O)NC(=O)[C@H]([NH3+])CC(C)C)C(=O<br>)OC | -6.89 |
| COC(=O)CC[C@@H](NS(=O)(=O)NCC(N)=O)C(=O)OC | -6.88 |
| CCCNC(=O)NS(=O)(=O)c1ccc(Cl)cc1 | -6.88 |
| O=C1NC(=O)C(c2ccccc2)(c2ccccc2)N1 | -6.88 |
| O=C(COCC(=O)NCc1ccc(F)cc1)NCc1ccc(F)cc1 | -6.86 |
| O=S(=O)(Nc1ccccc1)Nc1ccccc1 | -6.84 |
| CCCCNS(=O)(=O)N[C@@H](CCC(=O)OC)C(=O)OC | -6.83 |
| CC(C)(OC(=O)c1ccc(O)cc1)C(=O)NCCc1ccccc1 | -6.82 |
| CCCC(CCC)C(=O)OC(C)(C)C(=O)N[C@@H](C)c1ccccc1 | -6.8 |
| CCCC(CCC)C(=O)N[C@@H](C)COc1c(C)cccc1C | -6.79 |
| O=C(/C=C/c1ccc(O)c(O)c1)NCC1CCCCC1 | -6.77 |
| O=C(NCc1ccccc1)c1ccc(F)cc1 | -6.76 |
| O=C(NCc1ccccc1)c1ccccc1 | -6.74 |
| COC(=O)CC[C@@H](NS(=O)(=O)N[C@H](C(=O)OC)C(C)C)C(=O)O<br>C | -6.66 |
| CCC1(c2ccccc2)C(=O)NC(=O)NC1=O | -6.6 |
| COc1cc2nc(N3CCN(C(=O)[C@@H]4COc5ccccc5O4)CC3)nc(N)c2cc1O<br>C | -6.57 |
| O=C(NC1CCCCC1)c1ccccc1 | -6.55 |
| C[C@@H](c1ccccc1)N1C(=O)C(C)(C)OS1(=O)=O | -6.54 |
| NC(=O)N1c2ccccc2CC(=O)c2ccccc21 | -6.52 |
| CC1(C)OS(=O)(=O)N(c2ccccc2)C1=O | -6.51 |
| CCCC(CCC)C(=O)N(c1ccccc1)c1ccccc1 | -6.48 |
| CCCC(CCC)C(=O)NCCc1ccccc1 | -6.44 |
| O=C(NC1CCCCC1)c1ccc(F)cc1 | -6.36 |
| Cc1cccc(C)c1NC(=O)/C=C/c1ccc(O)c(O)c1 | -6.35 |
| Cn1cc(NC(=O)C(C)(C)O)c(=O)n1-c1ccccc1 | -6.34 |
| CCCC(CCC)C(=O)NC1CCCCC1 | -6.32 |
| CCCCCCNC(=O)c1ccccc1 | -6.29 |
| CCCCCCNC(=O)c1ccccc1 | -6.27 |
| C[NH2+][C@H](Cc1c[nH]c2ccccc12)C(=O)[O-] | -6.27 |
| O=C([O-])c1ccccc1[C@H]1COc2ccccc2C1 | -6.26 |
| COC(=O)CC[C@H](NS(=O)(=O)N[C@@H](Cc1ccccc1)C(=O)OC)C(=<br>O)OC | -6.24 |
| O=C(NC1CCCCC1)c1ccccc1 | -6.19 |
| O=C(NC1CCCCC1)c1ccc(F)cc1 | -6.19 |
| COC(=O)CC[C@H](NS(=O)(=O)NCC(=O)OC)C(=O)OC | -6.18 |
| CCCCCCNC(=O)c1ccc(F)cc1 | -6.18 |
| Cc1ccc(C)c(OCCCC(C)(C)C(=O)[O-])c1 | -6.17 |
| CC1(C)CC2(C(N)=O)CCC1CC2 | -6.17 |
| CCCCCCNC(=O)c1ccc(F)cc1 | -6.13 |

| | |
|---|---|
| O=C(/C=C/c1ccc(O)c(O)c1)Oc1ccccc1 | -6.1 |
| CCC[C@H](C)C1(CC)C(=O)NC(=O)NC1=O | -6.01 |
| CC(C)Cc1ccc([C@@H](C)C(=O)[O-])cc1 | -6 |
| COC(=O)CC[C@@H](NS(=O)(=O)N[C@H](C)C(=O)OC)C(=O)OC | -5.98 |
| CCCCNc1ccc(C(=O)OCC[NH+](C)C)cc1 | -5.94 |
| Cc1cccc(C)c1OC[C@H](C)[NH3+] | -5.92 |
| CCCC(CCC)C(=O)Nc1ccccc1 | -5.89 |
| O=C(Nc1ccccc1)c1ccccc1 | -5.81 |
| O=C(Nc1ccccc1)c1ccc(F)cc1 | -5.8 |
| CC(C)(C)OC(=O)/C=C/c1ccc(O)c(O)c1 | -5.73 |
| CC(C)CNC(=O)c1ccccc1 | -5.71 |
| COC(=O)C1=C(C)NC(C)=C(C(=O)OC)C1c1ccccc1[N+](=O)[O-] | -5.69 |
| CCCCN1C(=O)C(C)(C)OS1(=O)=O | -5.66 |
| CCCCNC(=O)c1ccccc1 | -5.65 |
| CCCCNC(=O)c1ccccc1 | -5.64 |
| CC(C)CNC(=O)c1ccc(F)cc1 | -5.61 |
| COc1cc2c(c(OC)c1OC)-c1ccc(OC)c(=O)cc1[C@H](NC(C)=O)CC2 | -5.58 |
| CCC1(CC)C(=O)NC(=O)NC1=O | -5.55 |
| NC(=O)NCCC[C@H]([NH3+])C(=O)[O-] | -5.54 |
| CCCCNC(=O)c1ccc(F)cc1 | -5.51 |
| CCCCNC(=O)c1ccc(F)cc1 | -5.51 |
| Cc1occc1C(=O)N[C@H](C)C(C)(C)C | -5.49 |
| CC(C)NC(=O)c1ccccc1 | -5.45 |
| CC(C)(O)[C@H]1[C@@H]2C(=O)O[C@H]1[C@H]1OC(=O)[C@@]34<br>O[C@@H]3C[C@]2(O)[C@@]14C | -5.41 |
| CC(C)NC(=O)c1ccc(F)cc1 | -5.39 |
| C1C[NH2+]CC[NH2+]1 | -5.23 |
| CC(C)OC(=O)/C=C/c1ccc(O)c(O)c1 | -5.19 |
| CCCC(CCC)C(=O)NCC(C)C | -5.17 |
| CCCCOC(=O)/C=C/c1ccc(O)c(O)c1 | -5.14 |
| CC[C@H](C(=O)[O-])c1ccccc1 | -5.09 |
| O=C([O-])/C=C/c1ccc(O)cc1 | -5.06 |
| O=C([O-])CCCC[C@H]1CCSS1 | -5.01 |
| CC[C@@H](C(N)=O)N1CCCC1=O | -5 |
| CCCC(CCC)C(=O)NC(C)C | -4.96 |
| COC(=O)CC[C@H](N[S@](=O)Oc1ccccc1O)C(=O)OC | -4.93 |
| CCCCNC(=O)C(CCC)CCC | -4.9 |
| CC(C)=C[C@H]1[C@@H](C(=O)[O-])C1(C)C | -4.87 |
| CCCCN(CCCC)S(=O)(=O)N(CCCC)CCCC | -4.86 |
| O=C([O-])/C=C/c1ccc(O)c(O)c1 | -4.85 |
| CC(=O)NCCCS(=O)(=O)[O-] | -4.76 |
| COC(=O)/C=C/c1ccc(O)c(O)c1 | -4.76 |
| CCCOC(=O)/C=C/c1ccc(O)c(O)c1 | -4.76 |
| O=C([O-])CCCCCCCC(=O)[O-] | -4.75 |
| CO[C@@H]1[C@@H](OC(N)=O)[C@@H](O)[C@H](Oc2ccc3c(O)c(N<br>C(=O)c4ccc(O)c(CC=C(C)C)c4)c(=O)oc3c2C)OC1(C)C | -4.68 |
| CC[C@H](C)[C@H](NC(=O)OC(C)(C)C)C(=O)[O-] | -4.66 |

| | |
|---|---|
| COC(=O)c1ccc(O)cc1 | -4.66 |
| CCCNC(=O)C(CCC)CCC | -4.65 |
| O=C([O-])C1(O)CCCCCC1 | -4.57 |
| CCCOC(=O)c1ccc(O)cc1 | -4.26 |
| OC[C@@H](O)[C@@H](O)[C@H](O)[C@H](O)CO | -4.04 |
| OC[C@H](O)[C@@H](O)[C@H](O)[C@H](O)CO | -3.88 |
| CC[C@@H]([C@H](C)O)n1ncn(-c2ccc(N3CCN(c4ccc(OC[C@@H]5CO[C@@](Cn6cncn6)(c6ccc(F)cc6F)C5)cc4)CC3)cc2)c1=O | -2.96 |

Table S3

| | **Pearson's R** | | **Kendall's Tau B** | | **Spearman's Rho** | |
|---|---|---|---|---|---|---|
| Snap | Mean | STD | Mean | STD | Mean | STD |
| 10595_r | 0.492 | 0.061 | 0.300 | 0.053 | 0.476 | 0.072 |
| 12684_r | 0.441 | 0.057 | 0.295 | 0.050 | 0.469 | 0.071 |
| 1554_r | 0.588 | 0.067 | 0.350 | 0.063 | 0.516 | 0.081 |
| 15589_r | 0.214 | 0.058 | 0.244 | 0.056 | 0.401 | 0.081 |
| **17423_r** | **0.615** | **0.053** | **0.417** | **0.058** | **0.607** | **0.070** |
| 18985_r | 0.472 | 0.065 | 0.325 | 0.054 | 0.502 | 0.072 |
| 22514_r | 0.458 | 0.066 | 0.341 | 0.054 | 0.519 | 0.070 |
| 307_r | 0.583 | 0.062 | 0.384 | 0.056 | 0.567 | 0.071 |
| 3513_r | 0.365 | 0.066 | 0.243 | 0.056 | 0.386 | 0.080 |
| 5747_r | 0.255 | 0.058 | 0.214 | 0.047 | 0.375 | 0.074 |

Coordinates of the TcCA model

| | | | | | | | | | | |
|---|---|---|---|---|---|---|---|---|---|---|
| ATOM | 1 | N | GLN | 1 | 49.783 | 17.833 | 36.038 | 1.00 | 0.00 | N |
| ATOM | 2 | H1 | GLN | 1 | 50.699 | 17.943 | 36.450 | 1.00 | 0.00 | H |
| ATOM | 3 | H2 | GLN | 1 | 49.258 | 17.157 | 36.576 | 1.00 | 0.00 | H |
| ATOM | 4 | H3 | GLN | 1 | 49.880 | 17.506 | 35.087 | 1.00 | 0.00 | H |
| ATOM | 5 | CA | GLN | 1 | 49.092 | 19.093 | 36.052 | 1.00 | 0.00 | C |
| ATOM | 6 | HA | GLN | 1 | 49.676 | 19.837 | 35.509 | 1.00 | 0.00 | H |
| ATOM | 7 | CB | GLN | 1 | 47.694 | 18.999 | 35.435 | 1.00 | 0.00 | C |
| ATOM | 8 | HB2 | GLN | 1 | 47.787 | 18.753 | 34.376 | 1.00 | 0.00 | H |
| ATOM | 9 | HB3 | GLN | 1 | 47.158 | 18.179 | 35.916 | 1.00 | 0.00 | H |
| ATOM | 10 | CG | GLN | 1 | 46.856 | 20.255 | 35.598 | 1.00 | 0.00 | C |
| ATOM | 11 | HG2 | GLN | 1 | 45.858 | 20.047 | 35.210 | 1.00 | 0.00 | H |
| ATOM | 12 | HG3 | GLN | 1 | 46.750 | 20.512 | 36.652 | 1.00 | 0.00 | H |
| ATOM | 13 | CD | GLN | 1 | 47.422 | 21.433 | 34.829 | 1.00 | 0.00 | C |
| ATOM | 14 | OE1 | GLN | 1 | 47.697 | 22.523 | 35.537 | 1.00 | 0.00 | O |
| ATOM | 15 | NE2 | GLN | 1 | 47.611 | 21.365 | 33.612 | 1.00 | 0.00 | N |
| ATOM | 16 | HE21 | GLN | 1 | 47.353 | 20.539 | 33.095 | 1.00 | 0.00 | H |
| ATOM | 17 | HE22 | GLN | 1 | 47.970 | 22.178 | 33.132 | 1.00 | 0.00 | H |
| ATOM | 18 | C | GLN | 1 | 49.031 | 19.471 | 37.501 | 1.00 | 0.00 | C |
| ATOM | 19 | O | GLN | 1 | 48.649 | 18.721 | 38.402 | 1.00 | 0.00 | O |
| ATOM | 20 | N | TRP | 2 | 49.531 | 20.660 | 37.830 | 1.00 | 0.00 | N |
| ATOM | 21 | H | TRP | 2 | 49.876 | 21.295 | 37.123 | 1.00 | 0.00 | H |
| ATOM | 22 | CA | TRP | 2 | 49.423 | 21.151 | 39.219 | 1.00 | 0.00 | C |
| ATOM | 23 | HA | TRP | 2 | 49.552 | 20.322 | 39.911 | 1.00 | 0.00 | H |
| ATOM | 24 | CB | TRP | 2 | 50.545 | 22.155 | 39.488 | 1.00 | 0.00 | C |
| ATOM | 25 | HB2 | TRP | 2 | 50.584 | 22.883 | 38.676 | 1.00 | 0.00 | H |
| ATOM | 26 | HB3 | TRP | 2 | 50.321 | 22.697 | 40.406 | 1.00 | 0.00 | H |
| ATOM | 27 | CG | TRP | 2 | 51.891 | 21.517 | 39.658 | 1.00 | 0.00 | C |
| ATOM | 28 | CD1 | TRP | 2 | 52.147 | 20.275 | 40.156 | 1.00 | 0.00 | C |

ATOM 29 HD1 TRP 2 51.410 19.583 40.531 1.00 0.00 H

ATOM 30 NE1 TRP 2 53.499 20.036 40.160 1.00 0.00 N

ATOM 31 HE1 TRP 2 53.927 19.180 40.484 1.00 0.00 H

ATOM 32 CE2 TRP 2 54.146 21.135 39.654 1.00 0.00 C

ATOM 33 CZ2 TRP 2 55.499 21.366 39.462 1.00 0.00 C

ATOM 34 HZ2 TRP 2 56.237 20.627 39.735 1.00 0.00 H

ATOM 35 CH2 TRP 2 55.872 22.597 38.921 1.00 0.00 C

ATOM 36 HH2 TRP 2 56.918 22.802 38.746 1.00 0.00 H

ATOM 37 CZ3 TRP 2 54.939 23.548 38.593 1.00 0.00 C

ATOM 38 HZ3 TRP 2 55.263 24.488 38.170 1.00 0.00 H

ATOM 39 CE3 TRP 2 53.583 23.317 38.786 1.00 0.00 C

ATOM 40 HE3 TRP 2 52.857 24.079 38.544 1.00 0.00 H

ATOM 41 CD2 TRP 2 53.179 22.090 39.327 1.00 0.00 C

ATOM 42 C TRP 2 48.091 21.808 39.594 1.00 0.00 C

ATOM 43 O TRP 2 47.510 22.554 38.785 1.00 0.00 O

ATOM 44 N GLY 3 47.627 21.732 40.796 1.00 0.00 N

ATOM 45 H GLY 3 48.176 21.176 41.436 1.00 0.00 H

ATOM 46 CA GLY 3 46.456 22.309 41.396 1.00 0.00 C

ATOM 47 HA2 GLY 3 46.584 23.384 41.514 1.00 0.00 H

ATOM 48 HA3 GLY 3 45.612 22.076 40.756 1.00 0.00 H

ATOM 49 C GLY 3 46.200 21.645 42.806 1.00 0.00 C

ATOM 50 O GLY 3 46.960 20.825 43.316 1.00 0.00 O

ATOM 51 N TYR 4 45.075 22.045 43.429 1.00 0.00 N

ATOM 52 H TYR 4 44.488 22.737 42.989 1.00 0.00 H

ATOM 53 CA TYR 4 44.689 21.571 44.754 1.00 0.00 C

ATOM 54 HA TYR 4 45.579 21.538 45.383 1.00 0.00 H

ATOM 55 CB TYR 4 43.720 22.571 45.390 1.00 0.00 C

ATOM 56 HB2 TYR 4 42.942 22.829 44.669 1.00 0.00 H

ATOM 57 HB3 TYR 4 43.213 22.128 46.247 1.00 0.00 H

ATOM 58 CG TYR 4 44.389 23.829 45.899 1.00 0.00 C

ATOM 59 CD1 TYR 4 44.299 25.006 45.170 1.00 0.00 C

ATOM 60 HD1 TYR 4 43.765 25.019 44.236 1.00 0.00 H

ATOM 61 CE1 TYR 4 44.910 26.155 45.635 1.00 0.00 C

ATOM 62 HE1 TYR 4 44.848 27.072 45.069 1.00 0.00 H

ATOM 63 CZ TYR 4 45.612 26.125 46.834 1.00 0.00 C

ATOM 64 OH TYR 4 46.220 27.269 47.298 1.00 0.00 O

ATOM 65 HH TYR 4 46.621 27.163 48.170 1.00 0.00 H

ATOM 66 CE2 TYR 4 45.701 24.954 47.560 1.00 0.00 C

ATOM 67 HE2 TYR 4 46.244 24.942 48.492 1.00 0.00 H

ATOM 68 CD2 TYR 4 45.091 23.806 47.095 1.00 0.00 C

ATOM 69 HD2 TYR 4 45.170 22.894 47.667 1.00 0.00 H

ATOM 70 C TYR 4 44.068 20.185 44.786 1.00 0.00 C

ATOM 71 O TYR 4 43.948 19.512 43.767 1.00 0.00 O

ATOM 72 N THR 5 43.786 19.685 46.036 1.00 0.00 N

ATOM 73 H THR 5 43.944 20.292 46.828 1.00 0.00 H

ATOM 74 CA THR 5 43.350 18.370 46.342 1.00 0.00 C

ATOM 75 HA THR 5 43.095 18.353 47.402 1.00 0.00 H

ATOM 76 CB THR 5 42.070 18.057 45.545 1.00 0.00 C

ATOM 77 HB THR 5 42.232 18.122 44.472 1.00 0.00 H

ATOM 78 CG2 THR 5 41.596 16.641 45.832 1.00 0.00 C

ATOM 79 HG21 THR 5 40.592 16.509 45.425 1.00 0.00 H

ATOM 80 HG22 THR 5 42.244 15.907 45.353 1.00 0.00 H

ATOM 81 HG23 THR 5 41.566 16.460 46.907 1.00 0.00 H

ATOM 82 OG1 THR 5 41.039 18.985 45.908 1.00 0.00 O

ATOM 83 HG1 THR 5 40.839 18.861 46.840 1.00 0.00 H

ATOM 84 C THR 5 44.325 17.272 46.110 1.00 0.00 C

ATOM 85 O THR 5 44.674 16.477 47.017 1.00 0.00 O

ATOM 86 N ASN 6 44.965 17.202 44.972 1.00 0.00 N

ATOM 87 H ASN 6 44.705 17.861 44.253 1.00 0.00 H

ATOM 88 CA ASN 6 46.079 16.325 44.647 1.00 0.00 C

```
ATOM     89  HA  ASN     6      46.011  15.423  45.257  1.00  0.00           H
ATOM     90  CB  ASN     6      46.008  15.914  43.188  1.00  0.00           C
ATOM     91  HB2 ASN     6      46.022  16.802  42.552  1.00  0.00           H
ATOM     92  HB3 ASN     6      46.873  15.294  42.946  1.00  0.00           H
ATOM     93  CG  ASN     6      44.787  15.094  42.877  1.00  0.00           C
ATOM     94  OD1 ASN     6      44.187  15.348  41.742  1.00  0.00           O
ATOM     95  ND2 ASN     6      44.387  14.230  43.666  1.00  0.00           N
ATOM     96 HD21 ASN     6      43.523  13.750  43.464  1.00  0.00           H
ATOM     97 HD22 ASN     6      44.919  13.997  44.489  1.00  0.00           H
ATOM     98  C   ASN     6      47.430  16.927  44.943  1.00  0.00           C
ATOM     99  O   ASN     6      48.213  17.123  44.035  1.00  0.00           O
ATOM    100  N   LEU     7      47.712  17.314  46.190  1.00  0.00           N
ATOM    101  H   LEU     7      47.036  17.156  46.925  1.00  0.00           H
ATOM    102  CA  LEU     7      48.945  18.093  46.521  1.00  0.00           C
ATOM    103  HA  LEU     7      49.001  18.955  45.855  1.00  0.00           H
ATOM    104  CB  LEU     7      48.874  18.598  47.967  1.00  0.00           C
ATOM    105  HB2 LEU     7      48.368  17.850  48.581  1.00  0.00           H
ATOM    106  HB3 LEU     7      49.887  18.694  48.361  1.00  0.00           H
ATOM    107  CG  LEU     7      48.208  19.966  48.162  1.00  0.00           C
ATOM    108  HG  LEU     7      48.807  20.725  47.664  1.00  0.00           H
ATOM    109  CD1 LEU     7      46.817  19.947  47.543  1.00  0.00           C
ATOM    110 HD11 LEU     7      46.306  20.877  47.786  1.00  0.00           H
ATOM    111 HD12 LEU     7      46.884  19.876  46.458  1.00  0.00           H
ATOM    112 HD13 LEU     7      46.235  19.114  47.939  1.00  0.00           H
ATOM    113  CD2 LEU     7      48.142  20.292  49.647  1.00  0.00           C
ATOM    114 HD21 LEU     7      47.699  21.279  49.786  1.00  0.00           H
ATOM    115 HD22 LEU     7      47.540  19.552  50.175  1.00  0.00           H
ATOM    116 HD23 LEU     7      49.149  20.301  50.064  1.00  0.00           H
ATOM    117  C   LEU     7      50.239  17.337  46.357  1.00  0.00           C
ATOM    118  O   LEU     7      51.377  17.756  46.376  1.00  0.00           O
```

```
ATOM    119  N   THR     8      50.173  15.981  46.318  1.00  0.00           N
ATOM    120  H   THR     8      49.276  15.518  46.351  1.00  0.00           H
ATOM    121  CA  THR     8      51.402  15.121  46.126  1.00  0.00           C
ATOM    122  HA  THR     8      52.128  15.387  46.896  1.00  0.00           H
ATOM    123  CB  THR     8      51.043  13.635  46.310  1.00  0.00           C
ATOM    124  HB  THR     8      51.939  13.031  46.167  1.00  0.00           H
ATOM    125  CG2 THR     8      50.497  13.387  47.708  1.00  0.00           C
ATOM    126 HG21 THR     8      50.362  12.316  47.859  1.00  0.00           H
ATOM    127 HG22 THR     8      51.202  13.759  48.453  1.00  0.00           H
ATOM    128 HG23 THR     8      49.535  13.883  47.843  1.00  0.00           H
ATOM    129  OG1 THR     8      50.056  13.255  45.342  1.00  0.00           O
ATOM    130  HG1 THR     8      50.189  12.329  45.123  1.00  0.00           H
ATOM    131  C   THR     8      52.112  15.312  44.717  1.00  0.00           C
ATOM    132  O   THR     8      53.220  14.838  44.612  1.00  0.00           O
ATOM    133  N   ALA     9      51.457  15.975  43.768  1.00  0.00           N
ATOM    134  H   ALA     9      50.524  16.323  43.936  1.00  0.00           H
ATOM    135  CA  ALA     9      52.088  16.252  42.515  1.00  0.00           C
ATOM    136  HA  ALA     9      52.722  15.413  42.225  1.00  0.00           H
ATOM    137  CB  ALA     9      50.929  16.362  41.488  1.00  0.00           C
ATOM    138  HB1 ALA     9      51.340  16.546  40.495  1.00  0.00           H
ATOM    139  HB2 ALA     9      50.359  15.432  41.468  1.00  0.00           H
ATOM    140  HB3 ALA     9      50.267  17.187  41.758  1.00  0.00           H
ATOM    141  C   ALA     9      52.973  17.522  42.638  1.00  0.00           C
ATOM    142  O   ALA     9      53.651  17.771  41.637  1.00  0.00           O
ATOM    143  N   TRP    10      52.945  18.231  43.759  1.00  0.00           N
ATOM    144  H   TRP    10      52.379  17.912  44.532  1.00  0.00           H
ATOM    145  CA  TRP    10      53.644  19.508  43.923  1.00  0.00           C
ATOM    146  HA  TRP    10      53.509  20.083  43.009  1.00  0.00           H
ATOM    147  CB  TRP    10      53.010  20.292  45.074  1.00  0.00           C
ATOM    148  HB2 TRP    10      53.038  19.697  45.986  1.00  0.00           H
```

ATOM 149 HB3 TRP 10 53.601 21.191 45.253 1.00 0.00 H

ATOM 150 CG TRP 10 51.602 20.726 44.798 1.00 0.00 C

ATOM 151 CD1 TRP 10 50.807 20.309 43.773 1.00 0.00 C

ATOM 152 HD1 TRP 10 51.080 19.624 42.988 1.00 0.00 H

ATOM 153 NE1 TRP 10 49.582 20.925 43.844 1.00 0.00 N

ATOM 154 HE1 TRP 10 48.817 20.742 43.213 1.00 0.00 H

ATOM 155 CE2 TRP 10 49.566 21.759 44.933 1.00 0.00 C

ATOM 156 CZ2 TRP 10 48.561 22.585 45.412 1.00 0.00 C

ATOM 157 HZ2 TRP 10 47.609 22.676 44.918 1.00 0.00 H

ATOM 158 CH2 TRP 10 48.833 23.332 46.558 1.00 0.00 C

ATOM 159 HH2 TRP 10 48.076 23.988 46.954 1.00 0.00 H

ATOM 160 CZ3 TRP 10 50.048 23.258 47.191 1.00 0.00 C

ATOM 161 HZ3 TRP 10 50.206 23.851 48.071 1.00 0.00 H

ATOM 162 CE3 TRP 10 51.056 22.431 46.710 1.00 0.00 C

ATOM 163 HE3 TRP 10 52.014 22.384 47.208 1.00 0.00 H

ATOM 164 CD2 TRP 10 50.811 21.669 45.561 1.00 0.00 C

ATOM 165 C TRP 10 55.180 19.327 44.187 1.00 0.00 C

ATOM 166 O TRP 10 55.529 18.263 44.699 1.00 0.00 O

ATOM 167 N PRO 11 56.015 20.309 43.829 1.00 0.00 N

ATOM 168 CD PRO 11 55.669 21.565 43.138 1.00 0.00 C

ATOM 169 HD2 PRO 11 55.264 22.282 43.853 1.00 0.00 H

ATOM 170 HD3 PRO 11 54.987 21.411 42.304 1.00 0.00 H

ATOM 171 CG PRO 11 57.011 22.031 42.640 1.00 0.00 C

ATOM 172 HG2 PRO 11 57.027 23.108 42.493 1.00 0.00 H

ATOM 173 HG3 PRO 11 57.252 21.526 41.705 1.00 0.00 H

ATOM 174 CB PRO 11 57.971 21.589 43.691 1.00 0.00 C

ATOM 175 HB2 PRO 11 57.925 22.284 44.532 1.00 0.00 H

ATOM 176 HB3 PRO 11 58.990 21.534 43.306 1.00 0.00 H

ATOM 177 CA PRO 11 57.456 20.209 44.114 1.00 0.00 C

ATOM 178 HA PRO 11 57.896 19.438 43.480 1.00 0.00 H

| | | | | | | | | | | |
|---|---|---|---|---|---|---|---|---|---|---|
| ATOM | 179 | C | PRO | 11 | 57.767 | 19.928 | 45.596 | 1.00 | 0.00 | C |
| ATOM | 180 | O | PRO | 11 | 57.009 | 20.400 | 46.449 | 1.00 | 0.00 | O |
| ATOM | 181 | N | LYS | 12 | 58.788 | 19.020 | 45.934 | 1.00 | 0.00 | N |
| ATOM | 182 | H | LYS | 12 | 59.321 | 18.631 | 45.170 | 1.00 | 0.00 | H |
| ATOM | 183 | CA | LYS | 12 | 59.241 | 18.641 | 47.279 | 1.00 | 0.00 | C |
| ATOM | 184 | HA | LYS | 12 | 58.982 | 19.456 | 47.957 | 1.00 | 0.00 | H |
| ATOM | 185 | CB | LYS | 12 | 58.497 | 17.389 | 47.747 | 1.00 | 0.00 | C |
| ATOM | 186 | HB2 | LYS | 12 | 58.772 | 17.183 | 48.783 | 1.00 | 0.00 | H |
| ATOM | 187 | HB3 | LYS | 12 | 57.426 | 17.601 | 47.727 | 1.00 | 0.00 | H |
| ATOM | 188 | CG | LYS | 12 | 58.765 | 16.147 | 46.908 | 1.00 | 0.00 | C |
| ATOM | 189 | HG2 | LYS | 12 | 58.513 | 16.343 | 45.865 | 1.00 | 0.00 | H |
| ATOM | 190 | HG3 | LYS | 12 | 59.821 | 15.882 | 46.972 | 1.00 | 0.00 | H |
| ATOM | 191 | CD | LYS | 12 | 57.941 | 14.964 | 47.393 | 1.00 | 0.00 | C |
| ATOM | 192 | HD2 | LYS | 12 | 58.163 | 14.779 | 48.445 | 1.00 | 0.00 | H |
| ATOM | 193 | HD3 | LYS | 12 | 56.880 | 15.201 | 47.291 | 1.00 | 0.00 | H |
| ATOM | 194 | CE | LYS | 12 | 58.251 | 13.708 | 46.593 | 1.00 | 0.00 | C |
| ATOM | 195 | HE2 | LYS | 12 | 58.021 | 13.915 | 45.546 | 1.00 | 0.00 | H |
| ATOM | 196 | HE3 | LYS | 12 | 59.312 | 13.467 | 46.674 | 1.00 | 0.00 | H |
| ATOM | 197 | NZ | LYS | 12 | 57.434 | 12.547 | 47.040 | 1.00 | 0.00 | N |
| ATOM | 198 | HZ1 | LYS | 12 | 57.375 | 11.865 | 46.298 | 1.00 | 0.00 | H |
| ATOM | 199 | HZ2 | LYS | 12 | 57.858 | 12.126 | 47.853 | 1.00 | 0.00 | H |
| ATOM | 200 | HZ3 | LYS | 12 | 56.504 | 12.861 | 47.274 | 1.00 | 0.00 | H |
| ATOM | 201 | C | LYS | 12 | 60.721 | 18.397 | 47.398 | 1.00 | 0.00 | C |
| ATOM | 202 | O | LYS | 12 | 61.318 | 18.087 | 46.352 | 1.00 | 0.00 | O |
| ATOM | 203 | N | LEU | 13 | 61.299 | 18.649 | 48.603 | 1.00 | 0.00 | N |
| ATOM | 204 | H | LEU | 13 | 60.718 | 18.975 | 49.362 | 1.00 | 0.00 | H |
| ATOM | 205 | CA | LEU | 13 | 62.724 | 18.399 | 48.908 | 1.00 | 0.00 | C |
| ATOM | 206 | HA | LEU | 13 | 63.002 | 17.458 | 48.433 | 1.00 | 0.00 | H |
| ATOM | 207 | CB | LEU | 13 | 63.578 | 19.521 | 48.304 | 1.00 | 0.00 | C |
| ATOM | 208 | HB2 | LEU | 13 | 63.440 | 19.521 | 47.222 | 1.00 | 0.00 | H |

ATOM 209 HB3 LEU 13 63.192 20.471 48.669 1.00 0.00 H

ATOM 210 CG LEU 13 65.081 19.447 48.601 1.00 0.00 C

ATOM 211 HG LEU 13 65.247 19.433 49.671 1.00 0.00 H

ATOM 212 CD1 LEU 13 65.659 18.181 47.984 1.00 0.00 C

ATOM 213 HD11 LEU 13 66.739 18.165 48.135 1.00 0.00 H

ATOM 214 HD12 LEU 13 65.244 17.294 48.461 1.00 0.00 H

ATOM 215 HD13 LEU 13 65.452 18.149 46.913 1.00 0.00 H

ATOM 216 CD2 LEU 13 65.769 20.687 48.051 1.00 0.00 C

ATOM 217 HD21 LEU 13 66.838 20.636 48.250 1.00 0.00 H

ATOM 218 HD22 LEU 13 65.616 20.753 46.973 1.00 0.00 H

ATOM 219 HD23 LEU 13 65.369 21.585 48.520 1.00 0.00 H

ATOM 220 C LEU 13 63.019 18.288 50.438 1.00 0.00 C

ATOM 221 O LEU 13 62.753 19.255 51.160 1.00 0.00 O

ATOM 222 N CYS 14 63.668 17.230 50.850 1.00 0.00 N

ATOM 223 H CYS 14 63.927 16.525 50.175 1.00 0.00 H

ATOM 224 CA CYS 14 63.957 16.936 52.270 1.00 0.00 C

ATOM 225 HA CYS 14 63.010 16.856 52.805 1.00 0.00 H

ATOM 226 CB CYS 14 64.719 15.619 52.421 1.00 0.00 C

ATOM 227 HB2 CYS 14 64.814 15.380 53.481 1.00 0.00 H

ATOM 228 HB3 CYS 14 64.138 14.826 51.950 1.00 0.00 H

ATOM 229 SG CYS 14 66.371 15.634 51.686 1.00 0.00 S

ATOM 230 HG CYS 14 66.527 14.307 51.635 1.00 0.00 H

ATOM 231 C CYS 14 64.759 18.009 52.906 1.00 0.00 C

ATOM 232 O CYS 14 64.927 18.017 54.148 1.00 0.00 O

ATOM 233 N GLN 15 65.539 18.819 52.159 1.00 0.00 N

ATOM 234 H GLN 15 65.509 18.705 51.158 1.00 0.00 H

ATOM 235 CA GLN 15 66.403 19.911 52.672 1.00 0.00 C

ATOM 236 HA GLN 15 66.855 19.587 53.610 1.00 0.00 H

ATOM 237 CB GLN 15 67.529 20.194 51.674 1.00 0.00 C

ATOM 238 HB2 GLN 15 67.066 20.472 50.728 1.00 0.00 H

| | | | | | | | | | | |
|---|---|---|---|---|---|---|---|---|---|---|
| ATOM | 239 | HB3 | GLN | 15 | 68.118 | 21.042 | 52.026 | 1.00 | 0.00 | H |
| ATOM | 240 | CG | GLN | 15 | 68.488 | 19.033 | 51.472 | 1.00 | 0.00 | C |
| ATOM | 241 | HG2 | GLN | 15 | 68.990 | 18.813 | 52.415 | 1.00 | 0.00 | H |
| ATOM | 242 | HG3 | GLN | 15 | 67.928 | 18.154 | 51.157 | 1.00 | 0.00 | H |
| ATOM | 243 | CD | GLN | 15 | 69.526 | 19.319 | 50.404 | 1.00 | 0.00 | C |
| ATOM | 244 | OE1 | GLN | 15 | 70.642 | 18.601 | 50.457 | 1.00 | 0.00 | O |
| ATOM | 245 | NE2 | GLN | 15 | 69.328 | 20.177 | 49.539 | 1.00 | 0.00 | N |
| ATOM | 246 | HE21 | GLN | 15 | 68.488 | 20.734 | 49.554 | 1.00 | 0.00 | H |
| ATOM | 247 | HE22 | GLN | 15 | 70.029 | 20.322 | 48.828 | 1.00 | 0.00 | H |
| ATOM | 248 | C | GLN | 15 | 65.605 | 21.229 | 52.962 | 1.00 | 0.00 | C |
| ATOM | 249 | O | GLN | 15 | 66.271 | 22.240 | 53.239 | 1.00 | 0.00 | O |
| ATOM | 250 | N | THR | 16 | 64.312 | 21.241 | 52.760 | 1.00 | 0.00 | N |
| ATOM | 251 | H | THR | 16 | 63.825 | 20.406 | 52.468 | 1.00 | 0.00 | H |
| ATOM | 252 | CA | THR | 16 | 63.555 | 22.469 | 52.961 | 1.00 | 0.00 | C |
| ATOM | 253 | HA | THR | 16 | 64.226 | 23.328 | 52.941 | 1.00 | 0.00 | H |
| ATOM | 254 | CB | THR | 16 | 62.581 | 22.669 | 51.784 | 1.00 | 0.00 | C |
| ATOM | 255 | HB | THR | 16 | 62.021 | 23.594 | 51.926 | 1.00 | 0.00 | H |
| ATOM | 256 | CG2 | THR | 16 | 63.343 | 22.760 | 50.471 | 1.00 | 0.00 | C |
| ATOM | 257 | HG21 | THR | 16 | 62.650 | 22.960 | 49.654 | 1.00 | 0.00 | H |
| ATOM | 258 | HG22 | THR | 16 | 64.069 | 23.570 | 50.524 | 1.00 | 0.00 | H |
| ATOM | 259 | HG23 | THR | 16 | 63.876 | 21.834 | 50.275 | 1.00 | 0.00 | H |
| ATOM | 260 | OG1 | THR | 16 | 61.665 | 21.568 | 51.726 | 1.00 | 0.00 | O |
| ATOM | 261 | HG1 | THR | 16 | 62.156 | 20.774 | 51.488 | 1.00 | 0.00 | H |
| ATOM | 262 | C | THR | 16 | 62.764 | 22.585 | 54.258 | 1.00 | 0.00 | C |
| ATOM | 263 | O | THR | 16 | 62.101 | 23.564 | 54.525 | 1.00 | 0.00 | O |
| ATOM | 264 | N | GLY | 17 | 62.974 | 21.736 | 55.294 | 1.00 | 0.00 | N |
| ATOM | 265 | H | GLY | 17 | 63.617 | 20.971 | 55.149 | 1.00 | 0.00 | H |
| ATOM | 266 | CA | GLY | 17 | 62.228 | 21.727 | 56.548 | 1.00 | 0.00 | C |
| ATOM | 267 | HA2 | GLY | 17 | 61.250 | 21.274 | 56.387 | 1.00 | 0.00 | H |
| ATOM | 268 | HA3 | GLY | 17 | 62.775 | 21.135 | 57.282 | 1.00 | 0.00 | H |

| | | | | | | | | | | |
|---|---|---|---|---|---|---|---|---|---|---|
| ATOM | 269 | C | GLY | 17 | 62.049 | 23.166 | 57.097 | 1.00 | 0.00 | C |
| ATOM | 270 | O | GLY | 17 | 60.959 | 23.719 | 57.191 | 1.00 | 0.00 | O |
| ATOM | 271 | N | LYS | 18 | 63.102 | 23.982 | 57.252 | 1.00 | 0.00 | N |
| ATOM | 272 | H | LYS | 18 | 64.000 | 23.577 | 57.031 | 1.00 | 0.00 | H |
| ATOM | 273 | CA | LYS | 18 | 63.173 | 25.320 | 57.776 | 1.00 | 0.00 | C |
| ATOM | 274 | HA | LYS | 18 | 62.762 | 25.300 | 58.787 | 1.00 | 0.00 | H |
| ATOM | 275 | CB | LYS | 18 | 64.645 | 25.726 | 57.875 | 1.00 | 0.00 | C |
| ATOM | 276 | HB2 | LYS | 18 | 65.122 | 25.580 | 56.904 | 1.00 | 0.00 | H |
| ATOM | 277 | HB3 | LYS | 18 | 64.696 | 26.791 | 58.112 | 1.00 | 0.00 | H |
| ATOM | 278 | CG | LYS | 18 | 65.432 | 24.982 | 58.945 | 1.00 | 0.00 | C |
| ATOM | 279 | HG2 | LYS | 18 | 64.973 | 25.173 | 59.916 | 1.00 | 0.00 | H |
| ATOM | 280 | HG3 | LYS | 18 | 65.405 | 23.910 | 58.745 | 1.00 | 0.00 | H |
| ATOM | 281 | CD | LYS | 18 | 66.881 | 25.443 | 58.985 | 1.00 | 0.00 | C |
| ATOM | 282 | HD2 | LYS | 18 | 67.346 | 25.245 | 58.018 | 1.00 | 0.00 | H |
| ATOM | 283 | HD3 | LYS | 18 | 66.913 | 26.517 | 59.178 | 1.00 | 0.00 | H |
| ATOM | 284 | CE | LYS | 18 | 67.663 | 24.720 | 60.072 | 1.00 | 0.00 | C |
| ATOM | 285 | HE2 | LYS | 18 | 67.203 | 24.933 | 61.039 | 1.00 | 0.00 | H |
| ATOM | 286 | HE3 | LYS | 18 | 67.608 | 23.646 | 59.890 | 1.00 | 0.00 | H |
| ATOM | 287 | NZ | LYS | 18 | 69.091 | 25.139 | 60.103 | 1.00 | 0.00 | N |
| ATOM | 288 | HZ1 | LYS | 18 | 69.148 | 26.133 | 60.275 | 1.00 | 0.00 | H |
| ATOM | 289 | HZ2 | LYS | 18 | 69.581 | 24.644 | 60.836 | 1.00 | 0.00 | H |
| ATOM | 290 | HZ3 | LYS | 18 | 69.525 | 24.937 | 59.213 | 1.00 | 0.00 | H |
| ATOM | 291 | C | LYS | 18 | 62.427 | 26.399 | 57.038 | 1.00 | 0.00 | C |
| ATOM | 292 | O | LYS | 18 | 62.096 | 27.475 | 57.567 | 1.00 | 0.00 | O |
| ATOM | 293 | N | GLN | 19 | 62.142 | 26.087 | 55.795 | 1.00 | 0.00 | N |
| ATOM | 294 | H | GLN | 19 | 62.445 | 25.178 | 55.477 | 1.00 | 0.00 | H |
| ATOM | 295 | CA | GLN | 19 | 61.471 | 26.901 | 54.776 | 1.00 | 0.00 | C |
| ATOM | 296 | HA | GLN | 19 | 61.377 | 27.919 | 55.157 | 1.00 | 0.00 | H |
| ATOM | 297 | CB | GLN | 19 | 62.347 | 26.943 | 53.521 | 1.00 | 0.00 | C |
| ATOM | 298 | HB2 | GLN | 19 | 62.498 | 25.921 | 53.170 | 1.00 | 0.00 | H |

```
ATOM    299  HB3 GLN    19      61.833  27.484  52.727  1.00  0.00           H
ATOM    300  CG  GLN    19      63.709  27.582  53.734  1.00  0.00           C
ATOM    301  HG2 GLN    19      64.240  27.089  54.549  1.00  0.00           H
ATOM    302  HG3 GLN    19      64.299  27.442  52.829  1.00  0.00           H
ATOM    303  CD  GLN    19      63.613  29.072  54.001  1.00  0.00           C
ATOM    304  OE1 GLN    19      64.141  29.503  55.141  1.00  0.00           O
ATOM    305  NE2 GLN    19      63.071  29.829  53.191  1.00  0.00           N
ATOM    306 HE21 GLN    19      62.746  29.463  52.309  1.00  0.00           H
ATOM    307 HE22 GLN    19      63.013  30.813  53.403  1.00  0.00           H
ATOM    308  C   GLN    19      60.028  26.431  54.390  1.00  0.00           C
ATOM    309  O   GLN    19      59.466  27.031  53.461  1.00  0.00           O
ATOM    310  N   GLN    20      59.461  25.438  55.072  1.00  0.00           N
ATOM    311  H   GLN    20      59.963  24.971  55.814  1.00  0.00           H
ATOM    312  CA  GLN    20      58.146  24.881  54.612  1.00  0.00           C
ATOM    313  HA  GLN    20      58.249  24.548  53.578  1.00  0.00           H
ATOM    314  CB  GLN    20      57.709  23.689  55.468  1.00  0.00           C
ATOM    315  HB2 GLN    20      57.782  23.957  56.524  1.00  0.00           H
ATOM    316  HB3 GLN    20      56.666  23.485  55.240  1.00  0.00           H
ATOM    317  CG  GLN    20      58.491  22.414  55.205  1.00  0.00           C
ATOM    318  HG2 GLN    20      58.450  22.191  54.138  1.00  0.00           H
ATOM    319  HG3 GLN    20      59.533  22.563  55.472  1.00  0.00           H
ATOM    320  CD  GLN    20      57.941  21.228  55.975  1.00  0.00           C
ATOM    321  OE1 GLN    20      57.485  20.213  55.249  1.00  0.00           O
ATOM    322  NE2 GLN    20      57.925  21.224  57.209  1.00  0.00           N
ATOM    323 HE21 GLN    20      58.328  21.995  57.725  1.00  0.00           H
ATOM    324 HE22 GLN    20      57.592  20.409  57.699  1.00  0.00           H
ATOM    325  C   GLN    20      57.078  25.941  54.657  1.00  0.00           C
ATOM    326  O   GLN    20      57.198  26.807  55.534  1.00  0.00           O
ATOM    327  N   SER    21      56.038  25.902  53.804  1.00  0.00           N
ATOM    328  H   SER    21      55.951  25.193  53.091  1.00  0.00           H
```

ATOM 329 CA SER 21 55.013 26.999 53.899 1.00 0.00 C

ATOM 330 HA SER 21 54.951 27.350 54.926 1.00 0.00 H

ATOM 331 CB SER 21 55.534 28.122 53.051 1.00 0.00 C

ATOM 332 HB2 SER 21 56.545 28.382 53.368 1.00 0.00 H

ATOM 333 HB3 SER 21 55.574 27.749 52.029 1.00 0.00 H

ATOM 334 OG SER 21 54.780 29.280 53.033 1.00 0.00 O

ATOM 335 HG SER 21 55.104 29.816 52.299 1.00 0.00 H

ATOM 336 C SER 21 53.588 26.464 53.480 1.00 0.00 C

ATOM 337 O SER 21 53.592 25.522 52.591 1.00 0.00 O

ATOM 338 N PRO 22 52.447 26.925 54.001 1.00 0.00 N

ATOM 339 CD PRO 22 51.151 26.416 53.567 1.00 0.00 C

ATOM 340 HD2 PRO 22 51.115 25.328 53.611 1.00 0.00 H

ATOM 341 HD3 PRO 22 50.919 26.775 52.565 1.00 0.00 H

ATOM 342 CG PRO 22 50.206 27.013 54.575 1.00 0.00 C

ATOM 343 HG2 PRO 22 50.150 26.374 55.452 1.00 0.00 H

ATOM 344 HG3 PRO 22 49.218 27.148 54.155 1.00 0.00 H

ATOM 345 CB PRO 22 50.828 28.317 54.939 1.00 0.00 C

ATOM 346 HB2 PRO 22 50.454 28.697 55.889 1.00 0.00 H

ATOM 347 HB3 PRO 22 50.618 29.044 54.155 1.00 0.00 H

ATOM 348 CA PRO 22 52.330 28.019 54.979 1.00 0.00 C

ATOM 349 HA PRO 22 52.870 28.896 54.633 1.00 0.00 H

ATOM 350 C PRO 22 52.803 27.627 56.424 1.00 0.00 C

ATOM 351 O PRO 22 52.990 26.469 56.810 1.00 0.00 O

ATOM 352 N VAL 23 53.140 28.730 57.121 1.00 0.00 N

ATOM 353 H VAL 23 53.013 29.652 56.728 1.00 0.00 H

ATOM 354 CA VAL 23 53.531 28.600 58.495 1.00 0.00 C

ATOM 355 HA VAL 23 53.619 27.549 58.772 1.00 0.00 H

ATOM 356 CB VAL 23 54.920 29.236 58.690 1.00 0.00 C

ATOM 357 HB VAL 23 54.866 30.293 58.463 1.00 0.00 H

ATOM 358 CG1 VAL 23 55.364 29.111 60.140 1.00 0.00 C

| | | | | | | | | | | |
|---|---|---|---|---|---|---|---|---|---|---|
| ATOM | 359 | HG11 | VAL | 23 | 56.358 | 29.538 | 60.255 | 1.00 | 0.00 | H |
| ATOM | 360 | HG12 | VAL | 23 | 54.710 | 29.687 | 60.792 | 1.00 | 0.00 | H |
| ATOM | 361 | HG13 | VAL | 23 | 55.369 | 28.067 | 60.456 | 1.00 | 0.00 | H |
| ATOM | 362 | CG2 | VAL | 23 | 55.926 | 28.579 | 57.759 | 1.00 | 0.00 | C |
| ATOM | 363 | HG21 | VAL | 23 | 56.911 | 29.009 | 57.917 | 1.00 | 0.00 | H |
| ATOM | 364 | HG22 | VAL | 23 | 55.945 | 27.503 | 57.921 | 1.00 | 0.00 | H |
| ATOM | 365 | HG23 | VAL | 23 | 55.664 | 28.763 | 56.719 | 1.00 | 0.00 | H |
| ATOM | 366 | C | VAL | 23 | 52.538 | 29.240 | 59.468 | 1.00 | 0.00 | C |
| ATOM | 367 | O | VAL | 23 | 51.986 | 30.310 | 59.192 | 1.00 | 0.00 | O |
| ATOM | 368 | N | SER | 24 | 52.162 | 28.558 | 60.559 | 1.00 | 0.00 | N |
| ATOM | 369 | H | SER | 24 | 52.590 | 27.652 | 60.676 | 1.00 | 0.00 | H |
| ATOM | 370 | CA | SER | 24 | 51.347 | 28.981 | 61.698 | 1.00 | 0.00 | C |
| ATOM | 371 | HA | SER | 24 | 50.463 | 29.480 | 61.298 | 1.00 | 0.00 | H |
| ATOM | 372 | CB | SER | 24 | 50.879 | 27.738 | 62.430 | 1.00 | 0.00 | C |
| ATOM | 373 | HB2 | SER | 24 | 50.224 | 27.157 | 61.779 | 1.00 | 0.00 | H |
| ATOM | 374 | HB3 | SER | 24 | 51.742 | 27.128 | 62.701 | 1.00 | 0.00 | H |
| ATOM | 375 | OG | SER | 24 | 50.184 | 28.076 | 63.599 | 1.00 | 0.00 | O |
| ATOM | 376 | HG | SER | 24 | 50.710 | 27.800 | 64.358 | 1.00 | 0.00 | H |
| ATOM | 377 | C | SER | 24 | 52.032 | 29.947 | 62.717 | 1.00 | 0.00 | C |
| ATOM | 378 | O | SER | 24 | 53.250 | 29.789 | 63.013 | 1.00 | 0.00 | O |
| ATOM | 379 | N | PHE | 25 | 51.206 | 30.885 | 63.168 | 1.00 | 0.00 | N |
| ATOM | 380 | H | PHE | 25 | 50.255 | 30.910 | 62.828 | 1.00 | 0.00 | H |
| ATOM | 381 | CA | PHE | 25 | 51.591 | 31.887 | 64.224 | 1.00 | 0.00 | C |
| ATOM | 382 | HA | PHE | 25 | 52.662 | 31.831 | 64.420 | 1.00 | 0.00 | H |
| ATOM | 383 | CB | PHE | 25 | 51.217 | 33.320 | 63.840 | 1.00 | 0.00 | C |
| ATOM | 384 | HB2 | PHE | 25 | 50.161 | 33.366 | 63.580 | 1.00 | 0.00 | H |
| ATOM | 385 | HB3 | PHE | 25 | 51.363 | 33.988 | 64.683 | 1.00 | 0.00 | H |
| ATOM | 386 | CG | PHE | 25 | 52.035 | 33.877 | 62.709 | 1.00 | 0.00 | C |
| ATOM | 387 | CD1 | PHE | 25 | 51.561 | 33.838 | 61.406 | 1.00 | 0.00 | C |
| ATOM | 388 | HD1 | PHE | 25 | 50.596 | 33.401 | 61.196 | 1.00 | 0.00 | H |

ATOM 389 CE1 PHE 25 52.313 34.350 60.366 1.00 0.00 C

ATOM 390 HE1 PHE 25 51.944 34.297 59.353 1.00 0.00 H

ATOM 391 CZ PHE 25 53.548 34.909 60.617 1.00 0.00 C

ATOM 392 HZ PHE 25 54.110 35.350 59.812 1.00 0.00 H

ATOM 393 CE2 PHE 25 54.033 34.955 61.909 1.00 0.00 C

ATOM 394 HE2 PHE 25 54.980 35.427 62.108 1.00 0.00 H

ATOM 395 CD2 PHE 25 53.280 34.441 62.946 1.00 0.00 C

ATOM 396 HD2 PHE 25 53.663 34.497 63.952 1.00 0.00 H

ATOM 397 C PHE 25 50.850 31.469 65.481 1.00 0.00 C

ATOM 398 O PHE 25 50.692 32.378 66.348 1.00 0.00 O

ATOM 399 N SER 26 50.413 30.247 65.679 1.00 0.00 N

ATOM 400 H SER 26 50.572 29.549 64.967 1.00 0.00 H

ATOM 401 CA SER 26 49.737 29.799 66.953 1.00 0.00 C

ATOM 402 HA SER 26 48.839 30.404 67.084 1.00 0.00 H

ATOM 403 CB SER 26 49.308 28.351 66.815 1.00 0.00 C

ATOM 404 HB2 SER 26 48.728 28.064 67.693 1.00 0.00 H

ATOM 405 HB3 SER 26 48.680 28.238 65.931 1.00 0.00 H

ATOM 406 OG SER 26 50.420 27.505 66.709 1.00 0.00 O

ATOM 407 HG SER 26 51.025 27.672 67.438 1.00 0.00 H

ATOM 408 C SER 26 50.581 29.929 68.232 1.00 0.00 C

ATOM 409 O SER 26 50.002 29.996 69.357 1.00 0.00 O

ATOM 410 N GLU 27 51.900 30.044 68.081 1.00 0.00 N

ATOM 411 H GLU 27 52.269 30.073 67.143 1.00 0.00 H

ATOM 412 CA GLU 27 52.877 30.283 69.227 1.00 0.00 C

ATOM 413 HA GLU 27 52.608 29.617 70.048 1.00 0.00 H

ATOM 414 CB GLU 27 54.298 29.937 68.779 1.00 0.00 C

ATOM 415 HB2 GLU 27 54.543 30.477 67.863 1.00 0.00 H

ATOM 416 HB3 GLU 27 54.999 30.267 69.546 1.00 0.00 H

ATOM 417 CG GLU 27 54.552 28.448 68.588 1.00 0.00 C

ATOM 418 HG2 GLU 27 55.627 28.295 68.483 1.00 0.00 H

| | | | | | | | | | | |
|---|---|---|---|---|---|---|---|---|---|---|
| ATOM | 419 | HG3 | GLU | 27 | 54.217 | 27.906 | 69.474 | 1.00 | 0.00 | H |
| ATOM | 420 | CD | GLU | 27 | 53.871 | 27.891 | 67.369 | 1.00 | 0.00 | C |
| ATOM | 421 | OE1 | GLU | 27 | 53.067 | 28.583 | 66.792 | 1.00 | 0.00 | O |
| ATOM | 422 | OE2 | GLU | 27 | 54.156 | 26.771 | 67.014 | 1.00 | 0.00 | O |
| ATOM | 423 | C | GLU | 27 | 52.887 | 31.726 | 69.799 | 1.00 | 0.00 | C |
| ATOM | 424 | O | GLU | 27 | 53.545 | 31.929 | 70.770 | 1.00 | 0.00 | O |
| ATOM | 425 | N | LEU | 28 | 52.238 | 32.650 | 69.112 | 1.00 | 0.00 | N |
| ATOM | 426 | H | LEU | 28 | 51.742 | 32.401 | 68.267 | 1.00 | 0.00 | H |
| ATOM | 427 | CA | LEU | 28 | 52.219 | 34.051 | 69.572 | 1.00 | 0.00 | C |
| ATOM | 428 | HA | LEU | 28 | 53.233 | 34.351 | 69.841 | 1.00 | 0.00 | H |
| ATOM | 429 | CB | LEU | 28 | 51.726 | 34.963 | 68.442 | 1.00 | 0.00 | C |
| ATOM | 430 | HB2 | LEU | 28 | 50.971 | 34.435 | 67.863 | 1.00 | 0.00 | H |
| ATOM | 431 | HB3 | LEU | 28 | 51.237 | 35.829 | 68.892 | 1.00 | 0.00 | H |
| ATOM | 432 | CG | LEU | 28 | 52.812 | 35.508 | 67.505 | 1.00 | 0.00 | C |
| ATOM | 433 | HG | LEU | 28 | 53.517 | 36.102 | 68.085 | 1.00 | 0.00 | H |
| ATOM | 434 | CD1 | LEU | 28 | 53.554 | 34.347 | 66.858 | 1.00 | 0.00 | C |
| ATOM | 435 | HD11 | LEU | 28 | 54.279 | 34.750 | 66.154 | 1.00 | 0.00 | H |
| ATOM | 436 | HD12 | LEU | 28 | 54.098 | 33.765 | 67.600 | 1.00 | 0.00 | H |
| ATOM | 437 | HD13 | LEU | 28 | 52.869 | 33.694 | 66.327 | 1.00 | 0.00 | H |
| ATOM | 438 | CD2 | LEU | 28 | 52.173 | 36.403 | 66.454 | 1.00 | 0.00 | C |
| ATOM | 439 | HD21 | LEU | 28 | 51.438 | 35.853 | 65.870 | 1.00 | 0.00 | H |
| ATOM | 440 | HD22 | LEU | 28 | 51.671 | 37.238 | 66.941 | 1.00 | 0.00 | H |
| ATOM | 441 | HD23 | LEU | 28 | 52.943 | 36.797 | 65.791 | 1.00 | 0.00 | H |
| ATOM | 442 | C | LEU | 28 | 51.340 | 34.264 | 70.808 | 1.00 | 0.00 | C |
| ATOM | 443 | O | LEU | 28 | 51.630 | 35.007 | 71.755 | 1.00 | 0.00 | O |
| ATOM | 444 | N | GLN | 29 | 50.230 | 33.487 | 70.790 | 1.00 | 0.00 | N |
| ATOM | 445 | H | GLN | 29 | 50.115 | 32.874 | 69.996 | 1.00 | 0.00 | H |
| ATOM | 446 | CA | GLN | 29 | 49.174 | 33.451 | 71.759 | 1.00 | 0.00 | C |
| ATOM | 447 | HA | GLN | 29 | 48.683 | 34.426 | 71.754 | 1.00 | 0.00 | H |
| ATOM | 448 | CB | GLN | 29 | 48.143 | 32.405 | 71.328 | 1.00 | 0.00 | C |

```
ATOM    449  HB2 GLN    29      47.679  32.764  70.412  1.00  0.00           H
ATOM    450  HB3 GLN    29      48.625  31.461  71.088  1.00  0.00           H
ATOM    451  CG  GLN    29      47.050  32.149  72.352  1.00  0.00           C
ATOM    452  HG2 GLN    29      47.484  31.648  73.219  1.00  0.00           H
ATOM    453  HG3 GLN    29      46.634  33.098  72.689  1.00  0.00           H
ATOM    454  CD  GLN    29      45.928  31.292  71.799  1.00  0.00           C
ATOM    455  OE1 GLN    29      45.475  31.491  70.668  1.00  0.00           O
ATOM    456  NE2 GLN    29      45.472  30.331  72.594  1.00  0.00           N
ATOM    457 HE21 GLN    29      44.762  29.705  72.248  1.00  0.00           H
ATOM    458 HE22 GLN    29      45.859  30.212  73.517  1.00  0.00           H
ATOM    459  C   GLN    29      49.646  33.164  73.204  1.00  0.00           C
ATOM    460  O   GLN    29      49.299  33.987  74.039  1.00  0.00           O
ATOM    461  N   PRO    30      50.445  32.090  73.551  1.00  0.00           N
ATOM    462  CD  PRO    30      50.807  30.986  72.622  1.00  0.00           C
ATOM    463  HD2 PRO    30      51.278  31.341  71.715  1.00  0.00           H
ATOM    464  HD3 PRO    30      49.926  30.386  72.400  1.00  0.00           H
ATOM    465  CG  PRO    30      51.762  30.158  73.441  1.00  0.00           C
ATOM    466  HG2 PRO    30      52.786  30.470  73.229  1.00  0.00           H
ATOM    467  HG3 PRO    30      51.645  29.093  73.237  1.00  0.00           H
ATOM    468  CB  PRO    30      51.421  30.489  74.854  1.00  0.00           C
ATOM    469  HB2 PRO    30      52.238  30.232  75.530  1.00  0.00           H
ATOM    470  HB3 PRO    30      50.524  29.939  75.145  1.00  0.00           H
ATOM    471  CA  PRO    30      51.105  31.987  74.823  1.00  0.00           C
ATOM    472  HA  PRO    30      50.389  32.205  75.617  1.00  0.00           H
ATOM    473  C   PRO    30      52.348  32.770  75.107  1.00  0.00           C
ATOM    474  O   PRO    30      52.934  32.576  76.199  1.00  0.00           O
ATOM    475  N   ASN    31      52.697  33.687  74.247  1.00  0.00           N
ATOM    476  H   ASN    31      52.125  33.824  73.426  1.00  0.00           H
ATOM    477  CA  ASN    31      53.959  34.420  74.310  1.00  0.00           C
ATOM    478  HA  ASN    31      53.938  35.094  73.454  1.00  0.00           H
```

ATOM 479 CB ASN 31 53.980 35.226 75.596 1.00 0.00 C

ATOM 480 HB2 ASN 31 53.003 35.690 75.742 1.00 0.00 H

ATOM 481 HB3 ASN 31 54.196 34.596 76.458 1.00 0.00 H

ATOM 482 CG ASN 31 54.999 36.331 75.568 1.00 0.00 C

ATOM 483 OD1 ASN 31 55.550 36.646 76.713 1.00 0.00 O

ATOM 484 ND2 ASN 31 55.288 36.900 74.509 1.00 0.00 N

ATOM 485 HD21 ASN 31 54.845 36.635 73.644 1.00 0.00 H

ATOM 486 HD22 ASN 31 55.995 37.617 74.529 1.00 0.00 H

ATOM 487 C ASN 31 55.266 33.555 74.192 1.00 0.00 C

ATOM 488 O ASN 31 56.145 33.554 75.044 1.00 0.00 O

ATOM 489 N GLU 32 55.393 32.711 73.166 1.00 0.00 N

ATOM 490 H GLU 32 54.625 32.684 72.509 1.00 0.00 H

ATOM 491 CA GLU 32 56.529 31.861 72.785 1.00 0.00 C

ATOM 492 HA GLU 32 57.354 32.049 73.473 1.00 0.00 H

ATOM 493 CB GLU 32 56.144 30.386 72.915 1.00 0.00 C

ATOM 494 HB2 GLU 32 55.297 30.181 72.259 1.00 0.00 H

ATOM 495 HB3 GLU 32 56.990 29.786 72.580 1.00 0.00 H

ATOM 496 CG GLU 32 55.795 29.950 74.331 1.00 0.00 C

ATOM 497 HG2 GLU 32 56.634 30.185 74.988 1.00 0.00 H

ATOM 498 HG3 GLU 32 54.922 30.506 74.677 1.00 0.00 H

ATOM 499 CD GLU 32 55.497 28.480 74.434 1.00 0.00 C

ATOM 500 OE1 GLU 32 55.583 27.804 73.436 1.00 0.00 O

ATOM 501 OE2 GLU 32 55.184 28.031 75.511 1.00 0.00 O

ATOM 502 C GLU 32 57.036 32.116 71.379 1.00 0.00 C

ATOM 503 O GLU 32 56.320 32.685 70.622 1.00 0.00 O

ATOM 504 N VAL 33 58.329 31.838 71.153 1.00 0.00 N

ATOM 505 H VAL 33 58.841 31.452 71.932 1.00 0.00 H

ATOM 506 CA VAL 33 59.070 31.841 69.859 1.00 0.00 C

ATOM 507 HA VAL 33 60.086 31.496 70.054 1.00 0.00 H

ATOM 508 CB VAL 33 58.410 30.872 68.860 1.00 0.00 C

ATOM 509 HB VAL 33 57.360 31.134 68.724 1.00 0.00 H

ATOM 510 CG1 VAL 33 59.083 30.970 67.499 1.00 0.00 C

ATOM 511 HG11 VAL 33 58.683 30.199 66.840 1.00 0.00 H

ATOM 512 HG12 VAL 33 58.893 31.928 67.020 1.00 0.00 H

ATOM 513 HG13 VAL 33 60.158 30.817 67.601 1.00 0.00 H

ATOM 514 CG2 VAL 33 58.479 29.450 69.394 1.00 0.00 C

ATOM 515 HG21 VAL 33 58.060 28.763 68.659 1.00 0.00 H

ATOM 516 HG22 VAL 33 59.514 29.168 69.590 1.00 0.00 H

ATOM 517 HG23 VAL 33 57.896 29.353 70.309 1.00 0.00 H

ATOM 518 C VAL 33 59.163 33.163 69.194 1.00 0.00 C

ATOM 519 O VAL 33 60.230 33.623 68.791 1.00 0.00 O

ATOM 520 N VAL 34 58.096 33.922 69.046 1.00 0.00 N

ATOM 521 H VAL 34 57.212 33.589 69.395 1.00 0.00 H

ATOM 522 CA VAL 34 58.058 35.160 68.259 1.00 0.00 C

ATOM 523 HA VAL 34 58.299 34.900 67.237 1.00 0.00 H

ATOM 524 CB VAL 34 56.647 35.776 68.302 1.00 0.00 C

ATOM 525 HB VAL 34 55.939 35.007 68.008 1.00 0.00 H

ATOM 526 CG1 VAL 34 56.311 36.240 69.711 1.00 0.00 C

ATOM 527 HG11 VAL 34 55.238 36.421 69.769 1.00 0.00 H

ATOM 528 HG12 VAL 34 56.554 35.481 70.455 1.00 0.00 H

ATOM 529 HG13 VAL 34 56.807 37.179 69.956 1.00 0.00 H

ATOM 530 CG2 VAL 34 56.557 36.932 67.317 1.00 0.00 C

ATOM 531 HG21 VAL 34 55.532 37.295 67.279 1.00 0.00 H

ATOM 532 HG22 VAL 34 57.213 37.751 67.610 1.00 0.00 H

ATOM 533 HG23 VAL 34 56.821 36.590 66.321 1.00 0.00 H

ATOM 534 C VAL 34 59.099 36.178 68.810 1.00 0.00 C

ATOM 535 O VAL 34 59.126 36.385 69.975 1.00 0.00 O

ATOM 536 N HIE 35 59.896 36.854 67.965 1.00 0.00 N

ATOM 537 H HIE 35 59.858 36.662 66.973 1.00 0.00 H

ATOM 538 CA HIE 35 60.834 37.850 68.450 1.00 0.00 C

ATOM 539 HA HIE 35 60.893 37.847 69.539 1.00 0.00 H

ATOM 540 CB HIE 35 62.245 37.595 67.910 1.00 0.00 C

ATOM 541 HB2 HIE 35 62.200 37.476 66.826 1.00 0.00 H

ATOM 542 HB3 HIE 35 62.876 38.457 68.128 1.00 0.00 H

ATOM 543 CG HIE 35 62.910 36.394 68.507 1.00 0.00 C

ATOM 544 ND1 HIE 35 64.133 35.927 68.071 1.00 0.00 N

ATOM 545 CE1 HIE 35 64.469 34.861 68.777 1.00 0.00 C

ATOM 546 HE1 HIE 35 65.366 34.273 68.642 1.00 0.00 H

ATOM 547 NE2 HIE 35 63.510 34.620 69.651 1.00 0.00 N

ATOM 548 HE2 HIE 35 63.490 33.846 70.299 1.00 0.00 H

ATOM 549 CD2 HIE 35 62.523 35.564 69.504 1.00 0.00 C

ATOM 550 HD2 HIE 35 61.610 35.620 70.076 1.00 0.00 H

ATOM 551 C HIE 35 60.348 39.224 68.028 1.00 0.00 C

ATOM 552 O HIE 35 59.618 39.383 67.061 1.00 0.00 O

ATOM 553 N ARG 36 60.921 40.282 68.636 1.00 0.00 N

ATOM 554 H ARG 36 61.587 40.131 69.380 1.00 0.00 H

ATOM 555 CA ARG 36 60.634 41.721 68.203 1.00 0.00 C

ATOM 556 HA ARG 36 59.659 41.770 67.714 1.00 0.00 H

ATOM 557 CB ARG 36 60.623 42.689 69.376 1.00 0.00 C

ATOM 558 HB2 ARG 36 59.847 42.363 70.071 1.00 0.00 H

ATOM 559 HB3 ARG 36 61.580 42.627 69.897 1.00 0.00 H

ATOM 560 CG ARG 36 60.357 44.140 69.007 1.00 0.00 C

ATOM 561 HG2 ARG 36 61.141 44.495 68.337 1.00 0.00 H

ATOM 562 HG3 ARG 36 59.398 44.207 68.490 1.00 0.00 H

ATOM 563 CD ARG 36 60.328 45.016 70.206 1.00 0.00 C

ATOM 564 HD2 ARG 36 59.502 44.708 70.850 1.00 0.00 H

ATOM 565 HD3 ARG 36 61.265 44.898 70.752 1.00 0.00 H

ATOM 566 NE ARG 36 60.164 46.416 69.851 1.00 0.00 N

ATOM 567 HE ARG 36 60.108 46.637 68.867 1.00 0.00 H

ATOM 568 CZ ARG 36 60.079 47.426 70.739 1.00 0.00 C

ATOM 569 NH1 ARG 36 60.145 47.175 72.027 1.00 0.00 N
ATOM 570 HH11 ARG 36 60.289 46.228 72.343 1.00 0.00 H
ATOM 571 HH12 ARG 36 60.036 47.923 72.694 1.00 0.00 H
ATOM 572 NH2 ARG 36 59.931 48.668 70.314 1.00 0.00 N
ATOM 573 HH21 ARG 36 59.849 49.425 70.975 1.00 0.00 H
ATOM 574 HH22 ARG 36 59.877 48.861 69.325 1.00 0.00 H
ATOM 575 C ARG 36 61.722 42.140 67.212 1.00 0.00 C
ATOM 576 O ARG 36 62.878 42.231 67.584 1.00 0.00 O
ATOM 577 N LYS 37 61.305 42.329 65.953 1.00 0.00 N
ATOM 578 H LYS 37 60.321 42.218 65.746 1.00 0.00 H
ATOM 579 CA LYS 37 62.207 42.669 64.796 1.00 0.00 C
ATOM 580 HA LYS 37 63.236 42.542 65.130 1.00 0.00 H
ATOM 581 CB LYS 37 61.957 41.736 63.611 1.00 0.00 C
ATOM 582 HB2 LYS 37 61.929 40.705 63.965 1.00 0.00 H
ATOM 583 HB3 LYS 37 60.982 41.980 63.192 1.00 0.00 H
ATOM 584 CG LYS 37 62.991 41.843 62.497 1.00 0.00 C
ATOM 585 HG2 LYS 37 62.628 41.265 61.645 1.00 0.00 H
ATOM 586 HG3 LYS 37 63.102 42.876 62.178 1.00 0.00 H
ATOM 587 CD LYS 37 64.332 41.270 62.931 1.00 0.00 C
ATOM 588 HD2 LYS 37 64.750 41.850 63.754 1.00 0.00 H
ATOM 589 HD3 LYS 37 64.190 40.242 63.266 1.00 0.00 H
ATOM 590 CE LYS 37 65.330 41.264 61.783 1.00 0.00 C
ATOM 591 HE2 LYS 37 66.228 40.720 62.078 1.00 0.00 H
ATOM 592 HE3 LYS 37 64.871 40.747 60.939 1.00 0.00 H
ATOM 593 NZ LYS 37 65.703 42.642 61.362 1.00 0.00 N
ATOM 594 HZ1 LYS 37 66.050 42.644 60.416 1.00 0.00 H
ATOM 595 HZ2 LYS 37 64.901 43.252 61.426 1.00 0.00 H
ATOM 596 HZ3 LYS 37 66.407 43.017 61.983 1.00 0.00 H
ATOM 597 C LYS 37 62.021 44.114 64.348 1.00 0.00 C
ATOM 598 O LYS 37 60.901 44.537 64.173 1.00 0.00 O

| | | | | | | | | | | |
|---|---|---|---|---|---|---|---|---|---|---|
| ATOM | 599 | N | ASP | 38 | 63.101 | 44.761 | 64.021 | 1.00 | 0.00 | N |
| ATOM | 600 | H | ASP | 38 | 63.978 | 44.274 | 64.134 | 1.00 | 0.00 | H |
| ATOM | 601 | CA | ASP | 38 | 63.225 | 46.142 | 63.527 | 1.00 | 0.00 | C |
| ATOM | 602 | HA | ASP | 38 | 62.572 | 46.769 | 64.132 | 1.00 | 0.00 | H |
| ATOM | 603 | CB | ASP | 38 | 64.659 | 46.631 | 63.745 | 1.00 | 0.00 | C |
| ATOM | 604 | HB2 | ASP | 38 | 64.760 | 47.661 | 63.400 | 1.00 | 0.00 | H |
| ATOM | 605 | HB3 | ASP | 38 | 64.864 | 46.627 | 64.816 | 1.00 | 0.00 | H |
| ATOM | 606 | CG | ASP | 38 | 65.696 | 45.749 | 63.062 | 1.00 | 0.00 | C |
| ATOM | 607 | OD1 | ASP | 38 | 65.494 | 44.559 | 63.011 | 1.00 | 0.00 | O |
| ATOM | 608 | OD2 | ASP | 38 | 66.680 | 46.274 | 62.598 | 1.00 | 0.00 | O |
| ATOM | 609 | C | ASP | 38 | 62.871 | 46.390 | 62.082 | 1.00 | 0.00 | C |
| ATOM | 610 | O | ASP | 38 | 63.684 | 46.684 | 61.216 | 1.00 | 0.00 | O |
| ATOM | 611 | N | MET | 39 | 61.625 | 46.079 | 61.721 | 1.00 | 0.00 | N |
| ATOM | 612 | H | MET | 39 | 61.000 | 45.764 | 62.450 | 1.00 | 0.00 | H |
| ATOM | 613 | CA | MET | 39 | 61.028 | 46.261 | 60.452 | 1.00 | 0.00 | C |
| ATOM | 614 | HA | MET | 39 | 61.691 | 46.840 | 59.808 | 1.00 | 0.00 | H |
| ATOM | 615 | CB | MET | 39 | 60.761 | 44.914 | 59.783 | 1.00 | 0.00 | C |
| ATOM | 616 | HB2 | MET | 39 | 60.161 | 44.298 | 60.455 | 1.00 | 0.00 | H |
| ATOM | 617 | HB3 | MET | 39 | 60.175 | 45.090 | 58.883 | 1.00 | 0.00 | H |
| ATOM | 618 | CG | MET | 39 | 62.015 | 44.137 | 59.408 | 1.00 | 0.00 | C |
| ATOM | 619 | HG2 | MET | 39 | 62.626 | 44.738 | 58.735 | 1.00 | 0.00 | H |
| ATOM | 620 | HG3 | MET | 39 | 62.593 | 43.944 | 60.309 | 1.00 | 0.00 | H |
| ATOM | 621 | SD | MET | 39 | 61.645 | 42.559 | 58.618 | 1.00 | 0.00 | S |
| ATOM | 622 | CE | MET | 39 | 61.044 | 43.111 | 57.024 | 1.00 | 0.00 | C |
| ATOM | 623 | HE1 | MET | 39 | 60.936 | 42.250 | 56.366 | 1.00 | 0.00 | H |
| ATOM | 624 | HE2 | MET | 39 | 60.077 | 43.597 | 57.135 | 1.00 | 0.00 | H |
| ATOM | 625 | HE3 | MET | 39 | 61.756 | 43.811 | 56.585 | 1.00 | 0.00 | H |
| ATOM | 626 | C | MET | 39 | 59.766 | 47.041 | 60.619 | 1.00 | 0.00 | C |
| ATOM | 627 | O | MET | 39 | 59.122 | 46.960 | 61.679 | 1.00 | 0.00 | O |
| ATOM | 628 | N | GLY | 40 | 59.303 | 47.836 | 59.644 | 1.00 | 0.00 | N |

```
ATOM    629  H   GLY    40      59.836  47.914  58.790  1.00  0.00           H
ATOM    630  CA  GLY    40      58.037  48.572  59.689  1.00  0.00           C
ATOM    631  HA2 GLY    40      57.891  48.931  60.707  1.00  0.00           H
ATOM    632  HA3 GLY    40      58.123  49.444  59.042  1.00  0.00           H
ATOM    633  C   GLY    40      56.838  47.756  59.217  1.00  0.00           C
ATOM    634  O   GLY    40      56.865  46.805  58.458  1.00  0.00           O
ATOM    635  N   PRO    41      55.658  48.145  59.741  1.00  0.00           N
ATOM    636  CD  PRO    41      55.411  49.220  60.742  1.00  0.00           C
ATOM    637  HD2 PRO    41      55.836  50.176  60.435  1.00  0.00           H
ATOM    638  HD3 PRO    41      55.797  48.900  61.705  1.00  0.00           H
ATOM    639  CG  PRO    41      53.909  49.289  60.815  1.00  0.00           C
ATOM    640  HG2 PRO    41      53.547  49.992  60.064  1.00  0.00           H
ATOM    641  HG3 PRO    41      53.570  49.592  61.807  1.00  0.00           H
ATOM    642  CB  PRO    41      53.457  47.910  60.476  1.00  0.00           C
ATOM    643  HB2 PRO    41      52.425  47.911  60.126  1.00  0.00           H
ATOM    644  HB3 PRO    41      53.560  47.268  61.352  1.00  0.00           H
ATOM    645  CA  PRO    41      54.435  47.450  59.389  1.00  0.00           C
ATOM    646  HA  PRO    41      54.559  46.367  59.443  1.00  0.00           H
ATOM    647  C   PRO    41      53.965  47.856  57.986  1.00  0.00           C
ATOM    648  O   PRO    41      54.496  48.791  57.364  1.00  0.00           O
ATOM    649  N   LEU    42      52.909  47.192  57.469  1.00  0.00           N
ATOM    650  H   LEU    42      52.530  46.454  58.045  1.00  0.00           H
ATOM    651  CA  LEU    42      52.200  47.408  56.205  1.00  0.00           C
ATOM    652  HA  LEU    42      52.739  48.145  55.608  1.00  0.00           H
ATOM    653  CB  LEU    42      52.101  46.098  55.413  1.00  0.00           C
ATOM    654  HB2 LEU    42      51.475  45.404  55.976  1.00  0.00           H
ATOM    655  HB3 LEU    42      51.598  46.304  54.466  1.00  0.00           H
ATOM    656  CG  LEU    42      53.431  45.389  55.129  1.00  0.00           C
ATOM    657  HG  LEU    42      53.933  45.156  56.068  1.00  0.00           H
ATOM    658  CD1 LEU    42      53.164  44.079  54.401  1.00  0.00           C
```

ATOM 659 HD11 LEU 42 54.103 43.547 54.255 1.00 0.00 H
ATOM 660 HD12 LEU 42 52.481 43.461 54.980 1.00 0.00 H
ATOM 661 HD13 LEU 42 52.714 44.291 53.433 1.00 0.00 H
ATOM 662 CD2 LEU 42 54.326 46.301 54.303 1.00 0.00 C
ATOM 663 HD21 LEU 42 55.236 45.767 54.026 1.00 0.00 H
ATOM 664 HD22 LEU 42 53.797 46.595 53.401 1.00 0.00 H
ATOM 665 HD23 LEU 42 54.613 47.185 54.872 1.00 0.00 H
ATOM 666 C LEU 42 50.795 47.965 56.486 1.00 0.00 C
ATOM 667 O LEU 42 49.770 47.331 56.314 1.00 0.00 O
ATOM 668 N VAL 43 50.749 49.284 56.828 1.00 0.00 N
ATOM 669 H VAL 43 51.616 49.796 56.912 1.00 0.00 H
ATOM 670 CA VAL 43 49.496 49.994 57.166 1.00 0.00 C
ATOM 671 HA VAL 43 48.831 49.349 57.724 1.00 0.00 H
ATOM 672 CB VAL 43 49.787 51.229 58.038 1.00 0.00 C
ATOM 673 HB VAL 43 50.474 51.888 57.505 1.00 0.00 H
ATOM 674 CG1 VAL 43 48.505 52.000 58.315 1.00 0.00 C
ATOM 675 HG11 VAL 43 48.722 52.823 58.997 1.00 0.00 H
ATOM 676 HG12 VAL 43 48.097 52.429 57.399 1.00 0.00 H
ATOM 677 HG13 VAL 43 47.763 51.349 58.779 1.00 0.00 H
ATOM 678 CG2 VAL 43 50.449 50.800 59.339 1.00 0.00 C
ATOM 679 HG21 VAL 43 49.804 50.107 59.880 1.00 0.00 H
ATOM 680 HG22 VAL 43 51.408 50.322 59.142 1.00 0.00 H
ATOM 681 HG23 VAL 43 50.631 51.675 59.963 1.00 0.00 H
ATOM 682 C VAL 43 48.778 50.429 55.873 1.00 0.00 C
ATOM 683 O VAL 43 49.404 50.876 54.892 1.00 0.00 O
ATOM 684 N PHE 44 47.515 50.051 55.716 1.00 0.00 N
ATOM 685 H PHE 44 47.041 49.583 56.475 1.00 0.00 H
ATOM 686 CA PHE 44 46.744 50.353 54.535 1.00 0.00 C
ATOM 687 HA PHE 44 47.420 50.457 53.692 1.00 0.00 H
ATOM 688 CB PHE 44 45.913 49.106 54.230 1.00 0.00 C

ATOM 689 HB2 PHE 44 45.380 48.783 55.127 1.00 0.00 H
ATOM 690 HB3 PHE 44 45.157 49.362 53.487 1.00 0.00 H
ATOM 691 CG PHE 44 46.716 47.963 53.677 1.00 0.00 C
ATOM 692 CD1 PHE 44 47.341 47.061 54.526 1.00 0.00 C
ATOM 693 HD1 PHE 44 47.257 47.183 55.595 1.00 0.00 H
ATOM 694 CE1 PHE 44 48.081 46.010 54.018 1.00 0.00 C
ATOM 695 HE1 PHE 44 48.553 45.308 54.689 1.00 0.00 H
ATOM 696 CZ PHE 44 48.203 45.847 52.655 1.00 0.00 C
ATOM 697 HZ PHE 44 48.809 45.055 52.256 1.00 0.00 H
ATOM 698 CE2 PHE 44 47.586 46.736 51.798 1.00 0.00 C
ATOM 699 HE2 PHE 44 47.685 46.617 50.729 1.00 0.00 H
ATOM 700 CD2 PHE 44 46.849 47.787 52.308 1.00 0.00 C
ATOM 701 HD2 PHE 44 46.369 48.474 51.634 1.00 0.00 H
ATOM 702 C PHE 44 45.798 51.583 54.459 1.00 0.00 C
ATOM 703 O PHE 44 44.692 51.610 55.053 1.00 0.00 O
ATOM 704 N SER 45 46.179 52.591 53.700 1.00 0.00 N
ATOM 705 H SER 45 47.067 52.491 53.229 1.00 0.00 H
ATOM 706 CA SER 45 45.510 53.879 53.485 1.00 0.00 C
ATOM 707 HA SER 45 44.441 53.712 53.364 1.00 0.00 H
ATOM 708 CB SER 45 45.727 54.808 54.664 1.00 0.00 C
ATOM 709 HB2 SER 45 45.318 54.346 55.563 1.00 0.00 H
ATOM 710 HB3 SER 45 46.795 54.977 54.808 1.00 0.00 H
ATOM 711 OG SER 45 45.089 56.039 54.458 1.00 0.00 O
ATOM 712 HG SER 45 45.544 56.511 53.756 1.00 0.00 H
ATOM 713 C SER 45 46.060 54.505 52.210 1.00 0.00 C
ATOM 714 O SER 45 47.258 54.432 51.989 1.00 0.00 O
ATOM 715 N ARG 46 45.333 55.274 51.427 1.00 0.00 N
ATOM 716 H ARG 46 44.359 55.428 51.647 1.00 0.00 H
ATOM 717 CA ARG 46 45.862 55.902 50.187 1.00 0.00 C
ATOM 718 HA ARG 46 46.949 55.976 50.218 1.00 0.00 H

| | | | | | | | | | | |
|---|---|---|---|---|---|---|---|---|---|---|
| ATOM | 719 | CB | ARG | 46 | 45.482 | 55.102 | 48.950 | 1.00 | 0.00 | C |
| ATOM | 720 | HB2 | ARG | 46 | 46.046 | 55.474 | 48.100 | 1.00 | 0.00 | H |
| ATOM | 721 | HB3 | ARG | 46 | 45.784 | 54.065 | 49.105 | 1.00 | 0.00 | H |
| ATOM | 722 | CG | ARG | 46 | 44.005 | 55.139 | 48.593 | 1.00 | 0.00 | C |
| ATOM | 723 | HG2 | ARG | 46 | 43.392 | 54.797 | 49.423 | 1.00 | 0.00 | H |
| ATOM | 724 | HG3 | ARG | 46 | 43.772 | 56.169 | 48.369 | 1.00 | 0.00 | H |
| ATOM | 725 | CD | ARG | 46 | 43.715 | 54.350 | 47.368 | 1.00 | 0.00 | C |
| ATOM | 726 | HD2 | ARG | 46 | 44.410 | 54.651 | 46.582 | 1.00 | 0.00 | H |
| ATOM | 727 | HD3 | ARG | 46 | 43.865 | 53.291 | 47.583 | 1.00 | 0.00 | H |
| ATOM | 728 | NE | ARG | 46 | 42.357 | 54.562 | 46.894 | 1.00 | 0.00 | N |
| ATOM | 729 | HE | ARG | 46 | 41.770 | 55.176 | 47.440 | 1.00 | 0.00 | H |
| ATOM | 730 | CZ | ARG | 46 | 41.844 | 54.016 | 45.775 | 1.00 | 0.00 | C |
| ATOM | 731 | NH1 | ARG | 46 | 42.586 | 53.230 | 45.026 | 1.00 | 0.00 | N |
| ATOM | 732 | HH11 | ARG | 46 | 43.546 | 53.049 | 45.281 | 1.00 | 0.00 | H |
| ATOM | 733 | HH12 | ARG | 46 | 42.195 | 52.822 | 44.192 | 1.00 | 0.00 | H |
| ATOM | 734 | NH2 | ARG | 46 | 40.594 | 54.272 | 45.428 | 1.00 | 0.00 | N |
| ATOM | 735 | HH21 | ARG | 46 | 40.194 | 53.839 | 44.607 | 1.00 | 0.00 | H |
| ATOM | 736 | HH22 | ARG | 46 | 40.037 | 54.909 | 45.980 | 1.00 | 0.00 | H |
| ATOM | 737 | C | ARG | 46 | 45.298 | 57.329 | 50.076 | 1.00 | 0.00 | C |
| ATOM | 738 | O | ARG | 46 | 44.210 | 57.573 | 50.519 | 1.00 | 0.00 | O |
| ATOM | 739 | N | ASP | 47 | 45.939 | 58.169 | 49.267 | 1.00 | 0.00 | N |
| ATOM | 740 | H | ASP | 47 | 46.803 | 57.884 | 48.822 | 1.00 | 0.00 | H |
| ATOM | 741 | CA | ASP | 47 | 45.462 | 59.532 | 49.024 | 1.00 | 0.00 | C |
| ATOM | 742 | HA | ASP | 47 | 45.367 | 60.044 | 49.982 | 1.00 | 0.00 | H |
| ATOM | 743 | CB | ASP | 47 | 46.497 | 60.271 | 48.173 | 1.00 | 0.00 | C |
| ATOM | 744 | HB2 | ASP | 47 | 46.768 | 59.665 | 47.307 | 1.00 | 0.00 | H |
| ATOM | 745 | HB3 | ASP | 47 | 46.048 | 61.195 | 47.807 | 1.00 | 0.00 | H |
| ATOM | 746 | CG | ASP | 47 | 47.747 | 60.650 | 48.957 | 1.00 | 0.00 | C |
| ATOM | 747 | OD1 | ASP | 47 | 47.722 | 60.551 | 50.161 | 1.00 | 0.00 | O |
| ATOM | 748 | OD2 | ASP | 47 | 48.714 | 61.033 | 48.344 | 1.00 | 0.00 | O |

| | | | | | | | | | | |
|---|---|---|---|---|---|---|---|---|---|---|
| ATOM | 749 | C | ASP | 47 | 44.039 | 59.613 | 48.328 | 1.00 | 0.00 | C |
| ATOM | 750 | O | ASP | 47 | 43.191 | 60.458 | 48.652 | 1.00 | 0.00 | O |
| ATOM | 751 | N | CYS | 48 | 43.737 | 58.639 | 47.423 | 1.00 | 0.00 | N |
| ATOM | 752 | H | CYS | 48 | 44.426 | 57.936 | 47.197 | 1.00 | 0.00 | H |
| ATOM | 753 | CA | CYS | 48 | 42.460 | 58.615 | 46.745 | 1.00 | 0.00 | C |
| ATOM | 754 | HA | CYS | 48 | 42.189 | 59.638 | 46.479 | 1.00 | 0.00 | H |
| ATOM | 755 | CB | CYS | 48 | 42.585 | 57.826 | 45.441 | 1.00 | 0.00 | C |
| ATOM | 756 | HB2 | CYS | 48 | 43.313 | 58.327 | 44.802 | 1.00 | 0.00 | H |
| ATOM | 757 | HB3 | CYS | 48 | 42.967 | 56.830 | 45.661 | 1.00 | 0.00 | H |
| ATOM | 758 | SG | CYS | 48 | 41.033 | 57.661 | 44.526 | 1.00 | 0.00 | S |
| ATOM | 759 | HG | CYS | 48 | 41.535 | 56.930 | 43.521 | 1.00 | 0.00 | H |
| ATOM | 760 | C | CYS | 48 | 41.333 | 58.019 | 47.565 | 1.00 | 0.00 | C |
| ATOM | 761 | O | CYS | 48 | 41.586 | 56.940 | 48.061 | 1.00 | 0.00 | O |
| ATOM | 762 | N | LYS | 49 | 40.211 | 58.728 | 47.762 | 1.00 | 0.00 | N |
| ATOM | 763 | H | LYS | 49 | 40.104 | 59.638 | 47.338 | 1.00 | 0.00 | H |
| ATOM | 764 | CA | LYS | 49 | 39.131 | 58.189 | 48.585 | 1.00 | 0.00 | C |
| ATOM | 765 | HA | LYS | 49 | 39.446 | 58.241 | 49.628 | 1.00 | 0.00 | H |
| ATOM | 766 | CB | LYS | 49 | 37.858 | 59.019 | 48.414 | 1.00 | 0.00 | C |
| ATOM | 767 | HB2 | LYS | 49 | 38.111 | 60.064 | 48.599 | 1.00 | 0.00 | H |
| ATOM | 768 | HB3 | LYS | 49 | 37.513 | 58.934 | 47.382 | 1.00 | 0.00 | H |
| ATOM | 769 | CG | LYS | 49 | 36.722 | 58.629 | 49.351 | 1.00 | 0.00 | C |
| ATOM | 770 | HG2 | LYS | 49 | 36.396 | 57.612 | 49.133 | 1.00 | 0.00 | H |
| ATOM | 771 | HG3 | LYS | 49 | 37.083 | 58.671 | 50.380 | 1.00 | 0.00 | H |
| ATOM | 772 | CD | LYS | 49 | 35.535 | 59.569 | 49.202 | 1.00 | 0.00 | C |
| ATOM | 773 | HD2 | LYS | 49 | 35.869 | 60.598 | 49.348 | 1.00 | 0.00 | H |
| ATOM | 774 | HD3 | LYS | 49 | 35.125 | 59.470 | 48.195 | 1.00 | 0.00 | H |
| ATOM | 775 | CE | LYS | 49 | 34.449 | 59.257 | 50.221 | 1.00 | 0.00 | C |
| ATOM | 776 | HE2 | LYS | 49 | 34.112 | 58.227 | 50.097 | 1.00 | 0.00 | H |
| ATOM | 777 | HE3 | LYS | 49 | 34.881 | 59.365 | 51.217 | 1.00 | 0.00 | H |
| ATOM | 778 | NZ | LYS | 49 | 33.290 | 60.182 | 50.100 | 1.00 | 0.00 | N |

| | | | | | | | | | | |
|---|---|---|---|---|---|---|---|---|---|---|
| ATOM | 779 | HZ1 | LYS | 49 | 33.619 | 61.120 | 49.929 | 1.00 | 0.00 | H |
| ATOM | 780 | HZ2 | LYS | 49 | 32.702 | 59.889 | 49.333 | 1.00 | 0.00 | H |
| ATOM | 781 | HZ3 | LYS | 49 | 32.756 | 60.172 | 50.957 | 1.00 | 0.00 | H |
| ATOM | 782 | C | LYS | 49 | 38.877 | 56.709 | 48.208 | 1.00 | 0.00 | C |
| ATOM | 783 | O | LYS | 49 | 38.830 | 56.334 | 47.031 | 1.00 | 0.00 | O |
| ATOM | 784 | N | PHE | 50 | 38.707 | 55.830 | 49.241 | 1.00 | 0.00 | N |
| ATOM | 785 | H | PHE | 50 | 38.794 | 56.169 | 50.188 | 1.00 | 0.00 | H |
| ATOM | 786 | CA | PHE | 50 | 38.405 | 54.413 | 49.060 | 1.00 | 0.00 | C |
| ATOM | 787 | HA | PHE | 50 | 39.199 | 53.941 | 48.511 | 1.00 | 0.00 | H |
| ATOM | 788 | CB | PHE | 50 | 38.160 | 53.759 | 50.421 | 1.00 | 0.00 | C |
| ATOM | 789 | HB2 | PHE | 50 | 37.528 | 54.402 | 51.037 | 1.00 | 0.00 | H |
| ATOM | 790 | HB3 | PHE | 50 | 37.631 | 52.819 | 50.261 | 1.00 | 0.00 | H |
| ATOM | 791 | CG | PHE | 50 | 39.420 | 53.415 | 51.164 | 1.00 | 0.00 | C |
| ATOM | 792 | CD1 | PHE | 50 | 40.648 | 53.432 | 50.520 | 1.00 | 0.00 | C |
| ATOM | 793 | HD1 | PHE | 50 | 40.806 | 53.706 | 49.511 | 1.00 | 0.00 | H |
| ATOM | 794 | CE1 | PHE | 50 | 41.808 | 53.115 | 51.202 | 1.00 | 0.00 | C |
| ATOM | 795 | HE1 | PHE | 50 | 42.759 | 53.122 | 50.695 | 1.00 | 0.00 | H |
| ATOM | 796 | CZ | PHE | 50 | 41.752 | 52.778 | 52.538 | 1.00 | 0.00 | C |
| ATOM | 797 | HZ | PHE | 50 | 42.649 | 52.501 | 53.071 | 1.00 | 0.00 | H |
| ATOM | 798 | CE2 | PHE | 50 | 40.537 | 52.757 | 53.191 | 1.00 | 0.00 | C |
| ATOM | 799 | HE2 | PHE | 50 | 40.496 | 52.499 | 54.240 | 1.00 | 0.00 | H |
| ATOM | 800 | CD2 | PHE | 50 | 39.379 | 53.073 | 52.507 | 1.00 | 0.00 | C |
| ATOM | 801 | HD2 | PHE | 50 | 38.435 | 53.049 | 53.031 | 1.00 | 0.00 | H |
| ATOM | 802 | C | PHE | 50 | 37.224 | 54.125 | 48.177 | 1.00 | 0.00 | C |
| ATOM | 803 | O | PHE | 50 | 37.277 | 53.183 | 47.315 | 1.00 | 0.00 | O |
| ATOM | 804 | N | ALA | 51 | 36.163 | 54.903 | 48.386 | 1.00 | 0.00 | N |
| ATOM | 805 | H | ALA | 51 | 36.255 | 55.598 | 49.111 | 1.00 | 0.00 | H |
| ATOM | 806 | CA | ALA | 51 | 34.891 | 54.941 | 47.654 | 1.00 | 0.00 | C |
| ATOM | 807 | HA | ALA | 51 | 34.707 | 53.931 | 47.283 | 1.00 | 0.00 | H |
| ATOM | 808 | CB | ALA | 51 | 33.865 | 55.173 | 48.744 | 1.00 | 0.00 | C |

ATOM 809 HB1 ALA 51 32.865 55.148 48.309 1.00 0.00 H
ATOM 810 HB2 ALA 51 33.934 54.391 49.502 1.00 0.00 H
ATOM 811 HB3 ALA 51 34.023 56.146 49.210 1.00 0.00 H
ATOM 812 C ALA 51 34.886 55.835 46.434 1.00 0.00 C
ATOM 813 O ALA 51 34.383 56.938 46.510 1.00 0.00 O
ATOM 814 N ALA 52 35.517 55.390 45.344 1.00 0.00 N
ATOM 815 H ALA 52 35.910 54.459 45.347 1.00 0.00 H
ATOM 816 CA ALA 52 35.772 56.217 44.155 1.00 0.00 C
ATOM 817 HA ALA 52 35.001 56.988 44.116 1.00 0.00 H
ATOM 818 CB ALA 52 37.171 56.976 44.263 1.00 0.00 C
ATOM 819 HB1 ALA 52 37.304 57.640 43.408 1.00 0.00 H
ATOM 820 HB2 ALA 52 37.197 57.571 45.176 1.00 0.00 H
ATOM 821 HB3 ALA 52 37.982 56.247 44.281 1.00 0.00 H
ATOM 822 C ALA 52 35.678 55.474 42.855 1.00 0.00 C
ATOM 823 O ALA 52 35.882 54.260 42.791 1.00 0.00 O
ATOM 824 N GLU 53 35.545 56.251 41.746 1.00 0.00 N
ATOM 825 H GLU 53 35.368 57.229 41.844 1.00 0.00 H
ATOM 826 CA GLU 53 35.503 55.564 40.403 1.00 0.00 C
ATOM 827 HA GLU 53 34.784 54.744 40.445 1.00 0.00 H
ATOM 828 CB GLU 53 35.070 56.533 39.301 1.00 0.00 C
ATOM 829 HB2 GLU 53 34.980 55.955 38.380 1.00 0.00 H
ATOM 830 HB3 GLU 53 34.081 56.935 39.522 1.00 0.00 H
ATOM 831 CG GLU 53 36.052 57.667 39.039 1.00 0.00 C
ATOM 832 HG2 GLU 53 37.083 57.319 39.054 1.00 0.00 H
ATOM 833 HG3 GLU 53 35.857 58.039 38.032 1.00 0.00 H
ATOM 834 CD GLU 53 35.881 58.819 39.989 1.00 0.00 C
ATOM 835 OE1 GLU 53 35.255 58.638 41.006 1.00 0.00 O
ATOM 836 OE2 GLU 53 36.377 59.882 39.698 1.00 0.00 O
ATOM 837 C GLU 53 36.930 54.976 40.088 1.00 0.00 C
ATOM 838 O GLU 53 36.930 54.058 39.279 1.00 0.00 O

ATOM 839 N LYS 54 38.002 55.315 40.840 1.00 0.00 N
ATOM 840 H LYS 54 37.865 56.004 41.566 1.00 0.00 H
ATOM 841 CA LYS 54 39.312 54.851 40.642 1.00 0.00 C
ATOM 842 HA LYS 54 39.442 54.609 39.591 1.00 0.00 H
ATOM 843 CB LYS 54 40.292 55.983 40.952 1.00 0.00 C
ATOM 844 HB2 LYS 54 40.123 56.326 41.974 1.00 0.00 H
ATOM 845 HB3 LYS 54 41.304 55.579 40.897 1.00 0.00 H
ATOM 846 CG LYS 54 40.199 57.172 40.005 1.00 0.00 C
ATOM 847 HG2 LYS 54 40.384 56.835 38.984 1.00 0.00 H
ATOM 848 HG3 LYS 54 39.199 57.603 40.059 1.00 0.00 H
ATOM 849 CD LYS 54 41.208 58.251 40.371 1.00 0.00 C
ATOM 850 HD2 LYS 54 41.028 58.576 41.397 1.00 0.00 H
ATOM 851 HD3 LYS 54 42.216 57.839 40.303 1.00 0.00 H
ATOM 852 CE LYS 54 41.095 59.453 39.446 1.00 0.00 C
ATOM 853 HE2 LYS 54 41.273 59.131 38.419 1.00 0.00 H
ATOM 854 HE3 LYS 54 40.080 59.850 39.512 1.00 0.00 H
ATOM 855 NZ LYS 54 42.063 60.525 39.804 1.00 0.00 N
ATOM 856 HZ1 LYS 54 42.578 60.259 40.632 1.00 0.00 H
ATOM 857 HZ2 LYS 54 42.709 60.671 39.041 1.00 0.00 H
ATOM 858 HZ3 LYS 54 41.563 61.384 39.988 1.00 0.00 H
ATOM 859 C LYS 54 39.589 53.570 41.529 1.00 0.00 C
ATOM 860 O LYS 54 39.680 53.655 42.723 1.00 0.00 O
ATOM 861 N THR 55 39.668 52.345 40.982 1.00 0.00 N
ATOM 862 H THR 55 39.828 51.587 41.629 1.00 0.00 H
ATOM 863 CA THR 55 39.484 51.934 39.624 1.00 0.00 C
ATOM 864 HA THR 55 39.032 52.733 39.044 1.00 0.00 H
ATOM 865 CB THR 55 40.842 51.609 38.975 1.00 0.00 C
ATOM 866 HB THR 55 40.699 51.429 37.909 1.00 0.00 H
ATOM 867 CG2 THR 55 41.809 52.769 39.153 1.00 0.00 C
ATOM 868 HG21 THR 55 42.715 52.569 38.582 1.00 0.00 H

ATOM 869 HG22 THR 55 41.367 53.688 38.772 1.00 0.00 H
ATOM 870 HG23 THR 55 42.087 52.893 40.199 1.00 0.00 H
ATOM 871 OG1 THR 55 41.395 50.433 39.580 1.00 0.00 O
ATOM 872 HG1 THR 55 40.832 49.685 39.361 1.00 0.00 H
ATOM 873 C THR 55 38.565 50.743 39.485 1.00 0.00 C
ATOM 874 O THR 55 38.000 50.227 40.476 1.00 0.00 O
ATOM 875 N SER 56 38.199 50.364 38.236 1.00 0.00 N
ATOM 876 H SER 56 38.563 50.909 37.469 1.00 0.00 H
ATOM 877 CA SER 56 37.524 49.102 37.840 1.00 0.00 C
ATOM 878 HA SER 56 36.647 48.965 38.475 1.00 0.00 H
ATOM 879 CB SER 56 37.059 49.176 36.398 1.00 0.00 C
ATOM 880 HB2 SER 56 36.451 48.301 36.166 1.00 0.00 H
ATOM 881 HB3 SER 56 36.448 50.069 36.261 1.00 0.00 H
ATOM 882 OG SER 56 38.150 49.221 35.520 1.00 0.00 O
ATOM 883 HG SER 56 38.594 48.370 35.542 1.00 0.00 H
ATOM 884 C SER 56 38.450 47.907 38.011 1.00 0.00 C
ATOM 885 O SER 56 39.710 48.080 38.049 1.00 0.00 O
ATOM 886 N LEU 57 37.817 46.717 38.083 1.00 0.00 N
ATOM 887 H LEU 57 36.809 46.649 38.036 1.00 0.00 H
ATOM 888 CA LEU 57 38.620 45.497 38.313 1.00 0.00 C
ATOM 889 HA LEU 57 39.683 45.699 38.181 1.00 0.00 H
ATOM 890 CB LEU 57 38.407 44.968 39.737 1.00 0.00 C
ATOM 891 HB2 LEU 57 37.342 44.795 39.901 1.00 0.00 H
ATOM 892 HB3 LEU 57 38.890 43.991 39.786 1.00 0.00 H
ATOM 893 CG LEU 57 38.984 45.837 40.862 1.00 0.00 C
ATOM 894 HG LEU 57 39.838 46.400 40.491 1.00 0.00 H
ATOM 895 CD1 LEU 57 37.927 46.827 41.333 1.00 0.00 C
ATOM 896 HD11 LEU 57 38.309 47.386 42.184 1.00 0.00 H
ATOM 897 HD12 LEU 57 37.683 47.535 40.549 1.00 0.00 H
ATOM 898 HD13 LEU 57 37.024 46.292 41.624 1.00 0.00 H

ATOM 899 CD2 LEU 57 39.447 44.947 42.005 1.00 0.00 C
ATOM 900 HD21 LEU 57 39.805 45.564 42.830 1.00 0.00 H
ATOM 901 HD22 LEU 57 38.625 44.320 42.350 1.00 0.00 H
ATOM 902 HD23 LEU 57 40.269 44.319 41.668 1.00 0.00 H
ATOM 903 C LEU 57 38.224 44.417 37.281 1.00 0.00 C
ATOM 904 O LEU 57 37.162 44.505 36.674 1.00 0.00 O
ATOM 905 N LYS 58 39.054 43.424 37.124 1.00 0.00 N
ATOM 906 H LYS 58 39.889 43.419 37.691 1.00 0.00 H
ATOM 907 CA LYS 58 38.842 42.247 36.268 1.00 0.00 C
ATOM 908 HA LYS 58 37.847 42.293 35.821 1.00 0.00 H
ATOM 909 CB LYS 58 39.873 42.228 35.139 1.00 0.00 C
ATOM 910 HB2 LYS 58 40.872 42.231 35.575 1.00 0.00 H
ATOM 911 HB3 LYS 58 39.753 41.300 34.578 1.00 0.00 H
ATOM 912 CG LYS 58 39.758 43.391 34.162 1.00 0.00 C
ATOM 913 HG2 LYS 58 38.755 43.399 33.733 1.00 0.00 H
ATOM 914 HG3 LYS 58 39.920 44.331 34.692 1.00 0.00 H
ATOM 915 CD LYS 58 40.781 43.275 33.042 1.00 0.00 C
ATOM 916 HD2 LYS 58 41.784 43.265 33.472 1.00 0.00 H
ATOM 917 HD3 LYS 58 40.619 42.340 32.503 1.00 0.00 H
ATOM 918 CE LYS 58 40.668 44.437 32.066 1.00 0.00 C
ATOM 919 HE2 LYS 58 39.661 44.447 31.644 1.00 0.00 H
ATOM 920 HE3 LYS 58 40.826 45.371 32.608 1.00 0.00 H
ATOM 921 NZ LYS 58 41.661 44.335 30.962 1.00 0.00 N
ATOM 922 HZ1 LYS 58 41.514 43.475 30.452 1.00 0.00 H
ATOM 923 HZ2 LYS 58 41.551 45.117 30.331 1.00 0.00 H
ATOM 924 HZ3 LYS 58 42.597 44.340 31.342 1.00 0.00 H
ATOM 925 C LYS 58 38.915 40.920 37.115 1.00 0.00 C
ATOM 926 O LYS 58 39.540 40.900 38.174 1.00 0.00 O
ATOM 927 N ILE 59 38.457 39.804 36.543 1.00 0.00 N
ATOM 928 H ILE 59 38.022 39.852 35.634 1.00 0.00 H

ATOM 929 CA ILE 59 38.451 38.500 37.227 1.00 0.00 C

ATOM 930 HA ILE 59 39.100 38.558 38.101 1.00 0.00 H

ATOM 931 CB ILE 59 37.035 38.157 37.727 1.00 0.00 C

ATOM 932 HB ILE 59 36.715 38.962 38.387 1.00 0.00 H

ATOM 933 CG2 ILE 59 36.070 38.047 36.557 1.00 0.00 C

ATOM 934 HG21 ILE 59 35.070 37.928 36.963 1.00 0.00 H

ATOM 935 HG22 ILE 59 36.089 38.944 35.942 1.00 0.00 H

ATOM 936 HG23 ILE 59 36.263 37.188 35.931 1.00 0.00 H

ATOM 937 CG1 ILE 59 37.055 36.856 38.534 1.00 0.00 C

ATOM 938 HG12 ILE 59 37.221 35.988 37.900 1.00 0.00 H

ATOM 939 HG13 ILE 59 37.863 36.917 39.256 1.00 0.00 H

ATOM 940 CD1 ILE 59 35.798 36.619 39.340 1.00 0.00 C

ATOM 941 HD11 ILE 59 34.976 36.302 38.703 1.00 0.00 H

ATOM 942 HD12 ILE 59 35.983 35.846 40.084 1.00 0.00 H

ATOM 943 HD13 ILE 59 35.511 37.535 39.844 1.00 0.00 H

ATOM 944 C ILE 59 38.936 37.358 36.385 1.00 0.00 C

ATOM 945 O ILE 59 38.544 37.229 35.215 1.00 0.00 O

ATOM 946 N GLU 60 39.684 36.419 37.047 1.00 0.00 N

ATOM 947 H GLU 60 39.880 36.570 38.026 1.00 0.00 H

ATOM 948 CA GLU 60 40.232 35.223 36.466 1.00 0.00 C

ATOM 949 HA GLU 60 39.823 35.102 35.462 1.00 0.00 H

ATOM 950 CB GLU 60 41.748 35.378 36.328 1.00 0.00 C

ATOM 951 HB2 GLU 60 41.942 36.284 35.750 1.00 0.00 H

ATOM 952 HB3 GLU 60 42.183 35.507 37.321 1.00 0.00 H

ATOM 953 CG GLU 60 42.438 34.212 35.634 1.00 0.00 C

ATOM 954 HG2 GLU 60 42.296 33.303 36.219 1.00 0.00 H

ATOM 955 HG3 GLU 60 41.980 34.066 34.654 1.00 0.00 H

ATOM 956 CD GLU 60 43.915 34.431 35.452 1.00 0.00 C

ATOM 957 OE1 GLU 60 44.396 35.465 35.848 1.00 0.00 O

ATOM 958 OE2 GLU 60 44.562 33.563 34.915 1.00 0.00 O

| | | | | | | | | | | |
|---|---|---|---|---|---|---|---|---|---|---|
| ATOM | 959 | C | GLU | 60 | 39.916 | 33.954 | 37.255 | 1.00 | 0.00 | C |
| ATOM | 960 | O | GLU | 60 | 40.229 | 33.766 | 38.434 | 1.00 | 0.00 | O |
| ATOM | 961 | N | ASN | 61 | 39.412 | 32.898 | 36.570 | 1.00 | 0.00 | N |
| ATOM | 962 | H | ASN | 61 | 39.224 | 33.034 | 35.588 | 1.00 | 0.00 | H |
| ATOM | 963 | CA | ASN | 61 | 39.053 | 31.617 | 37.092 | 1.00 | 0.00 | C |
| ATOM | 964 | HA | ASN | 61 | 38.948 | 31.679 | 38.176 | 1.00 | 0.00 | H |
| ATOM | 965 | CB | ASN | 61 | 37.743 | 31.113 | 36.516 | 1.00 | 0.00 | C |
| ATOM | 966 | HB2 | ASN | 61 | 36.972 | 31.855 | 36.729 | 1.00 | 0.00 | H |
| ATOM | 967 | HB3 | ASN | 61 | 37.820 | 31.010 | 35.433 | 1.00 | 0.00 | H |
| ATOM | 968 | CG | ASN | 61 | 37.321 | 29.796 | 37.104 | 1.00 | 0.00 | C |
| ATOM | 969 | OD1 | ASN | 61 | 38.161 | 28.938 | 37.403 | 1.00 | 0.00 | O |
| ATOM | 970 | ND2 | ASN | 61 | 36.037 | 29.618 | 37.278 | 1.00 | 0.00 | N |
| ATOM | 971 | HD21 | ASN | 61 | 35.704 | 28.764 | 37.697 | 1.00 | 0.00 | H |
| ATOM | 972 | HD22 | ASN | 61 | 35.389 | 30.354 | 37.030 | 1.00 | 0.00 | H |
| ATOM | 973 | C | ASN | 61 | 40.235 | 30.716 | 36.745 | 1.00 | 0.00 | C |
| ATOM | 974 | O | ASN | 61 | 40.371 | 30.072 | 35.688 | 1.00 | 0.00 | O |
| ATOM | 975 | N | GLU | 62 | 41.013 | 30.385 | 37.718 | 1.00 | 0.00 | N |
| ATOM | 976 | H | GLU | 62 | 40.852 | 30.807 | 38.622 | 1.00 | 0.00 | H |
| ATOM | 977 | CA | GLU | 62 | 42.250 | 29.540 | 37.596 | 1.00 | 0.00 | C |
| ATOM | 978 | HA | GLU | 62 | 42.668 | 29.684 | 36.598 | 1.00 | 0.00 | H |
| ATOM | 979 | CB | GLU | 62 | 43.291 | 30.030 | 38.605 | 1.00 | 0.00 | C |
| ATOM | 980 | HB2 | GLU | 62 | 42.925 | 29.915 | 39.620 | 1.00 | 0.00 | H |
| ATOM | 981 | HB3 | GLU | 62 | 44.170 | 29.395 | 38.500 | 1.00 | 0.00 | H |
| ATOM | 982 | CG | GLU | 62 | 43.753 | 31.464 | 38.385 | 1.00 | 0.00 | C |
| ATOM | 983 | HG2 | GLU | 62 | 44.071 | 31.579 | 37.348 | 1.00 | 0.00 | H |
| ATOM | 984 | HG3 | GLU | 62 | 42.915 | 32.141 | 38.565 | 1.00 | 0.00 | H |
| ATOM | 985 | CD | GLU | 62 | 44.896 | 31.853 | 39.281 | 1.00 | 0.00 | C |
| ATOM | 986 | OE1 | GLU | 62 | 45.605 | 30.981 | 39.721 | 1.00 | 0.00 | O |
| ATOM | 987 | OE2 | GLU | 62 | 45.060 | 33.026 | 39.525 | 1.00 | 0.00 | O |
| ATOM | 988 | C | GLU | 62 | 42.051 | 27.998 | 37.792 | 1.00 | 0.00 | C |

ATOM 989 O GLU 62 43.028 27.236 37.989 1.00 0.00 O

ATOM 990 N VAL 63 40.822 27.530 37.786 1.00 0.00 N

ATOM 991 H VAL 63 40.055 28.170 37.625 1.00 0.00 H

ATOM 992 CA VAL 63 40.479 26.067 38.060 1.00 0.00 C

ATOM 993 HA VAL 63 39.417 25.936 37.850 1.00 0.00 H

ATOM 994 CB VAL 63 41.282 25.143 37.125 1.00 0.00 C

ATOM 995 HB VAL 63 42.356 25.237 37.221 1.00 0.00 H

ATOM 996 CG1 VAL 63 41.024 23.683 37.469 1.00 0.00 C

ATOM 997 HG11 VAL 63 41.526 23.060 36.728 1.00 0.00 H

ATOM 998 HG12 VAL 63 41.436 23.400 38.436 1.00 0.00 H

ATOM 999 HG13 VAL 63 39.965 23.460 37.410 1.00 0.00 H

ATOM 1000 CG2 VAL 63 40.917 25.428 35.676 1.00 0.00 C

ATOM 1001 HG21 VAL 63 39.891 25.119 35.483 1.00 0.00 H

ATOM 1002 HG22 VAL 63 41.025 26.485 35.436 1.00 0.00 H

ATOM 1003 HG23 VAL 63 41.582 24.867 35.020 1.00 0.00 H

ATOM 1004 C VAL 63 40.725 25.580 39.454 1.00 0.00 C

ATOM 1005 O VAL 63 40.015 24.779 40.062 1.00 0.00 O

ATOM 1006 N ASN 64 41.858 25.973 39.973 1.00 0.00 N

ATOM 1007 H ASN 64 42.394 26.605 39.413 1.00 0.00 H

ATOM 1008 CA ASN 64 42.469 25.745 41.310 1.00 0.00 C

ATOM 1009 HA ASN 64 42.209 24.735 41.626 1.00 0.00 H

ATOM 1010 CB ASN 64 43.978 25.840 41.170 1.00 0.00 C

ATOM 1011 HB2 ASN 64 44.245 26.811 40.748 1.00 0.00 H

ATOM 1012 HB3 ASN 64 44.436 25.758 42.155 1.00 0.00 H

ATOM 1013 CG ASN 64 44.547 24.749 40.306 1.00 0.00 C

ATOM 1014 OD1 ASN 64 45.704 24.993 39.744 1.00 0.00 O

ATOM 1015 ND2 ASN 64 43.939 23.684 40.148 1.00 0.00 N

ATOM 1016 HD21 ASN 64 43.025 23.559 40.550 1.00 0.00 H

ATOM 1017 HD22 ASN 64 44.339 22.998 39.527 1.00 0.00 H

ATOM 1018 C ASN 64 41.998 26.672 42.489 1.00 0.00 C

```
ATOM   1019  O   ASN    64      41.729  26.246  43.609  1.00  0.00           O
ATOM   1020  N   THR    65      41.830  27.937  42.150  1.00  0.00           N
ATOM   1021  H   THR    65      42.016  28.160  41.182  1.00  0.00           H
ATOM   1022  CA  THR    65      41.510  29.115  42.972  1.00  0.00           C
ATOM   1023  HA  THR    65      40.820  28.815  43.761  1.00  0.00           H
ATOM   1024  CB  THR    65      42.777  29.694  43.630  1.00  0.00           C
ATOM   1025  HB  THR    65      43.298  28.897  44.161  1.00  0.00           H
ATOM   1026  CG2 THR    65      43.698  30.292  42.579  1.00  0.00           C
ATOM   1027 HG21 THR    65      44.618  30.626  43.060  1.00  0.00           H
ATOM   1028 HG22 THR    65      43.964  29.545  41.831  1.00  0.00           H
ATOM   1029 HG23 THR    65      43.242  31.153  42.089  1.00  0.00           H
ATOM   1030  OG1 THR    65      42.407  30.710  44.571  1.00  0.00           O
ATOM   1031  HG1 THR    65      42.284  31.535  44.094  1.00  0.00           H
ATOM   1032  C   THR    65      40.811  30.200  42.085  1.00  0.00           C
ATOM   1033  O   THR    65      40.825  30.152  40.835  1.00  0.00           O
ATOM   1034  N   ILE    66      40.143  31.206  42.709  1.00  0.00           N
ATOM   1035  H   ILE    66      40.112  31.262  43.717  1.00  0.00           H
ATOM   1036  CA  ILE    66      39.615  32.368  41.898  1.00  0.00           C
ATOM   1037  HA  ILE    66      39.749  32.189  40.833  1.00  0.00           H
ATOM   1038  CB  ILE    66      38.112  32.577  42.158  1.00  0.00           C
ATOM   1039  HB  ILE    66      37.969  32.859  43.203  1.00  0.00           H
ATOM   1040  CG2 ILE    66      37.571  33.694  41.279  1.00  0.00           C
ATOM   1041 HG21 ILE    66      36.482  33.725  41.343  1.00  0.00           H
ATOM   1042 HG22 ILE    66      37.956  34.657  41.617  1.00  0.00           H
ATOM   1043 HG23 ILE    66      37.865  33.552  40.238  1.00  0.00           H
ATOM   1044  CG1 ILE    66      37.342  31.276  41.913  1.00  0.00           C
ATOM   1045 HG12 ILE    66      37.699  30.486  42.573  1.00  0.00           H
ATOM   1046 HG13 ILE    66      36.296  31.443  42.170  1.00  0.00           H
ATOM   1047  CD1 ILE    66      37.402  30.791  40.483  1.00  0.00           C
ATOM   1048 HD11 ILE    66      36.830  29.867  40.407  1.00  0.00           H
```

ATOM 1049 HD12 ILE 66 36.952 31.513 39.803 1.00 0.00 H

ATOM 1050 HD13 ILE 66 38.420 30.576 40.174 1.00 0.00 H

ATOM 1051 C ILE 66 40.411 33.706 42.235 1.00 0.00 C

ATOM 1052 O ILE 66 40.574 34.120 43.389 1.00 0.00 O

ATOM 1053 N SER 67 40.941 34.407 41.221 1.00 0.00 N

ATOM 1054 H SER 67 40.814 34.060 40.280 1.00 0.00 H

ATOM 1055 CA SER 67 41.680 35.677 41.360 1.00 0.00 C

ATOM 1056 HA SER 67 41.857 35.869 42.417 1.00 0.00 H

ATOM 1057 CB SER 67 43.042 35.514 40.715 1.00 0.00 C

ATOM 1058 HB2 SER 67 42.917 35.217 39.674 1.00 0.00 H

ATOM 1059 HB3 SER 67 43.583 36.460 40.748 1.00 0.00 H

ATOM 1060 OG SER 67 43.802 34.545 41.383 1.00 0.00 O

ATOM 1061 HG SER 67 44.306 34.042 40.727 1.00 0.00 H

ATOM 1062 C SER 67 41.032 36.940 40.788 1.00 0.00 C

ATOM 1063 O SER 67 40.245 36.860 39.816 1.00 0.00 O

ATOM 1064 N VAL 68 41.380 38.060 41.439 1.00 0.00 N

ATOM 1065 H VAL 68 42.028 37.984 42.208 1.00 0.00 H

ATOM 1066 CA VAL 68 40.884 39.393 41.114 1.00 0.00 C

ATOM 1067 HA VAL 68 40.146 39.330 40.313 1.00 0.00 H

ATOM 1068 CB VAL 68 40.235 40.067 42.337 1.00 0.00 C

ATOM 1069 HB VAL 68 40.957 40.129 43.149 1.00 0.00 H

ATOM 1070 CG1 VAL 68 39.803 41.486 41.997 1.00 0.00 C

ATOM 1071 HG11 VAL 68 39.271 41.923 42.842 1.00 0.00 H

ATOM 1072 HG12 VAL 68 40.679 42.100 41.794 1.00 0.00 H

ATOM 1073 HG13 VAL 68 39.150 41.484 41.124 1.00 0.00 H

ATOM 1074 CG2 VAL 68 39.050 39.243 42.815 1.00 0.00 C

ATOM 1075 HG21 VAL 68 38.556 39.754 43.641 1.00 0.00 H

ATOM 1076 HG22 VAL 68 38.335 39.113 42.001 1.00 0.00 H

ATOM 1077 HG23 VAL 68 39.381 38.263 43.157 1.00 0.00 H

ATOM 1078 C VAL 68 42.083 40.204 40.630 1.00 0.00 C

```
ATOM   1079  O   VAL    68      43.084  40.360  41.290  1.00  0.00           O
ATOM   1080  N   ARG    69      41.962  40.912  39.509  1.00  0.00           N
ATOM   1081  H   ARG    69      41.102  40.811  38.995  1.00  0.00           H
ATOM   1082  CA  ARG    69      42.946  41.874  38.951  1.00  0.00           C
ATOM   1083  HA  ARG    69      43.826  41.843  39.586  1.00  0.00           H
ATOM   1084  CB  ARG    69      43.375  41.384  37.576  1.00  0.00           C
ATOM   1085  HB2 ARG    69      42.488  41.287  36.950  1.00  0.00           H
ATOM   1086  HB3 ARG    69      44.027  42.132  37.120  1.00  0.00           H
ATOM   1087  CG  ARG    69      44.125  40.062  37.573  1.00  0.00           C
ATOM   1088  HG2 ARG    69      45.037  40.168  38.163  1.00  0.00           H
ATOM   1089  HG3 ARG    69      43.503  39.291  38.030  1.00  0.00           H
ATOM   1090  CD  ARG    69      44.473  39.630  36.195  1.00  0.00           C
ATOM   1091  HD2 ARG    69      43.555  39.561  35.610  1.00  0.00           H
ATOM   1092  HD3 ARG    69      45.114  40.376  35.724  1.00  0.00           H
ATOM   1093  NE  ARG    69      45.110  38.323  36.180  1.00  0.00           N
ATOM   1094  HE  ARG    69      44.516  37.507  36.085  1.00  0.00           H
ATOM   1095  CZ  ARG    69      46.436  38.117  36.294  1.00  0.00           C
ATOM   1096  NH1 ARG    69      47.251  39.140  36.430  1.00  0.00           N
ATOM   1097 HH11 ARG    69      48.243  38.980  36.513  1.00  0.00           H
ATOM   1098 HH12 ARG    69      46.873  40.071  36.493  1.00  0.00           H
ATOM   1099  NH2 ARG    69      46.919  36.887  36.269  1.00  0.00           N
ATOM   1100 HH21 ARG    69      47.904  36.713  36.374  1.00  0.00           H
ATOM   1101 HH22 ARG    69      46.276  36.107  36.164  1.00  0.00           H
ATOM   1102  C   ARG    69      42.574  43.341  38.794  1.00  0.00           C
ATOM   1103  O   ARG    69      41.412  43.674  38.700  1.00  0.00           O
ATOM   1104  N   PHE    70      43.554  44.201  38.996  1.00  0.00           N
ATOM   1105  H   PHE    70      44.495  43.876  39.171  1.00  0.00           H
ATOM   1106  CA  PHE    70      43.304  45.618  38.999  1.00  0.00           C
ATOM   1107  HA  PHE    70      42.488  45.844  38.310  1.00  0.00           H
ATOM   1108  CB  PHE    70      42.857  46.034  40.401  1.00  0.00           C
```

ATOM 1109 HB2 PHE 70 42.739 47.117 40.454 1.00 0.00 H
ATOM 1110 HB3 PHE 70 41.880 45.600 40.583 1.00 0.00 H
ATOM 1111 CG PHE 70 43.773 45.564 41.494 1.00 0.00 C
ATOM 1112 CD1 PHE 70 44.768 46.393 41.990 1.00 0.00 C
ATOM 1113 HD1 PHE 70 44.887 47.392 41.596 1.00 0.00 H
ATOM 1114 CE1 PHE 70 45.612 45.961 42.996 1.00 0.00 C
ATOM 1115 HE1 PHE 70 46.360 46.629 43.398 1.00 0.00 H
ATOM 1116 CZ PHE 70 45.470 44.694 43.519 1.00 0.00 C
ATOM 1117 HZ PHE 70 46.116 44.351 44.310 1.00 0.00 H
ATOM 1118 CE2 PHE 70 44.484 43.857 43.035 1.00 0.00 C
ATOM 1119 HE2 PHE 70 44.367 42.867 43.441 1.00 0.00 H
ATOM 1120 CD2 PHE 70 43.643 44.291 42.029 1.00 0.00 C
ATOM 1121 HD2 PHE 70 42.870 43.626 41.673 1.00 0.00 H
ATOM 1122 C PHE 70 44.493 46.494 38.564 1.00 0.00 C
ATOM 1123 O PHE 70 45.627 46.000 38.481 1.00 0.00 O
ATOM 1124 N ILE 71 44.263 47.812 38.302 1.00 0.00 N
ATOM 1125 H ILE 71 43.318 48.122 38.478 1.00 0.00 H
ATOM 1126 CA ILE 71 45.110 48.849 37.740 1.00 0.00 C
ATOM 1127 HA ILE 71 45.464 48.493 36.777 1.00 0.00 H
ATOM 1128 CB ILE 71 44.311 50.151 37.548 1.00 0.00 C
ATOM 1129 HB ILE 71 43.904 50.459 38.512 1.00 0.00 H
ATOM 1130 CG2 ILE 71 45.219 51.263 37.045 1.00 0.00 C
ATOM 1131 HG21 ILE 71 44.625 52.151 36.827 1.00 0.00 H
ATOM 1132 HG22 ILE 71 45.957 51.533 37.795 1.00 0.00 H
ATOM 1133 HG23 ILE 71 45.746 50.969 36.141 1.00 0.00 H
ATOM 1134 CG1 ILE 71 43.148 49.925 36.578 1.00 0.00 C
ATOM 1135 HG12 ILE 71 42.449 49.198 36.992 1.00 0.00 H
ATOM 1136 HG13 ILE 71 42.603 50.863 36.467 1.00 0.00 H
ATOM 1137 CD1 ILE 71 43.579 49.467 35.203 1.00 0.00 C
ATOM 1138 HD11 ILE 71 42.696 49.409 34.565 1.00 0.00 H

```
ATOM   1139 HD12 ILE    71      44.268  50.173  34.742  1.00  0.00           H
ATOM   1140 HD13 ILE    71      44.026  48.475  35.236  1.00  0.00           H
ATOM   1141  C   ILE    71      46.332  49.126  38.615  1.00  0.00           C
ATOM   1142  O   ILE    71      46.051  49.211  39.814  1.00  0.00           O
ATOM   1143  N   PRO    72      47.598  49.124  38.159  1.00  0.00           N
ATOM   1144  CD  PRO    72      47.969  48.931  36.725  1.00  0.00           C
ATOM   1145  HD2 PRO    72      47.391  49.565  36.056  1.00  0.00           H
ATOM   1146  HD3 PRO    72      47.850  47.880  36.458  1.00  0.00           H
ATOM   1147  CG  PRO    72      49.424  49.314  36.680  1.00  0.00           C
ATOM   1148  HG2 PRO    72      49.514  50.387  36.504  1.00  0.00           H
ATOM   1149  HG3 PRO    72      49.955  48.763  35.902  1.00  0.00           H
ATOM   1150  CB  PRO    72      49.940  48.973  38.036  1.00  0.00           C
ATOM   1151  HB2 PRO    72      50.871  49.494  38.255  1.00  0.00           H
ATOM   1152  HB3 PRO    72      50.101  47.898  38.075  1.00  0.00           H
ATOM   1153  CA  PRO    72      48.799  49.363  38.981  1.00  0.00           C
ATOM   1154  HA  PRO    72      48.799  48.719  39.861  1.00  0.00           H
ATOM   1155  C   PRO    72      48.892  50.856  39.418  1.00  0.00           C
ATOM   1156  O   PRO    72      49.640  51.683  38.846  1.00  0.00           O
ATOM   1157  N   LEU    73      48.053  51.209  40.342  1.00  0.00           N
ATOM   1158  H   LEU    73      47.459  50.495  40.742  1.00  0.00           H
ATOM   1159  CA  LEU    73      47.859  52.561  40.832  1.00  0.00           C
ATOM   1160  HA  LEU    73      48.548  53.228  40.316  1.00  0.00           H
ATOM   1161  CB  LEU    73      46.425  52.995  40.503  1.00  0.00           C
ATOM   1162  HB2 LEU    73      46.289  52.921  39.427  1.00  0.00           H
ATOM   1163  HB3 LEU    73      45.729  52.293  40.967  1.00  0.00           H
ATOM   1164  CG  LEU    73      46.049  54.422  40.923  1.00  0.00           C
ATOM   1165  HG  LEU    73      46.187  54.590  41.987  1.00  0.00           H
ATOM   1166  CD1 LEU    73      46.926  55.418  40.177  1.00  0.00           C
ATOM   1167 HD11 LEU    73      46.595  56.433  40.397  1.00  0.00           H
ATOM   1168 HD12 LEU    73      47.962  55.333  40.505  1.00  0.00           H
```

```
ATOM   1169 HD13 LEU    73      46.867  55.248  39.101  1.00  0.00           H
ATOM   1170  CD2 LEU    73      44.575  54.665  40.633  1.00  0.00           C
ATOM   1171 HD21 LEU    73      44.307  55.682  40.922  1.00  0.00           H
ATOM   1172 HD22 LEU    73      44.372  54.528  39.570  1.00  0.00           H
ATOM   1173 HD23 LEU    73      43.969  53.969  41.213  1.00  0.00           H
ATOM   1174  C   LEU    73      48.138  52.715  42.389  1.00  0.00           C
ATOM   1175  O   LEU    73      47.165  52.719  43.078  1.00  0.00           O
ATOM   1176  N   ASP    74      49.372  52.721  42.923  1.00  0.00           N
ATOM   1177  H   ASP    74      50.148  52.639  42.291  1.00  0.00           H
ATOM   1178  CA  ASP    74      49.724  52.942  44.337  1.00  0.00           C
ATOM   1179  HA  ASP    74      48.824  52.913  44.952  1.00  0.00           H
ATOM   1180  CB  ASP    74      50.687  51.864  44.840  1.00  0.00           C
ATOM   1181  HB2 ASP    74      51.654  51.966  44.354  1.00  0.00           H
ATOM   1182  HB3 ASP    74      50.838  52.009  45.910  1.00  0.00           H
ATOM   1183  CG  ASP    74      50.154  50.452  44.637  1.00  0.00           C
ATOM   1184  OD1 ASP    74      48.964  50.302  44.489  1.00  0.00           O
ATOM   1185  OD2 ASP    74      50.942  49.536  44.631  1.00  0.00           O
ATOM   1186  C   ASP    74      50.366  54.362  44.458  1.00  0.00           C
ATOM   1187  O   ASP    74      50.715  54.951  43.448  1.00  0.00           O
ATOM   1188  N   ASP    75      50.392  54.926  45.638  1.00  0.00           N
ATOM   1189  H   ASP    75      50.050  54.428  46.448  1.00  0.00           H
ATOM   1190  CA  ASP    75      50.863  56.355  45.817  1.00  0.00           C
ATOM   1191  HA  ASP    75      50.722  56.897  44.882  1.00  0.00           H
ATOM   1192  CB  ASP    75      49.994  57.048  46.869  1.00  0.00           C
ATOM   1193  HB2 ASP    75      50.032  56.462  47.782  1.00  0.00           H
ATOM   1194  HB3 ASP    75      50.397  58.040  47.078  1.00  0.00           H
ATOM   1195  CG  ASP    75      48.546  57.211  46.427  1.00  0.00           C
ATOM   1196  OD1 ASP    75      48.325  57.460  45.265  1.00  0.00           O
ATOM   1197  OD2 ASP    75      47.675  57.086  47.254  1.00  0.00           O
ATOM   1198  C   ASP    75      52.348  56.564  46.220  1.00  0.00           C
```

ATOM 1199 O ASP 75 52.967 57.596 45.919 1.00 0.00 O

ATOM 1200 N PRO 76 53.090 55.627 46.875 1.00 0.00 N

ATOM 1201 CD PRO 76 52.558 54.330 47.390 1.00 0.00 C

ATOM 1202 HD2 PRO 76 52.164 53.690 46.607 1.00 0.00 H

ATOM 1203 HD3 PRO 76 51.841 54.532 48.178 1.00 0.00 H

ATOM 1204 CG PRO 76 53.771 53.645 47.960 1.00 0.00 C

ATOM 1205 HG2 PRO 76 54.273 53.102 47.161 1.00 0.00 H

ATOM 1206 HG3 PRO 76 53.503 52.964 48.766 1.00 0.00 H

ATOM 1207 CB PRO 76 54.635 54.760 48.441 1.00 0.00 C

ATOM 1208 HB2 PRO 76 55.677 54.468 48.528 1.00 0.00 H

ATOM 1209 HB3 PRO 76 54.284 55.113 49.403 1.00 0.00 H

ATOM 1210 CA PRO 76 54.484 55.842 47.367 1.00 0.00 C

ATOM 1211 HA PRO 76 54.575 56.824 47.832 1.00 0.00 H

ATOM 1212 C PRO 76 55.555 55.687 46.300 1.00 0.00 C

ATOM 1213 O PRO 76 55.340 55.118 45.268 1.00 0.00 O

ATOM 1214 N THR 77 56.743 56.182 46.522 1.00 0.00 N

ATOM 1215 H THR 77 56.867 56.718 47.368 1.00 0.00 H

ATOM 1216 CA THR 77 57.980 55.816 45.820 1.00 0.00 C

ATOM 1217 HA THR 77 57.922 54.767 45.523 1.00 0.00 H

ATOM 1218 CB THR 77 58.168 56.679 44.559 1.00 0.00 C

ATOM 1219 HB THR 77 58.259 57.728 44.843 1.00 0.00 H

ATOM 1220 CG2 THR 77 59.422 56.259 43.807 1.00 0.00 C

ATOM 1221 HG21 THR 77 59.468 56.780 42.850 1.00 0.00 H

ATOM 1222 HG22 THR 77 60.312 56.521 44.378 1.00 0.00 H

ATOM 1223 HG23 THR 77 59.411 55.183 43.625 1.00 0.00 H

ATOM 1224 OG1 THR 77 57.029 56.532 43.701 1.00 0.00 O

ATOM 1225 HG1 THR 77 56.766 55.608 43.710 1.00 0.00 H

ATOM 1226 C THR 77 59.216 55.985 46.831 1.00 0.00 C

ATOM 1227 O THR 77 59.250 56.793 47.736 1.00 0.00 O

ATOM 1228 N ASN 78 60.188 55.004 46.781 1.00 0.00 N

ATOM 1229 H ASN 78 60.111 54.256 46.106 1.00 0.00 H

ATOM 1230 CA ASN 78 61.392 55.088 47.588 1.00 0.00 C

ATOM 1231 HA ASN 78 61.964 54.197 47.332 1.00 0.00 H

ATOM 1232 CB ASN 78 62.176 56.324 47.187 1.00 0.00 C

ATOM 1233 HB2 ASN 78 62.197 56.381 46.098 1.00 0.00 H

ATOM 1234 HB3 ASN 78 61.710 57.237 47.559 1.00 0.00 H

ATOM 1235 CG ASN 78 63.608 56.274 47.642 1.00 0.00 C

ATOM 1236 OD1 ASN 78 64.168 57.419 47.935 1.00 0.00 O

ATOM 1237 ND2 ASN 78 64.207 55.195 47.728 1.00 0.00 N

ATOM 1238 HD21 ASN 78 63.722 54.354 47.453 1.00 0.00 H

ATOM 1239 HD22 ASN 78 65.178 55.165 47.998 1.00 0.00 H

ATOM 1240 C ASN 78 61.197 55.090 49.084 1.00 0.00 C

ATOM 1241 O ASN 78 61.779 55.898 49.731 1.00 0.00 O

ATOM 1242 N LYS 79 60.442 54.126 49.549 1.00 0.00 N

ATOM 1243 H LYS 79 60.066 53.499 48.853 1.00 0.00 H

ATOM 1244 CA LYS 79 59.974 53.853 50.902 1.00 0.00 C

ATOM 1245 HA LYS 79 60.407 54.578 51.590 1.00 0.00 H

ATOM 1246 CB LYS 79 58.449 53.943 50.980 1.00 0.00 C

ATOM 1247 HB2 LYS 79 58.057 53.162 50.342 1.00 0.00 H

ATOM 1248 HB3 LYS 79 58.133 53.740 52.003 1.00 0.00 H

ATOM 1249 CG LYS 79 57.874 55.279 50.529 1.00 0.00 C

ATOM 1250 HG2 LYS 79 58.251 55.525 49.544 1.00 0.00 H

ATOM 1251 HG3 LYS 79 56.797 55.216 50.421 1.00 0.00 H

ATOM 1252 CD LYS 79 58.224 56.390 51.507 1.00 0.00 C

ATOM 1253 HD2 LYS 79 57.895 56.107 52.508 1.00 0.00 H

ATOM 1254 HD3 LYS 79 59.304 56.541 51.515 1.00 0.00 H

ATOM 1255 CE LYS 79 57.553 57.700 51.122 1.00 0.00 C

ATOM 1256 HE2 LYS 79 57.846 57.968 50.105 1.00 0.00 H

ATOM 1257 HE3 LYS 79 56.471 57.555 51.144 1.00 0.00 H

ATOM 1258 NZ LYS 79 57.914 58.805 52.052 1.00 0.00 N

ATOM 1259 HZ1 LYS 79 57.727 58.520 53.002 1.00 0.00 H

ATOM 1260 HZ2 LYS 79 58.892 59.033 51.947 1.00 0.00 H

ATOM 1261 HZ3 LYS 79 57.349 59.614 51.835 1.00 0.00 H

ATOM 1262 C LYS 79 60.471 52.450 51.324 1.00 0.00 C

ATOM 1263 O LYS 79 61.504 52.030 50.730 1.00 0.00 O

ATOM 1264 N PHE 80 60.032 51.923 52.473 1.00 0.00 N

ATOM 1265 H PHE 80 59.272 52.404 52.931 1.00 0.00 H

ATOM 1266 CA PHE 80 60.427 50.633 53.091 1.00 0.00 C

ATOM 1267 HA PHE 80 61.462 50.737 53.411 1.00 0.00 H

ATOM 1268 CB PHE 80 59.568 50.356 54.327 1.00 0.00 C

ATOM 1269 HB2 PHE 80 59.505 51.253 54.945 1.00 0.00 H

ATOM 1270 HB3 PHE 80 58.577 50.085 53.996 1.00 0.00 H

ATOM 1271 CG PHE 80 60.064 49.213 55.166 1.00 0.00 C

ATOM 1272 CD1 PHE 80 61.087 49.400 56.083 1.00 0.00 C

ATOM 1273 HD1 PHE 80 61.513 50.383 56.220 1.00 0.00 H

ATOM 1274 CE1 PHE 80 61.545 48.349 56.856 1.00 0.00 C

ATOM 1275 HE1 PHE 80 62.361 48.501 57.547 1.00 0.00 H

ATOM 1276 CZ PHE 80 60.983 47.097 56.719 1.00 0.00 C

ATOM 1277 HZ PHE 80 61.332 46.281 57.328 1.00 0.00 H

ATOM 1278 CE2 PHE 80 59.964 46.896 55.809 1.00 0.00 C

ATOM 1279 HE2 PHE 80 59.517 45.918 55.703 1.00 0.00 H

ATOM 1280 CD2 PHE 80 59.510 47.948 55.038 1.00 0.00 C

ATOM 1281 HD2 PHE 80 58.702 47.785 54.343 1.00 0.00 H

ATOM 1282 C PHE 80 60.307 49.446 52.110 1.00 0.00 C

ATOM 1283 O PHE 80 59.392 49.449 51.281 1.00 0.00 O

ATOM 1284 N PRO 81 61.216 48.392 52.131 1.00 0.00 N

ATOM 1285 CD PRO 81 62.398 48.278 52.991 1.00 0.00 C

ATOM 1286 HD2 PRO 81 62.116 47.907 53.974 1.00 0.00 H

ATOM 1287 HD3 PRO 81 62.951 49.214 53.062 1.00 0.00 H

ATOM 1288 CG PRO 81 63.211 47.235 52.272 1.00 0.00 C

ATOM 1289 HG2 PRO 81 63.829 46.674 52.972 1.00 0.00 H
ATOM 1290 HG3 PRO 81 63.825 47.717 51.510 1.00 0.00 H
ATOM 1291 CB PRO 81 62.197 46.361 51.616 1.00 0.00 C
ATOM 1292 HB2 PRO 81 61.813 45.653 52.352 1.00 0.00 H
ATOM 1293 HB3 PRO 81 62.627 45.834 50.766 1.00 0.00 H
ATOM 1294 CA PRO 81 61.092 47.329 51.181 1.00 0.00 C
ATOM 1295 HA PRO 81 61.310 47.721 50.193 1.00 0.00 H
ATOM 1296 C PRO 81 59.752 46.673 51.219 1.00 0.00 C
ATOM 1297 O PRO 81 59.067 46.661 52.172 1.00 0.00 O
ATOM 1298 N SER 82 59.470 46.089 50.049 1.00 0.00 N
ATOM 1299 H SER 82 60.168 46.118 49.319 1.00 0.00 H
ATOM 1300 CA SER 82 58.164 45.519 49.688 1.00 0.00 C
ATOM 1301 HA SER 82 58.345 44.657 49.045 1.00 0.00 H
ATOM 1302 CB SER 82 57.477 45.023 50.945 1.00 0.00 C
ATOM 1303 HB2 SER 82 56.662 44.368 50.648 1.00 0.00 H
ATOM 1304 HB3 SER 82 58.156 44.434 51.560 1.00 0.00 H
ATOM 1305 OG SER 82 56.937 46.090 51.676 1.00 0.00 O
ATOM 1306 HG SER 82 57.683 46.551 52.085 1.00 0.00 H
ATOM 1307 C SER 82 57.217 46.471 48.951 1.00 0.00 C
ATOM 1308 O SER 82 57.542 47.614 48.705 1.00 0.00 O
ATOM 1309 N LEU 83 56.080 45.964 48.501 1.00 0.00 N
ATOM 1310 H LEU 83 55.893 44.999 48.731 1.00 0.00 H
ATOM 1311 CA LEU 83 55.009 46.625 47.678 1.00 0.00 C
ATOM 1312 HA LEU 83 55.027 47.699 47.857 1.00 0.00 H
ATOM 1313 CB LEU 83 55.181 46.358 46.177 1.00 0.00 C
ATOM 1314 HB2 LEU 83 56.154 46.743 45.866 1.00 0.00 H
ATOM 1315 HB3 LEU 83 55.202 45.276 46.034 1.00 0.00 H
ATOM 1316 CG LEU 83 54.094 46.945 45.268 1.00 0.00 C
ATOM 1317 HG LEU 83 53.107 46.659 45.632 1.00 0.00 H
ATOM 1318 CD1 LEU 83 54.200 48.464 45.264 1.00 0.00 C

ATOM 1319 HD11 LEU 83 53.474 48.885 44.569 1.00 0.00 H
ATOM 1320 HD12 LEU 83 53.990 48.864 46.256 1.00 0.00 H
ATOM 1321 HD13 LEU 83 55.200 48.776 44.958 1.00 0.00 H
ATOM 1322 CD2 LEU 83 54.249 46.382 43.863 1.00 0.00 C
ATOM 1323 HD21 LEU 83 53.508 46.819 43.204 1.00 0.00 H
ATOM 1324 HD22 LEU 83 55.242 46.612 43.475 1.00 0.00 H
ATOM 1325 HD23 LEU 83 54.096 45.303 43.886 1.00 0.00 H
ATOM 1326 C LEU 83 53.679 46.020 48.204 1.00 0.00 C
ATOM 1327 O LEU 83 53.604 44.764 48.289 1.00 0.00 O
ATOM 1328 N CYS 84 52.689 46.904 48.424 1.00 0.00 N
ATOM 1329 H CYS 84 52.934 47.879 48.327 1.00 0.00 H
ATOM 1330 CA CYS 84 51.264 46.663 48.476 1.00 0.00 C
ATOM 1331 HA CYS 84 51.000 45.978 47.671 1.00 0.00 H
ATOM 1332 CB CYS 84 50.876 46.029 49.812 1.00 0.00 C
ATOM 1333 HB2 CYS 84 49.807 45.815 49.803 1.00 0.00 H
ATOM 1334 HB3 CYS 84 51.405 45.082 49.919 1.00 0.00 H
ATOM 1335 SG CYS 84 51.241 47.061 51.252 1.00 0.00 S
ATOM 1336 HG CYS 84 52.569 47.000 51.133 1.00 0.00 H
ATOM 1337 C CYS 84 50.497 47.979 48.279 1.00 0.00 C
ATOM 1338 O CYS 84 51.101 49.048 48.376 1.00 0.00 O
ATOM 1339 N THR 85 49.176 47.961 48.070 1.00 0.00 N
ATOM 1340 H THR 85 48.667 47.093 48.004 1.00 0.00 H
ATOM 1341 CA THR 85 48.484 49.225 47.770 1.00 0.00 C
ATOM 1342 HA THR 85 49.106 49.809 47.089 1.00 0.00 H
ATOM 1343 CB THR 85 47.158 48.946 47.039 1.00 0.00 C
ATOM 1344 HB THR 85 46.533 48.297 47.654 1.00 0.00 H
ATOM 1345 CG2 THR 85 46.412 50.244 46.774 1.00 0.00 C
ATOM 1346 HG21 THR 85 46.136 50.758 47.688 1.00 0.00 H
ATOM 1347 HG22 THR 85 47.030 50.916 46.178 1.00 0.00 H
ATOM 1348 HG23 THR 85 45.499 50.026 46.220 1.00 0.00 H

| | | | | | | | | | | |
|---|---|---|---|---|---|---|---|---|---|---|
| ATOM | 1349 | OG1 | THR | 85 | 47.426 | 48.290 | 45.793 | 1.00 | 0.00 | O |
| ATOM | 1350 | HG1 | THR | 85 | 47.945 | 48.891 | 45.243 | 1.00 | 0.00 | H |
| ATOM | 1351 | C | THR | 85 | 48.198 | 50.094 | 49.006 | 1.00 | 0.00 | C |
| ATOM | 1352 | O | THR | 85 | 47.337 | 49.826 | 49.839 | 1.00 | 0.00 | O |
| ATOM | 1353 | N | VAL | 86 | 49.137 | 51.024 | 49.315 | 1.00 | 0.00 | N |
| ATOM | 1354 | H | VAL | 86 | 49.889 | 51.088 | 48.642 | 1.00 | 0.00 | H |
| ATOM | 1355 | CA | VAL | 86 | 49.270 | 52.006 | 50.409 | 1.00 | 0.00 | C |
| ATOM | 1356 | HA | VAL | 86 | 48.315 | 52.074 | 50.931 | 1.00 | 0.00 | H |
| ATOM | 1357 | CB | VAL | 86 | 50.342 | 51.552 | 51.417 | 1.00 | 0.00 | C |
| ATOM | 1358 | HB | VAL | 86 | 50.336 | 52.220 | 52.273 | 1.00 | 0.00 | H |
| ATOM | 1359 | CG1 | VAL | 86 | 50.012 | 50.169 | 51.958 | 1.00 | 0.00 | C |
| ATOM | 1360 | HG11 | VAL | 86 | 50.728 | 49.885 | 52.730 | 1.00 | 0.00 | H |
| ATOM | 1361 | HG12 | VAL | 86 | 49.009 | 50.157 | 52.373 | 1.00 | 0.00 | H |
| ATOM | 1362 | HG13 | VAL | 86 | 50.050 | 49.445 | 51.151 | 1.00 | 0.00 | H |
| ATOM | 1363 | CG2 | VAL | 86 | 51.712 | 51.559 | 50.755 | 1.00 | 0.00 | C |
| ATOM | 1364 | HG21 | VAL | 86 | 52.425 | 51.051 | 51.398 | 1.00 | 0.00 | H |
| ATOM | 1365 | HG22 | VAL | 86 | 51.696 | 51.051 | 49.795 | 1.00 | 0.00 | H |
| ATOM | 1366 | HG23 | VAL | 86 | 52.066 | 52.580 | 50.612 | 1.00 | 0.00 | H |
| ATOM | 1367 | C | VAL | 86 | 49.634 | 53.366 | 49.937 | 1.00 | 0.00 | C |
| ATOM | 1368 | O | VAL | 86 | 49.787 | 53.578 | 48.745 | 1.00 | 0.00 | O |
| ATOM | 1369 | N | ARG | 87 | 49.736 | 54.310 | 50.867 | 1.00 | 0.00 | N |
| ATOM | 1370 | H | ARG | 87 | 49.476 | 54.042 | 51.804 | 1.00 | 0.00 | H |
| ATOM | 1371 | CA | ARG | 87 | 50.301 | 55.676 | 50.738 | 1.00 | 0.00 | C |
| ATOM | 1372 | HA | ARG | 87 | 49.956 | 56.068 | 49.786 | 1.00 | 0.00 | H |
| ATOM | 1373 | CB | ARG | 87 | 49.669 | 56.545 | 51.815 | 1.00 | 0.00 | C |
| ATOM | 1374 | HB2 | ARG | 87 | 48.588 | 56.466 | 51.731 | 1.00 | 0.00 | H |
| ATOM | 1375 | HB3 | ARG | 87 | 49.953 | 56.171 | 52.800 | 1.00 | 0.00 | H |
| ATOM | 1376 | CG | ARG | 87 | 49.994 | 58.027 | 51.714 | 1.00 | 0.00 | C |
| ATOM | 1377 | HG2 | ARG | 87 | 51.069 | 58.204 | 51.755 | 1.00 | 0.00 | H |
| ATOM | 1378 | HG3 | ARG | 87 | 49.626 | 58.396 | 50.755 | 1.00 | 0.00 | H |

| | | | | | | | | | | |
|---|---|---|---|---|---|---|---|---|---|---|
| ATOM | 1379 | CD | ARG | 87 | 49.347 | 58.807 | 52.800 | 1.00 | 0.00 | C |
| ATOM | 1380 | HD2 | ARG | 87 | 49.412 | 59.866 | 52.545 | 1.00 | 0.00 | H |
| ATOM | 1381 | HD3 | ARG | 87 | 48.293 | 58.531 | 52.849 | 1.00 | 0.00 | H |
| ATOM | 1382 | NE | ARG | 87 | 50.008 | 58.605 | 54.080 | 1.00 | 0.00 | N |
| ATOM | 1383 | HE | ARG | 87 | 51.001 | 58.415 | 54.056 | 1.00 | 0.00 | H |
| ATOM | 1384 | CZ | ARG | 87 | 49.403 | 58.715 | 55.279 | 1.00 | 0.00 | C |
| ATOM | 1385 | NH1 | ARG | 87 | 48.127 | 59.025 | 55.345 | 1.00 | 0.00 | N |
| ATOM | 1386 | HH11 | ARG | 87 | 47.668 | 59.116 | 56.237 | 1.00 | 0.00 | H |
| ATOM | 1387 | HH12 | ARG | 87 | 47.619 | 59.214 | 54.493 | 1.00 | 0.00 | H |
| ATOM | 1388 | NH2 | ARG | 87 | 50.092 | 58.512 | 56.388 | 1.00 | 0.00 | N |
| ATOM | 1389 | HH21 | ARG | 87 | 51.072 | 58.276 | 56.322 | 1.00 | 0.00 | H |
| ATOM | 1390 | HH22 | ARG | 87 | 49.655 | 58.630 | 57.288 | 1.00 | 0.00 | H |
| ATOM | 1391 | C | ARG | 87 | 51.812 | 55.933 | 50.805 | 1.00 | 0.00 | C |
| ATOM | 1392 | O | ARG | 87 | 52.371 | 56.533 | 49.895 | 1.00 | 0.00 | O |
| ATOM | 1393 | N | ASP | 88 | 52.416 | 55.579 | 51.932 | 1.00 | 0.00 | N |
| ATOM | 1394 | H | ASP | 88 | 51.829 | 55.192 | 52.651 | 1.00 | 0.00 | H |
| ATOM | 1395 | CA | ASP | 88 | 53.861 | 55.665 | 52.250 | 1.00 | 0.00 | C |
| ATOM | 1396 | HA | ASP | 88 | 54.384 | 56.095 | 51.396 | 1.00 | 0.00 | H |
| ATOM | 1397 | CB | ASP | 88 | 54.088 | 56.597 | 53.443 | 1.00 | 0.00 | C |
| ATOM | 1398 | HB2 | ASP | 88 | 53.586 | 56.230 | 54.336 | 1.00 | 0.00 | H |
| ATOM | 1399 | HB3 | ASP | 88 | 55.157 | 56.621 | 53.663 | 1.00 | 0.00 | H |
| ATOM | 1400 | CG | ASP | 88 | 53.642 | 58.027 | 53.170 | 1.00 | 0.00 | C |
| ATOM | 1401 | OD1 | ASP | 88 | 54.094 | 58.596 | 52.205 | 1.00 | 0.00 | O |
| ATOM | 1402 | OD2 | ASP | 88 | 52.854 | 58.537 | 53.930 | 1.00 | 0.00 | O |
| ATOM | 1403 | C | ASP | 88 | 54.500 | 54.329 | 52.558 | 1.00 | 0.00 | C |
| ATOM | 1404 | O | ASP | 88 | 55.691 | 54.187 | 52.222 | 1.00 | 0.00 | O |
| ATOM | 1405 | N | PRO | 89 | 53.870 | 53.418 | 53.335 | 1.00 | 0.00 | N |
| ATOM | 1406 | CD | PRO | 89 | 52.494 | 53.653 | 53.782 | 1.00 | 0.00 | C |
| ATOM | 1407 | HD2 | PRO | 89 | 51.794 | 53.472 | 52.974 | 1.00 | 0.00 | H |
| ATOM | 1408 | HD3 | PRO | 89 | 52.327 | 54.608 | 54.273 | 1.00 | 0.00 | H |

| | | | | | | | | | | |
|---|---|---|---|---|---|---|---|---|---|---|
| ATOM | 1409 | CG | PRO | 89 | 52.366 | 52.557 | 54.806 | 1.00 | 0.00 | C |
| ATOM | 1410 | HG2 | PRO | 89 | 51.362 | 52.163 | 54.828 | 1.00 | 0.00 | H |
| ATOM | 1411 | HG3 | PRO | 89 | 52.638 | 52.944 | 55.790 | 1.00 | 0.00 | H |
| ATOM | 1412 | CB | PRO | 89 | 53.364 | 51.535 | 54.382 | 1.00 | 0.00 | C |
| ATOM | 1413 | HB2 | PRO | 89 | 52.955 | 50.933 | 53.571 | 1.00 | 0.00 | H |
| ATOM | 1414 | HB3 | PRO | 89 | 53.642 | 50.887 | 55.214 | 1.00 | 0.00 | H |
| ATOM | 1415 | CA | PRO | 89 | 54.563 | 52.358 | 53.898 | 1.00 | 0.00 | C |
| ATOM | 1416 | HA | PRO | 89 | 55.113 | 52.727 | 54.765 | 1.00 | 0.00 | H |
| ATOM | 1417 | C | PRO | 89 | 55.537 | 51.499 | 53.012 | 1.00 | 0.00 | C |
| ATOM | 1418 | O | PRO | 89 | 56.584 | 51.089 | 53.486 | 1.00 | 0.00 | O |
| ATOM | 1419 | N | THR | 90 | 55.246 | 51.254 | 51.706 | 1.00 | 0.00 | N |
| ATOM | 1420 | H | THR | 90 | 54.414 | 51.690 | 51.337 | 1.00 | 0.00 | H |
| ATOM | 1421 | CA | THR | 90 | 55.936 | 50.355 | 50.756 | 1.00 | 0.00 | C |
| ATOM | 1422 | HA | THR | 90 | 56.831 | 49.959 | 51.235 | 1.00 | 0.00 | H |
| ATOM | 1423 | CB | THR | 90 | 55.041 | 49.159 | 50.384 | 1.00 | 0.00 | C |
| ATOM | 1424 | HB | THR | 90 | 55.610 | 48.432 | 49.823 | 1.00 | 0.00 | H |
| ATOM | 1425 | CG2 | THR | 90 | 54.515 | 48.476 | 51.637 | 1.00 | 0.00 | C |
| ATOM | 1426 | HG21 | THR | 90 | 54.166 | 47.484 | 51.359 | 1.00 | 0.00 | H |
| ATOM | 1427 | HG22 | THR | 90 | 55.322 | 48.364 | 52.363 | 1.00 | 0.00 | H |
| ATOM | 1428 | HG23 | THR | 90 | 53.698 | 49.043 | 52.085 | 1.00 | 0.00 | H |
| ATOM | 1429 | OG1 | THR | 90 | 53.935 | 49.614 | 49.593 | 1.00 | 0.00 | O |
| ATOM | 1430 | HG1 | THR | 90 | 53.230 | 48.965 | 49.646 | 1.00 | 0.00 | H |
| ATOM | 1431 | C | THR | 90 | 56.373 | 51.060 | 49.476 | 1.00 | 0.00 | C |
| ATOM | 1432 | O | THR | 90 | 56.119 | 52.255 | 49.329 | 1.00 | 0.00 | O |
| ATOM | 1433 | N | ASP | 91 | 56.967 | 50.299 | 48.605 | 1.00 | 0.00 | N |
| ATOM | 1434 | H | ASP | 91 | 57.097 | 49.328 | 48.837 | 1.00 | 0.00 | H |
| ATOM | 1435 | CA | ASP | 91 | 57.613 | 50.783 | 47.364 | 1.00 | 0.00 | C |
| ATOM | 1436 | HA | ASP | 91 | 57.748 | 49.932 | 46.702 | 1.00 | 0.00 | H |
| ATOM | 1437 | CB | ASP | 91 | 56.659 | 51.711 | 46.609 | 1.00 | 0.00 | C |
| ATOM | 1438 | HB2 | ASP | 91 | 55.673 | 51.244 | 46.568 | 1.00 | 0.00 | H |

ATOM 1439 HB3 ASP 91 56.570 52.686 47.081 1.00 0.00 H
ATOM 1440 CG ASP 91 57.111 51.994 45.183 1.00 0.00 C
ATOM 1441 OD1 ASP 91 58.277 51.834 44.907 1.00 0.00 O
ATOM 1442 OD2 ASP 91 56.287 52.367 44.382 1.00 0.00 O
ATOM 1443 C ASP 91 58.949 51.523 47.611 1.00 0.00 C
ATOM 1444 O ASP 91 59.018 52.740 47.566 1.00 0.00 O
ATOM 1445 N PRO 92 60.045 50.712 47.551 1.00 0.00 N
ATOM 1446 CD PRO 92 59.914 49.225 47.654 1.00 0.00 C
ATOM 1447 HD2 PRO 92 59.353 48.773 46.837 1.00 0.00 H
ATOM 1448 HD3 PRO 92 59.515 48.966 48.632 1.00 0.00 H
ATOM 1449 CG PRO 92 61.353 48.790 47.573 1.00 0.00 C
ATOM 1450 HG2 PRO 92 61.617 48.631 46.526 1.00 0.00 H
ATOM 1451 HG3 PRO 92 61.542 47.875 48.130 1.00 0.00 H
ATOM 1452 CB PRO 92 62.123 49.954 48.095 1.00 0.00 C
ATOM 1453 HB2 PRO 92 63.174 49.902 47.811 1.00 0.00 H
ATOM 1454 HB3 PRO 92 62.053 49.959 49.183 1.00 0.00 H
ATOM 1455 CA PRO 92 61.386 51.174 47.532 1.00 0.00 C
ATOM 1456 HA PRO 92 61.499 52.011 48.216 1.00 0.00 H
ATOM 1457 C PRO 92 61.970 51.578 46.213 1.00 0.00 C
ATOM 1458 O PRO 92 63.160 51.507 45.937 1.00 0.00 O
ATOM 1459 N ASP 93 61.202 51.851 45.187 1.00 0.00 N
ATOM 1460 H ASP 93 60.204 51.828 45.355 1.00 0.00 H
ATOM 1461 CA ASP 93 61.572 52.232 43.829 1.00 0.00 C
ATOM 1462 HA ASP 93 60.638 52.419 43.300 1.00 0.00 H
ATOM 1463 CB ASP 93 62.382 53.529 43.885 1.00 0.00 C
ATOM 1464 HB2 ASP 93 61.916 54.201 44.607 1.00 0.00 H
ATOM 1465 HB3 ASP 93 63.403 53.346 44.224 1.00 0.00 H
ATOM 1466 CG ASP 93 62.411 54.268 42.553 1.00 0.00 C
ATOM 1467 OD1 ASP 93 61.485 54.116 41.792 1.00 0.00 O
ATOM 1468 OD2 ASP 93 63.358 54.977 42.310 1.00 0.00 O

| | | | | | | | | | | |
|---|---|---|---|---|---|---|---|---|---|---|
| ATOM | 1469 | C | ASP | 93 | 62.370 | 51.172 | 43.025 | 1.00 | 0.00 | C |
| ATOM | 1470 | O | ASP | 93 | 63.387 | 51.523 | 42.439 | 1.00 | 0.00 | O |
| ATOM | 1471 | N | SER | 94 | 62.048 | 49.927 | 42.953 | 1.00 | 0.00 | N |
| ATOM | 1472 | H | SER | 94 | 61.265 | 49.628 | 43.512 | 1.00 | 0.00 | H |
| ATOM | 1473 | CA | SER | 94 | 62.644 | 48.915 | 42.114 | 1.00 | 0.00 | C |
| ATOM | 1474 | HA | SER | 94 | 63.164 | 49.417 | 41.298 | 1.00 | 0.00 | H |
| ATOM | 1475 | CB | SER | 94 | 63.684 | 48.175 | 42.894 | 1.00 | 0.00 | C |
| ATOM | 1476 | HB2 | SER | 94 | 64.207 | 47.480 | 42.237 | 1.00 | 0.00 | H |
| ATOM | 1477 | HB3 | SER | 94 | 64.409 | 48.887 | 43.290 | 1.00 | 0.00 | H |
| ATOM | 1478 | OG | SER | 94 | 63.101 | 47.444 | 43.990 | 1.00 | 0.00 | O |
| ATOM | 1479 | HG | SER | 94 | 62.886 | 46.561 | 43.677 | 1.00 | 0.00 | H |
| ATOM | 1480 | C | SER | 94 | 61.566 | 47.980 | 41.402 | 1.00 | 0.00 | C |
| ATOM | 1481 | O | SER | 94 | 61.947 | 46.979 | 40.774 | 1.00 | 0.00 | O |
| ATOM | 1482 | N | PRO | 95 | 60.238 | 48.177 | 41.498 | 1.00 | 0.00 | N |
| ATOM | 1483 | CD | PRO | 95 | 59.533 | 49.265 | 42.260 | 1.00 | 0.00 | C |
| ATOM | 1484 | HD2 | PRO | 95 | 59.836 | 50.260 | 41.932 | 1.00 | 0.00 | H |
| ATOM | 1485 | HD3 | PRO | 95 | 59.662 | 49.124 | 43.334 | 1.00 | 0.00 | H |
| ATOM | 1486 | CG | PRO | 95 | 58.082 | 49.067 | 41.910 | 1.00 | 0.00 | C |
| ATOM | 1487 | HG2 | PRO | 95 | 57.836 | 49.649 | 41.020 | 1.00 | 0.00 | H |
| ATOM | 1488 | HG3 | PRO | 95 | 57.431 | 49.356 | 42.736 | 1.00 | 0.00 | H |
| ATOM | 1489 | CB | PRO | 95 | 57.968 | 47.613 | 41.606 | 1.00 | 0.00 | C |
| ATOM | 1490 | HB2 | PRO | 95 | 57.079 | 47.402 | 41.014 | 1.00 | 0.00 | H |
| ATOM | 1491 | HB3 | PRO | 95 | 57.941 | 47.046 | 42.538 | 1.00 | 0.00 | H |
| ATOM | 1492 | CA | PRO | 95 | 59.257 | 47.286 | 40.845 | 1.00 | 0.00 | C |
| ATOM | 1493 | HA | PRO | 95 | 59.516 | 46.240 | 41.013 | 1.00 | 0.00 | H |
| ATOM | 1494 | C | PRO | 95 | 59.087 | 47.535 | 39.327 | 1.00 | 0.00 | C |
| ATOM | 1495 | O | PRO | 95 | 59.433 | 48.609 | 38.860 | 1.00 | 0.00 | O |
| ATOM | 1496 | N | GLU | 96 | 58.490 | 46.603 | 38.624 | 1.00 | 0.00 | N |
| ATOM | 1497 | H | GLU | 96 | 58.266 | 45.719 | 39.055 | 1.00 | 0.00 | H |
| ATOM | 1498 | CA | GLU | 96 | 58.096 | 46.825 | 37.189 | 1.00 | 0.00 | C |

| | | | | | | | | | | |
|---|---|---|---|---|---|---|---|---|---|---|
| ATOM | 1499 | HA | GLU | 96 | 58.767 | 47.559 | 36.742 | 1.00 | 0.00 | H |
| ATOM | 1500 | CB | GLU | 96 | 58.246 | 45.526 | 36.395 | 1.00 | 0.00 | C |
| ATOM | 1501 | HB2 | GLU | 96 | 57.621 | 44.760 | 36.855 | 1.00 | 0.00 | H |
| ATOM | 1502 | HB3 | GLU | 96 | 57.884 | 45.698 | 35.380 | 1.00 | 0.00 | H |
| ATOM | 1503 | CG | GLU | 96 | 59.674 | 45.008 | 36.300 | 1.00 | 0.00 | C |
| ATOM | 1504 | HG2 | GLU | 96 | 60.293 | 45.767 | 35.819 | 1.00 | 0.00 | H |
| ATOM | 1505 | HG3 | GLU | 96 | 60.062 | 44.834 | 37.305 | 1.00 | 0.00 | H |
| ATOM | 1506 | CD | GLU | 96 | 59.781 | 43.726 | 35.523 | 1.00 | 0.00 | C |
| ATOM | 1507 | OE1 | GLU | 96 | 58.773 | 43.247 | 35.062 | 1.00 | 0.00 | O |
| ATOM | 1508 | OE2 | GLU | 96 | 60.873 | 43.224 | 35.390 | 1.00 | 0.00 | O |
| ATOM | 1509 | C | GLU | 96 | 56.658 | 47.350 | 37.012 | 1.00 | 0.00 | C |
| ATOM | 1510 | O | GLU | 96 | 56.316 | 47.838 | 35.947 | 1.00 | 0.00 | O |
| ATOM | 1511 | N | TYR | 97 | 55.863 | 47.322 | 38.017 | 1.00 | 0.00 | N |
| ATOM | 1512 | H | TYR | 97 | 56.215 | 46.960 | 38.892 | 1.00 | 0.00 | H |
| ATOM | 1513 | CA | TYR | 97 | 54.440 | 47.775 | 38.008 | 1.00 | 0.00 | C |
| ATOM | 1514 | HA | TYR | 97 | 54.376 | 48.756 | 37.536 | 1.00 | 0.00 | H |
| ATOM | 1515 | CB | TYR | 97 | 53.525 | 46.801 | 37.261 | 1.00 | 0.00 | C |
| ATOM | 1516 | HB2 | TYR | 97 | 52.492 | 47.135 | 37.366 | 1.00 | 0.00 | H |
| ATOM | 1517 | HB3 | TYR | 97 | 53.766 | 46.838 | 36.198 | 1.00 | 0.00 | H |
| ATOM | 1518 | CG | TYR | 97 | 53.633 | 45.372 | 37.743 | 1.00 | 0.00 | C |
| ATOM | 1519 | CD1 | TYR | 97 | 52.916 | 44.959 | 38.856 | 1.00 | 0.00 | C |
| ATOM | 1520 | HD1 | TYR | 97 | 52.319 | 45.650 | 39.395 | 1.00 | 0.00 | H |
| ATOM | 1521 | CE1 | TYR | 97 | 53.014 | 43.653 | 39.297 | 1.00 | 0.00 | C |
| ATOM | 1522 | HE1 | TYR | 97 | 52.442 | 43.326 | 40.151 | 1.00 | 0.00 | H |
| ATOM | 1523 | CZ | TYR | 97 | 53.834 | 42.757 | 38.620 | 1.00 | 0.00 | C |
| ATOM | 1524 | OH | TYR | 97 | 53.932 | 41.457 | 39.058 | 1.00 | 0.00 | O |
| ATOM | 1525 | HH | TYR | 97 | 54.456 | 40.914 | 38.464 | 1.00 | 0.00 | H |
| ATOM | 1526 | CE2 | TYR | 97 | 54.547 | 43.168 | 37.512 | 1.00 | 0.00 | C |
| ATOM | 1527 | HE2 | TYR | 97 | 55.193 | 42.476 | 36.991 | 1.00 | 0.00 | H |
| ATOM | 1528 | CD2 | TYR | 97 | 54.449 | 44.473 | 37.072 | 1.00 | 0.00 | C |

ATOM 1529 HD2 TYR 97 55.010 44.788 36.203 1.00 0.00 H
ATOM 1530 C TYR 97 54.031 47.906 39.485 1.00 0.00 C
ATOM 1531 O TYR 97 54.538 47.162 40.348 1.00 0.00 O
ATOM 1532 N HIP 98 53.077 48.763 39.817 1.00 0.00 N
ATOM 1533 H HIP 98 52.696 49.362 39.099 1.00 0.00 H
ATOM 1534 CA HIP 98 52.363 48.744 41.147 1.00 0.00 C
ATOM 1535 HA HIP 98 53.093 48.586 41.938 1.00 0.00 H
ATOM 1536 CB HIP 98 51.677 50.092 41.391 1.00 0.00 C
ATOM 1537 HB2 HIP 98 51.219 50.418 40.465 1.00 0.00 H
ATOM 1538 HB3 HIP 98 50.888 49.981 42.133 1.00 0.00 H
ATOM 1539 CG HIP 98 52.611 51.167 41.854 1.00 0.00 C
ATOM 1540 ND1 HIP 98 52.276 52.504 41.829 1.00 0.00 N
ATOM 1541 HD1 HIP 98 51.405 52.888 41.493 1.00 0.00 H
ATOM 1542 CE1 HIP 98 53.288 53.217 42.294 1.00 0.00 C
ATOM 1543 HE1 HIP 98 53.303 54.292 42.421 1.00 0.00 H
ATOM 1544 NE2 HIP 98 54.265 52.390 42.618 1.00 0.00 N
ATOM 1545 HE2 HIP 98 55.146 52.658 43.042 1.00 0.00 H
ATOM 1546 CD2 HIP 98 53.867 51.102 42.353 1.00 0.00 C
ATOM 1547 HD2 HIP 98 54.457 50.213 42.518 1.00 0.00 H
ATOM 1548 C HIP 98 51.289 47.576 41.268 1.00 0.00 C
ATOM 1549 O HIP 98 50.797 46.992 40.313 1.00 0.00 O
ATOM 1550 N LEU 99 50.990 47.181 42.573 1.00 0.00 N
ATOM 1551 H LEU 99 51.358 47.709 43.353 1.00 0.00 H
ATOM 1552 CA LEU 99 50.239 45.998 42.855 1.00 0.00 C
ATOM 1553 HA LEU 99 50.901 45.145 42.713 1.00 0.00 H
ATOM 1554 CB LEU 99 49.762 46.034 44.312 1.00 0.00 C
ATOM 1555 HB2 LEU 99 50.614 46.236 44.963 1.00 0.00 H
ATOM 1556 HB3 LEU 99 49.082 46.880 44.403 1.00 0.00 H
ATOM 1557 CG LEU 99 49.045 44.771 44.807 1.00 0.00 C
ATOM 1558 HG LEU 99 48.310 44.454 44.071 1.00 0.00 H

ATOM 1559 CD1 LEU 99 50.058 43.649 44.987 1.00 0.00 C

ATOM 1560 HD11 LEU 99 49.548 42.748 45.327 1.00 0.00 H

ATOM 1561 HD12 LEU 99 50.547 43.420 44.041 1.00 0.00 H

ATOM 1562 HD13 LEU 99 50.813 43.929 45.724 1.00 0.00 H

ATOM 1563 CD2 LEU 99 48.327 45.075 46.114 1.00 0.00 C

ATOM 1564 HD21 LEU 99 47.836 44.170 46.456 1.00 0.00 H

ATOM 1565 HD22 LEU 99 49.025 45.419 46.872 1.00 0.00 H

ATOM 1566 HD23 LEU 99 47.550 45.815 45.947 1.00 0.00 H

ATOM 1567 C LEU 99 49.031 45.868 41.878 1.00 0.00 C

ATOM 1568 O LEU 99 48.255 46.771 41.711 1.00 0.00 O

ATOM 1569 N VAL 100 48.804 44.689 41.268 1.00 0.00 N

ATOM 1570 H VAL 100 49.472 43.954 41.441 1.00 0.00 H

ATOM 1571 CA VAL 100 47.669 44.326 40.314 1.00 0.00 C

ATOM 1572 HA VAL 100 46.948 45.141 40.315 1.00 0.00 H

ATOM 1573 CB VAL 100 48.190 44.103 38.881 1.00 0.00 C

ATOM 1574 HB VAL 100 47.357 43.834 38.230 1.00 0.00 H

ATOM 1575 CG1 VAL 100 48.802 45.383 38.332 1.00 0.00 C

ATOM 1576 HG11 VAL 100 48.835 45.327 37.244 1.00 0.00 H

ATOM 1577 HG12 VAL 100 48.219 46.256 38.626 1.00 0.00 H

ATOM 1578 HG13 VAL 100 49.823 45.483 38.688 1.00 0.00 H

ATOM 1579 CG2 VAL 100 49.207 42.971 38.870 1.00 0.00 C

ATOM 1580 HG21 VAL 100 49.691 42.926 37.894 1.00 0.00 H

ATOM 1581 HG22 VAL 100 49.985 43.124 39.617 1.00 0.00 H

ATOM 1582 HG23 VAL 100 48.711 42.022 39.034 1.00 0.00 H

ATOM 1583 C VAL 100 46.944 43.090 40.718 1.00 0.00 C

ATOM 1584 O VAL 100 45.948 42.721 40.112 1.00 0.00 O

ATOM 1585 N SER 101 47.546 42.272 41.648 1.00 0.00 N

ATOM 1586 H SER 101 48.375 42.604 42.117 1.00 0.00 H

ATOM 1587 CA SER 101 47.103 40.897 41.971 1.00 0.00 C

ATOM 1588 HA SER 101 46.313 40.612 41.275 1.00 0.00 H

ATOM 1589 CB SER 101 48.268 39.961 41.715 1.00 0.00 C
ATOM 1590 HB2 SER 101 49.134 40.279 42.290 1.00 0.00 H
ATOM 1591 HB3 SER 101 47.984 38.955 42.023 1.00 0.00 H
ATOM 1592 OG SER 101 48.591 39.923 40.352 1.00 0.00 O
ATOM 1593 HG SER 101 49.034 40.744 40.126 1.00 0.00 H
ATOM 1594 C SER 101 46.551 40.623 43.414 1.00 0.00 C
ATOM 1595 O SER 101 47.273 40.695 44.407 1.00 0.00 O
ATOM 1596 N MET 102 45.271 40.209 43.521 1.00 0.00 N
ATOM 1597 H MET 102 44.701 40.146 42.687 1.00 0.00 H
ATOM 1598 CA MET 102 44.659 39.779 44.785 1.00 0.00 C
ATOM 1599 HA MET 102 45.441 39.511 45.492 1.00 0.00 H
ATOM 1600 CB MET 102 43.847 40.926 45.384 1.00 0.00 C
ATOM 1601 HB2 MET 102 44.524 41.757 45.574 1.00 0.00 H
ATOM 1602 HB3 MET 102 43.114 41.267 44.652 1.00 0.00 H
ATOM 1603 CG MET 102 43.131 40.580 46.683 1.00 0.00 C
ATOM 1604 HG2 MET 102 43.756 39.910 47.274 1.00 0.00 H
ATOM 1605 HG3 MET 102 42.971 41.493 47.256 1.00 0.00 H
ATOM 1606 SD MET 102 41.528 39.800 46.407 1.00 0.00 S
ATOM 1607 CE MET 102 40.529 41.223 45.979 1.00 0.00 C
ATOM 1608 HE1 MET 102 41.158 42.031 45.607 1.00 0.00 H
ATOM 1609 HE2 MET 102 39.812 40.945 45.209 1.00 0.00 H
ATOM 1610 HE3 MET 102 39.992 41.564 46.860 1.00 0.00 H
ATOM 1611 C MET 102 43.763 38.509 44.562 1.00 0.00 C
ATOM 1612 O MET 102 42.867 38.545 43.719 1.00 0.00 O
ATOM 1613 N HID 103 43.793 37.492 45.416 1.00 0.00 N
ATOM 1614 H HID 103 44.458 37.522 46.177 1.00 0.00 H
ATOM 1615 CA HID 103 43.022 36.277 45.297 1.00 0.00 C
ATOM 1616 HA HID 103 42.095 36.493 44.765 1.00 0.00 H
ATOM 1617 CB HID 103 43.781 35.241 44.501 1.00 0.00 C
ATOM 1618 HB2 HID 103 43.099 34.432 44.238 1.00 0.00 H

ATOM 1619 HB3 HID 103 44.064 35.724 43.568 1.00 0.00 H

ATOM 1620 CG HID 103 45.000 34.626 45.049 1.00 0.00 C

ATOM 1621 ND1 HID 103 46.273 34.873 44.651 1.00 0.00 N

ATOM 1622 HD1 HID 103 46.533 35.464 43.875 1.00 0.00 H

ATOM 1623 CE1 HID 103 47.145 34.292 45.531 1.00 0.00 C

ATOM 1624 HE1 HID 103 48.223 34.344 45.496 1.00 0.00 H

ATOM 1625 NE2 HID 103 46.399 33.543 46.403 1.00 0.00 N

ATOM 1626 CD2 HID 103 45.078 33.710 46.103 1.00 0.00 C

ATOM 1627 HD2 HID 103 44.247 33.295 46.653 1.00 0.00 H

ATOM 1628 C HID 103 42.619 35.573 46.655 1.00 0.00 C

ATOM 1629 O HID 103 43.104 35.919 47.717 1.00 0.00 O

ATOM 1630 N PHE 104 41.750 34.590 46.614 1.00 0.00 N

ATOM 1631 H PHE 104 41.448 34.302 45.693 1.00 0.00 H

ATOM 1632 CA PHE 104 41.009 34.009 47.686 1.00 0.00 C

ATOM 1633 HA PHE 104 41.170 34.601 48.585 1.00 0.00 H

ATOM 1634 CB PHE 104 39.515 34.070 47.359 1.00 0.00 C

ATOM 1635 HB2 PHE 104 39.337 33.610 46.385 1.00 0.00 H

ATOM 1636 HB3 PHE 104 38.972 33.479 48.098 1.00 0.00 H

ATOM 1637 CG PHE 104 38.951 35.462 47.362 1.00 0.00 C

ATOM 1638 CD1 PHE 104 39.048 36.268 46.238 1.00 0.00 C

ATOM 1639 HD1 PHE 104 39.518 35.888 45.342 1.00 0.00 H

ATOM 1640 CE1 PHE 104 38.529 37.549 46.239 1.00 0.00 C

ATOM 1641 HE1 PHE 104 38.630 38.175 45.369 1.00 0.00 H

ATOM 1642 CZ PHE 104 37.906 38.039 47.367 1.00 0.00 C

ATOM 1643 HZ PHE 104 37.483 39.032 47.361 1.00 0.00 H

ATOM 1644 CE2 PHE 104 37.801 37.248 48.493 1.00 0.00 C

ATOM 1645 HE2 PHE 104 37.309 37.626 49.377 1.00 0.00 H

ATOM 1646 CD2 PHE 104 38.322 35.969 48.489 1.00 0.00 C

ATOM 1647 HD2 PHE 104 38.249 35.365 49.382 1.00 0.00 H

ATOM 1648 C PHE 104 41.420 32.561 47.979 1.00 0.00 C

ATOM 1649 O PHE 104 41.521 31.697 47.054 1.00 0.00 O

ATOM 1650 N HID 105 41.359 32.195 49.286 1.00 0.00 N

ATOM 1651 H HID 105 41.238 32.944 49.954 1.00 0.00 H

ATOM 1652 CA HID 105 41.388 30.782 49.906 1.00 0.00 C

ATOM 1653 HA HID 105 41.201 30.066 49.104 1.00 0.00 H

ATOM 1654 CB HID 105 42.740 30.477 50.474 1.00 0.00 C

ATOM 1655 HB2 HID 105 42.953 31.151 51.299 1.00 0.00 H

ATOM 1656 HB3 HID 105 42.732 29.463 50.875 1.00 0.00 H

ATOM 1657 CG HID 105 43.869 30.571 49.491 1.00 0.00 C

ATOM 1658 ND1 HID 105 44.273 29.641 48.621 1.00 0.00 N

ATOM 1659 HD1 HID 105 43.863 28.724 48.499 1.00 0.00 H

ATOM 1660 CE1 HID 105 45.319 30.172 47.898 1.00 0.00 C

ATOM 1661 HE1 HID 105 45.848 29.685 47.094 1.00 0.00 H

ATOM 1662 NE2 HID 105 45.605 31.400 48.388 1.00 0.00 N

ATOM 1663 CD2 HID 105 44.615 31.688 49.311 1.00 0.00 C

ATOM 1664 HD2 HID 105 44.518 32.610 49.868 1.00 0.00 H

ATOM 1665 C HID 105 40.327 30.559 51.002 1.00 0.00 C

ATOM 1666 O HID 105 40.225 31.410 51.897 1.00 0.00 O

ATOM 1667 N SER 106 39.706 29.398 50.940 1.00 0.00 N

ATOM 1668 H SER 106 39.895 28.740 50.196 1.00 0.00 H

ATOM 1669 CA SER 106 38.817 29.023 52.013 1.00 0.00 C

ATOM 1670 HA SER 106 39.225 29.367 52.958 1.00 0.00 H

ATOM 1671 CB SER 106 37.491 29.742 51.856 1.00 0.00 C

ATOM 1672 HB2 SER 106 37.636 30.822 51.877 1.00 0.00 H

ATOM 1673 HB3 SER 106 37.061 29.460 50.896 1.00 0.00 H

ATOM 1674 OG SER 106 36.599 29.376 52.873 1.00 0.00 O

ATOM 1675 HG SER 106 36.726 29.948 53.639 1.00 0.00 H

ATOM 1676 C SER 106 38.613 27.483 52.020 1.00 0.00 C

ATOM 1677 O SER 106 38.202 26.916 51.007 1.00 0.00 O

ATOM 1678 N PRO 107 39.091 26.747 53.020 1.00 0.00 N

| | | | | | | | | | | |
|---|---|---|---|---|---|---|---|---|---|---|
| ATOM | 1679 | CD | PRO | 107 | 39.048 | 25.324 | 52.985 | 1.00 | 0.00 | C |
| ATOM | 1680 | HD2 | PRO | 107 | 39.901 | 24.946 | 52.425 | 1.00 | 0.00 | H |
| ATOM | 1681 | HD3 | PRO | 107 | 38.107 | 24.948 | 52.582 | 1.00 | 0.00 | H |
| ATOM | 1682 | CG | PRO | 107 | 39.162 | 24.976 | 54.445 | 1.00 | 0.00 | C |
| ATOM | 1683 | HG2 | PRO | 107 | 39.531 | 23.959 | 54.583 | 1.00 | 0.00 | H |
| ATOM | 1684 | HG3 | PRO | 107 | 38.189 | 25.092 | 54.926 | 1.00 | 0.00 | H |
| ATOM | 1685 | CB | PRO | 107 | 40.117 | 25.982 | 54.992 | 1.00 | 0.00 | C |
| ATOM | 1686 | HB2 | PRO | 107 | 41.138 | 25.672 | 54.761 | 1.00 | 0.00 | H |
| ATOM | 1687 | HB3 | PRO | 107 | 39.998 | 26.104 | 56.069 | 1.00 | 0.00 | H |
| ATOM | 1688 | CA | PRO | 107 | 39.775 | 27.272 | 54.241 | 1.00 | 0.00 | C |
| ATOM | 1689 | HA | PRO | 107 | 39.052 | 27.844 | 54.824 | 1.00 | 0.00 | H |
| ATOM | 1690 | C | PRO | 107 | 41.049 | 28.112 | 54.001 | 1.00 | 0.00 | C |
| ATOM | 1691 | O | PRO | 107 | 41.690 | 28.136 | 52.974 | 1.00 | 0.00 | O |
| ATOM | 1692 | N | ALA | 108 | 41.302 | 28.955 | 54.947 | 1.00 | 0.00 | N |
| ATOM | 1693 | H | ALA | 108 | 40.725 | 28.951 | 55.774 | 1.00 | 0.00 | H |
| ATOM | 1694 | CA | ALA | 108 | 42.459 | 29.888 | 54.935 | 1.00 | 0.00 | C |
| ATOM | 1695 | HA | ALA | 108 | 42.415 | 30.517 | 54.047 | 1.00 | 0.00 | H |
| ATOM | 1696 | CB | ALA | 108 | 42.327 | 30.783 | 56.230 | 1.00 | 0.00 | C |
| ATOM | 1697 | HB1 | ALA | 108 | 43.213 | 31.411 | 56.297 | 1.00 | 0.00 | H |
| ATOM | 1698 | HB2 | ALA | 108 | 41.429 | 31.399 | 56.172 | 1.00 | 0.00 | H |
| ATOM | 1699 | HB3 | ALA | 108 | 42.278 | 30.151 | 57.117 | 1.00 | 0.00 | H |
| ATOM | 1700 | C | ALA | 108 | 43.702 | 28.994 | 54.859 | 1.00 | 0.00 | C |
| ATOM | 1701 | O | ALA | 108 | 43.618 | 27.873 | 55.251 | 1.00 | 0.00 | O |
| ATOM | 1702 | N | GLU | 109 | 44.836 | 29.422 | 54.239 | 1.00 | 0.00 | N |
| ATOM | 1703 | H | GLU | 109 | 44.869 | 30.348 | 53.849 | 1.00 | 0.00 | H |
| ATOM | 1704 | CA | GLU | 109 | 46.106 | 28.635 | 54.273 | 1.00 | 0.00 | C |
| ATOM | 1705 | HA | GLU | 109 | 45.869 | 27.572 | 54.331 | 1.00 | 0.00 | H |
| ATOM | 1706 | CB | GLU | 109 | 46.903 | 28.857 | 52.974 | 1.00 | 0.00 | C |
| ATOM | 1707 | HB2 | GLU | 109 | 47.121 | 29.919 | 52.855 | 1.00 | 0.00 | H |
| ATOM | 1708 | HB3 | GLU | 109 | 47.848 | 28.327 | 53.042 | 1.00 | 0.00 | H |

ATOM 1709 CG GLU 109 46.189 28.348 51.762 1.00 0.00 C

ATOM 1710 HG2 GLU 109 45.835 27.340 51.961 1.00 0.00 H

ATOM 1711 HG3 GLU 109 45.347 28.993 51.510 1.00 0.00 H

ATOM 1712 CD GLU 109 47.182 28.319 50.634 1.00 0.00 C

ATOM 1713 OE1 GLU 109 47.966 29.276 50.437 1.00 0.00 O

ATOM 1714 OE2 GLU 109 47.109 27.313 49.919 1.00 0.00 O

ATOM 1715 C GLU 109 46.984 28.980 55.503 1.00 0.00 C

ATOM 1716 O GLU 109 47.547 28.100 56.127 1.00 0.00 O

ATOM 1717 N HIE 110 47.027 30.206 56.004 1.00 0.00 N

ATOM 1718 H HIE 110 46.511 30.938 55.532 1.00 0.00 H

ATOM 1719 CA HIE 110 47.820 30.614 57.196 1.00 0.00 C

ATOM 1720 HA HIE 110 48.633 29.904 57.352 1.00 0.00 H

ATOM 1721 CB HIE 110 48.449 31.994 56.983 1.00 0.00 C

ATOM 1722 HB2 HIE 110 47.680 32.694 56.659 1.00 0.00 H

ATOM 1723 HB3 HIE 110 48.842 32.355 57.934 1.00 0.00 H

ATOM 1724 CG HIE 110 49.583 31.994 56.005 1.00 0.00 C

ATOM 1725 ND1 HIE 110 49.412 32.283 54.667 1.00 0.00 N

ATOM 1726 CE1 HIE 110 50.579 32.207 54.051 1.00 0.00 C

ATOM 1727 HE1 HIE 110 50.753 32.402 53.003 1.00 0.00 H

ATOM 1728 NE2 HIE 110 51.498 31.878 54.940 1.00 0.00 N

ATOM 1729 HE2 HIE 110 52.484 31.809 54.748 1.00 0.00 H

ATOM 1730 CD2 HIE 110 50.902 31.739 56.169 1.00 0.00 C

ATOM 1731 HD2 HIE 110 51.386 31.481 57.096 1.00 0.00 H

ATOM 1732 C HIE 110 46.956 30.636 58.476 1.00 0.00 C

ATOM 1733 O HIE 110 45.769 30.859 58.425 1.00 0.00 O

ATOM 1734 N VAL 111 47.657 30.459 59.658 1.00 0.00 N

ATOM 1735 H VAL 111 48.655 30.307 59.636 1.00 0.00 H

ATOM 1736 CA VAL 111 46.942 30.446 60.987 1.00 0.00 C

ATOM 1737 HA VAL 111 45.871 30.565 60.817 1.00 0.00 H

ATOM 1738 CB VAL 111 47.149 29.086 61.679 1.00 0.00 C

ATOM 1739 HB VAL 111 48.217 28.907 61.796 1.00 0.00 H
ATOM 1740 CG1 VAL 111 46.510 29.088 63.060 1.00 0.00 C
ATOM 1741 HG11 VAL 111 47.047 29.745 63.745 1.00 0.00 H
ATOM 1742 HG12 VAL 111 45.464 29.390 62.997 1.00 0.00 H
ATOM 1743 HG13 VAL 111 46.559 28.081 63.476 1.00 0.00 H
ATOM 1744 CG2 VAL 111 46.571 27.973 60.819 1.00 0.00 C
ATOM 1745 HG21 VAL 111 45.503 28.137 60.669 1.00 0.00 H
ATOM 1746 HG22 VAL 111 47.059 27.933 59.847 1.00 0.00 H
ATOM 1747 HG23 VAL 111 46.716 27.010 61.310 1.00 0.00 H
ATOM 1748 C VAL 111 47.407 31.573 61.960 1.00 0.00 C
ATOM 1749 O VAL 111 48.560 31.858 62.284 1.00 0.00 O
ATOM 1750 N LEU 112 46.381 32.261 62.454 1.00 0.00 N
ATOM 1751 H LEU 112 45.442 32.008 62.181 1.00 0.00 H
ATOM 1752 CA LEU 112 46.544 33.430 63.449 1.00 0.00 C
ATOM 1753 HA LEU 112 47.560 33.811 63.361 1.00 0.00 H
ATOM 1754 CB LEU 112 45.578 34.575 63.119 1.00 0.00 C
ATOM 1755 HB2 LEU 112 44.610 34.153 62.844 1.00 0.00 H
ATOM 1756 HB3 LEU 112 45.424 35.165 64.024 1.00 0.00 H
ATOM 1757 CG LEU 112 46.046 35.546 62.027 1.00 0.00 C
ATOM 1758 HG LEU 112 46.992 35.989 62.333 1.00 0.00 H
ATOM 1759 CD1 LEU 112 46.242 34.786 60.722 1.00 0.00 C
ATOM 1760 HD11 LEU 112 46.364 35.490 59.899 1.00 0.00 H
ATOM 1761 HD12 LEU 112 47.150 34.187 60.772 1.00 0.00 H
ATOM 1762 HD13 LEU 112 45.388 34.143 60.510 1.00 0.00 H
ATOM 1763 CD2 LEU 112 45.021 36.658 61.864 1.00 0.00 C
ATOM 1764 HD21 LEU 112 45.320 37.321 61.052 1.00 0.00 H
ATOM 1765 HD22 LEU 112 44.037 36.244 61.649 1.00 0.00 H
ATOM 1766 HD23 LEU 112 44.966 37.242 62.783 1.00 0.00 H
ATOM 1767 C LEU 112 46.292 32.975 64.902 1.00 0.00 C
ATOM 1768 O LEU 112 45.393 32.120 65.063 1.00 0.00 O

```
ATOM   1769  N   PRO   113      46.905  33.615  65.935  1.00  0.00           N
ATOM   1770  CD  PRO   113      47.986  34.561  65.696  1.00  0.00           C
ATOM   1771  HD2 PRO   113      47.729  35.313  64.950  1.00  0.00           H
ATOM   1772  HD3 PRO   113      48.898  34.030  65.429  1.00  0.00           H
ATOM   1773  CG  PRO   113      48.128  35.207  67.048  1.00  0.00           C
ATOM   1774  HG2 PRO   113      47.422  36.036  67.126  1.00  0.00           H
ATOM   1775  HG3 PRO   113      49.140  35.571  67.215  1.00  0.00           H
ATOM   1776  CB  PRO   113      47.742  34.137  68.012  1.00  0.00           C
ATOM   1777  HB2 PRO   113      47.423  34.561  68.965  1.00  0.00           H
ATOM   1778  HB3 PRO   113      48.586  33.461  68.161  1.00  0.00           H
ATOM   1779  CA  PRO   113      46.608  33.386  67.306  1.00  0.00           C
ATOM   1780  HA  PRO   113      46.666  32.322  67.541  1.00  0.00           H
ATOM   1781  C   PRO   113      45.225  33.938  67.684  1.00  0.00           C
ATOM   1782  O   PRO   113      44.740  34.886  67.095  1.00  0.00           O
ATOM   1783  N   CYS   114      44.541  33.210  68.617  1.00  0.00           N
ATOM   1784  H   CYS   114      44.969  32.403  69.049  1.00  0.00           H
ATOM   1785  CA  CYS   114      43.201  33.693  69.138  1.00  0.00           C
ATOM   1786  HA  CYS   114      42.747  32.881  69.707  1.00  0.00           H
ATOM   1787  CB  CYS   114      43.396  34.890  70.068  1.00  0.00           C
ATOM   1788  HB2 CYS   114      43.866  35.703  69.512  1.00  0.00           H
ATOM   1789  HB3 CYS   114      42.421  35.236  70.413  1.00  0.00           H
ATOM   1790  SG  CYS   114      44.406  34.536  71.526  1.00  0.00           S
ATOM   1791  HG  CYS   114      43.521  33.716  72.118  1.00  0.00           H
ATOM   1792  C   CYS   114      42.203  34.104  68.007  1.00  0.00           C
ATOM   1793  O   CYS   114      41.740  35.244  67.933  1.00  0.00           O
ATOM   1794  N   ALA   115      42.074  33.296  66.956  1.00  0.00           N
ATOM   1795  H   ALA   115      42.572  32.418  66.986  1.00  0.00           H
ATOM   1796  CA  ALA   115      41.213  33.514  65.728  1.00  0.00           C
ATOM   1797  HA  ALA   115      40.378  34.161  66.000  1.00  0.00           H
ATOM   1798  CB  ALA   115      42.085  34.248  64.788  1.00  0.00           C
```

| | | | | | | | | | | |
|---|---|---|---|---|---|---|---|---|---|---|
| ATOM | 1799 | HB1 | ALA | 115 | 42.918 | 33.613 | 64.484 | 1.00 | 0.00 | H |
| ATOM | 1800 | HB2 | ALA | 115 | 41.513 | 34.532 | 63.904 | 1.00 | 0.00 | H |
| ATOM | 1801 | HB3 | ALA | 115 | 42.476 | 35.155 | 65.253 | 1.00 | 0.00 | H |
| ATOM | 1802 | C | ALA | 115 | 40.643 | 32.240 | 65.099 | 1.00 | 0.00 | C |
| ATOM | 1803 | O | ALA | 115 | 41.276 | 31.222 | 65.189 | 1.00 | 0.00 | O |
| ATOM | 1804 | N | ASN | 116 | 39.482 | 32.339 | 64.444 | 1.00 | 0.00 | N |
| ATOM | 1805 | H | ASN | 116 | 38.970 | 33.207 | 64.386 | 1.00 | 0.00 | H |
| ATOM | 1806 | CA | ASN | 116 | 39.011 | 31.126 | 63.696 | 1.00 | 0.00 | C |
| ATOM | 1807 | HA | ASN | 116 | 39.826 | 30.423 | 63.521 | 1.00 | 0.00 | H |
| ATOM | 1808 | CB | ASN | 116 | 37.914 | 30.386 | 64.439 | 1.00 | 0.00 | C |
| ATOM | 1809 | HB2 | ASN | 116 | 37.080 | 31.065 | 64.628 | 1.00 | 0.00 | H |
| ATOM | 1810 | HB3 | ASN | 116 | 37.549 | 29.572 | 63.811 | 1.00 | 0.00 | H |
| ATOM | 1811 | CG | ASN | 116 | 38.386 | 29.815 | 65.747 | 1.00 | 0.00 | C |
| ATOM | 1812 | OD1 | ASN | 116 | 38.067 | 30.484 | 66.826 | 1.00 | 0.00 | O |
| ATOM | 1813 | ND2 | ASN | 116 | 39.040 | 28.767 | 65.781 | 1.00 | 0.00 | N |
| ATOM | 1814 | HD21 | ASN | 116 | 39.249 | 28.276 | 64.926 | 1.00 | 0.00 | H |
| ATOM | 1815 | HD22 | ASN | 116 | 39.383 | 28.431 | 66.668 | 1.00 | 0.00 | H |
| ATOM | 1816 | C | ASN | 116 | 38.524 | 31.630 | 62.305 | 1.00 | 0.00 | C |
| ATOM | 1817 | O | ASN | 116 | 37.286 | 31.798 | 62.054 | 1.00 | 0.00 | O |
| ATOM | 1818 | N | PRO | 117 | 39.421 | 31.851 | 61.297 | 1.00 | 0.00 | N |
| ATOM | 1819 | CD | PRO | 117 | 40.846 | 31.759 | 61.487 | 1.00 | 0.00 | C |
| ATOM | 1820 | HD2 | PRO | 117 | 41.175 | 30.730 | 61.338 | 1.00 | 0.00 | H |
| ATOM | 1821 | HD3 | PRO | 117 | 41.179 | 32.144 | 62.448 | 1.00 | 0.00 | H |
| ATOM | 1822 | CG | PRO | 117 | 41.360 | 32.643 | 60.382 | 1.00 | 0.00 | C |
| ATOM | 1823 | HG2 | PRO | 117 | 42.364 | 32.347 | 60.079 | 1.00 | 0.00 | H |
| ATOM | 1824 | HG3 | PRO | 117 | 41.355 | 33.681 | 60.718 | 1.00 | 0.00 | H |
| ATOM | 1825 | CB | PRO | 117 | 40.373 | 32.470 | 59.279 | 1.00 | 0.00 | C |
| ATOM | 1826 | HB2 | PRO | 117 | 40.564 | 31.522 | 58.772 | 1.00 | 0.00 | H |
| ATOM | 1827 | HB3 | PRO | 117 | 40.399 | 33.287 | 58.561 | 1.00 | 0.00 | H |
| ATOM | 1828 | CA | PRO | 117 | 39.018 | 32.417 | 59.992 | 1.00 | 0.00 | C |

| | | | | | | | | | | |
|---|---|---|---|---|---|---|---|---|---|---|
| ATOM | 1829 | HA | PRO | 117 | 38.659 | 33.435 | 60.154 | 1.00 | 0.00 | H |
| ATOM | 1830 | C | PRO | 117 | 37.955 | 31.613 | 59.129 | 1.00 | 0.00 | C |
| ATOM | 1831 | O | PRO | 117 | 37.879 | 30.380 | 59.029 | 1.00 | 0.00 | O |
| ATOM | 1832 | N | ASP | 118 | 37.160 | 32.403 | 58.367 | 1.00 | 0.00 | N |
| ATOM | 1833 | H | ASP | 118 | 37.225 | 33.408 | 58.438 | 1.00 | 0.00 | H |
| ATOM | 1834 | CA | ASP | 118 | 36.286 | 31.844 | 57.384 | 1.00 | 0.00 | C |
| ATOM | 1835 | HA | ASP | 118 | 36.056 | 30.811 | 57.643 | 1.00 | 0.00 | H |
| ATOM | 1836 | CB | ASP | 118 | 34.957 | 32.596 | 57.483 | 1.00 | 0.00 | C |
| ATOM | 1837 | HB2 | ASP | 118 | 35.124 | 33.659 | 57.295 | 1.00 | 0.00 | H |
| ATOM | 1838 | HB3 | ASP | 118 | 34.282 | 32.226 | 56.710 | 1.00 | 0.00 | H |
| ATOM | 1839 | CG | ASP | 118 | 34.270 | 32.411 | 58.829 | 1.00 | 0.00 | C |
| ATOM | 1840 | OD1 | ASP | 118 | 34.062 | 31.286 | 59.218 | 1.00 | 0.00 | O |
| ATOM | 1841 | OD2 | ASP | 118 | 33.959 | 33.396 | 59.455 | 1.00 | 0.00 | O |
| ATOM | 1842 | C | ASP | 118 | 36.728 | 31.815 | 55.901 | 1.00 | 0.00 | C |
| ATOM | 1843 | O | ASP | 118 | 36.356 | 31.002 | 55.027 | 1.00 | 0.00 | O |
| ATOM | 1844 | N | ALA | 119 | 37.496 | 32.787 | 55.559 | 1.00 | 0.00 | N |
| ATOM | 1845 | H | ALA | 119 | 37.768 | 33.449 | 56.273 | 1.00 | 0.00 | H |
| ATOM | 1846 | CA | ALA | 119 | 38.057 | 33.021 | 54.199 | 1.00 | 0.00 | C |
| ATOM | 1847 | HA | ALA | 119 | 38.301 | 32.045 | 53.777 | 1.00 | 0.00 | H |
| ATOM | 1848 | CB | ALA | 119 | 36.950 | 33.632 | 53.273 | 1.00 | 0.00 | C |
| ATOM | 1849 | HB1 | ALA | 119 | 37.379 | 33.873 | 52.300 | 1.00 | 0.00 | H |
| ATOM | 1850 | HB2 | ALA | 119 | 36.143 | 32.913 | 53.132 | 1.00 | 0.00 | H |
| ATOM | 1851 | HB3 | ALA | 119 | 36.539 | 34.530 | 53.724 | 1.00 | 0.00 | H |
| ATOM | 1852 | C | ALA | 119 | 39.439 | 33.792 | 54.274 | 1.00 | 0.00 | C |
| ATOM | 1853 | O | ALA | 119 | 39.708 | 34.287 | 55.378 | 1.00 | 0.00 | O |
| ATOM | 1854 | N | GLU | 120 | 40.260 | 33.789 | 53.261 | 1.00 | 0.00 | N |
| ATOM | 1855 | H | GLU | 120 | 40.002 | 33.294 | 52.419 | 1.00 | 0.00 | H |
| ATOM | 1856 | CA | GLU | 120 | 41.602 | 34.378 | 53.309 | 1.00 | 0.00 | C |
| ATOM | 1857 | HA | GLU | 120 | 41.629 | 35.118 | 54.106 | 1.00 | 0.00 | H |
| ATOM | 1858 | CB | GLU | 120 | 42.633 | 33.295 | 53.635 | 1.00 | 0.00 | C |

ATOM 1859 HB2 GLU 120 42.390 32.892 54.612 1.00 0.00 H

ATOM 1860 HB3 GLU 120 42.559 32.500 52.902 1.00 0.00 H

ATOM 1861 CG GLU 120 44.072 33.790 53.682 1.00 0.00 C

ATOM 1862 HG2 GLU 120 44.358 34.203 52.714 1.00 0.00 H

ATOM 1863 HG3 GLU 120 44.154 34.581 54.427 1.00 0.00 H

ATOM 1864 CD GLU 120 45.055 32.700 54.005 1.00 0.00 C

ATOM 1865 OE1 GLU 120 45.295 31.872 53.159 1.00 0.00 O

ATOM 1866 OE2 GLU 120 45.568 32.695 55.099 1.00 0.00 O

ATOM 1867 C GLU 120 41.999 35.082 52.007 1.00 0.00 C

ATOM 1868 O GLU 120 41.948 34.503 50.891 1.00 0.00 O

ATOM 1869 N LEU 121 42.735 36.236 52.070 1.00 0.00 N

ATOM 1870 H LEU 121 42.883 36.617 52.991 1.00 0.00 H

ATOM 1871 CA LEU 121 43.353 37.013 50.980 1.00 0.00 C

ATOM 1872 HA LEU 121 42.979 36.627 50.035 1.00 0.00 H

ATOM 1873 CB LEU 121 42.961 38.492 51.084 1.00 0.00 C

ATOM 1874 HB2 LEU 121 43.474 38.910 51.945 1.00 0.00 H

ATOM 1875 HB3 LEU 121 43.333 39.014 50.201 1.00 0.00 H

ATOM 1876 CG LEU 121 41.460 38.772 51.238 1.00 0.00 C

ATOM 1877 HG LEU 121 41.064 38.238 52.102 1.00 0.00 H

ATOM 1878 CD1 LEU 121 41.244 40.260 51.480 1.00 0.00 C

ATOM 1879 HD11 LEU 121 40.187 40.464 51.630 1.00 0.00 H

ATOM 1880 HD12 LEU 121 41.785 40.575 52.373 1.00 0.00 H

ATOM 1881 HD13 LEU 121 41.604 40.832 50.625 1.00 0.00 H

ATOM 1882 CD2 LEU 121 40.726 38.309 49.989 1.00 0.00 C

ATOM 1883 HD21 LEU 121 39.673 38.567 50.073 1.00 0.00 H

ATOM 1884 HD22 LEU 121 41.147 38.786 49.106 1.00 0.00 H

ATOM 1885 HD23 LEU 121 40.800 37.227 49.881 1.00 0.00 H

ATOM 1886 C LEU 121 44.860 36.897 50.997 1.00 0.00 C

ATOM 1887 O LEU 121 45.598 36.981 52.010 1.00 0.00 O

ATOM 1888 N HIE 122 45.357 36.923 49.726 1.00 0.00 N

ATOM 1889 H HIE 122 44.727 36.884 48.936 1.00 0.00 H

ATOM 1890 CA HIE 122 46.765 37.123 49.470 1.00 0.00 C

ATOM 1891 HA HIE 122 47.267 37.396 50.388 1.00 0.00 H

ATOM 1892 CB HIE 122 47.533 35.921 48.889 1.00 0.00 C

ATOM 1893 HB2 HIE 122 46.976 35.536 48.039 1.00 0.00 H

ATOM 1894 HB3 HIE 122 48.506 36.257 48.533 1.00 0.00 H

ATOM 1895 CG HIE 122 47.767 34.800 49.889 1.00 0.00 C

ATOM 1896 ND1 HIE 122 47.769 33.425 49.618 1.00 0.00 N

ATOM 1897 CE1 HIE 122 47.940 32.725 50.750 1.00 0.00 C

ATOM 1898 HE1 HIE 122 47.953 31.653 50.860 1.00 0.00 H

ATOM 1899 NE2 HIE 122 48.303 33.640 51.686 1.00 0.00 N

ATOM 1900 HE2 HIE 122 48.522 33.414 52.648 1.00 0.00 H

ATOM 1901 CD2 HIE 122 48.188 34.899 51.191 1.00 0.00 C

ATOM 1902 HD2 HIE 122 48.345 35.820 51.728 1.00 0.00 H

ATOM 1903 C HIE 122 46.877 38.323 48.516 1.00 0.00 C

ATOM 1904 O HIE 122 46.183 38.385 47.560 1.00 0.00 O

ATOM 1905 N LEU 123 47.837 39.219 48.705 1.00 0.00 N

ATOM 1906 H LEU 123 48.396 39.135 49.543 1.00 0.00 H

ATOM 1907 CA LEU 123 48.285 40.232 47.716 1.00 0.00 C

ATOM 1908 HA LEU 123 47.634 40.222 46.844 1.00 0.00 H

ATOM 1909 CB LEU 123 48.323 41.648 48.305 1.00 0.00 C

ATOM 1910 HB2 LEU 123 49.127 41.699 49.035 1.00 0.00 H

ATOM 1911 HB3 LEU 123 48.600 42.329 47.502 1.00 0.00 H

ATOM 1912 CG LEU 123 47.019 42.136 48.949 1.00 0.00 C

ATOM 1913 HG LEU 123 46.745 41.478 49.775 1.00 0.00 H

ATOM 1914 CD1 LEU 123 47.221 43.538 49.506 1.00 0.00 C

ATOM 1915 HD11 LEU 123 46.277 43.920 49.888 1.00 0.00 H

ATOM 1916 HD12 LEU 123 47.948 43.519 50.315 1.00 0.00 H

ATOM 1917 HD13 LEU 123 47.574 44.213 48.730 1.00 0.00 H

ATOM 1918 CD2 LEU 123 45.903 42.113 47.916 1.00 0.00 C

ATOM 1919 HD21 LEU 123 44.987 42.509 48.355 1.00 0.00 H

ATOM 1920 HD22 LEU 123 46.172 42.713 47.048 1.00 0.00 H

ATOM 1921 HD23 LEU 123 45.716 41.089 47.605 1.00 0.00 H

ATOM 1922 C LEU 123 49.669 39.787 47.301 1.00 0.00 C

ATOM 1923 O LEU 123 50.539 39.562 48.172 1.00 0.00 O

ATOM 1924 N VAL 124 49.966 39.682 45.988 1.00 0.00 N

ATOM 1925 H VAL 124 49.257 39.912 45.305 1.00 0.00 H

ATOM 1926 CA VAL 124 51.230 39.066 45.489 1.00 0.00 C

ATOM 1927 HA VAL 124 51.972 39.013 46.288 1.00 0.00 H

ATOM 1928 CB VAL 124 51.009 37.654 44.914 1.00 0.00 C

ATOM 1929 HB VAL 124 51.967 37.255 44.576 1.00 0.00 H

ATOM 1930 CG1 VAL 124 50.462 36.722 45.985 1.00 0.00 C

ATOM 1931 HG11 VAL 124 51.111 36.736 46.858 1.00 0.00 H

ATOM 1932 HG12 VAL 124 49.456 37.022 46.282 1.00 0.00 H

ATOM 1933 HG13 VAL 124 50.421 35.703 45.598 1.00 0.00 H

ATOM 1934 CG2 VAL 124 50.064 37.722 43.724 1.00 0.00 C

ATOM 1935 HG21 VAL 124 49.863 36.710 43.370 1.00 0.00 H

ATOM 1936 HG22 VAL 124 49.115 38.176 44.006 1.00 0.00 H

ATOM 1937 HG23 VAL 124 50.511 38.268 42.894 1.00 0.00 H

ATOM 1938 C VAL 124 51.739 39.979 44.415 1.00 0.00 C

ATOM 1939 O VAL 124 51.141 40.740 43.675 1.00 0.00 O

ATOM 1940 N PHE 125 53.073 39.805 44.137 1.00 0.00 N

ATOM 1941 H PHE 125 53.605 39.166 44.712 1.00 0.00 H

ATOM 1942 CA PHE 125 53.765 40.386 43.008 1.00 0.00 C

ATOM 1943 HA PHE 125 53.091 40.386 42.150 1.00 0.00 H

ATOM 1944 CB PHE 125 54.095 41.842 43.343 1.00 0.00 C

ATOM 1945 HB2 PHE 125 54.666 42.275 42.522 1.00 0.00 H

ATOM 1946 HB3 PHE 125 53.169 42.414 43.408 1.00 0.00 H

ATOM 1947 CG PHE 125 54.856 42.011 44.627 1.00 0.00 C

ATOM 1948 CD1 PHE 125 56.241 41.937 44.644 1.00 0.00 C

ATOM 1949 HD1 PHE 125 56.769 41.718 43.740 1.00 0.00 H

ATOM 1950 CE1 PHE 125 56.942 42.092 45.825 1.00 0.00 C

ATOM 1951 HE1 PHE 125 58.021 42.033 45.822 1.00 0.00 H

ATOM 1952 CZ PHE 125 56.266 42.324 47.004 1.00 0.00 C

ATOM 1953 HZ PHE 125 56.828 42.454 47.912 1.00 0.00 H

ATOM 1954 CE2 PHE 125 54.887 42.400 47.002 1.00 0.00 C

ATOM 1955 HE2 PHE 125 54.355 42.527 47.930 1.00 0.00 H

ATOM 1956 CD2 PHE 125 54.189 42.244 45.820 1.00 0.00 C

ATOM 1957 HD2 PHE 125 53.110 42.302 45.834 1.00 0.00 H

ATOM 1958 C PHE 125 55.061 39.664 42.554 1.00 0.00 C

ATOM 1959 O PHE 125 55.608 38.862 43.300 1.00 0.00 O

ATOM 1960 N ALA 126 55.549 39.925 41.344 1.00 0.00 N

ATOM 1961 H ALA 126 55.051 40.631 40.821 1.00 0.00 H

ATOM 1962 CA ALA 126 56.633 39.287 40.580 1.00 0.00 C

ATOM 1963 HA ALA 126 56.744 38.277 40.976 1.00 0.00 H

ATOM 1964 CB ALA 126 56.398 39.147 39.081 1.00 0.00 C

ATOM 1965 HB1 ALA 126 57.201 38.561 38.632 1.00 0.00 H

ATOM 1966 HB2 ALA 126 55.447 38.643 38.902 1.00 0.00 H

ATOM 1967 HB3 ALA 126 56.379 40.135 38.618 1.00 0.00 H

ATOM 1968 C ALA 126 58.000 40.010 40.817 1.00 0.00 C

ATOM 1969 O ALA 126 59.068 39.656 40.375 1.00 0.00 O

ATOM 1970 N HIE 127 57.954 41.020 41.648 1.00 0.00 N

ATOM 1971 H HIE 127 57.069 41.270 42.060 1.00 0.00 H

ATOM 1972 CA HIE 127 59.075 41.943 41.848 1.00 0.00 C

ATOM 1973 HA HIE 127 59.551 42.128 40.883 1.00 0.00 H

ATOM 1974 CB HIE 127 58.602 43.293 42.396 1.00 0.00 C

ATOM 1975 HB2 HIE 127 58.227 43.227 43.413 1.00 0.00 H

ATOM 1976 HB3 HIE 127 59.449 43.982 42.405 1.00 0.00 H

ATOM 1977 CG HIE 127 57.516 43.926 41.582 1.00 0.00 C

ATOM 1978 ND1 HIE 127 57.626 44.124 40.221 1.00 0.00 N

| | | | | | | | | | | |
|---|---|---|---|---|---|---|---|---|---|---|
| ATOM | 1979 | CE1 | HIE | 127 | 56.523 | 44.698 | 39.773 | 1.00 | 0.00 | C |
| ATOM | 1980 | HE1 | HIE | 127 | 56.321 | 44.939 | 38.745 | 1.00 | 0.00 | H |
| ATOM | 1981 | NE2 | HIE | 127 | 55.704 | 44.879 | 40.793 | 1.00 | 0.00 | N |
| ATOM | 1982 | HE2 | HIE | 127 | 54.789 | 45.305 | 40.736 | 1.00 | 0.00 | H |
| ATOM | 1983 | CD2 | HIE | 127 | 56.300 | 44.405 | 41.935 | 1.00 | 0.00 | C |
| ATOM | 1984 | HD2 | HIE | 127 | 55.889 | 44.403 | 42.931 | 1.00 | 0.00 | H |
| ATOM | 1985 | C | HIE | 127 | 60.073 | 41.399 | 42.762 | 1.00 | 0.00 | C |
| ATOM | 1986 | O | HIE | 127 | 60.090 | 41.901 | 43.893 | 1.00 | 0.00 | O |
| ATOM | 1987 | N | THR | 128 | 60.936 | 40.519 | 42.271 | 1.00 | 0.00 | N |
| ATOM | 1988 | H | THR | 128 | 60.806 | 40.222 | 41.312 | 1.00 | 0.00 | H |
| ATOM | 1989 | CA | THR | 128 | 62.048 | 39.923 | 42.962 | 1.00 | 0.00 | C |
| ATOM | 1990 | HA | THR | 128 | 61.709 | 39.608 | 43.948 | 1.00 | 0.00 | H |
| ATOM | 1991 | CB | THR | 128 | 62.546 | 38.682 | 42.199 | 1.00 | 0.00 | C |
| ATOM | 1992 | HB | THR | 128 | 63.420 | 38.274 | 42.707 | 1.00 | 0.00 | H |
| ATOM | 1993 | CG2 | THR | 128 | 61.464 | 37.614 | 42.152 | 1.00 | 0.00 | C |
| ATOM | 1994 | HG21 | THR | 128 | 61.877 | 36.711 | 41.702 | 1.00 | 0.00 | H |
| ATOM | 1995 | HG22 | THR | 128 | 61.127 | 37.372 | 43.158 | 1.00 | 0.00 | H |
| ATOM | 1996 | HG23 | THR | 128 | 60.616 | 37.941 | 41.553 | 1.00 | 0.00 | H |
| ATOM | 1997 | OG1 | THR | 128 | 62.906 | 39.052 | 40.861 | 1.00 | 0.00 | O |
| ATOM | 1998 | HG1 | THR | 128 | 63.315 | 38.297 | 40.433 | 1.00 | 0.00 | H |
| ATOM | 1999 | C | THR | 128 | 63.207 | 40.899 | 43.168 | 1.00 | 0.00 | C |
| ATOM | 2000 | O | THR | 128 | 64.119 | 40.591 | 43.956 | 1.00 | 0.00 | O |
| ATOM | 2001 | N | GLN | 129 | 63.234 | 42.081 | 42.584 | 1.00 | 0.00 | N |
| ATOM | 2002 | H | GLN | 129 | 62.453 | 42.332 | 41.995 | 1.00 | 0.00 | H |
| ATOM | 2003 | CA | GLN | 129 | 64.409 | 42.985 | 42.533 | 1.00 | 0.00 | C |
| ATOM | 2004 | HA | GLN | 129 | 65.308 | 42.373 | 42.450 | 1.00 | 0.00 | H |
| ATOM | 2005 | CB | GLN | 129 | 64.319 | 43.870 | 41.287 | 1.00 | 0.00 | C |
| ATOM | 2006 | HB2 | GLN | 129 | 63.418 | 44.483 | 41.353 | 1.00 | 0.00 | H |
| ATOM | 2007 | HB3 | GLN | 129 | 65.181 | 44.539 | 41.257 | 1.00 | 0.00 | H |
| ATOM | 2008 | CG | GLN | 129 | 64.264 | 43.100 | 39.979 | 1.00 | 0.00 | C |

ATOM 2009 HG2 GLN 129 63.398 42.437 39.965 1.00 0.00 H

ATOM 2010 HG3 GLN 129 64.138 43.818 39.167 1.00 0.00 H

ATOM 2011 CD GLN 129 65.528 42.302 39.723 1.00 0.00 C

ATOM 2012 OE1 GLN 129 65.414 40.980 39.781 1.00 0.00 O

ATOM 2013 NE2 GLN 129 66.597 42.867 39.476 1.00 0.00 N

ATOM 2014 HE21 GLN 129 67.423 42.305 39.339 1.00 0.00 H

ATOM 2015 HE22 GLN 129 66.635 43.872 39.405 1.00 0.00 H

ATOM 2016 C GLN 129 64.566 43.883 43.806 1.00 0.00 C

ATOM 2017 O GLN 129 64.457 45.100 43.715 1.00 0.00 O

ATOM 2018 N GLN 130 64.880 43.262 44.933 1.00 0.00 N

ATOM 2019 H GLN 130 64.942 42.253 44.934 1.00 0.00 H

ATOM 2020 CA GLN 130 65.273 43.962 46.155 1.00 0.00 C

ATOM 2021 HA GLN 130 65.776 44.888 45.871 1.00 0.00 H

ATOM 2022 CB GLN 130 64.020 44.337 46.951 1.00 0.00 C

ATOM 2023 HB2 GLN 130 64.324 44.925 47.819 1.00 0.00 H

ATOM 2024 HB3 GLN 130 63.388 44.970 46.326 1.00 0.00 H

ATOM 2025 CG GLN 130 63.204 43.146 47.424 1.00 0.00 C

ATOM 2026 HG2 GLN 130 62.977 42.501 46.574 1.00 0.00 H

ATOM 2027 HG3 GLN 130 63.776 42.562 48.146 1.00 0.00 H

ATOM 2028 CD GLN 130 61.896 43.561 48.070 1.00 0.00 C

ATOM 2029 OE1 GLN 130 61.775 44.667 48.604 1.00 0.00 O

ATOM 2030 NE2 GLN 130 60.908 42.674 48.026 1.00 0.00 N

ATOM 2031 HE21 GLN 130 61.056 41.774 47.592 1.00 0.00 H

ATOM 2032 HE22 GLN 130 60.009 42.917 48.408 1.00 0.00 H

ATOM 2033 C GLN 130 66.246 43.141 47.053 1.00 0.00 C

ATOM 2034 O GLN 130 66.357 41.963 47.015 1.00 0.00 O

ATOM 2035 N SER 131 67.114 43.904 47.681 1.00 0.00 N

ATOM 2036 H SER 131 67.024 44.904 47.578 1.00 0.00 H

ATOM 2037 CA SER 131 68.238 43.420 48.543 1.00 0.00 C

ATOM 2038 HA SER 131 68.401 42.363 48.327 1.00 0.00 H

ATOM 2039 CB SER 131 69.507 44.147 48.141 1.00 0.00 C
ATOM 2040 HB2 SER 131 70.351 43.760 48.714 1.00 0.00 H
ATOM 2041 HB3 SER 131 69.699 43.973 47.082 1.00 0.00 H
ATOM 2042 OG SER 131 69.387 45.526 48.363 1.00 0.00 O
ATOM 2043 HG SER 131 70.189 45.954 48.053 1.00 0.00 H
ATOM 2044 C SER 131 68.123 43.538 50.046 1.00 0.00 C
ATOM 2045 O SER 131 68.790 42.761 50.790 1.00 0.00 O
ATOM 2046 N ARG 132 67.142 44.278 50.521 1.00 0.00 N
ATOM 2047 H ARG 132 66.559 44.799 49.883 1.00 0.00 H
ATOM 2048 CA ARG 132 67.007 44.533 51.956 1.00 0.00 C
ATOM 2049 HA ARG 132 67.989 44.433 52.423 1.00 0.00 H
ATOM 2050 CB ARG 132 66.550 45.968 52.170 1.00 0.00 C
ATOM 2051 HB2 ARG 132 65.644 46.120 51.584 1.00 0.00 H
ATOM 2052 HB3 ARG 132 66.306 46.121 53.222 1.00 0.00 H
ATOM 2053 CG ARG 132 67.566 47.029 51.777 1.00 0.00 C
ATOM 2054 HG2 ARG 132 68.465 46.917 52.385 1.00 0.00 H
ATOM 2055 HG3 ARG 132 67.840 46.898 50.730 1.00 0.00 H
ATOM 2056 CD ARG 132 67.021 48.400 51.943 1.00 0.00 C
ATOM 2057 HD2 ARG 132 67.755 49.114 51.567 1.00 0.00 H
ATOM 2058 HD3 ARG 132 66.111 48.495 51.349 1.00 0.00 H
ATOM 2059 NE ARG 132 66.740 48.708 53.336 1.00 0.00 N
ATOM 2060 HE ARG 132 67.085 48.063 54.031 1.00 0.00 H
ATOM 2061 CZ ARG 132 66.054 49.787 53.758 1.00 0.00 C
ATOM 2062 NH1 ARG 132 65.585 50.651 52.885 1.00 0.00 N
ATOM 2063 HH11 ARG 132 65.772 50.526 51.902 1.00 0.00 H
ATOM 2064 HH12 ARG 132 65.037 51.439 53.195 1.00 0.00 H
ATOM 2065 NH2 ARG 132 65.851 49.978 55.050 1.00 0.00 N
ATOM 2066 HH21 ARG 132 65.364 50.802 55.370 1.00 0.00 H
ATOM 2067 HH22 ARG 132 66.170 49.294 55.720 1.00 0.00 H
ATOM 2068 C ARG 132 66.084 43.657 52.739 1.00 0.00 C

| | | | | | | | | | | |
|---|---|---|---|---|---|---|---|---|---|---|
| ATOM | 2069 | O | ARG | 132 | 65.917 | 43.822 | 54.002 | 1.00 | 0.00 | O |
| ATOM | 2070 | N | GLN | 133 | 65.539 | 42.655 | 52.089 | 1.00 | 0.00 | N |
| ATOM | 2071 | H | GLN | 133 | 65.794 | 42.578 | 51.114 | 1.00 | 0.00 | H |
| ATOM | 2072 | CA | GLN | 133 | 64.588 | 41.593 | 52.537 | 1.00 | 0.00 | C |
| ATOM | 2073 | HA | GLN | 133 | 64.972 | 41.181 | 53.470 | 1.00 | 0.00 | H |
| ATOM | 2074 | CB | GLN | 133 | 63.186 | 42.160 | 52.776 | 1.00 | 0.00 | C |
| ATOM | 2075 | HB2 | GLN | 133 | 62.597 | 41.449 | 53.347 | 1.00 | 0.00 | H |
| ATOM | 2076 | HB3 | GLN | 133 | 63.259 | 43.053 | 53.399 | 1.00 | 0.00 | H |
| ATOM | 2077 | CG | GLN | 133 | 62.433 | 42.514 | 51.505 | 1.00 | 0.00 | C |
| ATOM | 2078 | HG2 | GLN | 133 | 62.742 | 43.506 | 51.179 | 1.00 | 0.00 | H |
| ATOM | 2079 | HG3 | GLN | 133 | 62.664 | 41.839 | 50.687 | 1.00 | 0.00 | H |
| ATOM | 2080 | CD | GLN | 133 | 60.929 | 42.498 | 51.700 | 1.00 | 0.00 | C |
| ATOM | 2081 | OE1 | GLN | 133 | 60.356 | 43.414 | 52.297 | 1.00 | 0.00 | O |
| ATOM | 2082 | NE2 | GLN | 133 | 60.279 | 41.454 | 51.198 | 1.00 | 0.00 | N |
| ATOM | 2083 | HE21 | GLN | 133 | 60.790 | 40.736 | 50.701 | 1.00 | 0.00 | H |
| ATOM | 2084 | HE22 | GLN | 133 | 59.282 | 41.380 | 51.320 | 1.00 | 0.00 | H |
| ATOM | 2085 | C | GLN | 133 | 64.569 | 40.485 | 51.417 | 1.00 | 0.00 | C |
| ATOM | 2086 | O | GLN | 133 | 65.128 | 40.743 | 50.312 | 1.00 | 0.00 | O |
| ATOM | 2087 | N | PRO | 134 | 64.036 | 39.312 | 51.698 | 1.00 | 0.00 | N |
| ATOM | 2088 | CD | PRO | 134 | 63.477 | 38.995 | 53.034 | 1.00 | 0.00 | C |
| ATOM | 2089 | HD2 | PRO | 134 | 62.463 | 39.383 | 53.121 | 1.00 | 0.00 | H |
| ATOM | 2090 | HD3 | PRO | 134 | 64.111 | 39.320 | 53.858 | 1.00 | 0.00 | H |
| ATOM | 2091 | CG | PRO | 134 | 63.413 | 37.491 | 53.000 | 1.00 | 0.00 | C |
| ATOM | 2092 | HG2 | PRO | 134 | 62.648 | 37.102 | 53.651 | 1.00 | 0.00 | H |
| ATOM | 2093 | HG3 | PRO | 134 | 64.387 | 37.074 | 53.262 | 1.00 | 0.00 | H |
| ATOM | 2094 | CB | PRO | 134 | 63.088 | 37.167 | 51.581 | 1.00 | 0.00 | C |
| ATOM | 2095 | HB2 | PRO | 134 | 62.019 | 37.312 | 51.413 | 1.00 | 0.00 | H |
| ATOM | 2096 | HB3 | PRO | 134 | 63.375 | 36.146 | 51.333 | 1.00 | 0.00 | H |
| ATOM | 2097 | CA | PRO | 134 | 63.896 | 38.184 | 50.769 | 1.00 | 0.00 | C |
| ATOM | 2098 | HA | PRO | 134 | 64.879 | 37.784 | 50.518 | 1.00 | 0.00 | H |

```
ATOM   2099  C   PRO   134      63.135  38.656  49.483  1.00  0.00           C
ATOM   2100  O   PRO   134      62.273  39.508  49.623  1.00  0.00           O
ATOM   2101  N   HID   135      63.410  38.167  48.299  1.00  0.00           N
ATOM   2102  H   HID   135      64.126  37.456  48.264  1.00  0.00           H
ATOM   2103  CA  HID   135      62.859  38.551  46.983  1.00  0.00           C
ATOM   2104  HA  HID   135      63.514  39.317  46.564  1.00  0.00           H
ATOM   2105  CB  HID   135      62.876  37.343  46.042  1.00  0.00           C
ATOM   2106  HB2 HID   135      62.358  36.503  46.507  1.00  0.00           H
ATOM   2107  HB3 HID   135      62.339  37.601  45.130  1.00  0.00           H
ATOM   2108  CG  HID   135      64.253  36.911  45.643  1.00  0.00           C
ATOM   2109  ND1 HID   135      65.120  37.734  44.954  1.00  0.00           N
ATOM   2110  HD1 HID   135      64.920  38.679  44.655  1.00  0.00           H
ATOM   2111  CE1 HID   135      66.255  37.090  44.740  1.00  0.00           C
ATOM   2112  HE1 HID   135      67.118  37.483  44.221  1.00  0.00           H
ATOM   2113  NE2 HID   135      66.154  35.883  45.264  1.00  0.00           N
ATOM   2114  CD2 HID   135      64.913  35.745  45.835  1.00  0.00           C
ATOM   2115  HD2 HID   135      64.533  34.869  46.341  1.00  0.00           H
ATOM   2116  C   HID   135      61.460  39.118  46.981  1.00  0.00           C
ATOM   2117  O   HID   135      61.241  40.239  46.656  1.00  0.00           O
ATOM   2118  N   LEU   136      60.447  38.423  47.518  1.00  0.00           N
ATOM   2119  H   LEU   136      60.642  37.516  47.919  1.00  0.00           H
ATOM   2120  CA  LEU   136      58.992  38.819  47.376  1.00  0.00           C
ATOM   2121  HA  LEU   136      58.909  39.760  46.833  1.00  0.00           H
ATOM   2122  CB  LEU   136      58.209  37.750  46.603  1.00  0.00           C
ATOM   2123  HB2 LEU   136      58.226  36.834  47.192  1.00  0.00           H
ATOM   2124  HB3 LEU   136      57.168  38.068  46.524  1.00  0.00           H
ATOM   2125  CG  LEU   136      58.731  37.429  45.196  1.00  0.00           C
ATOM   2126  HG  LEU   136      59.759  37.073  45.256  1.00  0.00           H
ATOM   2127  CD1 LEU   136      57.887  36.321  44.581  1.00  0.00           C
ATOM   2128 HD11 LEU   136      58.266  36.081  43.587  1.00  0.00           H
```

ATOM 2129 HD12 LEU 136 57.940 35.425 45.200 1.00 0.00 H
ATOM 2130 HD13 LEU 136 56.847 36.640 44.498 1.00 0.00 H
ATOM 2131 CD2 LEU 136 58.690 38.687 44.341 1.00 0.00 C
ATOM 2132 HD21 LEU 136 58.821 38.437 43.289 1.00 0.00 H
ATOM 2133 HD22 LEU 136 57.746 39.209 44.473 1.00 0.00 H
ATOM 2134 HD23 LEU 136 59.493 39.354 44.645 1.00 0.00 H
ATOM 2135 C LEU 136 58.336 39.028 48.752 1.00 0.00 C
ATOM 2136 O LEU 136 58.711 38.464 49.791 1.00 0.00 O
ATOM 2137 N MET 137 57.154 39.765 48.735 1.00 0.00 N
ATOM 2138 H MET 137 56.799 40.170 47.880 1.00 0.00 H
ATOM 2139 CA MET 137 56.385 39.899 49.982 1.00 0.00 C
ATOM 2140 HA MET 137 56.769 39.215 50.723 1.00 0.00 H
ATOM 2141 CB MET 137 56.514 41.309 50.555 1.00 0.00 C
ATOM 2142 HB2 MET 137 57.575 41.523 50.675 1.00 0.00 H
ATOM 2143 HB3 MET 137 56.109 42.031 49.856 1.00 0.00 H
ATOM 2144 CG MET 137 55.854 41.499 51.913 1.00 0.00 C
ATOM 2145 HG2 MET 137 55.996 40.602 52.516 1.00 0.00 H
ATOM 2146 HG3 MET 137 56.335 42.333 52.424 1.00 0.00 H
ATOM 2147 SD MET 137 54.092 41.863 51.785 1.00 0.00 S
ATOM 2148 CE MET 137 54.137 43.528 51.128 1.00 0.00 C
ATOM 2149 HE1 MET 137 53.121 43.901 51.010 1.00 0.00 H
ATOM 2150 HE2 MET 137 54.678 44.178 51.816 1.00 0.00 H
ATOM 2151 HE3 MET 137 54.630 43.533 50.161 1.00 0.00 H
ATOM 2152 C MET 137 54.917 39.553 49.693 1.00 0.00 C
ATOM 2153 O MET 137 54.329 40.005 48.732 1.00 0.00 O
ATOM 2154 N VAL 138 54.219 38.829 50.553 1.00 0.00 N
ATOM 2155 H VAL 138 54.670 38.515 51.396 1.00 0.00 H
ATOM 2156 CA VAL 138 52.782 38.488 50.402 1.00 0.00 C
ATOM 2157 HA VAL 138 52.390 38.938 49.495 1.00 0.00 H
ATOM 2158 CB VAL 138 52.534 36.969 50.331 1.00 0.00 C

ATOM 2159 HB VAL 138 52.920 36.496 51.235 1.00 0.00 H

ATOM 2160 CG1 VAL 138 51.045 36.677 50.232 1.00 0.00 C

ATOM 2161 HG11 VAL 138 50.898 35.606 50.092 1.00 0.00 H

ATOM 2162 HG12 VAL 138 50.525 36.966 51.144 1.00 0.00 H

ATOM 2163 HG13 VAL 138 50.611 37.201 49.381 1.00 0.00 H

ATOM 2164 CG2 VAL 138 53.280 36.378 49.144 1.00 0.00 C

ATOM 2165 HG21 VAL 138 53.094 35.305 49.086 1.00 0.00 H

ATOM 2166 HG22 VAL 138 52.945 36.851 48.222 1.00 0.00 H

ATOM 2167 HG23 VAL 138 54.349 36.543 49.248 1.00 0.00 H

ATOM 2168 C VAL 138 52.092 39.023 51.558 1.00 0.00 C

ATOM 2169 O VAL 138 52.360 38.657 52.678 1.00 0.00 O

ATOM 2170 N VAL 139 51.044 39.791 51.276 1.00 0.00 N

ATOM 2171 H VAL 139 50.809 40.019 50.319 1.00 0.00 H

ATOM 2172 CA VAL 139 50.200 40.272 52.381 1.00 0.00 C

ATOM 2173 HA VAL 139 50.814 40.445 53.267 1.00 0.00 H

ATOM 2174 CB VAL 139 49.548 41.613 51.997 1.00 0.00 C

ATOM 2175 HB VAL 139 48.915 41.454 51.131 1.00 0.00 H

ATOM 2176 CG1 VAL 139 48.659 42.115 53.125 1.00 0.00 C

ATOM 2177 HG11 VAL 139 48.208 43.062 52.848 1.00 0.00 H

ATOM 2178 HG12 VAL 139 47.850 41.414 53.305 1.00 0.00 H

ATOM 2179 HG13 VAL 139 49.242 42.242 54.038 1.00 0.00 H

ATOM 2180 CG2 VAL 139 50.623 42.636 51.662 1.00 0.00 C

ATOM 2181 HG21 VAL 139 50.168 43.591 51.416 1.00 0.00 H

ATOM 2182 HG22 VAL 139 51.289 42.762 52.513 1.00 0.00 H

ATOM 2183 HG23 VAL 139 51.198 42.317 50.793 1.00 0.00 H

ATOM 2184 C VAL 139 49.089 39.214 52.733 1.00 0.00 C

ATOM 2185 O VAL 139 48.266 38.830 51.888 1.00 0.00 O

ATOM 2186 N VAL 140 49.120 38.678 53.916 1.00 0.00 N

ATOM 2187 H VAL 140 49.824 39.012 54.551 1.00 0.00 H

ATOM 2188 CA VAL 140 48.265 37.582 54.399 1.00 0.00 C

ATOM 2189 HA VAL 140 47.799 37.081 53.550 1.00 0.00 H
ATOM 2190 CB VAL 140 49.135 36.546 55.136 1.00 0.00 C
ATOM 2191 HB VAL 140 49.602 37.011 55.994 1.00 0.00 H
ATOM 2192 CG1 VAL 140 48.280 35.390 55.633 1.00 0.00 C
ATOM 2193 HG11 VAL 140 48.923 34.684 56.154 1.00 0.00 H
ATOM 2194 HG12 VAL 140 47.525 35.730 56.341 1.00 0.00 H
ATOM 2195 HG13 VAL 140 47.793 34.885 54.797 1.00 0.00 H
ATOM 2196 CG2 VAL 140 50.237 36.046 54.215 1.00 0.00 C
ATOM 2197 HG21 VAL 140 50.786 35.252 54.719 1.00 0.00 H
ATOM 2198 HG22 VAL 140 49.821 35.641 53.298 1.00 0.00 H
ATOM 2199 HG23 VAL 140 50.942 36.841 53.980 1.00 0.00 H
ATOM 2200 C VAL 140 47.112 38.095 55.359 1.00 0.00 C
ATOM 2201 O VAL 140 47.304 38.548 56.440 1.00 0.00 O
ATOM 2202 N VAL 141 45.858 38.079 54.906 1.00 0.00 N
ATOM 2203 H VAL 141 45.698 37.692 53.985 1.00 0.00 H
ATOM 2204 CA VAL 141 44.684 38.603 55.592 1.00 0.00 C
ATOM 2205 HA VAL 141 44.957 38.868 56.614 1.00 0.00 H
ATOM 2206 CB VAL 141 44.224 39.893 54.886 1.00 0.00 C
ATOM 2207 HB VAL 141 44.105 39.705 53.823 1.00 0.00 H
ATOM 2208 CG1 VAL 141 42.880 40.348 55.433 1.00 0.00 C
ATOM 2209 HG11 VAL 141 42.603 41.285 54.957 1.00 0.00 H
ATOM 2210 HG12 VAL 141 42.084 39.650 55.174 1.00 0.00 H
ATOM 2211 HG13 VAL 141 42.929 40.479 56.515 1.00 0.00 H
ATOM 2212 CG2 VAL 141 45.273 40.980 55.059 1.00 0.00 C
ATOM 2213 HG21 VAL 141 44.889 41.939 54.738 1.00 0.00 H
ATOM 2214 HG22 VAL 141 45.545 41.060 56.110 1.00 0.00 H
ATOM 2215 HG23 VAL 141 46.156 40.736 54.476 1.00 0.00 H
ATOM 2216 C VAL 141 43.496 37.676 55.687 1.00 0.00 C
ATOM 2217 O VAL 141 43.148 36.927 54.742 1.00 0.00 O
ATOM 2218 N GLN 142 42.899 37.568 56.882 1.00 0.00 N

ATOM 2219 H GLN 142 43.214 38.155 57.640 1.00 0.00 H

ATOM 2220 CA GLN 142 41.808 36.666 57.156 1.00 0.00 C

ATOM 2221 HA GLN 142 41.844 35.820 56.468 1.00 0.00 H

ATOM 2222 CB GLN 142 41.917 36.115 58.580 1.00 0.00 C

ATOM 2223 HB2 GLN 142 41.806 36.942 59.280 1.00 0.00 H

ATOM 2224 HB3 GLN 142 41.104 35.412 58.753 1.00 0.00 H

ATOM 2225 CG GLN 142 43.247 35.450 58.890 1.00 0.00 C

ATOM 2226 HG2 GLN 142 44.032 36.207 58.915 1.00 0.00 H

ATOM 2227 HG3 GLN 142 43.192 35.000 59.879 1.00 0.00 H

ATOM 2228 CD GLN 142 43.601 34.368 57.888 1.00 0.00 C

ATOM 2229 OE1 GLN 142 42.830 33.429 57.669 1.00 0.00 O

ATOM 2230 NE2 GLN 142 44.772 34.492 57.274 1.00 0.00 N

ATOM 2231 HE21 GLN 142 45.383 35.267 57.471 1.00 0.00 H

ATOM 2232 HE22 GLN 142 45.047 33.784 56.602 1.00 0.00 H

ATOM 2233 C GLN 142 40.462 37.389 56.952 1.00 0.00 C

ATOM 2234 O GLN 142 40.389 38.590 57.254 1.00 0.00 O

ATOM 2235 N LEU 143 39.411 36.715 56.561 1.00 0.00 N

ATOM 2236 H LEU 143 39.496 35.724 56.398 1.00 0.00 H

ATOM 2237 CA LEU 143 38.077 37.287 56.543 1.00 0.00 C

ATOM 2238 HA LEU 143 38.086 38.333 56.849 1.00 0.00 H

ATOM 2239 CB LEU 143 37.456 37.199 55.144 1.00 0.00 C

ATOM 2240 HB2 LEU 143 37.282 36.147 54.937 1.00 0.00 H

ATOM 2241 HB3 LEU 143 36.486 37.698 55.165 1.00 0.00 H

ATOM 2242 CG LEU 143 38.303 37.776 54.002 1.00 0.00 C

ATOM 2243 HG LEU 143 39.267 37.268 53.960 1.00 0.00 H

ATOM 2244 CD1 LEU 143 37.595 37.541 52.675 1.00 0.00 C

ATOM 2245 HD11 LEU 143 38.214 37.915 51.861 1.00 0.00 H

ATOM 2246 HD12 LEU 143 37.434 36.475 52.522 1.00 0.00 H

ATOM 2247 HD13 LEU 143 36.638 38.060 52.659 1.00 0.00 H

ATOM 2248 CD2 LEU 143 38.536 39.260 54.243 1.00 0.00 C

ATOM 2249 HD21 LEU 143 39.019 39.700 53.375 1.00 0.00 H

ATOM 2250 HD22 LEU 143 37.584 39.765 54.403 1.00 0.00 H

ATOM 2251 HD23 LEU 143 39.189 39.406 55.100 1.00 0.00 H

ATOM 2252 C LEU 143 37.248 36.486 57.583 1.00 0.00 C

ATOM 2253 O LEU 143 37.440 35.241 57.654 1.00 0.00 O

ATOM 2254 N VAL 144 36.373 37.197 58.326 1.00 0.00 N

ATOM 2255 H VAL 144 36.307 38.196 58.185 1.00 0.00 H

ATOM 2256 CA VAL 144 35.448 36.600 59.236 1.00 0.00 C

ATOM 2257 HA VAL 144 35.562 35.516 59.212 1.00 0.00 H

ATOM 2258 CB VAL 144 35.765 37.077 60.665 1.00 0.00 C

ATOM 2259 HB VAL 144 36.793 36.802 60.902 1.00 0.00 H

ATOM 2260 CG1 VAL 144 35.618 38.588 60.768 1.00 0.00 C

ATOM 2261 HG11 VAL 144 35.863 38.889 61.786 1.00 0.00 H

ATOM 2262 HG12 VAL 144 36.313 39.097 60.113 1.00 0.00 H

ATOM 2263 HG13 VAL 144 34.599 38.920 60.581 1.00 0.00 H

ATOM 2264 CG2 VAL 144 34.851 36.378 61.659 1.00 0.00 C

ATOM 2265 HG21 VAL 144 35.175 36.613 62.673 1.00 0.00 H

ATOM 2266 HG22 VAL 144 33.819 36.708 61.545 1.00 0.00 H

ATOM 2267 HG23 VAL 144 34.904 35.297 61.521 1.00 0.00 H

ATOM 2268 C VAL 144 33.976 36.940 58.875 1.00 0.00 C

ATOM 2269 O VAL 144 33.655 38.120 58.493 1.00 0.00 O

ATOM 2270 N ALA 145 33.068 35.963 58.925 1.00 0.00 N

ATOM 2271 H ALA 145 33.399 35.059 59.226 1.00 0.00 H

ATOM 2272 CA ALA 145 31.615 36.042 58.685 1.00 0.00 C

ATOM 2273 HA ALA 145 31.452 36.465 57.695 1.00 0.00 H

ATOM 2274 CB ALA 145 31.080 34.617 58.685 1.00 0.00 C

ATOM 2275 HB1 ALA 145 30.006 34.623 58.520 1.00 0.00 H

ATOM 2276 HB2 ALA 145 31.562 34.040 57.895 1.00 0.00 H

ATOM 2277 HB3 ALA 145 31.284 34.145 59.647 1.00 0.00 H

ATOM 2278 C ALA 145 30.917 36.954 59.687 1.00 0.00 C

```
ATOM   2279  O   ALA   145      31.151  36.747  60.873  1.00  0.00           O
ATOM   2280  N   SER   146      29.939  37.852  59.320  1.00  0.00           N
ATOM   2281  H   SER   146      29.741  37.991  58.340  1.00  0.00           H
ATOM   2282  CA  SER   146      29.172  38.661  60.312  1.00  0.00           C
ATOM   2283  HA  SER   146      29.020  38.062  61.211  1.00  0.00           H
ATOM   2284  CB  SER   146      29.963  39.898  60.691  1.00  0.00           C
ATOM   2285  HB2 SER   146      30.994  39.619  60.911  1.00  0.00           H
ATOM   2286  HB3 SER   146      29.960  40.601  59.857  1.00  0.00           H
ATOM   2287  OG  SER   146      29.419  40.513  61.827  1.00  0.00           O
ATOM   2288  HG  SER   146      29.624  39.971  62.594  1.00  0.00           H
ATOM   2289  C   SER   146      27.783  39.084  59.788  1.00  0.00           C
ATOM   2290  O   SER   146      27.290  38.678  58.731  1.00  0.00           O
ATOM   2291  N   ASP   147      27.192  39.982  60.510  1.00  0.00           N
ATOM   2292  H   ASP   147      27.622  40.336  61.353  1.00  0.00           H
ATOM   2293  CA  ASP   147      25.845  40.515  60.133  1.00  0.00           C
ATOM   2294  HA  ASP   147      25.290  39.727  59.624  1.00  0.00           H
ATOM   2295  CB  ASP   147      25.071  40.833  61.415  1.00  0.00           C
ATOM   2296  HB2 ASP   147      24.057  41.160  61.179  1.00  0.00           H
ATOM   2297  HB3 ASP   147      24.982  39.914  61.997  1.00  0.00           H
ATOM   2298  CG  ASP   147      25.765  41.874  62.283  1.00  0.00           C
ATOM   2299  OD1 ASP   147      26.935  42.101  62.084  1.00  0.00           O
ATOM   2300  OD2 ASP   147      25.118  42.434  63.135  1.00  0.00           O
ATOM   2301  C   ASP   147      25.790  41.778  59.205  1.00  0.00           C
ATOM   2302  O   ASP   147      24.777  42.500  59.197  1.00  0.00           O
ATOM   2303  N   LYS   148      26.951  42.207  58.758  1.00  0.00           N
ATOM   2304  H   LYS   148      27.758  41.631  58.953  1.00  0.00           H
ATOM   2305  CA  LYS   148      27.206  43.393  58.008  1.00  0.00           C
ATOM   2306  HA  LYS   148      26.348  43.579  57.361  1.00  0.00           H
ATOM   2307  CB  LYS   148      27.335  44.576  58.969  1.00  0.00           C
ATOM   2308  HB2 LYS   148      27.470  45.489  58.387  1.00  0.00           H
```

```
ATOM   2309  HB3 LYS   148      26.394  44.677  59.511  1.00  0.00           H
ATOM   2310  CG  LYS   148      28.476  44.452  59.969  1.00  0.00           C
ATOM   2311  HG2 LYS   148      28.368  43.561  60.583  1.00  0.00           H
ATOM   2312  HG3 LYS   148      29.413  44.365  59.420  1.00  0.00           H
ATOM   2313  CD  LYS   148      28.584  45.694  60.842  1.00  0.00           C
ATOM   2314  HD2 LYS   148      29.531  45.671  61.383  1.00  0.00           H
ATOM   2315  HD3 LYS   148      28.573  46.586  60.214  1.00  0.00           H
ATOM   2316  CE  LYS   148      27.441  45.768  61.843  1.00  0.00           C
ATOM   2317  HE2 LYS   148      27.467  46.745  62.327  1.00  0.00           H
ATOM   2318  HE3 LYS   148      26.488  45.667  61.322  1.00  0.00           H
ATOM   2319  NZ  LYS   148      27.549  44.716  62.890  1.00  0.00           N
ATOM   2320  HZ1 LYS   148      26.788  44.804  63.548  1.00  0.00           H
ATOM   2321  HZ2 LYS   148      27.482  43.798  62.467  1.00  0.00           H
ATOM   2322  HZ3 LYS   148      28.430  44.794  63.375  1.00  0.00           H
ATOM   2323  C   LYS   148      28.447  43.300  57.115  1.00  0.00           C
ATOM   2324  O   LYS   148      29.381  42.552  57.434  1.00  0.00           O
ATOM   2325  N   ILE   149      28.622  44.321  56.202  1.00  0.00           N
ATOM   2326  H   ILE   149      27.915  45.028  56.068  1.00  0.00           H
ATOM   2327  CA  ILE   149      29.782  44.277  55.277  1.00  0.00           C
ATOM   2328  HA  ILE   149      30.578  43.679  55.721  1.00  0.00           H
ATOM   2329  CB  ILE   149      29.381  43.620  53.943  1.00  0.00           C
ATOM   2330  HB  ILE   149      28.978  42.632  54.158  1.00  0.00           H
ATOM   2331  CG2 ILE   149      28.308  44.444  53.247  1.00  0.00           C
ATOM   2332 HG21 ILE   149      27.998  43.960  52.322  1.00  0.00           H
ATOM   2333 HG22 ILE   149      27.424  44.525  53.880  1.00  0.00           H
ATOM   2334 HG23 ILE   149      28.663  45.447  53.022  1.00  0.00           H
ATOM   2335  CG1 ILE   149      30.605  43.455  53.040  1.00  0.00           C
ATOM   2336 HG12 ILE   149      30.938  44.422  52.664  1.00  0.00           H
ATOM   2337 HG13 ILE   149      31.418  43.031  53.628  1.00  0.00           H
ATOM   2338  CD1 ILE   149      30.375  42.538  51.860  1.00  0.00           C
```

```
ATOM   2339 HD11 ILE   149      31.319  42.383  51.341  1.00  0.00           H
ATOM   2340 HD12 ILE   149      29.997  41.575  52.205  1.00  0.00           H
ATOM   2341 HD13 ILE   149      29.663  42.971  51.160  1.00  0.00           H
ATOM   2342  C   ILE   149      30.375  45.696  54.998  1.00  0.00           C
ATOM   2343  O   ILE   149      29.653  46.680  55.176  1.00  0.00           O
ATOM   2344  N   ASN   150      31.755  45.773  54.884  1.00  0.00           N
ATOM   2345  H   ASN   150      32.266  44.902  54.906  1.00  0.00           H
ATOM   2346  CA  ASN   150      32.563  46.973  54.612  1.00  0.00           C
ATOM   2347  HA  ASN   150      32.343  47.688  55.407  1.00  0.00           H
ATOM   2348  CB  ASN   150      34.035  46.620  54.714  1.00  0.00           C
ATOM   2349  HB2 ASN   150      34.196  46.073  55.646  1.00  0.00           H
ATOM   2350  HB3 ASN   150      34.321  45.962  53.892  1.00  0.00           H
ATOM   2351  CG  ASN   150      34.923  47.833  54.698  1.00  0.00           C
ATOM   2352  OD1 ASN   150      34.696  48.774  53.927  1.00  0.00           O
ATOM   2353  ND2 ASN   150      35.930  47.831  55.533  1.00  0.00           N
ATOM   2354 HD21 ASN   150      36.093  47.037  56.135  1.00  0.00           H
ATOM   2355 HD22 ASN   150      36.571  48.610  55.532  1.00  0.00           H
ATOM   2356  C   ASN   150      32.289  47.662  53.260  1.00  0.00           C
ATOM   2357  O   ASN   150      32.417  47.013  52.207  1.00  0.00           O
ATOM   2358  N   SER   151      31.846  48.903  53.300  1.00  0.00           N
ATOM   2359  H   SER   151      31.776  49.379  54.188  1.00  0.00           H
ATOM   2360  CA  SER   151      31.238  49.577  52.089  1.00  0.00           C
ATOM   2361  HA  SER   151      30.370  48.999  51.774  1.00  0.00           H
ATOM   2362  CB  SER   151      30.767  50.973  52.445  1.00  0.00           C
ATOM   2363  HB2 SER   151      30.228  51.401  51.600  1.00  0.00           H
ATOM   2364  HB3 SER   151      30.091  50.918  53.299  1.00  0.00           H
ATOM   2365  OG  SER   151      31.850  51.802  52.766  1.00  0.00           O
ATOM   2366  HG  SER   151      32.317  52.021  51.956  1.00  0.00           H
ATOM   2367  C   SER   151      32.222  49.668  50.905  1.00  0.00           C
ATOM   2368  O   SER   151      31.756  49.831  49.755  1.00  0.00           O
```

ATOM 2369 N THR 152 33.537 49.584 51.229 1.00 0.00 N
ATOM 2370 H THR 152 33.756 49.451 52.207 1.00 0.00 H
ATOM 2371 CA THR 152 34.715 49.676 50.318 1.00 0.00 C
ATOM 2372 HA THR 152 34.546 50.521 49.649 1.00 0.00 H
ATOM 2373 CB THR 152 36.010 49.946 51.107 1.00 0.00 C
ATOM 2374 HB THR 152 36.844 50.078 50.417 1.00 0.00 H
ATOM 2375 CG2 THR 152 35.872 51.211 51.941 1.00 0.00 C
ATOM 2376 HG21 THR 152 36.826 51.424 52.420 1.00 0.00 H
ATOM 2377 HG22 THR 152 35.605 52.048 51.297 1.00 0.00 H
ATOM 2378 HG23 THR 152 35.112 51.100 52.714 1.00 0.00 H
ATOM 2379 OG1 THR 152 36.286 48.836 51.971 1.00 0.00 O
ATOM 2380 HG1 THR 152 35.626 48.822 52.675 1.00 0.00 H
ATOM 2381 C THR 152 34.955 48.484 49.474 1.00 0.00 C
ATOM 2382 O THR 152 35.830 48.678 48.616 1.00 0.00 O
ATOM 2383 N SER 153 34.261 47.358 49.651 1.00 0.00 N
ATOM 2384 H SER 153 33.603 47.308 50.417 1.00 0.00 H
ATOM 2385 CA SER 153 34.369 46.170 48.770 1.00 0.00 C
ATOM 2386 HA SER 153 34.960 46.441 47.899 1.00 0.00 H
ATOM 2387 CB SER 153 35.105 45.071 49.511 1.00 0.00 C
ATOM 2388 HB2 SER 153 35.240 44.207 48.861 1.00 0.00 H
ATOM 2389 HB3 SER 153 36.088 45.440 49.805 1.00 0.00 H
ATOM 2390 OG SER 153 34.401 44.681 50.659 1.00 0.00 O
ATOM 2391 HG SER 153 34.922 44.022 51.125 1.00 0.00 H
ATOM 2392 C SER 153 33.040 45.599 48.245 1.00 0.00 C
ATOM 2393 O SER 153 33.086 44.647 47.532 1.00 0.00 O
ATOM 2394 N VAL 154 31.845 46.137 48.669 1.00 0.00 N
ATOM 2395 H VAL 154 31.859 46.937 49.285 1.00 0.00 H
ATOM 2396 CA VAL 154 30.568 45.505 48.411 1.00 0.00 C
ATOM 2397 HA VAL 154 30.573 44.518 48.876 1.00 0.00 H
ATOM 2398 CB VAL 154 29.461 46.356 49.060 1.00 0.00 C

| | | | | | | | | | | |
|---|---|---|---|---|---|---|---|---|---|---|
| ATOM | 2399 | HB | VAL | 154 | 29.553 | 47.390 | 48.722 | 1.00 | 0.00 | H |
| ATOM | 2400 | CG1 | VAL | 154 | 28.088 | 45.853 | 48.642 | 1.00 | 0.00 | C |
| ATOM | 2401 | HG11 | VAL | 154 | 27.320 | 46.372 | 49.216 | 1.00 | 0.00 | H |
| ATOM | 2402 | HG12 | VAL | 154 | 27.902 | 46.060 | 47.588 | 1.00 | 0.00 | H |
| ATOM | 2403 | HG13 | VAL | 154 | 28.011 | 44.783 | 48.834 | 1.00 | 0.00 | H |
| ATOM | 2404 | CG2 | VAL | 154 | 29.607 | 46.329 | 50.574 | 1.00 | 0.00 | C |
| ATOM | 2405 | HG21 | VAL | 154 | 28.752 | 46.822 | 51.037 | 1.00 | 0.00 | H |
| ATOM | 2406 | HG22 | VAL | 154 | 29.679 | 45.304 | 50.925 | 1.00 | 0.00 | H |
| ATOM | 2407 | HG23 | VAL | 154 | 30.493 | 46.884 | 50.848 | 1.00 | 0.00 | H |
| ATOM | 2408 | C | VAL | 154 | 30.266 | 45.308 | 46.861 | 1.00 | 0.00 | C |
| ATOM | 2409 | O | VAL | 154 | 29.863 | 44.217 | 46.469 | 1.00 | 0.00 | O |
| ATOM | 2410 | N | ALA | 155 | 30.453 | 46.311 | 45.979 | 1.00 | 0.00 | N |
| ATOM | 2411 | H | ALA | 155 | 30.815 | 47.189 | 46.320 | 1.00 | 0.00 | H |
| ATOM | 2412 | CA | ALA | 155 | 30.076 | 46.265 | 44.551 | 1.00 | 0.00 | C |
| ATOM | 2413 | HA | ALA | 155 | 28.998 | 46.108 | 44.493 | 1.00 | 0.00 | H |
| ATOM | 2414 | CB | ALA | 155 | 30.399 | 47.571 | 43.836 | 1.00 | 0.00 | C |
| ATOM | 2415 | HB1 | ALA | 155 | 29.908 | 47.585 | 42.868 | 1.00 | 0.00 | H |
| ATOM | 2416 | HB2 | ALA | 155 | 30.012 | 48.410 | 44.416 | 1.00 | 0.00 | H |
| ATOM | 2417 | HB3 | ALA | 155 | 31.470 | 47.701 | 43.709 | 1.00 | 0.00 | H |
| ATOM | 2418 | C | ALA | 155 | 30.777 | 45.142 | 43.760 | 1.00 | 0.00 | C |
| ATOM | 2419 | O | ALA | 155 | 30.112 | 44.263 | 43.284 | 1.00 | 0.00 | O |
| ATOM | 2420 | N | ALA | 156 | 32.133 | 45.086 | 43.872 | 1.00 | 0.00 | N |
| ATOM | 2421 | H | ALA | 156 | 32.648 | 45.827 | 44.326 | 1.00 | 0.00 | H |
| ATOM | 2422 | CA | ALA | 156 | 32.834 | 44.029 | 43.257 | 1.00 | 0.00 | C |
| ATOM | 2423 | HA | ALA | 156 | 32.596 | 44.030 | 42.194 | 1.00 | 0.00 | H |
| ATOM | 2424 | CB | ALA | 156 | 34.304 | 44.369 | 43.386 | 1.00 | 0.00 | C |
| ATOM | 2425 | HB1 | ALA | 156 | 34.902 | 43.545 | 42.994 | 1.00 | 0.00 | H |
| ATOM | 2426 | HB2 | ALA | 156 | 34.556 | 45.257 | 42.819 | 1.00 | 0.00 | H |
| ATOM | 2427 | HB3 | ALA | 156 | 34.542 | 44.492 | 44.439 | 1.00 | 0.00 | H |
| ATOM | 2428 | C | ALA | 156 | 32.553 | 42.619 | 43.781 | 1.00 | 0.00 | C |

```
ATOM   2429  O   ALA   156      32.442  41.652  43.014  1.00  0.00           O
ATOM   2430  N   LEU   157      32.424  42.513  45.092  1.00  0.00           N
ATOM   2431  H   LEU   157      32.518  43.322  45.690  1.00  0.00           H
ATOM   2432  CA  LEU   157      32.286  41.160  45.701  1.00  0.00           C
ATOM   2433  HA  LEU   157      33.094  40.524  45.345  1.00  0.00           H
ATOM   2434  CB  LEU   157      32.308  41.217  47.234  1.00  0.00           C
ATOM   2435  HB2 LEU   157      31.574  41.958  47.556  1.00  0.00           H
ATOM   2436  HB3 LEU   157      31.982  40.253  47.625  1.00  0.00           H
ATOM   2437  CG  LEU   157      33.661  41.567  47.867  1.00  0.00           C
ATOM   2438  HG  LEU   157      34.046  42.494  47.458  1.00  0.00           H
ATOM   2439  CD1 LEU   157      33.496  41.704  49.374  1.00  0.00           C
ATOM   2440 HD11 LEU   157      34.460  41.925  49.829  1.00  0.00           H
ATOM   2441 HD12 LEU   157      32.813  42.525  49.589  1.00  0.00           H
ATOM   2442 HD13 LEU   157      33.105  40.779  49.799  1.00  0.00           H
ATOM   2443  CD2 LEU   157      34.677  40.487  47.524  1.00  0.00           C
ATOM   2444 HD21 LEU   157      35.605  40.679  48.063  1.00  0.00           H
ATOM   2445 HD22 LEU   157      34.307  39.505  47.808  1.00  0.00           H
ATOM   2446 HD23 LEU   157      34.909  40.497  46.461  1.00  0.00           H
ATOM   2447  C   LEU   157      31.024  40.554  45.269  1.00  0.00           C
ATOM   2448  O   LEU   157      31.036  39.312  45.101  1.00  0.00           O
ATOM   2449  N   LYS   158      29.903  41.333  45.172  1.00  0.00           N
ATOM   2450  H   LYS   158      30.032  42.310  45.400  1.00  0.00           H
ATOM   2451  CA  LYS   158      28.605  41.019  44.663  1.00  0.00           C
ATOM   2452  HA  LYS   158      28.241  40.137  45.187  1.00  0.00           H
ATOM   2453  CB  LYS   158      27.655  42.178  44.968  1.00  0.00           C
ATOM   2454  HB2 LYS   158      28.116  43.124  44.681  1.00  0.00           H
ATOM   2455  HB3 LYS   158      26.763  42.051  44.352  1.00  0.00           H
ATOM   2456  CG  LYS   158      27.195  42.249  46.419  1.00  0.00           C
ATOM   2457  HG2 LYS   158      26.747  41.291  46.686  1.00  0.00           H
ATOM   2458  HG3 LYS   158      28.046  42.425  47.077  1.00  0.00           H
```

ATOM 2459 CD LYS 158 26.159 43.345 46.616 1.00 0.00 C

ATOM 2460 HD2 LYS 158 26.634 44.316 46.473 1.00 0.00 H

ATOM 2461 HD3 LYS 158 25.368 43.232 45.873 1.00 0.00 H

ATOM 2462 CE LYS 158 25.533 43.273 48.002 1.00 0.00 C

ATOM 2463 HE2 LYS 158 25.036 42.308 48.115 1.00 0.00 H

ATOM 2464 HE3 LYS 158 26.324 43.347 48.750 1.00 0.00 H

ATOM 2465 NZ LYS 158 24.546 44.365 48.224 1.00 0.00 N

ATOM 2466 HZ1 LYS 158 23.806 44.301 47.539 1.00 0.00 H

ATOM 2467 HZ2 LYS 158 24.151 44.283 49.151 1.00 0.00 H

ATOM 2468 HZ3 LYS 158 25.001 45.263 48.135 1.00 0.00 H

ATOM 2469 C LYS 158 28.539 40.705 43.202 1.00 0.00 C

ATOM 2470 O LYS 158 27.931 39.737 42.744 1.00 0.00 O

ATOM 2471 N HIE 159 29.389 41.385 42.437 1.00 0.00 N

ATOM 2472 H HIE 159 29.935 42.137 42.834 1.00 0.00 H

ATOM 2473 CA HIE 159 29.595 41.039 41.046 1.00 0.00 C

ATOM 2474 HA HIE 159 28.622 40.914 40.569 1.00 0.00 H

ATOM 2475 CB HIE 159 30.348 42.160 40.321 1.00 0.00 C

ATOM 2476 HB2 HIE 159 29.912 43.112 40.622 1.00 0.00 H

ATOM 2477 HB3 HIE 159 31.392 42.159 40.622 1.00 0.00 H

ATOM 2478 CG HIE 159 30.254 42.079 38.828 1.00 0.00 C

ATOM 2479 ND1 HIE 159 30.654 43.108 38.002 1.00 0.00 N

ATOM 2480 CE1 HIE 159 30.457 42.758 36.742 1.00 0.00 C

ATOM 2481 HE1 HIE 159 30.657 43.352 35.867 1.00 0.00 H

ATOM 2482 NE2 HIE 159 29.944 41.541 36.724 1.00 0.00 N

ATOM 2483 HE2 HIE 159 29.681 41.026 35.896 1.00 0.00 H

ATOM 2484 CD2 HIE 159 29.807 41.094 38.015 1.00 0.00 C

ATOM 2485 HD2 HIE 159 29.427 40.137 38.319 1.00 0.00 H

ATOM 2486 C HIE 159 30.381 39.693 40.954 1.00 0.00 C

ATOM 2487 O HIE 159 29.959 38.782 40.230 1.00 0.00 O

ATOM 2488 N ILE 160 31.457 39.418 41.697 1.00 0.00 N

ATOM 2489 H ILE 160 31.777 40.133 42.329 1.00 0.00 H

ATOM 2490 CA ILE 160 32.261 38.200 41.659 1.00 0.00 C

ATOM 2491 HA ILE 160 32.663 38.098 40.649 1.00 0.00 H

ATOM 2492 CB ILE 160 33.445 38.336 42.634 1.00 0.00 C

ATOM 2493 HB ILE 160 33.066 38.647 43.607 1.00 0.00 H

ATOM 2494 CG2 ILE 160 34.144 36.997 42.812 1.00 0.00 C

ATOM 2495 HG21 ILE 160 35.076 37.109 43.362 1.00 0.00 H

ATOM 2496 HG22 ILE 160 33.533 36.306 43.392 1.00 0.00 H

ATOM 2497 HG23 ILE 160 34.365 36.553 41.842 1.00 0.00 H

ATOM 2498 CG1 ILE 160 34.429 39.396 42.134 1.00 0.00 C

ATOM 2499 HG12 ILE 160 34.957 39.023 41.262 1.00 0.00 H

ATOM 2500 HG13 ILE 160 33.903 40.278 41.809 1.00 0.00 H

ATOM 2501 CD1 ILE 160 35.449 39.818 43.167 1.00 0.00 C

ATOM 2502 HD11 ILE 160 36.004 40.680 42.797 1.00 0.00 H

ATOM 2503 HD12 ILE 160 34.948 40.099 44.091 1.00 0.00 H

ATOM 2504 HD13 ILE 160 36.154 39.013 43.367 1.00 0.00 H

ATOM 2505 C ILE 160 31.524 36.899 41.977 1.00 0.00 C

ATOM 2506 O ILE 160 31.333 36.016 41.130 1.00 0.00 O

ATOM 2507 N LEU 161 30.790 36.858 43.107 1.00 0.00 N

ATOM 2508 H LEU 161 30.842 37.646 43.737 1.00 0.00 H

ATOM 2509 CA LEU 161 29.999 35.688 43.557 1.00 0.00 C

ATOM 2510 HA LEU 161 30.653 34.815 43.547 1.00 0.00 H

ATOM 2511 CB LEU 161 29.508 35.909 44.993 1.00 0.00 C

ATOM 2512 HB2 LEU 161 29.019 35.008 45.344 1.00 0.00 H

ATOM 2513 HB3 LEU 161 30.395 36.017 45.609 1.00 0.00 H

ATOM 2514 CG LEU 161 28.619 37.140 45.210 1.00 0.00 C

ATOM 2515 HG LEU 161 28.924 37.954 44.558 1.00 0.00 H

ATOM 2516 CD1 LEU 161 27.173 36.786 44.892 1.00 0.00 C

ATOM 2517 HD11 LEU 161 26.496 37.486 45.380 1.00 0.00 H

ATOM 2518 HD12 LEU 161 27.010 36.872 43.827 1.00 0.00 H

| | | | | | | | | | | |
|---|---|---|---|---|---|---|---|---|---|---|
| ATOM | 2519 | HD13 | LEU | 161 | 26.930 | 35.776 | 45.217 | 1.00 | 0.00 | H |
| ATOM | 2520 | CD2 | LEU | 161 | 28.761 | 37.622 | 46.646 | 1.00 | 0.00 | C |
| ATOM | 2521 | HD21 | LEU | 161 | 28.123 | 38.489 | 46.813 | 1.00 | 0.00 | H |
| ATOM | 2522 | HD22 | LEU | 161 | 28.478 | 36.833 | 47.340 | 1.00 | 0.00 | H |
| ATOM | 2523 | HD23 | LEU | 161 | 29.790 | 37.914 | 46.844 | 1.00 | 0.00 | H |
| ATOM | 2524 | C | LEU | 161 | 28.804 | 35.380 | 42.685 | 1.00 | 0.00 | C |
| ATOM | 2525 | O | LEU | 161 | 28.135 | 34.374 | 42.971 | 1.00 | 0.00 | O |
| ATOM | 2526 | N | LEU | 162 | 28.469 | 36.108 | 41.591 | 1.00 | 0.00 | N |
| ATOM | 2527 | H | LEU | 162 | 29.079 | 36.887 | 41.386 | 1.00 | 0.00 | H |
| ATOM | 2528 | CA | LEU | 162 | 27.395 | 35.939 | 40.652 | 1.00 | 0.00 | C |
| ATOM | 2529 | HA | LEU | 162 | 26.974 | 34.948 | 40.809 | 1.00 | 0.00 | H |
| ATOM | 2530 | CB | LEU | 162 | 26.323 | 36.988 | 40.972 | 1.00 | 0.00 | C |
| ATOM | 2531 | HB2 | LEU | 162 | 25.949 | 36.807 | 41.977 | 1.00 | 0.00 | H |
| ATOM | 2532 | HB3 | LEU | 162 | 26.792 | 37.973 | 40.953 | 1.00 | 0.00 | H |
| ATOM | 2533 | CG | LEU | 162 | 25.116 | 37.017 | 40.025 | 1.00 | 0.00 | C |
| ATOM | 2534 | HG | LEU | 162 | 25.416 | 37.278 | 39.020 | 1.00 | 0.00 | H |
| ATOM | 2535 | CD1 | LEU | 162 | 24.426 | 35.660 | 40.040 | 1.00 | 0.00 | C |
| ATOM | 2536 | HD11 | LEU | 162 | 23.525 | 35.704 | 39.428 | 1.00 | 0.00 | H |
| ATOM | 2537 | HD12 | LEU | 162 | 25.062 | 34.883 | 39.628 | 1.00 | 0.00 | H |
| ATOM | 2538 | HD13 | LEU | 162 | 24.149 | 35.389 | 41.059 | 1.00 | 0.00 | H |
| ATOM | 2539 | CD2 | LEU | 162 | 24.161 | 38.121 | 40.453 | 1.00 | 0.00 | C |
| ATOM | 2540 | HD21 | LEU | 162 | 23.307 | 38.152 | 39.776 | 1.00 | 0.00 | H |
| ATOM | 2541 | HD22 | LEU | 162 | 23.808 | 37.943 | 41.469 | 1.00 | 0.00 | H |
| ATOM | 2542 | HD23 | LEU | 162 | 24.671 | 39.084 | 40.412 | 1.00 | 0.00 | H |
| ATOM | 2543 | C | LEU | 162 | 27.787 | 36.034 | 39.139 | 1.00 | 0.00 | C |
| ATOM | 2544 | O | LEU | 162 | 26.954 | 35.692 | 38.283 | 1.00 | 0.00 | O |
| ATOM | 2545 | N | ASP | 163 | 28.956 | 36.567 | 38.821 | 1.00 | 0.00 | N |
| ATOM | 2546 | H | ASP | 163 | 29.606 | 36.800 | 39.560 | 1.00 | 0.00 | H |
| ATOM | 2547 | CA | ASP | 163 | 29.330 | 36.989 | 37.433 | 1.00 | 0.00 | C |
| ATOM | 2548 | HA | ASP | 163 | 30.242 | 37.577 | 37.513 | 1.00 | 0.00 | H |

ATOM 2549 CB ASP 163 29.621 35.757 36.572 1.00 0.00 C

ATOM 2550 HB2 ASP 163 28.761 35.086 36.559 1.00 0.00 H

ATOM 2551 HB3 ASP 163 29.824 36.056 35.543 1.00 0.00 H

ATOM 2552 CG ASP 163 30.863 34.999 37.022 1.00 0.00 C

ATOM 2553 OD1 ASP 163 31.794 35.632 37.461 1.00 0.00 O

ATOM 2554 OD2 ASP 163 30.868 33.796 36.922 1.00 0.00 O

ATOM 2555 C ASP 163 28.364 37.814 36.673 1.00 0.00 C

ATOM 2556 O ASP 163 28.075 37.750 35.473 1.00 0.00 O

ATOM 2557 N GLY 164 27.593 38.658 37.405 1.00 0.00 N

ATOM 2558 H GLY 164 27.796 38.718 38.392 1.00 0.00 H

ATOM 2559 CA GLY 164 26.432 39.348 36.974 1.00 0.00 C

ATOM 2560 HA2 GLY 164 26.133 40.047 37.755 1.00 0.00 H

ATOM 2561 HA3 GLY 164 26.675 39.919 36.077 1.00 0.00 H

ATOM 2562 C GLY 164 25.190 38.379 36.647 1.00 0.00 C

ATOM 2563 O GLY 164 24.065 38.591 37.095 1.00 0.00 O

ATOM 2564 N SER 165 25.507 37.335 35.889 1.00 0.00 N

ATOM 2565 H SER 165 26.431 37.222 35.508 1.00 0.00 H

ATOM 2566 CA SER 165 24.646 36.182 35.904 1.00 0.00 C

ATOM 2567 HA SER 165 24.200 36.077 36.895 1.00 0.00 H

ATOM 2568 CB SER 165 23.526 36.383 34.902 1.00 0.00 C

ATOM 2569 HB2 SER 165 23.950 36.580 33.917 1.00 0.00 H

ATOM 2570 HB3 SER 165 22.918 35.481 34.848 1.00 0.00 H

ATOM 2571 OG SER 165 22.703 37.453 35.276 1.00 0.00 O

ATOM 2572 HG SER 165 23.261 38.188 35.549 1.00 0.00 H

ATOM 2573 C SER 165 25.450 34.869 35.588 1.00 0.00 C

ATOM 2574 O SER 165 26.062 34.658 34.531 1.00 0.00 O

ATOM 2575 N LEU 166 25.512 34.063 36.641 1.00 0.00 N

ATOM 2576 H LEU 166 25.079 34.333 37.512 1.00 0.00 H

ATOM 2577 CA LEU 166 26.334 32.817 36.613 1.00 0.00 C

ATOM 2578 HA LEU 166 27.253 33.024 36.061 1.00 0.00 H

| | | | | | | | | | | |
|---|---|---|---|---|---|---|---|---|---|---|
| ATOM | 2579 | CB | LEU | 166 | 26.729 | 32.446 | 38.048 | 1.00 | 0.00 | C |
| ATOM | 2580 | HB2 | LEU | 166 | 27.634 | 31.843 | 38.010 | 1.00 | 0.00 | H |
| ATOM | 2581 | HB3 | LEU | 166 | 27.024 | 33.343 | 38.577 | 1.00 | 0.00 | H |
| ATOM | 2582 | CG | LEU | 166 | 25.656 | 31.714 | 38.865 | 1.00 | 0.00 | C |
| ATOM | 2583 | HG | LEU | 166 | 24.670 | 32.122 | 38.656 | 1.00 | 0.00 | H |
| ATOM | 2584 | CD1 | LEU | 166 | 25.677 | 30.231 | 38.519 | 1.00 | 0.00 | C |
| ATOM | 2585 | HD11 | LEU | 166 | 25.350 | 29.661 | 39.389 | 1.00 | 0.00 | H |
| ATOM | 2586 | HD12 | LEU | 166 | 24.965 | 29.979 | 37.741 | 1.00 | 0.00 | H |
| ATOM | 2587 | HD13 | LEU | 166 | 26.673 | 29.875 | 38.269 | 1.00 | 0.00 | H |
| ATOM | 2588 | CD2 | LEU | 166 | 25.911 | 31.933 | 40.349 | 1.00 | 0.00 | C |
| ATOM | 2589 | HD21 | LEU | 166 | 25.153 | 31.413 | 40.936 | 1.00 | 0.00 | H |
| ATOM | 2590 | HD22 | LEU | 166 | 26.898 | 31.553 | 40.619 | 1.00 | 0.00 | H |
| ATOM | 2591 | HD23 | LEU | 166 | 25.862 | 32.991 | 40.593 | 1.00 | 0.00 | H |
| ATOM | 2592 | C | LEU | 166 | 25.609 | 31.583 | 35.929 | 1.00 | 0.00 | C |
| ATOM | 2593 | O | LEU | 166 | 26.329 | 30.645 | 35.638 | 1.00 | 0.00 | O |
| ATOM | 2594 | N | PRO | 167 | 24.318 | 31.510 | 35.685 | 1.00 | 0.00 | N |
| ATOM | 2595 | CD | PRO | 167 | 23.346 | 32.553 | 36.081 | 1.00 | 0.00 | C |
| ATOM | 2596 | HD2 | PRO | 167 | 23.371 | 33.381 | 35.376 | 1.00 | 0.00 | H |
| ATOM | 2597 | HD3 | PRO | 167 | 23.431 | 32.874 | 37.117 | 1.00 | 0.00 | H |
| ATOM | 2598 | CG | PRO | 167 | 22.043 | 31.819 | 35.906 | 1.00 | 0.00 | C |
| ATOM | 2599 | HG2 | PRO | 167 | 21.232 | 32.502 | 35.651 | 1.00 | 0.00 | H |
| ATOM | 2600 | HG3 | PRO | 167 | 21.804 | 31.276 | 36.823 | 1.00 | 0.00 | H |
| ATOM | 2601 | CB | PRO | 167 | 22.308 | 30.849 | 34.807 | 1.00 | 0.00 | C |
| ATOM | 2602 | HB2 | PRO | 167 | 22.281 | 31.367 | 33.847 | 1.00 | 0.00 | H |
| ATOM | 2603 | HB3 | PRO | 167 | 21.582 | 30.035 | 34.811 | 1.00 | 0.00 | H |
| ATOM | 2604 | CA | PRO | 167 | 23.721 | 30.331 | 35.095 | 1.00 | 0.00 | C |
| ATOM | 2605 | HA | PRO | 167 | 23.678 | 29.535 | 35.839 | 1.00 | 0.00 | H |
| ATOM | 2606 | C | PRO | 167 | 24.461 | 29.792 | 33.757 | 1.00 | 0.00 | C |
| ATOM | 2607 | O | PRO | 167 | 24.497 | 28.594 | 33.543 | 1.00 | 0.00 | O |
| ATOM | 2608 | N | PRO | 168 | 25.077 | 30.597 | 32.808 | 1.00 | 0.00 | N |

| | | | | | | | | | | |
|---|---|---|---|---|---|---|---|---|---|---|
| ATOM | 2609 | CD | PRO | 168 | 25.085 | 32.090 | 32.832 | 1.00 | 0.00 | C |
| ATOM | 2610 | HD2 | PRO | 168 | 25.867 | 32.432 | 33.506 | 1.00 | 0.00 | H |
| ATOM | 2611 | HD3 | PRO | 168 | 24.116 | 32.513 | 33.092 | 1.00 | 0.00 | H |
| ATOM | 2612 | CG | PRO | 168 | 25.466 | 32.452 | 31.421 | 1.00 | 0.00 | C |
| ATOM | 2613 | HG2 | PRO | 168 | 25.956 | 33.425 | 31.380 | 1.00 | 0.00 | H |
| ATOM | 2614 | HG3 | PRO | 168 | 24.575 | 32.452 | 30.791 | 1.00 | 0.00 | H |
| ATOM | 2615 | CB | PRO | 168 | 26.382 | 31.357 | 30.992 | 1.00 | 0.00 | C |
| ATOM | 2616 | HB2 | PRO | 168 | 27.370 | 31.529 | 31.423 | 1.00 | 0.00 | H |
| ATOM | 2617 | HB3 | PRO | 168 | 26.453 | 31.296 | 29.906 | 1.00 | 0.00 | H |
| ATOM | 2618 | CA | PRO | 168 | 25.756 | 30.090 | 31.584 | 1.00 | 0.00 | C |
| ATOM | 2619 | HA | PRO | 168 | 25.010 | 29.687 | 30.898 | 1.00 | 0.00 | H |
| ATOM | 2620 | C | PRO | 168 | 26.833 | 29.029 | 31.910 | 1.00 | 0.00 | C |
| ATOM | 2621 | O | PRO | 168 | 27.686 | 29.155 | 32.794 | 1.00 | 0.00 | O |
| ATOM | 2622 | N | LYS | 169 | 26.750 | 27.837 | 31.316 | 1.00 | 0.00 | N |
| ATOM | 2623 | H | LYS | 169 | 26.022 | 27.700 | 30.631 | 1.00 | 0.00 | H |
| ATOM | 2624 | CA | LYS | 169 | 27.613 | 26.666 | 31.608 | 1.00 | 0.00 | C |
| ATOM | 2625 | HA | LYS | 169 | 27.126 | 25.783 | 31.195 | 1.00 | 0.00 | H |
| ATOM | 2626 | CB | LYS | 169 | 28.968 | 26.840 | 30.922 | 1.00 | 0.00 | C |
| ATOM | 2627 | HB2 | LYS | 169 | 29.463 | 27.732 | 31.312 | 1.00 | 0.00 | H |
| ATOM | 2628 | HB3 | LYS | 169 | 29.599 | 25.978 | 31.147 | 1.00 | 0.00 | H |
| ATOM | 2629 | CG | LYS | 169 | 28.898 | 26.928 | 29.403 | 1.00 | 0.00 | C |
| ATOM | 2630 | HG2 | LYS | 169 | 28.404 | 26.036 | 29.014 | 1.00 | 0.00 | H |
| ATOM | 2631 | HG3 | LYS | 169 | 28.318 | 27.805 | 29.114 | 1.00 | 0.00 | H |
| ATOM | 2632 | CD | LYS | 169 | 30.287 | 27.037 | 28.791 | 1.00 | 0.00 | C |
| ATOM | 2633 | HD2 | LYS | 169 | 30.786 | 27.920 | 29.194 | 1.00 | 0.00 | H |
| ATOM | 2634 | HD3 | LYS | 169 | 30.868 | 26.152 | 29.055 | 1.00 | 0.00 | H |
| ATOM | 2635 | CE | LYS | 169 | 30.217 | 27.155 | 27.276 | 1.00 | 0.00 | C |
| ATOM | 2636 | HE2 | LYS | 169 | 29.733 | 26.264 | 26.873 | 1.00 | 0.00 | H |
| ATOM | 2637 | HE3 | LYS | 169 | 29.612 | 28.026 | 27.019 | 1.00 | 0.00 | H |
| ATOM | 2638 | NZ | LYS | 169 | 31.566 | 27.301 | 26.666 | 1.00 | 0.00 | N |

| | | | | | | | | | | |
|---|---|---|---|---|---|---|---|---|---|---|
| ATOM | 2639 | HZ1 | LYS | 169 | 31.482 | 27.393 | 25.663 | 1.00 | 0.00 | H |
| ATOM | 2640 | HZ2 | LYS | 169 | 32.020 | 28.124 | 27.040 | 1.00 | 0.00 | H |
| ATOM | 2641 | HZ3 | LYS | 169 | 32.127 | 26.487 | 26.878 | 1.00 | 0.00 | H |
| ATOM | 2642 | C | LYS | 169 | 27.833 | 26.401 | 33.118 | 1.00 | 0.00 | C |
| ATOM | 2643 | O | LYS | 169 | 28.864 | 25.906 | 33.448 | 1.00 | 0.00 | O |
| ATOM | 2644 | N | ARG | 170 | 26.776 | 26.605 | 33.925 | 1.00 | 0.00 | N |
| ATOM | 2645 | H | ARG | 170 | 25.957 | 27.004 | 33.488 | 1.00 | 0.00 | H |
| ATOM | 2646 | CA | ARG | 170 | 26.667 | 26.431 | 35.398 | 1.00 | 0.00 | C |
| ATOM | 2647 | HA | ARG | 170 | 25.780 | 26.967 | 35.737 | 1.00 | 0.00 | H |
| ATOM | 2648 | CB | ARG | 170 | 26.505 | 24.950 | 35.711 | 1.00 | 0.00 | C |
| ATOM | 2649 | HB2 | ARG | 170 | 27.397 | 24.411 | 35.384 | 1.00 | 0.00 | H |
| ATOM | 2650 | HB3 | ARG | 170 | 26.409 | 24.828 | 36.791 | 1.00 | 0.00 | H |
| ATOM | 2651 | CG | ARG | 170 | 25.276 | 24.298 | 35.099 | 1.00 | 0.00 | C |
| ATOM | 2652 | HG2 | ARG | 170 | 24.388 | 24.848 | 35.416 | 1.00 | 0.00 | H |
| ATOM | 2653 | HG3 | ARG | 170 | 25.344 | 24.339 | 34.011 | 1.00 | 0.00 | H |
| ATOM | 2654 | CD | ARG | 170 | 25.144 | 22.878 | 35.515 | 1.00 | 0.00 | C |
| ATOM | 2655 | HD2 | ARG | 170 | 26.038 | 22.336 | 35.203 | 1.00 | 0.00 | H |
| ATOM | 2656 | HD3 | ARG | 170 | 25.066 | 22.832 | 36.603 | 1.00 | 0.00 | H |
| ATOM | 2657 | NE | ARG | 170 | 23.972 | 22.248 | 34.927 | 1.00 | 0.00 | N |
| ATOM | 2658 | HE | ARG | 170 | 23.367 | 22.834 | 34.371 | 1.00 | 0.00 | H |
| ATOM | 2659 | CZ | ARG | 170 | 23.645 | 20.949 | 35.079 | 1.00 | 0.00 | C |
| ATOM | 2660 | NH1 | ARG | 170 | 24.408 | 20.159 | 35.800 | 1.00 | 0.00 | N |
| ATOM | 2661 | HH11 | ARG | 170 | 24.179 | 19.182 | 35.898 | 1.00 | 0.00 | H |
| ATOM | 2662 | HH12 | ARG | 170 | 25.223 | 20.536 | 36.260 | 1.00 | 0.00 | H |
| ATOM | 2663 | NH2 | ARG | 170 | 22.556 | 20.471 | 34.502 | 1.00 | 0.00 | N |
| ATOM | 2664 | HH21 | ARG | 170 | 21.955 | 21.086 | 33.975 | 1.00 | 0.00 | H |
| ATOM | 2665 | HH22 | ARG | 170 | 22.322 | 19.494 | 34.592 | 1.00 | 0.00 | H |
| ATOM | 2666 | C | ARG | 170 | 27.930 | 27.026 | 36.214 | 1.00 | 0.00 | C |
| ATOM | 2667 | O | ARG | 170 | 28.270 | 26.318 | 37.209 | 1.00 | 0.00 | O |
| ATOM | 2668 | N | ALA | 171 | 28.594 | 28.065 | 35.743 | 1.00 | 0.00 | N |

```
ATOM   2669  H   ALA   171      28.194  28.519  34.934  1.00  0.00           H
ATOM   2670  CA  ALA   171      29.854  28.639  36.192  1.00  0.00           C
ATOM   2671  HA  ALA   171      30.048  29.470  35.514  1.00  0.00           H
ATOM   2672  CB  ALA   171      29.530  29.262  37.548  1.00  0.00           C
ATOM   2673  HB1 ALA   171      30.380  29.851  37.895  1.00  0.00           H
ATOM   2674  HB2 ALA   171      28.689  29.940  37.440  1.00  0.00           H
ATOM   2675  HB3 ALA   171      29.304  28.498  38.293  1.00  0.00           H
ATOM   2676  C   ALA   171      31.170  27.814  36.210  1.00  0.00           C
ATOM   2677  O   ALA   171      31.987  27.923  37.106  1.00  0.00           O
ATOM   2678  N   VAL   172      31.317  26.876  35.277  1.00  0.00           N
ATOM   2679  H   VAL   172      30.582  26.771  34.591  1.00  0.00           H
ATOM   2680  CA  VAL   172      32.503  25.984  35.126  1.00  0.00           C
ATOM   2681  HA  VAL   172      32.843  25.675  36.109  1.00  0.00           H
ATOM   2682  CB  VAL   172      32.129  24.721  34.329  1.00  0.00           C
ATOM   2683  HB  VAL   172      31.743  25.012  33.350  1.00  0.00           H
ATOM   2684  CG1 VAL   172      33.351  23.839  34.124  1.00  0.00           C
ATOM   2685 HG11 VAL   172      33.039  22.882  33.705  1.00  0.00           H
ATOM   2686 HG12 VAL   172      34.046  24.290  33.417  1.00  0.00           H
ATOM   2687 HG13 VAL   172      33.852  23.657  35.076  1.00  0.00           H
ATOM   2688  CG2 VAL   172      31.029  23.959  35.053  1.00  0.00           C
ATOM   2689 HG21 VAL   172      30.678  23.145  34.418  1.00  0.00           H
ATOM   2690 HG22 VAL   172      31.414  23.530  35.975  1.00  0.00           H
ATOM   2691 HG23 VAL   172      30.176  24.594  35.284  1.00  0.00           H
ATOM   2692  C   VAL   172      33.665  26.651  34.431  1.00  0.00           C
ATOM   2693  O   VAL   172      33.523  26.976  33.253  1.00  0.00           O
ATOM   2694  N   THR   173      34.868  26.855  35.036  1.00  0.00           N
ATOM   2695  H   THR   173      34.966  26.573  36.000  1.00  0.00           H
ATOM   2696  CA  THR   173      36.008  27.513  34.424  1.00  0.00           C
ATOM   2697  HA  THR   173      36.695  27.773  35.229  1.00  0.00           H
ATOM   2698  CB  THR   173      36.717  26.484  33.524  1.00  0.00           C
```

| | | | | | | | | | | |
|---|---|---|---|---|---|---|---|---|---|---|
| ATOM | 2699 | HB | THR | 173 | 37.646 | 26.914 | 33.148 | 1.00 | 0.00 | H |
| ATOM | 2700 | CG2 | THR | 173 | 37.050 | 25.226 | 34.312 | 1.00 | 0.00 | C |
| ATOM | 2701 | HG21 | THR | 173 | 37.729 | 24.628 | 33.710 | 1.00 | 0.00 | H |
| ATOM | 2702 | HG22 | THR | 173 | 37.552 | 25.494 | 35.242 | 1.00 | 0.00 | H |
| ATOM | 2703 | HG23 | THR | 173 | 36.160 | 24.638 | 34.532 | 1.00 | 0.00 | H |
| ATOM | 2704 | OG1 | THR | 173 | 35.867 | 26.143 | 32.422 | 1.00 | 0.00 | O |
| ATOM | 2705 | HG1 | THR | 173 | 35.701 | 26.955 | 31.933 | 1.00 | 0.00 | H |
| ATOM | 2706 | C | THR | 173 | 35.759 | 28.840 | 33.593 | 1.00 | 0.00 | C |
| ATOM | 2707 | O | THR | 173 | 36.279 | 28.985 | 32.468 | 1.00 | 0.00 | O |
| ATOM | 2708 | N | THR | 174 | 34.878 | 29.684 | 34.181 | 1.00 | 0.00 | N |
| ATOM | 2709 | H | THR | 174 | 34.492 | 29.436 | 35.081 | 1.00 | 0.00 | H |
| ATOM | 2710 | CA | THR | 174 | 34.448 | 30.954 | 33.616 | 1.00 | 0.00 | C |
| ATOM | 2711 | HA | THR | 174 | 35.240 | 31.322 | 32.963 | 1.00 | 0.00 | H |
| ATOM | 2712 | CB | THR | 174 | 33.179 | 30.774 | 32.763 | 1.00 | 0.00 | C |
| ATOM | 2713 | HB | THR | 174 | 33.364 | 30.020 | 31.998 | 1.00 | 0.00 | H |
| ATOM | 2714 | CG2 | THR | 174 | 32.012 | 30.328 | 33.630 | 1.00 | 0.00 | C |
| ATOM | 2715 | HG21 | THR | 174 | 31.155 | 30.122 | 32.989 | 1.00 | 0.00 | H |
| ATOM | 2716 | HG22 | THR | 174 | 32.262 | 29.412 | 34.160 | 1.00 | 0.00 | H |
| ATOM | 2717 | HG23 | THR | 174 | 31.727 | 31.099 | 34.346 | 1.00 | 0.00 | H |
| ATOM | 2718 | OG1 | THR | 174 | 32.849 | 32.015 | 32.125 | 1.00 | 0.00 | O |
| ATOM | 2719 | HG1 | THR | 174 | 33.535 | 32.221 | 31.486 | 1.00 | 0.00 | H |
| ATOM | 2720 | C | THR | 174 | 34.183 | 32.003 | 34.631 | 1.00 | 0.00 | C |
| ATOM | 2721 | O | THR | 174 | 34.068 | 31.717 | 35.816 | 1.00 | 0.00 | O |
| ATOM | 2722 | N | CYS | 175 | 34.225 | 33.330 | 34.309 | 1.00 | 0.00 | N |
| ATOM | 2723 | H | CYS | 175 | 34.400 | 33.557 | 33.342 | 1.00 | 0.00 | H |
| ATOM | 2724 | CA | CYS | 175 | 33.965 | 34.461 | 35.149 | 1.00 | 0.00 | C |
| ATOM | 2725 | HA | CYS | 175 | 33.044 | 34.248 | 35.690 | 1.00 | 0.00 | H |
| ATOM | 2726 | CB | CYS | 175 | 35.091 | 34.556 | 36.179 | 1.00 | 0.00 | C |
| ATOM | 2727 | HB2 | CYS | 175 | 34.954 | 35.458 | 36.767 | 1.00 | 0.00 | H |
| ATOM | 2728 | HB3 | CYS | 175 | 35.027 | 33.716 | 36.868 | 1.00 | 0.00 | H |

| | | | | | | | | | | |
|---|---|---|---|---|---|---|---|---|---|---|
| ATOM | 2729 | SG | CYS | 175 | 36.751 | 34.559 | 35.461 | 1.00 | 0.00 | S |
| ATOM | 2730 | HG | CYS | 175 | 36.647 | 35.752 | 34.839 | 1.00 | 0.00 | H |
| ATOM | 2731 | C | CYS | 175 | 33.808 | 35.821 | 34.537 | 1.00 | 0.00 | C |
| ATOM | 2732 | O | CYS | 175 | 34.429 | 36.139 | 33.493 | 1.00 | 0.00 | O |
| ATOM | 2733 | N | THR | 176 | 33.104 | 36.766 | 35.262 | 1.00 | 0.00 | N |
| ATOM | 2734 | H | THR | 176 | 32.684 | 36.460 | 36.129 | 1.00 | 0.00 | H |
| ATOM | 2735 | CA | THR | 176 | 32.906 | 38.232 | 34.928 | 1.00 | 0.00 | C |
| ATOM | 2736 | HA | THR | 176 | 33.686 | 38.515 | 34.220 | 1.00 | 0.00 | H |
| ATOM | 2737 | CB | THR | 176 | 31.547 | 38.447 | 34.237 | 1.00 | 0.00 | C |
| ATOM | 2738 | HB | THR | 176 | 30.744 | 38.168 | 34.918 | 1.00 | 0.00 | H |
| ATOM | 2739 | CG2 | THR | 176 | 31.375 | 39.906 | 33.841 | 1.00 | 0.00 | C |
| ATOM | 2740 | HG21 | THR | 176 | 31.279 | 40.543 | 34.714 | 1.00 | 0.00 | H |
| ATOM | 2741 | HG22 | THR | 176 | 32.226 | 40.235 | 33.243 | 1.00 | 0.00 | H |
| ATOM | 2742 | HG23 | THR | 176 | 30.465 | 40.013 | 33.249 | 1.00 | 0.00 | H |
| ATOM | 2743 | OG1 | THR | 176 | 31.469 | 37.626 | 33.064 | 1.00 | 0.00 | O |
| ATOM | 2744 | HG1 | THR | 176 | 31.498 | 36.707 | 33.341 | 1.00 | 0.00 | H |
| ATOM | 2745 | C | THR | 176 | 32.988 | 39.208 | 36.122 | 1.00 | 0.00 | C |
| ATOM | 2746 | O | THR | 176 | 32.286 | 39.041 | 37.152 | 1.00 | 0.00 | O |
| ATOM | 2747 | N | LEU | 177 | 33.682 | 40.316 | 36.000 | 1.00 | 0.00 | N |
| ATOM | 2748 | H | LEU | 177 | 34.178 | 40.458 | 35.132 | 1.00 | 0.00 | H |
| ATOM | 2749 | CA | LEU | 177 | 33.808 | 41.413 | 37.006 | 1.00 | 0.00 | C |
| ATOM | 2750 | HA | LEU | 177 | 32.867 | 41.499 | 37.549 | 1.00 | 0.00 | H |
| ATOM | 2751 | CB | LEU | 177 | 34.929 | 41.115 | 38.010 | 1.00 | 0.00 | C |
| ATOM | 2752 | HB2 | LEU | 177 | 34.656 | 40.226 | 38.581 | 1.00 | 0.00 | H |
| ATOM | 2753 | HB3 | LEU | 177 | 35.828 | 40.891 | 37.436 | 1.00 | 0.00 | H |
| ATOM | 2754 | CG | LEU | 177 | 35.278 | 42.255 | 38.975 | 1.00 | 0.00 | C |
| ATOM | 2755 | HG | LEU | 177 | 35.578 | 43.144 | 38.423 | 1.00 | 0.00 | H |
| ATOM | 2756 | CD1 | LEU | 177 | 34.059 | 42.595 | 39.822 | 1.00 | 0.00 | C |
| ATOM | 2757 | HD11 | LEU | 177 | 34.348 | 43.279 | 40.618 | 1.00 | 0.00 | H |
| ATOM | 2758 | HD12 | LEU | 177 | 33.296 | 43.086 | 39.225 | 1.00 | 0.00 | H |

ATOM 2759 HD13 LEU 177 33.635 41.694 40.253 1.00 0.00 H

ATOM 2760 CD2 LEU 177 36.452 41.839 39.849 1.00 0.00 C

ATOM 2761 HD21 LEU 177 36.642 42.602 40.604 1.00 0.00 H

ATOM 2762 HD22 LEU 177 36.239 40.895 40.347 1.00 0.00 H

ATOM 2763 HD23 LEU 177 37.343 41.728 39.238 1.00 0.00 H

ATOM 2764 C LEU 177 34.096 42.735 36.278 1.00 0.00 C

ATOM 2765 O LEU 177 34.888 42.895 35.364 1.00 0.00 O

ATOM 2766 N THR 178 33.346 43.761 36.662 1.00 0.00 N

ATOM 2767 H THR 178 32.628 43.580 37.349 1.00 0.00 H

ATOM 2768 CA THR 178 33.586 45.199 36.330 1.00 0.00 C

ATOM 2769 HA THR 178 34.616 45.307 35.989 1.00 0.00 H

ATOM 2770 CB THR 178 32.660 45.662 35.191 1.00 0.00 C

ATOM 2771 HB THR 178 32.921 46.682 34.906 1.00 0.00 H

ATOM 2772 CG2 THR 178 32.812 44.757 33.978 1.00 0.00 C

ATOM 2773 HG21 THR 178 32.238 45.184 33.155 1.00 0.00 H

ATOM 2774 HG22 THR 178 33.858 44.710 33.674 1.00 0.00 H

ATOM 2775 HG23 THR 178 32.432 43.754 34.175 1.00 0.00 H

ATOM 2776 OG1 THR 178 31.297 45.633 35.636 1.00 0.00 O

ATOM 2777 HG1 THR 178 31.183 46.285 36.334 1.00 0.00 H

ATOM 2778 C THR 178 33.408 46.152 37.489 1.00 0.00 C

ATOM 2779 O THR 178 33.882 47.282 37.386 1.00 0.00 O

ATOM 2780 N GLU 179 32.580 45.742 38.433 1.00 0.00 N

ATOM 2781 H GLU 179 32.154 44.827 38.394 1.00 0.00 H

ATOM 2782 CA GLU 179 32.258 46.625 39.536 1.00 0.00 C

ATOM 2783 HA GLU 179 31.907 47.580 39.140 1.00 0.00 H

ATOM 2784 CB GLU 179 31.138 46.025 40.389 1.00 0.00 C

ATOM 2785 HB2 GLU 179 31.409 45.001 40.639 1.00 0.00 H

ATOM 2786 HB3 GLU 179 31.069 46.583 41.316 1.00 0.00 H

ATOM 2787 CG GLU 179 29.774 46.008 39.713 1.00 0.00 C

ATOM 2788 HG2 GLU 179 29.851 45.525 38.739 1.00 0.00 H

```
ATOM   2789  HG3 GLU   179      29.093  45.417  40.327  1.00  0.00           H
ATOM   2790  CD  GLU   179      29.186  47.381  39.548  1.00  0.00           C
ATOM   2791  OE1 GLU   179      29.141  48.107  40.512  1.00  0.00           O
ATOM   2792  OE2 GLU   179      28.781  47.705  38.456  1.00  0.00           O
ATOM   2793  C   GLU   179      33.503  46.882  40.395  1.00  0.00           C
ATOM   2794  O   GLU   179      34.451  46.072  40.386  1.00  0.00           O
ATOM   2795  N   ASN   180      33.550  47.988  41.062  1.00  0.00           N
ATOM   2796  H   ASN   180      32.762  48.620  41.035  1.00  0.00           H
ATOM   2797  CA  ASN   180      34.778  48.424  41.801  1.00  0.00           C
ATOM   2798  HA  ASN   180      35.641  48.066  41.248  1.00  0.00           H
ATOM   2799  CB  ASN   180      34.864  49.939  41.774  1.00  0.00           C
ATOM   2800  HB2 ASN   180      35.801  50.266  42.225  1.00  0.00           H
ATOM   2801  HB3 ASN   180      34.863  50.274  40.735  1.00  0.00           H
ATOM   2802  CG  ASN   180      33.726  50.595  42.506  1.00  0.00           C
ATOM   2803  OD1 ASN   180      33.263  51.705  41.990  1.00  0.00           O
ATOM   2804  ND2 ASN   180      33.266  50.096  43.539  1.00  0.00           N
ATOM   2805 HD21 ASN   180      32.461  50.531  43.962  1.00  0.00           H
ATOM   2806 HD22 ASN   180      33.662  49.265  43.946  1.00  0.00           H
ATOM   2807  C   ASN   180      34.908  47.926  43.276  1.00  0.00           C
ATOM   2808  O   ASN   180      33.943  47.386  43.840  1.00  0.00           O
ATOM   2809  N   MET   181      36.128  48.134  43.805  1.00  0.00           N
ATOM   2810  H   MET   181      36.832  48.541  43.203  1.00  0.00           H
ATOM   2811  CA  MET   181      36.501  47.976  45.140  1.00  0.00           C
ATOM   2812  HA  MET   181      35.811  48.538  45.772  1.00  0.00           H
ATOM   2813  CB  MET   181      36.452  46.504  45.545  1.00  0.00           C
ATOM   2814  HB2 MET   181      36.742  46.444  46.594  1.00  0.00           H
ATOM   2815  HB3 MET   181      35.432  46.139  45.479  1.00  0.00           H
ATOM   2816  CG  MET   181      37.393  45.603  44.758  1.00  0.00           C
ATOM   2817  HG2 MET   181      37.157  45.696  43.703  1.00  0.00           H
ATOM   2818  HG3 MET   181      38.427  45.910  44.918  1.00  0.00           H
```

```
ATOM   2819  SD  MET   181      37.214  43.861  45.193  1.00  0.00           S
ATOM   2820  CE  MET   181      37.852  43.862  46.866  1.00  0.00           C
ATOM   2821  HE1 MET   181      37.056  44.124  47.560  1.00  0.00           H
ATOM   2822  HE2 MET   181      38.213  42.863  47.097  1.00  0.00           H
ATOM   2823  HE3 MET   181      38.668  44.578  46.956  1.00  0.00           H
ATOM   2824  C   MET   181      37.879  48.556  45.287  1.00  0.00           C
ATOM   2825  O   MET   181      38.663  48.850  44.317  1.00  0.00           O
ATOM   2826  N   THR   182      38.284  48.848  46.532  1.00  0.00           N
ATOM   2827  H   THR   182      37.700  48.665  47.337  1.00  0.00           H
ATOM   2828  CA  THR   182      39.669  49.267  46.716  1.00  0.00           C
ATOM   2829  HA  THR   182      40.253  49.275  45.797  1.00  0.00           H
ATOM   2830  CB  THR   182      39.776  50.679  47.322  1.00  0.00           C
ATOM   2831  HB  THR   182      39.226  50.717  48.264  1.00  0.00           H
ATOM   2832  CG2 THR   182      41.230  51.037  47.586  1.00  0.00           C
ATOM   2833 HG21 THR   182      41.292  52.067  47.930  1.00  0.00           H
ATOM   2834 HG22 THR   182      41.617  50.387  48.361  1.00  0.00           H
ATOM   2835 HG23 THR   182      41.824  50.922  46.679  1.00  0.00           H
ATOM   2836  OG1 THR   182      39.208  51.634  46.416  1.00  0.00           O
ATOM   2837  HG1 THR   182      38.253  51.525  46.408  1.00  0.00           H
ATOM   2838  C   THR   182      40.323  48.227  47.634  1.00  0.00           C
ATOM   2839  O   THR   182      39.769  47.917  48.661  1.00  0.00           O
ATOM   2840  N   ILE   183      41.474  47.620  47.258  1.00  0.00           N
ATOM   2841  H   ILE   183      41.884  47.884  46.373  1.00  0.00           H
ATOM   2842  CA  ILE   183      42.165  46.608  48.002  1.00  0.00           C
ATOM   2843  HA  ILE   183      41.486  45.773  48.175  1.00  0.00           H
ATOM   2844  CB  ILE   183      43.382  46.106  47.203  1.00  0.00           C
ATOM   2845  HB  ILE   183      43.763  46.914  46.574  1.00  0.00           H
ATOM   2846  CG2 ILE   183      44.507  45.704  48.145  1.00  0.00           C
ATOM   2847 HG21 ILE   183      45.314  45.266  47.557  1.00  0.00           H
ATOM   2848 HG22 ILE   183      44.944  46.558  48.664  1.00  0.00           H
```

| | | | | | | | | | | |
|---|---|---|---|---|---|---|---|---|---|---|
| ATOM | 2849 | HG23 | ILE | 183 | 44.168 | 44.948 | 48.847 | 1.00 | 0.00 | H |
| ATOM | 2850 | CG1 | ILE | 183 | 42.985 | 44.932 | 46.305 | 1.00 | 0.00 | C |
| ATOM | 2851 | HG12 | ILE | 183 | 43.839 | 44.663 | 45.688 | 1.00 | 0.00 | H |
| ATOM | 2852 | HG13 | ILE | 183 | 42.737 | 44.069 | 46.925 | 1.00 | 0.00 | H |
| ATOM | 2853 | CD1 | ILE | 183 | 41.805 | 45.223 | 45.406 | 1.00 | 0.00 | C |
| ATOM | 2854 | HD11 | ILE | 183 | 41.692 | 44.405 | 44.694 | 1.00 | 0.00 | H |
| ATOM | 2855 | HD12 | ILE | 183 | 40.882 | 45.297 | 45.980 | 1.00 | 0.00 | H |
| ATOM | 2856 | HD13 | ILE | 183 | 41.976 | 46.145 | 44.848 | 1.00 | 0.00 | H |
| ATOM | 2857 | C | ILE | 183 | 42.614 | 47.123 | 49.390 | 1.00 | 0.00 | C |
| ATOM | 2858 | O | ILE | 183 | 42.335 | 46.504 | 50.405 | 1.00 | 0.00 | O |
| ATOM | 2859 | N | ALA | 184 | 43.084 | 48.361 | 49.377 | 1.00 | 0.00 | N |
| ATOM | 2860 | H | ALA | 184 | 43.216 | 48.812 | 48.484 | 1.00 | 0.00 | H |
| ATOM | 2861 | CA | ALA | 184 | 43.457 | 49.108 | 50.540 | 1.00 | 0.00 | C |
| ATOM | 2862 | HA | ALA | 184 | 44.273 | 48.581 | 51.032 | 1.00 | 0.00 | H |
| ATOM | 2863 | CB | ALA | 184 | 43.992 | 50.468 | 50.018 | 1.00 | 0.00 | C |
| ATOM | 2864 | HB1 | ALA | 184 | 43.190 | 51.088 | 49.632 | 1.00 | 0.00 | H |
| ATOM | 2865 | HB2 | ALA | 184 | 44.469 | 50.999 | 50.843 | 1.00 | 0.00 | H |
| ATOM | 2866 | HB3 | ALA | 184 | 44.732 | 50.298 | 49.243 | 1.00 | 0.00 | H |
| ATOM | 2867 | C | ALA | 184 | 42.313 | 49.282 | 51.567 | 1.00 | 0.00 | C |
| ATOM | 2868 | O | ALA | 184 | 42.493 | 49.186 | 52.795 | 1.00 | 0.00 | O |
| ATOM | 2869 | N | GLY | 185 | 41.021 | 49.454 | 51.136 | 1.00 | 0.00 | N |
| ATOM | 2870 | H | GLY | 185 | 40.860 | 49.515 | 50.142 | 1.00 | 0.00 | H |
| ATOM | 2871 | CA | GLY | 185 | 39.790 | 49.431 | 52.011 | 1.00 | 0.00 | C |
| ATOM | 2872 | HA2 | GLY | 185 | 40.030 | 49.903 | 52.965 | 1.00 | 0.00 | H |
| ATOM | 2873 | HA3 | GLY | 185 | 39.037 | 50.049 | 51.523 | 1.00 | 0.00 | H |
| ATOM | 2874 | C | GLY | 185 | 39.164 | 48.098 | 52.289 | 1.00 | 0.00 | C |
| ATOM | 2875 | O | GLY | 185 | 38.627 | 47.895 | 53.346 | 1.00 | 0.00 | O |
| ATOM | 2876 | N | PHE | 186 | 39.432 | 47.079 | 51.444 | 1.00 | 0.00 | N |
| ATOM | 2877 | H | PHE | 186 | 39.969 | 47.304 | 50.619 | 1.00 | 0.00 | H |
| ATOM | 2878 | CA | PHE | 186 | 39.042 | 45.715 | 51.561 | 1.00 | 0.00 | C |

ATOM 2879 HA PHE 186 37.979 45.730 51.790 1.00 0.00 H

ATOM 2880 CB PHE 186 39.299 44.979 50.245 1.00 0.00 C

ATOM 2881 HB2 PHE 186 38.821 45.530 49.435 1.00 0.00 H

ATOM 2882 HB3 PHE 186 40.369 44.945 50.048 1.00 0.00 H

ATOM 2883 CG PHE 186 38.758 43.578 50.218 1.00 0.00 C

ATOM 2884 CD1 PHE 186 37.626 43.242 50.945 1.00 0.00 C

ATOM 2885 HD1 PHE 186 37.097 43.961 51.546 1.00 0.00 H

ATOM 2886 CE1 PHE 186 37.128 41.953 50.921 1.00 0.00 C

ATOM 2887 HE1 PHE 186 36.243 41.701 51.483 1.00 0.00 H

ATOM 2888 CZ PHE 186 37.756 40.984 50.168 1.00 0.00 C

ATOM 2889 HZ PHE 186 37.363 39.978 50.145 1.00 0.00 H

ATOM 2890 CE2 PHE 186 38.884 41.304 49.439 1.00 0.00 C

ATOM 2891 HE2 PHE 186 39.384 40.547 48.855 1.00 0.00 H

ATOM 2892 CD2 PHE 186 39.381 42.593 49.466 1.00 0.00 C

ATOM 2893 HD2 PHE 186 40.262 42.834 48.889 1.00 0.00 H

ATOM 2894 C PHE 186 39.793 45.039 52.702 1.00 0.00 C

ATOM 2895 O PHE 186 39.153 44.303 53.531 1.00 0.00 O

ATOM 2896 N LEU 187 41.074 45.280 52.798 1.00 0.00 N

ATOM 2897 H LEU 187 41.530 45.890 52.133 1.00 0.00 H

ATOM 2898 CA LEU 187 41.856 44.731 53.882 1.00 0.00 C

ATOM 2899 HA LEU 187 41.568 43.690 54.030 1.00 0.00 H

ATOM 2900 CB LEU 187 43.343 44.767 53.510 1.00 0.00 C

ATOM 2901 HB2 LEU 187 43.568 45.699 52.986 1.00 0.00 H

ATOM 2902 HB3 LEU 187 43.917 44.788 54.438 1.00 0.00 H

ATOM 2903 CG LEU 187 43.855 43.571 52.697 1.00 0.00 C

ATOM 2904 HG LEU 187 43.588 42.649 53.206 1.00 0.00 H

ATOM 2905 CD1 LEU 187 43.177 43.556 51.334 1.00 0.00 C

ATOM 2906 HD11 LEU 187 43.568 42.718 50.756 1.00 0.00 H

ATOM 2907 HD12 LEU 187 42.101 43.419 51.414 1.00 0.00 H

ATOM 2908 HD13 LEU 187 43.423 44.464 50.807 1.00 0.00 H

ATOM 2909 CD2 LEU 187 45.367 43.662 52.555 1.00 0.00 C

ATOM 2910 HD21 LEU 187 45.734 42.785 52.021 1.00 0.00 H

ATOM 2911 HD22 LEU 187 45.636 44.567 52.011 1.00 0.00 H

ATOM 2912 HD23 LEU 187 45.838 43.693 53.538 1.00 0.00 H

ATOM 2913 C LEU 187 41.609 45.509 55.210 1.00 0.00 C

ATOM 2914 O LEU 187 41.160 46.667 55.146 1.00 0.00 O

ATOM 2915 N PRO 188 41.770 44.821 56.364 1.00 0.00 N

ATOM 2916 CD PRO 188 42.220 43.413 56.554 1.00 0.00 C

ATOM 2917 HD2 PRO 188 43.152 43.225 56.022 1.00 0.00 H

ATOM 2918 HD3 PRO 188 41.443 42.729 56.223 1.00 0.00 H

ATOM 2919 CG PRO 188 42.438 43.280 58.038 1.00 0.00 C

ATOM 2920 HG2 PRO 188 43.456 43.565 58.298 1.00 0.00 H

ATOM 2921 HG3 PRO 188 42.237 42.259 58.355 1.00 0.00 H

ATOM 2922 CB PRO 188 41.475 44.244 58.642 1.00 0.00 C

ATOM 2923 HB2 PRO 188 41.769 44.524 59.654 1.00 0.00 H

ATOM 2924 HB3 PRO 188 40.478 43.802 58.653 1.00 0.00 H

ATOM 2925 CA PRO 188 41.488 45.439 57.683 1.00 0.00 C

ATOM 2926 HA PRO 188 40.521 45.943 57.688 1.00 0.00 H

ATOM 2927 C PRO 188 42.637 46.455 58.010 1.00 0.00 C

ATOM 2928 O PRO 188 43.814 46.464 57.552 1.00 0.00 O

ATOM 2929 N GLN 189 42.368 47.310 59.015 1.00 0.00 N

ATOM 2930 H GLN 189 41.438 47.274 59.406 1.00 0.00 H

ATOM 2931 CA GLN 189 43.290 48.293 59.649 1.00 0.00 C

ATOM 2932 HA GLN 189 44.273 48.233 59.181 1.00 0.00 H

ATOM 2933 CB GLN 189 42.766 49.720 59.464 1.00 0.00 C

ATOM 2934 HB2 GLN 189 41.764 49.766 59.895 1.00 0.00 H

ATOM 2935 HB3 GLN 189 43.412 50.402 60.019 1.00 0.00 H

ATOM 2936 CG GLN 189 42.718 50.182 58.018 1.00 0.00 C

ATOM 2937 HG2 GLN 189 43.724 50.146 57.598 1.00 0.00 H

ATOM 2938 HG3 GLN 189 42.077 49.510 57.445 1.00 0.00 H

```
ATOM   2939  CD  GLN   189      42.166  51.588  57.878  1.00  0.00           C
ATOM   2940  OE1 GLN   189      42.644  52.313  56.873  1.00  0.00           O
ATOM   2941  NE2 GLN   189      41.319  52.019  58.665  1.00  0.00           N
ATOM   2942 HE21 GLN   189      40.998  51.441  59.426  1.00  0.00           H
ATOM   2943 HE22 GLN   189      40.956  52.951  58.533  1.00  0.00           H
ATOM   2944  C   GLN   189      43.467  47.979  61.164  1.00  0.00           C
ATOM   2945  O   GLN   189      42.831  48.512  62.052  1.00  0.00           O
ATOM   2946  N   SER   190      44.230  46.925  61.501  1.00  0.00           N
ATOM   2947  H   SER   190      44.720  46.461  60.749  1.00  0.00           H
ATOM   2948  CA  SER   190      44.442  46.334  62.871  1.00  0.00           C
ATOM   2949  HA  SER   190      43.481  46.320  63.387  1.00  0.00           H
ATOM   2950  CB  SER   190      44.938  44.905  62.763  1.00  0.00           C
ATOM   2951  HB2 SER   190      44.797  44.409  63.723  1.00  0.00           H
ATOM   2952  HB3 SER   190      44.360  44.367  62.011  1.00  0.00           H
ATOM   2953  OG  SER   190      46.297  44.869  62.424  1.00  0.00           O
ATOM   2954  HG  SER   190      46.669  45.751  62.523  1.00  0.00           H
ATOM   2955  C   SER   190      45.429  47.125  63.706  1.00  0.00           C
ATOM   2956  O   SER   190      46.315  47.827  63.162  1.00  0.00           O
ATOM   2957  N   GLY   191      45.531  46.755  64.974  1.00  0.00           N
ATOM   2958  H   GLY   191      44.877  46.096  65.370  1.00  0.00           H
ATOM   2959  CA  GLY   191      46.516  47.375  65.845  1.00  0.00           C
ATOM   2960  HA2 GLY   191      46.626  48.429  65.584  1.00  0.00           H
ATOM   2961  HA3 GLY   191      46.131  47.328  66.863  1.00  0.00           H
ATOM   2962  C   GLY   191      47.884  46.729  65.841  1.00  0.00           C
ATOM   2963  O   GLY   191      48.755  47.321  66.501  1.00  0.00           O
ATOM   2964  N   SER   192      48.181  45.664  65.080  1.00  0.00           N
ATOM   2965  H   SER   192      47.467  45.248  64.500  1.00  0.00           H
ATOM   2966  CA  SER   192      49.499  45.011  65.118  1.00  0.00           C
ATOM   2967  HA  SER   192      50.261  45.792  65.115  1.00  0.00           H
ATOM   2968  CB  SER   192      49.633  44.236  66.414  1.00  0.00           C
```

| | | | | | | | | | | |
|---|---|---|---|---|---|---|---|---|---|---|
| ATOM | 2969 | HB2 | SER | 192 | 49.537 | 44.916 | 67.261 | 1.00 | 0.00 | H |
| ATOM | 2970 | HB3 | SER | 192 | 48.860 | 43.478 | 66.485 | 1.00 | 0.00 | H |
| ATOM | 2971 | OG | SER | 192 | 50.878 | 43.598 | 66.492 | 1.00 | 0.00 | O |
| ATOM | 2972 | HG | SER | 192 | 50.896 | 42.866 | 65.870 | 1.00 | 0.00 | H |
| ATOM | 2973 | C | SER | 192 | 49.805 | 44.072 | 63.967 | 1.00 | 0.00 | C |
| ATOM | 2974 | O | SER | 192 | 48.951 | 43.438 | 63.383 | 1.00 | 0.00 | O |
| ATOM | 2975 | N | TYR | 193 | 51.159 | 43.963 | 63.606 | 1.00 | 0.00 | N |
| ATOM | 2976 | H | TYR | 193 | 51.851 | 44.480 | 64.130 | 1.00 | 0.00 | H |
| ATOM | 2977 | CA | TYR | 193 | 51.647 | 43.126 | 62.456 | 1.00 | 0.00 | C |
| ATOM | 2978 | HA | TYR | 193 | 50.803 | 42.603 | 62.007 | 1.00 | 0.00 | H |
| ATOM | 2979 | CB | TYR | 193 | 52.273 | 44.012 | 61.377 | 1.00 | 0.00 | C |
| ATOM | 2980 | HB2 | TYR | 193 | 53.017 | 44.662 | 61.834 | 1.00 | 0.00 | H |
| ATOM | 2981 | HB3 | TYR | 193 | 52.785 | 43.376 | 60.654 | 1.00 | 0.00 | H |
| ATOM | 2982 | CG | TYR | 193 | 51.272 | 44.871 | 60.636 | 1.00 | 0.00 | C |
| ATOM | 2983 | CD1 | TYR | 193 | 50.737 | 45.995 | 61.247 | 1.00 | 0.00 | C |
| ATOM | 2984 | HD1 | TYR | 193 | 51.032 | 46.272 | 62.249 | 1.00 | 0.00 | H |
| ATOM | 2985 | CE1 | TYR | 193 | 49.823 | 46.780 | 60.571 | 1.00 | 0.00 | C |
| ATOM | 2986 | HE1 | TYR | 193 | 49.415 | 47.664 | 61.040 | 1.00 | 0.00 | H |
| ATOM | 2987 | CZ | TYR | 193 | 49.444 | 46.438 | 59.278 | 1.00 | 0.00 | C |
| ATOM | 2988 | OH | TYR | 193 | 48.534 | 47.220 | 58.604 | 1.00 | 0.00 | O |
| ATOM | 2989 | HH | TYR | 193 | 48.528 | 47.031 | 57.661 | 1.00 | 0.00 | H |
| ATOM | 2990 | CE2 | TYR | 193 | 49.976 | 45.319 | 58.670 | 1.00 | 0.00 | C |
| ATOM | 2991 | HE2 | TYR | 193 | 49.676 | 45.050 | 57.669 | 1.00 | 0.00 | H |
| ATOM | 2992 | CD2 | TYR | 193 | 50.890 | 44.535 | 59.346 | 1.00 | 0.00 | C |
| ATOM | 2993 | HD2 | TYR | 193 | 51.302 | 43.662 | 58.866 | 1.00 | 0.00 | H |
| ATOM | 2994 | C | TYR | 193 | 52.677 | 42.045 | 62.935 | 1.00 | 0.00 | C |
| ATOM | 2995 | O | TYR | 193 | 53.453 | 42.251 | 63.878 | 1.00 | 0.00 | O |
| ATOM | 2996 | N | PHE | 194 | 52.714 | 40.924 | 62.243 | 1.00 | 0.00 | N |
| ATOM | 2997 | H | PHE | 194 | 52.026 | 40.796 | 61.513 | 1.00 | 0.00 | H |
| ATOM | 2998 | CA | PHE | 194 | 53.791 | 39.844 | 62.349 | 1.00 | 0.00 | C |

| | | | | | | | | | | |
|---|---|---|---|---|---|---|---|---|---|---|
| ATOM | 2999 | HA | PHE | 194 | 54.741 | 40.336 | 62.556 | 1.00 | 0.00 | H |
| ATOM | 3000 | CB | PHE | 194 | 53.480 | 38.912 | 63.521 | 1.00 | 0.00 | C |
| ATOM | 3001 | HB2 | PHE | 194 | 54.125 | 38.033 | 63.486 | 1.00 | 0.00 | H |
| ATOM | 3002 | HB3 | PHE | 194 | 53.722 | 39.437 | 64.443 | 1.00 | 0.00 | H |
| ATOM | 3003 | CG | PHE | 194 | 52.044 | 38.477 | 63.585 | 1.00 | 0.00 | C |
| ATOM | 3004 | CD1 | PHE | 194 | 51.616 | 37.345 | 62.908 | 1.00 | 0.00 | C |
| ATOM | 3005 | HD1 | PHE | 194 | 52.305 | 36.805 | 62.287 | 1.00 | 0.00 | H |
| ATOM | 3006 | CE1 | PHE | 194 | 50.294 | 36.944 | 62.966 | 1.00 | 0.00 | C |
| ATOM | 3007 | HE1 | PHE | 194 | 49.975 | 36.061 | 62.432 | 1.00 | 0.00 | H |
| ATOM | 3008 | CZ | PHE | 194 | 49.384 | 37.672 | 63.704 | 1.00 | 0.00 | C |
| ATOM | 3009 | HZ | PHE | 194 | 48.354 | 37.355 | 63.766 | 1.00 | 0.00 | H |
| ATOM | 3010 | CE2 | PHE | 194 | 49.796 | 38.801 | 64.383 | 1.00 | 0.00 | C |
| ATOM | 3011 | HE2 | PHE | 194 | 49.100 | 39.358 | 64.979 | 1.00 | 0.00 | H |
| ATOM | 3012 | CD2 | PHE | 194 | 51.117 | 39.200 | 64.322 | 1.00 | 0.00 | C |
| ATOM | 3013 | HD2 | PHE | 194 | 51.431 | 40.073 | 64.875 | 1.00 | 0.00 | H |
| ATOM | 3014 | C | PHE | 194 | 53.996 | 38.966 | 61.096 | 1.00 | 0.00 | C |
| ATOM | 3015 | O | PHE | 194 | 53.065 | 38.750 | 60.302 | 1.00 | 0.00 | O |
| ATOM | 3016 | N | ALA | 195 | 55.209 | 38.370 | 60.930 | 1.00 | 0.00 | N |
| ATOM | 3017 | H | ALA | 195 | 55.900 | 38.504 | 61.657 | 1.00 | 0.00 | H |
| ATOM | 3018 | CA | ALA | 195 | 55.680 | 37.778 | 59.722 | 1.00 | 0.00 | C |
| ATOM | 3019 | HA | ALA | 195 | 54.816 | 37.387 | 59.190 | 1.00 | 0.00 | H |
| ATOM | 3020 | CB | ALA | 195 | 56.313 | 38.875 | 58.876 | 1.00 | 0.00 | C |
| ATOM | 3021 | HB1 | ALA | 195 | 56.803 | 38.412 | 58.030 | 1.00 | 0.00 | H |
| ATOM | 3022 | HB2 | ALA | 195 | 55.536 | 39.531 | 58.508 | 1.00 | 0.00 | H |
| ATOM | 3023 | HB3 | ALA | 195 | 57.039 | 39.432 | 59.464 | 1.00 | 0.00 | H |
| ATOM | 3024 | C | ALA | 195 | 56.691 | 36.589 | 59.888 | 1.00 | 0.00 | C |
| ATOM | 3025 | O | ALA | 195 | 57.239 | 36.279 | 60.934 | 1.00 | 0.00 | O |
| ATOM | 3026 | N | TYR | 196 | 56.812 | 35.818 | 58.813 | 1.00 | 0.00 | N |
| ATOM | 3027 | H | TYR | 196 | 56.260 | 36.043 | 57.996 | 1.00 | 0.00 | H |
| ATOM | 3028 | CA | TYR | 196 | 57.921 | 34.866 | 58.622 | 1.00 | 0.00 | C |

| | | | | | | | | | | |
|---|---|---|---|---|---|---|---|---|---|---|
| ATOM | 3029 | HA | TYR | 196 | 58.733 | 35.145 | 59.295 | 1.00 | 0.00 | H |
| ATOM | 3030 | CB | TYR | 196 | 57.472 | 33.453 | 59.003 | 1.00 | 0.00 | C |
| ATOM | 3031 | HB2 | TYR | 196 | 58.355 | 32.826 | 59.139 | 1.00 | 0.00 | H |
| ATOM | 3032 | HB3 | TYR | 196 | 56.952 | 33.498 | 59.958 | 1.00 | 0.00 | H |
| ATOM | 3033 | CG | TYR | 196 | 56.561 | 32.807 | 57.983 | 1.00 | 0.00 | C |
| ATOM | 3034 | CD1 | TYR | 196 | 57.093 | 31.992 | 56.995 | 1.00 | 0.00 | C |
| ATOM | 3035 | HD1 | TYR | 196 | 58.157 | 31.818 | 56.948 | 1.00 | 0.00 | H |
| ATOM | 3036 | CE1 | TYR | 196 | 56.261 | 31.401 | 56.064 | 1.00 | 0.00 | C |
| ATOM | 3037 | HE1 | TYR | 196 | 56.672 | 30.757 | 55.300 | 1.00 | 0.00 | H |
| ATOM | 3038 | CZ | TYR | 196 | 54.891 | 31.627 | 56.123 | 1.00 | 0.00 | C |
| ATOM | 3039 | OH | TYR | 196 | 54.062 | 31.038 | 55.195 | 1.00 | 0.00 | O |
| ATOM | 3040 | HH | TYR | 196 | 54.539 | 30.556 | 54.511 | 1.00 | 0.00 | H |
| ATOM | 3041 | CE2 | TYR | 196 | 54.361 | 32.438 | 57.106 | 1.00 | 0.00 | C |
| ATOM | 3042 | HE2 | TYR | 196 | 53.303 | 32.638 | 57.139 | 1.00 | 0.00 | H |
| ATOM | 3043 | CD2 | TYR | 196 | 55.193 | 33.028 | 58.037 | 1.00 | 0.00 | C |
| ATOM | 3044 | HD2 | TYR | 196 | 54.776 | 33.629 | 58.817 | 1.00 | 0.00 | H |
| ATOM | 3045 | C | TYR | 196 | 58.489 | 34.834 | 57.230 | 1.00 | 0.00 | C |
| ATOM | 3046 | O | TYR | 196 | 57.884 | 35.311 | 56.300 | 1.00 | 0.00 | O |
| ATOM | 3047 | N | ASN | 197 | 59.798 | 34.332 | 57.038 | 1.00 | 0.00 | N |
| ATOM | 3048 | H | ASN | 197 | 60.301 | 33.989 | 57.844 | 1.00 | 0.00 | H |
| ATOM | 3049 | CA | ASN | 197 | 60.444 | 34.107 | 55.726 | 1.00 | 0.00 | C |
| ATOM | 3050 | HA | ASN | 197 | 59.902 | 34.666 | 54.964 | 1.00 | 0.00 | H |
| ATOM | 3051 | CB | ASN | 197 | 61.884 | 34.587 | 55.738 | 1.00 | 0.00 | C |
| ATOM | 3052 | HB2 | ASN | 197 | 62.454 | 34.011 | 56.469 | 1.00 | 0.00 | H |
| ATOM | 3053 | HB3 | ASN | 197 | 62.317 | 34.418 | 54.751 | 1.00 | 0.00 | H |
| ATOM | 3054 | CG | ASN | 197 | 62.002 | 36.051 | 56.060 | 1.00 | 0.00 | C |
| ATOM | 3055 | OD1 | ASN | 197 | 62.996 | 36.399 | 56.837 | 1.00 | 0.00 | O |
| ATOM | 3056 | ND2 | ASN | 197 | 61.193 | 36.867 | 55.604 | 1.00 | 0.00 | N |
| ATOM | 3057 | HD21 | ASN | 197 | 61.314 | 37.846 | 55.810 | 1.00 | 0.00 | H |
| ATOM | 3058 | HD22 | ASN | 197 | 60.432 | 36.553 | 55.023 | 1.00 | 0.00 | H |

```
ATOM   3059  C   ASN   197      60.363  32.609  55.356  1.00  0.00           C
ATOM   3060  O   ASN   197      60.782  31.826  56.187  1.00  0.00           O
ATOM   3061  N   GLY   198      59.918  32.276  54.163  1.00  0.00           N
ATOM   3062  H   GLY   198      59.609  33.017  53.548  1.00  0.00           H
ATOM   3063  CA  GLY   198      59.795  30.937  53.649  1.00  0.00           C
ATOM   3064  HA2 GLY   198      60.735  30.415  53.798  1.00  0.00           H
ATOM   3065  HA3 GLY   198      59.064  30.421  54.272  1.00  0.00           H
ATOM   3066  C   GLY   198      59.340  30.728  52.134  1.00  0.00           C
ATOM   3067  O   GLY   198      59.507  31.618  51.337  1.00  0.00           O
ATOM   3068  N   SER   199      58.966  29.486  51.743  1.00  0.00           N
ATOM   3069  H   SER   199      58.933  28.771  52.456  1.00  0.00           H
ATOM   3070  CA  SER   199      58.547  29.051  50.401  1.00  0.00           C
ATOM   3071  HA  SER   199      59.219  29.478  49.673  1.00  0.00           H
ATOM   3072  CB  SER   199      58.661  27.542  50.315  1.00  0.00           C
ATOM   3073  HB2 SER   199      58.388  27.218  49.310  1.00  0.00           H
ATOM   3074  HB3 SER   199      59.688  27.235  50.511  1.00  0.00           H
ATOM   3075  OG  SER   199      57.813  26.921  51.242  1.00  0.00           O
ATOM   3076  HG  SER   199      58.246  26.949  52.102  1.00  0.00           H
ATOM   3077  C   SER   199      57.100  29.487  50.014  1.00  0.00           C
ATOM   3078  O   SER   199      56.267  29.810  50.850  1.00  0.00           O
ATOM   3079  N   LEU   200      56.816  29.519  48.735  1.00  0.00           N
ATOM   3080  H   LEU   200      57.533  29.286  48.062  1.00  0.00           H
ATOM   3081  CA  LEU   200      55.426  29.828  48.221  1.00  0.00           C
ATOM   3082  HA  LEU   200      55.053  30.738  48.687  1.00  0.00           H
ATOM   3083  CB  LEU   200      55.380  29.964  46.694  1.00  0.00           C
ATOM   3084  HB2 LEU   200      55.850  29.093  46.256  1.00  0.00           H
ATOM   3085  HB3 LEU   200      54.337  29.962  46.373  1.00  0.00           H
ATOM   3086  CG  LEU   200      56.070  31.206  46.116  1.00  0.00           C
ATOM   3087  HG  LEU   200      57.069  31.297  46.542  1.00  0.00           H
ATOM   3088  CD1 LEU   200      56.209  31.054  44.607  1.00  0.00           C
```

ATOM 3089 HD11 LEU 200 56.702 31.934 44.194 1.00 0.00 H

ATOM 3090 HD12 LEU 200 56.813 30.176 44.374 1.00 0.00 H

ATOM 3091 HD13 LEU 200 55.226 30.945 44.146 1.00 0.00 H

ATOM 3092 CD2 LEU 200 55.264 32.446 46.468 1.00 0.00 C

ATOM 3093 HD21 LEU 200 55.761 33.326 46.057 1.00 0.00 H

ATOM 3094 HD22 LEU 200 54.264 32.380 46.035 1.00 0.00 H

ATOM 3095 HD23 LEU 200 55.176 32.572 47.546 1.00 0.00 H

ATOM 3096 C LEU 200 54.567 28.646 48.688 1.00 0.00 C

ATOM 3097 O LEU 200 54.986 27.466 48.750 1.00 0.00 O

ATOM 3098 N THR 201 53.302 28.890 49.031 1.00 0.00 N

ATOM 3099 H THR 201 52.952 29.836 48.970 1.00 0.00 H

ATOM 3100 CA THR 201 52.374 27.886 49.467 1.00 0.00 C

ATOM 3101 HA THR 201 52.864 27.298 50.245 1.00 0.00 H

ATOM 3102 CB THR 201 51.190 28.660 50.158 1.00 0.00 C

ATOM 3103 HB THR 201 50.526 27.894 50.560 1.00 0.00 H

ATOM 3104 CG2 THR 201 51.522 29.579 51.333 1.00 0.00 C

ATOM 3105 HG21 THR 201 51.841 28.982 52.181 1.00 0.00 H

ATOM 3106 HG22 THR 201 52.322 30.267 51.068 1.00 0.00 H

ATOM 3107 HG23 THR 201 50.639 30.156 51.611 1.00 0.00 H

ATOM 3108 OG1 THR 201 50.410 29.382 49.228 1.00 0.00 O

ATOM 3109 HG1 THR 201 49.521 29.474 49.598 1.00 0.00 H

ATOM 3110 C THR 201 51.842 26.917 48.457 1.00 0.00 C

ATOM 3111 O THR 201 51.386 25.847 48.824 1.00 0.00 O

ATOM 3112 N THR 202 51.920 27.277 47.198 1.00 0.00 N

ATOM 3113 H THR 202 52.279 28.203 47.015 1.00 0.00 H

ATOM 3114 CA THR 202 51.585 26.493 45.984 1.00 0.00 C

ATOM 3115 HA THR 202 51.425 25.458 46.280 1.00 0.00 H

ATOM 3116 CB THR 202 50.286 27.010 45.338 1.00 0.00 C

ATOM 3117 HB THR 202 49.961 26.330 44.554 1.00 0.00 H

ATOM 3118 CG2 THR 202 49.176 27.103 46.374 1.00 0.00 C

| | | | | | | | | | | |
|---|---|---|---|---|---|---|---|---|---|---|
| ATOM | 3119 | HG21 | THR | 202 | 48.252 | 27.293 | 45.832 | 1.00 | 0.00 | H |
| ATOM | 3120 | HG22 | THR | 202 | 49.082 | 26.170 | 46.928 | 1.00 | 0.00 | H |
| ATOM | 3121 | HG23 | THR | 202 | 49.347 | 27.928 | 47.066 | 1.00 | 0.00 | H |
| ATOM | 3122 | OG1 | THR | 202 | 50.514 | 28.306 | 44.770 | 1.00 | 0.00 | O |
| ATOM | 3123 | HG1 | THR | 202 | 51.093 | 28.216 | 44.009 | 1.00 | 0.00 | H |
| ATOM | 3124 | C | THR | 202 | 52.730 | 26.528 | 44.939 | 1.00 | 0.00 | C |
| ATOM | 3125 | O | THR | 202 | 53.724 | 27.240 | 45.067 | 1.00 | 0.00 | O |
| ATOM | 3126 | N | PRO | 203 | 52.790 | 25.628 | 43.928 | 1.00 | 0.00 | N |
| ATOM | 3127 | CD | PRO | 203 | 51.749 | 24.610 | 43.660 | 1.00 | 0.00 | C |
| ATOM | 3128 | HD2 | PRO | 203 | 50.900 | 25.067 | 43.151 | 1.00 | 0.00 | H |
| ATOM | 3129 | HD3 | PRO | 203 | 51.437 | 24.095 | 44.565 | 1.00 | 0.00 | H |
| ATOM | 3130 | CG | PRO | 203 | 52.446 | 23.663 | 42.720 | 1.00 | 0.00 | C |
| ATOM | 3131 | HG2 | PRO | 203 | 51.733 | 23.107 | 42.117 | 1.00 | 0.00 | H |
| ATOM | 3132 | HG3 | PRO | 203 | 53.061 | 22.971 | 43.296 | 1.00 | 0.00 | H |
| ATOM | 3133 | CB | PRO | 203 | 53.320 | 24.540 | 41.890 | 1.00 | 0.00 | C |
| ATOM | 3134 | HB2 | PRO | 203 | 52.716 | 25.023 | 41.119 | 1.00 | 0.00 | H |
| ATOM | 3135 | HB3 | PRO | 203 | 54.133 | 23.971 | 41.440 | 1.00 | 0.00 | H |
| ATOM | 3136 | CA | PRO | 203 | 53.863 | 25.575 | 42.880 | 1.00 | 0.00 | C |
| ATOM | 3137 | HA | PRO | 203 | 54.724 | 25.124 | 43.349 | 1.00 | 0.00 | H |
| ATOM | 3138 | C | PRO | 203 | 54.154 | 26.843 | 42.163 | 1.00 | 0.00 | C |
| ATOM | 3139 | O | PRO | 203 | 53.213 | 27.646 | 41.971 | 1.00 | 0.00 | O |
| ATOM | 3140 | N | PRO | 204 | 55.411 | 27.166 | 41.855 | 1.00 | 0.00 | N |
| ATOM | 3141 | CD | PRO | 204 | 55.822 | 28.434 | 41.201 | 1.00 | 0.00 | C |
| ATOM | 3142 | HD2 | PRO | 204 | 55.487 | 29.302 | 41.770 | 1.00 | 0.00 | H |
| ATOM | 3143 | HD3 | PRO | 204 | 55.431 | 28.464 | 40.183 | 1.00 | 0.00 | H |
| ATOM | 3144 | CG | PRO | 204 | 57.351 | 28.358 | 41.178 | 1.00 | 0.00 | C |
| ATOM | 3145 | HG2 | PRO | 204 | 57.749 | 28.761 | 42.110 | 1.00 | 0.00 | H |
| ATOM | 3146 | HG3 | PRO | 204 | 57.771 | 28.880 | 40.318 | 1.00 | 0.00 | H |
| ATOM | 3147 | CB | PRO | 204 | 57.592 | 26.868 | 41.109 | 1.00 | 0.00 | C |
| ATOM | 3148 | HB2 | PRO | 204 | 58.594 | 26.586 | 41.425 | 1.00 | 0.00 | H |

```
ATOM   3149  HB3 PRO   204      57.444  26.538  40.079  1.00  0.00           H
ATOM   3150  CA  PRO   204      56.519  26.282  41.960  1.00  0.00           C
ATOM   3151  HA  PRO   204      56.236  25.336  41.496  1.00  0.00           H
ATOM   3152  C   PRO   204      56.953  25.932  43.418  1.00  0.00           C
ATOM   3153  O   PRO   204      57.938  25.268  43.566  1.00  0.00           O
ATOM   3154  N   CYS   205      56.400  26.513  44.498  1.00  0.00           N
ATOM   3155  H   CYS   205      55.586  27.094  44.353  1.00  0.00           H
ATOM   3156  CA  CYS   205      56.898  26.460  45.829  1.00  0.00           C
ATOM   3157  HA  CYS   205      56.355  27.165  46.446  1.00  0.00           H
ATOM   3158  CB  CYS   205      56.642  25.070  46.412  1.00  0.00           C
ATOM   3159  HB2 CYS   205      57.161  24.318  45.818  1.00  0.00           H
ATOM   3160  HB3 CYS   205      57.047  25.035  47.424  1.00  0.00           H
ATOM   3161  SG  CYS   205      54.892  24.623  46.512  1.00  0.00           S
ATOM   3162  HG  CYS   205      54.553  25.595  47.358  1.00  0.00           H
ATOM   3163  C   CYS   205      58.396  26.788  45.918  1.00  0.00           C
ATOM   3164  O   CYS   205      59.180  26.060  46.468  1.00  0.00           O
ATOM   3165  N   THR   206      58.880  27.874  45.353  1.00  0.00           N
ATOM   3166  H   THR   206      58.248  28.461  44.829  1.00  0.00           H
ATOM   3167  CA  THR   206      60.184  28.383  45.556  1.00  0.00           C
ATOM   3168  HA  THR   206      60.874  27.558  45.738  1.00  0.00           H
ATOM   3169  CB  THR   206      60.661  29.077  44.267  1.00  0.00           C
ATOM   3170  HB  THR   206      61.674  29.455  44.411  1.00  0.00           H
ATOM   3171  CG2 THR   206      60.662  28.099  43.103  1.00  0.00           C
ATOM   3172 HG21 THR   206      59.668  27.698  42.940  1.00  0.00           H
ATOM   3173 HG22 THR   206      60.995  28.606  42.197  1.00  0.00           H
ATOM   3174 HG23 THR   206      61.345  27.276  43.317  1.00  0.00           H
ATOM   3175  OG1 THR   206      59.791  30.176  43.962  1.00  0.00           O
ATOM   3176  HG1 THR   206      59.895  30.844  44.645  1.00  0.00           H
ATOM   3177  C   THR   206      60.293  29.357  46.763  1.00  0.00           C
ATOM   3178  O   THR   206      59.321  29.871  47.262  1.00  0.00           O
```

| | | | | | | | | | | |
|---|---|---|---|---|---|---|---|---|---|---|
| ATOM | 3179 | N | GLU | 207 | 61.511 | 29.493 | 47.287 | 1.00 | 0.00 | N |
| ATOM | 3180 | H | GLU | 207 | 62.282 | 29.024 | 46.835 | 1.00 | 0.00 | H |
| ATOM | 3181 | CA | GLU | 207 | 61.817 | 30.164 | 48.516 | 1.00 | 0.00 | C |
| ATOM | 3182 | HA | GLU | 207 | 60.964 | 30.131 | 49.180 | 1.00 | 0.00 | H |
| ATOM | 3183 | CB | GLU | 207 | 62.951 | 29.403 | 49.206 | 1.00 | 0.00 | C |
| ATOM | 3184 | HB2 | GLU | 207 | 63.812 | 29.408 | 48.535 | 1.00 | 0.00 | H |
| ATOM | 3185 | HB3 | GLU | 207 | 63.228 | 29.928 | 50.121 | 1.00 | 0.00 | H |
| ATOM | 3186 | CG | GLU | 207 | 62.611 | 27.967 | 49.578 | 1.00 | 0.00 | C |
| ATOM | 3187 | HG2 | GLU | 207 | 61.817 | 27.985 | 50.325 | 1.00 | 0.00 | H |
| ATOM | 3188 | HG3 | GLU | 207 | 62.247 | 27.429 | 48.702 | 1.00 | 0.00 | H |
| ATOM | 3189 | CD | GLU | 207 | 63.787 | 27.211 | 50.128 | 1.00 | 0.00 | C |
| ATOM | 3190 | OE1 | GLU | 207 | 64.882 | 27.714 | 50.051 | 1.00 | 0.00 | O |
| ATOM | 3191 | OE2 | GLU | 207 | 63.591 | 26.127 | 50.627 | 1.00 | 0.00 | O |
| ATOM | 3192 | C | GLU | 207 | 62.199 | 31.680 | 48.359 | 1.00 | 0.00 | C |
| ATOM | 3193 | O | GLU | 207 | 62.376 | 32.196 | 47.293 | 1.00 | 0.00 | O |
| ATOM | 3194 | N | GLY | 208 | 62.302 | 32.429 | 49.435 | 1.00 | 0.00 | N |
| ATOM | 3195 | H | GLY | 208 | 62.133 | 31.952 | 50.308 | 1.00 | 0.00 | H |
| ATOM | 3196 | CA | GLY | 208 | 62.623 | 33.812 | 49.589 | 1.00 | 0.00 | C |
| ATOM | 3197 | HA2 | GLY | 208 | 63.162 | 33.926 | 50.529 | 1.00 | 0.00 | H |
| ATOM | 3198 | HA3 | GLY | 208 | 63.287 | 34.122 | 48.780 | 1.00 | 0.00 | H |
| ATOM | 3199 | C | GLY | 208 | 61.419 | 34.724 | 49.622 | 1.00 | 0.00 | C |
| ATOM | 3200 | O | GLY | 208 | 61.443 | 35.705 | 48.915 | 1.00 | 0.00 | O |
| ATOM | 3201 | N | VAL | 209 | 60.349 | 34.317 | 50.258 | 1.00 | 0.00 | N |
| ATOM | 3202 | H | VAL | 209 | 60.355 | 33.438 | 50.757 | 1.00 | 0.00 | H |
| ATOM | 3203 | CA | VAL | 209 | 59.079 | 35.138 | 50.267 | 1.00 | 0.00 | C |
| ATOM | 3204 | HA | VAL | 209 | 59.187 | 36.015 | 49.631 | 1.00 | 0.00 | H |
| ATOM | 3205 | CB | VAL | 209 | 57.872 | 34.323 | 49.767 | 1.00 | 0.00 | C |
| ATOM | 3206 | HB | VAL | 209 | 57.718 | 33.469 | 50.423 | 1.00 | 0.00 | H |
| ATOM | 3207 | CG1 | VAL | 209 | 56.613 | 35.178 | 49.773 | 1.00 | 0.00 | C |
| ATOM | 3208 | HG11 | VAL | 209 | 55.789 | 34.608 | 49.344 | 1.00 | 0.00 | H |

ATOM 3209 HG12 VAL 209 56.336 35.443 50.793 1.00 0.00 H
ATOM 3210 HG13 VAL 209 56.766 36.086 49.189 1.00 0.00 H
ATOM 3211 CG2 VAL 209 58.152 33.787 48.371 1.00 0.00 C
ATOM 3212 HG21 VAL 209 58.360 34.602 47.680 1.00 0.00 H
ATOM 3213 HG22 VAL 209 59.001 33.103 48.385 1.00 0.00 H
ATOM 3214 HG23 VAL 209 57.293 33.227 48.023 1.00 0.00 H
ATOM 3215 C VAL 209 58.843 35.600 51.742 1.00 0.00 C
ATOM 3216 O VAL 209 58.941 34.723 52.586 1.00 0.00 O
ATOM 3217 N LEU 210 58.556 36.863 51.957 1.00 0.00 N
ATOM 3218 H LEU 210 58.476 37.491 51.178 1.00 0.00 H
ATOM 3219 CA LEU 210 58.169 37.358 53.243 1.00 0.00 C
ATOM 3220 HA LEU 210 58.540 36.695 54.025 1.00 0.00 H
ATOM 3221 CB LEU 210 58.740 38.762 53.477 1.00 0.00 C
ATOM 3222 HB2 LEU 210 59.826 38.692 53.560 1.00 0.00 H
ATOM 3223 HB3 LEU 210 58.520 39.359 52.590 1.00 0.00 H
ATOM 3224 CG LEU 210 58.179 39.515 54.691 1.00 0.00 C
ATOM 3225 HG LEU 210 57.093 39.542 54.637 1.00 0.00 H
ATOM 3226 CD1 LEU 210 58.593 38.798 55.969 1.00 0.00 C
ATOM 3227 HD11 LEU 210 58.130 39.356 56.771 1.00 0.00 H
ATOM 3228 HD12 LEU 210 58.225 37.777 55.990 1.00 0.00 H
ATOM 3229 HD13 LEU 210 59.671 38.838 56.089 1.00 0.00 H
ATOM 3230 CD2 LEU 210 58.687 40.949 54.679 1.00 0.00 C
ATOM 3231 HD21 LEU 210 58.306 41.486 55.549 1.00 0.00 H
ATOM 3232 HD22 LEU 210 59.777 40.962 54.696 1.00 0.00 H
ATOM 3233 HD23 LEU 210 58.342 41.458 53.779 1.00 0.00 H
ATOM 3234 C LEU 210 56.605 37.355 53.263 1.00 0.00 C
ATOM 3235 O LEU 210 55.992 37.844 52.340 1.00 0.00 O
ATOM 3236 N PHE 211 56.031 36.769 54.316 1.00 0.00 N
ATOM 3237 H PHE 211 56.629 36.379 55.031 1.00 0.00 H
ATOM 3238 CA PHE 211 54.571 36.634 54.537 1.00 0.00 C

ATOM 3239 HA PHE 211 54.026 36.991 53.663 1.00 0.00 H

ATOM 3240 CB PHE 211 54.167 35.182 54.799 1.00 0.00 C

ATOM 3241 HB2 PHE 211 54.740 34.809 55.648 1.00 0.00 H

ATOM 3242 HB3 PHE 211 53.114 35.153 55.079 1.00 0.00 H

ATOM 3243 CG PHE 211 54.387 34.271 53.625 1.00 0.00 C

ATOM 3244 CD1 PHE 211 55.615 33.657 53.426 1.00 0.00 C

ATOM 3245 HD1 PHE 211 56.420 33.820 54.128 1.00 0.00 H

ATOM 3246 CE1 PHE 211 55.818 32.818 52.346 1.00 0.00 C

ATOM 3247 HE1 PHE 211 56.784 32.361 52.194 1.00 0.00 H

ATOM 3248 CZ PHE 211 54.795 32.584 51.453 1.00 0.00 C

ATOM 3249 HZ PHE 211 54.949 31.940 50.603 1.00 0.00 H

ATOM 3250 CE2 PHE 211 53.568 33.188 51.638 1.00 0.00 C

ATOM 3251 HE2 PHE 211 52.760 32.998 50.945 1.00 0.00 H

ATOM 3252 CD2 PHE 211 53.368 34.026 52.718 1.00 0.00 C

ATOM 3253 HD2 PHE 211 52.413 34.516 52.833 1.00 0.00 H

ATOM 3254 C PHE 211 54.228 37.528 55.752 1.00 0.00 C

ATOM 3255 O PHE 211 54.836 37.349 56.828 1.00 0.00 O

ATOM 3256 N VAL 212 53.347 38.491 55.614 1.00 0.00 N

ATOM 3257 H VAL 212 52.875 38.609 54.727 1.00 0.00 H

ATOM 3258 CA VAL 212 53.058 39.469 56.689 1.00 0.00 C

ATOM 3259 HA VAL 212 53.595 39.220 57.602 1.00 0.00 H

ATOM 3260 CB VAL 212 53.394 40.915 56.279 1.00 0.00 C

ATOM 3261 HB VAL 212 52.783 41.189 55.419 1.00 0.00 H

ATOM 3262 CG1 VAL 212 53.088 41.876 57.418 1.00 0.00 C

ATOM 3263 HG11 VAL 212 53.446 42.878 57.181 1.00 0.00 H

ATOM 3264 HG12 VAL 212 52.011 41.920 57.573 1.00 0.00 H

ATOM 3265 HG13 VAL 212 53.558 41.534 58.337 1.00 0.00 H

ATOM 3266 CG2 VAL 212 54.856 41.011 55.870 1.00 0.00 C

ATOM 3267 HG21 VAL 212 55.504 40.565 56.617 1.00 0.00 H

ATOM 3268 HG22 VAL 212 55.001 40.497 54.920 1.00 0.00 H

ATOM 3269 HG23 VAL 212 55.142 42.054 55.735 1.00 0.00 H
ATOM 3270 C VAL 212 51.539 39.285 56.919 1.00 0.00 C
ATOM 3271 O VAL 212 50.687 39.166 56.045 1.00 0.00 O
ATOM 3272 N VAL 213 51.264 39.154 58.218 1.00 0.00 N
ATOM 3273 H VAL 213 52.015 39.221 58.887 1.00 0.00 H
ATOM 3274 CA VAL 213 49.941 39.003 58.730 1.00 0.00 C
ATOM 3275 HA VAL 213 49.252 38.854 57.901 1.00 0.00 H
ATOM 3276 CB VAL 213 49.858 37.742 59.611 1.00 0.00 C
ATOM 3277 HB VAL 213 50.602 37.813 60.401 1.00 0.00 H
ATOM 3278 CG1 VAL 213 48.483 37.631 60.253 1.00 0.00 C
ATOM 3279 HG11 VAL 213 48.398 36.656 60.724 1.00 0.00 H
ATOM 3280 HG12 VAL 213 48.347 38.384 61.028 1.00 0.00 H
ATOM 3281 HG13 VAL 213 47.700 37.723 59.499 1.00 0.00 H
ATOM 3282 CG2 VAL 213 50.166 36.507 58.778 1.00 0.00 C
ATOM 3283 HG21 VAL 213 50.123 35.631 59.422 1.00 0.00 H
ATOM 3284 HG22 VAL 213 49.403 36.383 58.017 1.00 0.00 H
ATOM 3285 HG23 VAL 213 51.156 36.563 58.326 1.00 0.00 H
ATOM 3286 C VAL 213 49.476 40.137 59.517 1.00 0.00 C
ATOM 3287 O VAL 213 50.132 40.720 60.367 1.00 0.00 O
ATOM 3288 N LEU 214 48.229 40.525 59.211 1.00 0.00 N
ATOM 3289 H LEU 214 47.733 40.027 58.486 1.00 0.00 H
ATOM 3290 CA LEU 214 47.481 41.615 59.928 1.00 0.00 C
ATOM 3291 HA LEU 214 48.181 42.337 60.353 1.00 0.00 H
ATOM 3292 CB LEU 214 46.517 42.354 58.993 1.00 0.00 C
ATOM 3293 HB2 LEU 214 45.937 41.621 58.429 1.00 0.00 H
ATOM 3294 HB3 LEU 214 45.816 42.906 59.621 1.00 0.00 H
ATOM 3295 CG LEU 214 47.162 43.363 58.033 1.00 0.00 C
ATOM 3296 HG LEU 214 47.692 44.108 58.619 1.00 0.00 H
ATOM 3297 CD1 LEU 214 48.134 42.638 57.112 1.00 0.00 C
ATOM 3298 HD11 LEU 214 48.407 43.291 56.283 1.00 0.00 H

ATOM 3299 HD12 LEU 214 49.054 42.398 57.644 1.00 0.00 H

ATOM 3300 HD13 LEU 214 47.688 41.731 56.705 1.00 0.00 H

ATOM 3301 CD2 LEU 214 46.078 44.071 57.236 1.00 0.00 C

ATOM 3302 HD21 LEU 214 46.527 44.779 56.540 1.00 0.00 H

ATOM 3303 HD22 LEU 214 45.477 43.354 56.681 1.00 0.00 H

ATOM 3304 HD23 LEU 214 45.445 44.623 57.926 1.00 0.00 H

ATOM 3305 C LEU 214 46.705 40.921 61.085 1.00 0.00 C

ATOM 3306 O LEU 214 45.940 39.994 60.940 1.00 0.00 O

ATOM 3307 N THR 215 46.892 41.270 62.305 1.00 0.00 N

ATOM 3308 H THR 215 47.529 42.036 62.478 1.00 0.00 H

ATOM 3309 CA THR 215 46.381 40.586 63.483 1.00 0.00 C

ATOM 3310 HA THR 215 46.666 39.537 63.392 1.00 0.00 H

ATOM 3311 CB THR 215 47.060 41.143 64.748 1.00 0.00 C

ATOM 3312 HB THR 215 48.124 41.284 64.560 1.00 0.00 H

ATOM 3313 CG2 THR 215 46.443 42.476 65.142 1.00 0.00 C

ATOM 3314 HG21 THR 215 46.958 42.848 66.022 1.00 0.00 H

ATOM 3315 HG22 THR 215 46.586 43.191 64.342 1.00 0.00 H

ATOM 3316 HG23 THR 215 45.391 42.440 65.410 1.00 0.00 H

ATOM 3317 OG1 THR 215 46.904 40.211 65.826 1.00 0.00 O

ATOM 3318 HG1 THR 215 45.961 40.077 65.963 1.00 0.00 H

ATOM 3319 C THR 215 44.906 40.632 63.702 1.00 0.00 C

ATOM 3320 O THR 215 44.445 40.346 64.867 1.00 0.00 O

ATOM 3321 N SER 216 44.077 40.855 62.685 1.00 0.00 N

ATOM 3322 H SER 216 44.434 41.011 61.753 1.00 0.00 H

ATOM 3323 CA SER 216 42.588 40.938 62.925 1.00 0.00 C

ATOM 3324 HA SER 216 42.300 40.192 63.665 1.00 0.00 H

ATOM 3325 CB SER 216 42.164 42.307 63.418 1.00 0.00 C

ATOM 3326 HB2 SER 216 42.656 42.513 64.369 1.00 0.00 H

ATOM 3327 HB3 SER 216 42.462 43.068 62.696 1.00 0.00 H

ATOM 3328 OG SER 216 40.776 42.368 63.602 1.00 0.00 O

ATOM 3329 HG SER 216 40.343 42.345 62.743 1.00 0.00 H

ATOM 3330 C SER 216 41.914 40.624 61.593 1.00 0.00 C

ATOM 3331 O SER 216 42.400 41.266 60.640 1.00 0.00 O

ATOM 3332 N PRO 217 40.864 39.778 61.454 1.00 0.00 N

ATOM 3333 CD PRO 217 40.321 38.945 62.537 1.00 0.00 C

ATOM 3334 HD2 PRO 217 40.088 39.534 63.425 1.00 0.00 H

ATOM 3335 HD3 PRO 217 41.020 38.141 62.771 1.00 0.00 H

ATOM 3336 CG PRO 217 39.064 38.386 61.926 1.00 0.00 C

ATOM 3337 HG2 PRO 217 38.237 39.067 62.127 1.00 0.00 H

ATOM 3338 HG3 PRO 217 38.838 37.392 62.314 1.00 0.00 H

ATOM 3339 CB PRO 217 39.344 38.358 60.462 1.00 0.00 C

ATOM 3340 HB2 PRO 217 38.435 38.351 59.865 1.00 0.00 H

ATOM 3341 HB3 PRO 217 39.928 37.469 60.236 1.00 0.00 H

ATOM 3342 CA PRO 217 40.181 39.617 60.215 1.00 0.00 C

ATOM 3343 HA PRO 217 40.906 39.437 59.424 1.00 0.00 H

ATOM 3344 C PRO 217 39.318 40.785 59.868 1.00 0.00 C

ATOM 3345 O PRO 217 39.046 41.762 60.649 1.00 0.00 O

ATOM 3346 N GLN 218 39.077 40.984 58.583 1.00 0.00 N

ATOM 3347 H GLN 218 39.437 40.303 57.928 1.00 0.00 H

ATOM 3348 CA GLN 218 38.079 41.940 58.052 1.00 0.00 C

ATOM 3349 HA GLN 218 38.205 42.886 58.579 1.00 0.00 H

ATOM 3350 CB GLN 218 38.340 42.194 56.565 1.00 0.00 C

ATOM 3351 HB2 GLN 218 39.343 42.593 56.442 1.00 0.00 H

ATOM 3352 HB3 GLN 218 38.283 41.245 56.043 1.00 0.00 H

ATOM 3353 CG GLN 218 37.356 43.151 55.915 1.00 0.00 C

ATOM 3354 HG2 GLN 218 37.554 43.177 54.850 1.00 0.00 H

ATOM 3355 HG3 GLN 218 36.337 42.779 55.989 1.00 0.00 H

ATOM 3356 CD GLN 218 37.433 44.548 56.502 1.00 0.00 C

ATOM 3357 OE1 GLN 218 36.789 44.849 57.511 1.00 0.00 O

ATOM 3358 NE2 GLN 218 38.223 45.410 55.873 1.00 0.00 N

ATOM 3359 HE21 GLN 218 38.732 45.122 55.052 1.00 0.00 H

ATOM 3360 HE22 GLN 218 38.335 46.348 56.223 1.00 0.00 H

ATOM 3361 C GLN 218 36.625 41.485 58.236 1.00 0.00 C

ATOM 3362 O GLN 218 36.333 40.276 58.016 1.00 0.00 O

ATOM 3363 N ILE 219 35.672 42.385 58.414 1.00 0.00 N

ATOM 3364 H ILE 219 35.942 43.356 58.488 1.00 0.00 H

ATOM 3365 CA ILE 219 34.249 42.090 58.564 1.00 0.00 C

ATOM 3366 HA ILE 219 34.165 41.156 59.121 1.00 0.00 H

ATOM 3367 CB ILE 219 33.605 43.207 59.406 1.00 0.00 C

ATOM 3368 HB ILE 219 33.659 44.156 58.868 1.00 0.00 H

ATOM 3369 CG2 ILE 219 32.142 42.891 59.678 1.00 0.00 C

ATOM 3370 HG21 ILE 219 31.767 43.527 60.481 1.00 0.00 H

ATOM 3371 HG22 ILE 219 31.524 43.088 58.806 1.00 0.00 H

ATOM 3372 HG23 ILE 219 32.021 41.849 59.972 1.00 0.00 H

ATOM 3373 CG1 ILE 219 34.370 43.394 60.719 1.00 0.00 C

ATOM 3374 HG12 ILE 219 35.377 43.767 60.531 1.00 0.00 H

ATOM 3375 HG13 ILE 219 33.865 44.162 61.306 1.00 0.00 H

ATOM 3376 CD1 ILE 219 34.454 42.141 61.560 1.00 0.00 C

ATOM 3377 HD11 ILE 219 34.902 42.394 62.521 1.00 0.00 H

ATOM 3378 HD12 ILE 219 33.467 41.720 61.747 1.00 0.00 H

ATOM 3379 HD13 ILE 219 35.092 41.399 61.084 1.00 0.00 H

ATOM 3380 C ILE 219 33.387 41.903 57.319 1.00 0.00 C

ATOM 3381 O ILE 219 32.931 42.906 56.781 1.00 0.00 O

ATOM 3382 N ILE 220 33.207 40.696 56.798 1.00 0.00 N

ATOM 3383 H ILE 220 33.578 39.868 57.246 1.00 0.00 H

ATOM 3384 CA ILE 220 32.445 40.569 55.554 1.00 0.00 C

ATOM 3385 HA ILE 220 32.160 41.533 55.142 1.00 0.00 H

ATOM 3386 CB ILE 220 33.230 39.802 54.475 1.00 0.00 C

ATOM 3387 HB ILE 220 33.421 38.795 54.835 1.00 0.00 H

ATOM 3388 CG2 ILE 220 32.424 39.720 53.188 1.00 0.00 C

```
ATOM   3389 HG21 ILE   220      33.023  39.269  52.397  1.00  0.00           H
ATOM   3390 HG22 ILE   220      31.550  39.099  53.354  1.00  0.00           H
ATOM   3391 HG23 ILE   220      32.102  40.706  52.872  1.00  0.00           H
ATOM   3392  CG1 ILE   220      34.584  40.471  54.221  1.00  0.00           C
ATOM   3393 HG12 ILE   220      35.184  40.460  55.131  1.00  0.00           H
ATOM   3394 HG13 ILE   220      35.127  39.887  53.477  1.00  0.00           H
ATOM   3395  CD1 ILE   220      34.477  41.892  53.719  1.00  0.00           C
ATOM   3396 HD11 ILE   220      35.482  42.295  53.600  1.00  0.00           H
ATOM   3397 HD12 ILE   220      33.984  41.931  52.748  1.00  0.00           H
ATOM   3398 HD13 ILE   220      33.952  42.532  54.423  1.00  0.00           H
ATOM   3399  C   ILE   220      31.139  39.835  55.902  1.00  0.00           C
ATOM   3400  O   ILE   220      31.149  38.916  56.716  1.00  0.00           O
ATOM   3401  N   SER   221      29.959  40.215  55.442  1.00  0.00           N
ATOM   3402  H   SER   221      29.938  41.011  54.824  1.00  0.00           H
ATOM   3403  CA  SER   221      28.678  39.575  55.719  1.00  0.00           C
ATOM   3404  HA  SER   221      28.442  39.734  56.770  1.00  0.00           H
ATOM   3405  CB  SER   221      27.592  40.217  54.878  1.00  0.00           C
ATOM   3406  HB2 SER   221      27.469  41.259  55.173  1.00  0.00           H
ATOM   3407  HB3 SER   221      27.878  40.174  53.827  1.00  0.00           H
ATOM   3408  OG  SER   221      26.371  39.550  55.041  1.00  0.00           O
ATOM   3409  HG  SER   221      25.980  39.831  55.874  1.00  0.00           H
ATOM   3410  C   SER   221      28.752  38.043  55.437  1.00  0.00           C
ATOM   3411  O   SER   221      29.318  37.690  54.424  1.00  0.00           O
ATOM   3412  N   LYS   222      28.233  37.167  56.268  1.00  0.00           N
ATOM   3413  H   LYS   222      27.780  37.536  57.093  1.00  0.00           H
ATOM   3414  CA  LYS   222      28.276  35.664  56.158  1.00  0.00           C
ATOM   3415  HA  LYS   222      29.315  35.353  56.280  1.00  0.00           H
ATOM   3416  CB  LYS   222      27.446  35.004  57.261  1.00  0.00           C
ATOM   3417  HB2 LYS   222      27.825  35.351  58.223  1.00  0.00           H
ATOM   3418  HB3 LYS   222      26.410  35.339  57.179  1.00  0.00           H
```

| | | | | | | | | | | |
|---|---|---|---|---|---|---|---|---|---|---|
| ATOM | 3419 | CG | LYS | 222 | 27.470 | 33.481 | 57.239 | 1.00 | 0.00 | C |
| ATOM | 3420 | HG2 | LYS | 222 | 26.985 | 33.113 | 56.334 | 1.00 | 0.00 | H |
| ATOM | 3421 | HG3 | LYS | 222 | 28.507 | 33.142 | 57.244 | 1.00 | 0.00 | H |
| ATOM | 3422 | CD | LYS | 222 | 26.751 | 32.900 | 58.448 | 1.00 | 0.00 | C |
| ATOM | 3423 | HD2 | LYS | 222 | 27.182 | 33.329 | 59.354 | 1.00 | 0.00 | H |
| ATOM | 3424 | HD3 | LYS | 222 | 25.691 | 33.155 | 58.413 | 1.00 | 0.00 | H |
| ATOM | 3425 | CE | LYS | 222 | 26.922 | 31.390 | 58.521 | 1.00 | 0.00 | C |
| ATOM | 3426 | HE2 | LYS | 222 | 27.980 | 31.151 | 58.400 | 1.00 | 0.00 | H |
| ATOM | 3427 | HE3 | LYS | 222 | 26.597 | 31.039 | 59.501 | 1.00 | 0.00 | H |
| ATOM | 3428 | NZ | LYS | 222 | 26.137 | 30.689 | 57.470 | 1.00 | 0.00 | N |
| ATOM | 3429 | HZ1 | LYS | 222 | 26.348 | 31.095 | 56.569 | 1.00 | 0.00 | H |
| ATOM | 3430 | HZ2 | LYS | 222 | 26.405 | 29.715 | 57.426 | 1.00 | 0.00 | H |
| ATOM | 3431 | HZ3 | LYS | 222 | 25.147 | 30.767 | 57.645 | 1.00 | 0.00 | H |
| ATOM | 3432 | C | LYS | 222 | 27.797 | 35.149 | 54.860 | 1.00 | 0.00 | C |
| ATOM | 3433 | O | LYS | 222 | 28.483 | 34.269 | 54.290 | 1.00 | 0.00 | O |
| ATOM | 3434 | N | THR | 223 | 26.638 | 35.581 | 54.342 | 1.00 | 0.00 | N |
| ATOM | 3435 | H | THR | 223 | 26.100 | 36.266 | 54.852 | 1.00 | 0.00 | H |
| ATOM | 3436 | CA | THR | 223 | 26.108 | 35.131 | 52.992 | 1.00 | 0.00 | C |
| ATOM | 3437 | HA | THR | 223 | 26.001 | 34.045 | 53.010 | 1.00 | 0.00 | H |
| ATOM | 3438 | CB | THR | 223 | 24.738 | 35.757 | 52.674 | 1.00 | 0.00 | C |
| ATOM | 3439 | HB | THR | 223 | 24.817 | 36.844 | 52.707 | 1.00 | 0.00 | H |
| ATOM | 3440 | CG2 | THR | 223 | 24.267 | 35.336 | 51.290 | 1.00 | 0.00 | C |
| ATOM | 3441 | HG21 | THR | 223 | 23.231 | 35.647 | 51.149 | 1.00 | 0.00 | H |
| ATOM | 3442 | HG22 | THR | 223 | 24.871 | 35.817 | 50.521 | 1.00 | 0.00 | H |
| ATOM | 3443 | HG23 | THR | 223 | 24.332 | 34.252 | 51.183 | 1.00 | 0.00 | H |
| ATOM | 3444 | OG1 | THR | 223 | 23.778 | 35.334 | 53.650 | 1.00 | 0.00 | O |
| ATOM | 3445 | HG1 | THR | 223 | 23.714 | 34.376 | 53.618 | 1.00 | 0.00 | H |
| ATOM | 3446 | C | THR | 223 | 27.105 | 35.498 | 51.876 | 1.00 | 0.00 | C |
| ATOM | 3447 | O | THR | 223 | 27.428 | 34.677 | 51.094 | 1.00 | 0.00 | O |
| ATOM | 3448 | N | ALA | 224 | 27.736 | 36.690 | 51.942 | 1.00 | 0.00 | N |

| | | | | | | | | | | |
|---|---|---|---|---|---|---|---|---|---|---|
| ATOM | 3449 | H | ALA | 224 | 27.521 | 37.325 | 52.697 | 1.00 | 0.00 | H |
| ATOM | 3450 | CA | ALA | 224 | 28.684 | 37.136 | 50.919 | 1.00 | 0.00 | C |
| ATOM | 3451 | HA | ALA | 224 | 28.185 | 37.232 | 49.954 | 1.00 | 0.00 | H |
| ATOM | 3452 | CB | ALA | 224 | 29.198 | 38.475 | 51.383 | 1.00 | 0.00 | C |
| ATOM | 3453 | HB1 | ALA | 224 | 29.762 | 38.936 | 50.571 | 1.00 | 0.00 | H |
| ATOM | 3454 | HB2 | ALA | 224 | 28.369 | 39.135 | 51.640 | 1.00 | 0.00 | H |
| ATOM | 3455 | HB3 | ALA | 224 | 29.863 | 38.374 | 52.228 | 1.00 | 0.00 | H |
| ATOM | 3456 | C | ALA | 224 | 29.861 | 36.193 | 50.819 | 1.00 | 0.00 | C |
| ATOM | 3457 | O | ALA | 224 | 30.129 | 35.571 | 49.749 | 1.00 | 0.00 | O |
| ATOM | 3458 | N | LEU | 225 | 30.478 | 35.794 | 51.931 | 1.00 | 0.00 | N |
| ATOM | 3459 | H | LEU | 225 | 30.170 | 36.204 | 52.802 | 1.00 | 0.00 | H |
| ATOM | 3460 | CA | LEU | 225 | 31.601 | 34.848 | 52.029 | 1.00 | 0.00 | C |
| ATOM | 3461 | HA | LEU | 225 | 32.399 | 35.185 | 51.373 | 1.00 | 0.00 | H |
| ATOM | 3462 | CB | LEU | 225 | 32.110 | 34.815 | 53.476 | 1.00 | 0.00 | C |
| ATOM | 3463 | HB2 | LEU | 225 | 31.269 | 34.606 | 54.139 | 1.00 | 0.00 | H |
| ATOM | 3464 | HB3 | LEU | 225 | 32.807 | 33.979 | 53.566 | 1.00 | 0.00 | H |
| ATOM | 3465 | CG | LEU | 225 | 32.824 | 36.083 | 53.959 | 1.00 | 0.00 | C |
| ATOM | 3466 | HG | LEU | 225 | 32.118 | 36.912 | 53.951 | 1.00 | 0.00 | H |
| ATOM | 3467 | CD1 | LEU | 225 | 33.309 | 35.881 | 55.388 | 1.00 | 0.00 | C |
| ATOM | 3468 | HD11 | LEU | 225 | 33.729 | 36.801 | 55.791 | 1.00 | 0.00 | H |
| ATOM | 3469 | HD12 | LEU | 225 | 32.472 | 35.569 | 56.008 | 1.00 | 0.00 | H |
| ATOM | 3470 | HD13 | LEU | 225 | 34.072 | 35.101 | 55.417 | 1.00 | 0.00 | H |
| ATOM | 3471 | CD2 | LEU | 225 | 33.985 | 36.398 | 53.027 | 1.00 | 0.00 | C |
| ATOM | 3472 | HD21 | LEU | 225 | 34.623 | 37.154 | 53.482 | 1.00 | 0.00 | H |
| ATOM | 3473 | HD22 | LEU | 225 | 34.577 | 35.502 | 52.842 | 1.00 | 0.00 | H |
| ATOM | 3474 | HD23 | LEU | 225 | 33.620 | 36.794 | 52.082 | 1.00 | 0.00 | H |
| ATOM | 3475 | C | LEU | 225 | 31.228 | 33.400 | 51.571 | 1.00 | 0.00 | C |
| ATOM | 3476 | O | LEU | 225 | 31.974 | 32.921 | 50.721 | 1.00 | 0.00 | O |
| ATOM | 3477 | N | GLU | 226 | 30.060 | 32.902 | 51.903 | 1.00 | 0.00 | N |
| ATOM | 3478 | H | GLU | 226 | 29.454 | 33.384 | 52.552 | 1.00 | 0.00 | H |

ATOM 3479 CA GLU 226 29.657 31.589 51.383 1.00 0.00 C
ATOM 3480 HA GLU 226 30.415 30.856 51.667 1.00 0.00 H
ATOM 3481 CB GLU 226 28.332 31.171 52.025 1.00 0.00 C
ATOM 3482 HB2 GLU 226 27.613 31.980 51.892 1.00 0.00 H
ATOM 3483 HB3 GLU 226 27.955 30.291 51.501 1.00 0.00 H
ATOM 3484 CG GLU 226 28.432 30.823 53.504 1.00 0.00 C
ATOM 3485 HG2 GLU 226 29.096 29.964 53.618 1.00 0.00 H
ATOM 3486 HG3 GLU 226 28.868 31.661 54.048 1.00 0.00 H
ATOM 3487 CD GLU 226 27.099 30.497 54.119 1.00 0.00 C
ATOM 3488 OE1 GLU 226 26.107 30.624 53.443 1.00 0.00 O
ATOM 3489 OE2 GLU 226 27.074 30.120 55.267 1.00 0.00 O
ATOM 3490 C GLU 226 29.516 31.536 49.852 1.00 0.00 C
ATOM 3491 O GLU 226 30.039 30.647 49.203 1.00 0.00 O
ATOM 3492 N ARG 227 28.961 32.649 49.307 1.00 0.00 N
ATOM 3493 H ARG 227 28.620 33.399 49.892 1.00 0.00 H
ATOM 3494 CA ARG 227 28.727 32.677 47.882 1.00 0.00 C
ATOM 3495 HA ARG 227 28.337 31.710 47.558 1.00 0.00 H
ATOM 3496 CB ARG 227 27.706 33.749 47.531 1.00 0.00 C
ATOM 3497 HB2 ARG 227 28.045 34.708 47.922 1.00 0.00 H
ATOM 3498 HB3 ARG 227 27.648 33.806 46.446 1.00 0.00 H
ATOM 3499 CG ARG 227 26.295 33.475 48.024 1.00 0.00 C
ATOM 3500 HG2 ARG 227 25.949 32.534 47.593 1.00 0.00 H
ATOM 3501 HG3 ARG 227 26.297 33.376 49.109 1.00 0.00 H
ATOM 3502 CD ARG 227 25.357 34.560 47.638 1.00 0.00 C
ATOM 3503 HD2 ARG 227 25.703 35.496 48.080 1.00 0.00 H
ATOM 3504 HD3 ARG 227 25.358 34.659 46.552 1.00 0.00 H
ATOM 3505 NE ARG 227 23.999 34.288 48.081 1.00 0.00 N
ATOM 3506 HE ARG 227 23.826 33.381 48.491 1.00 0.00 H
ATOM 3507 CZ ARG 227 22.972 35.153 47.976 1.00 0.00 C
ATOM 3508 NH1 ARG 227 23.163 36.339 47.442 1.00 0.00 N

| | | | | | | | | | | |
|---|---|---|---|---|---|---|---|---|---|---|
| ATOM | 3509 | HH11 | ARG | 227 | 22.387 | 36.970 | 47.319 | 1.00 | 0.00 | H |
| ATOM | 3510 | HH12 | ARG | 227 | 24.082 | 36.591 | 47.113 | 1.00 | 0.00 | H |
| ATOM | 3511 | NH2 | ARG | 227 | 21.772 | 34.809 | 48.410 | 1.00 | 0.00 | N |
| ATOM | 3512 | HH21 | ARG | 227 | 21.004 | 35.460 | 48.357 | 1.00 | 0.00 | H |
| ATOM | 3513 | HH22 | ARG | 227 | 21.622 | 33.885 | 48.788 | 1.00 | 0.00 | H |
| ATOM | 3514 | C | ARG | 227 | 30.021 | 32.935 | 47.123 | 1.00 | 0.00 | C |
| ATOM | 3515 | O | ARG | 227 | 30.392 | 32.154 | 46.168 | 1.00 | 0.00 | O |
| ATOM | 3516 | N | LEU | 228 | 30.867 | 33.750 | 47.690 | 1.00 | 0.00 | N |
| ATOM | 3517 | H | LEU | 228 | 30.570 | 34.215 | 48.535 | 1.00 | 0.00 | H |
| ATOM | 3518 | CA | LEU | 228 | 32.249 | 34.093 | 47.186 | 1.00 | 0.00 | C |
| ATOM | 3519 | HA | LEU | 228 | 32.166 | 34.481 | 46.172 | 1.00 | 0.00 | H |
| ATOM | 3520 | CB | LEU | 228 | 32.904 | 35.142 | 48.094 | 1.00 | 0.00 | C |
| ATOM | 3521 | HB2 | LEU | 228 | 32.755 | 34.832 | 49.127 | 1.00 | 0.00 | H |
| ATOM | 3522 | HB3 | LEU | 228 | 33.981 | 35.132 | 47.918 | 1.00 | 0.00 | H |
| ATOM | 3523 | CG | LEU | 228 | 32.402 | 36.581 | 47.919 | 1.00 | 0.00 | C |
| ATOM | 3524 | HG | LEU | 228 | 31.317 | 36.596 | 47.892 | 1.00 | 0.00 | H |
| ATOM | 3525 | CD1 | LEU | 228 | 32.874 | 37.430 | 49.090 | 1.00 | 0.00 | C |
| ATOM | 3526 | HD11 | LEU | 228 | 32.493 | 38.444 | 48.982 | 1.00 | 0.00 | H |
| ATOM | 3527 | HD12 | LEU | 228 | 32.493 | 37.026 | 50.025 | 1.00 | 0.00 | H |
| ATOM | 3528 | HD13 | LEU | 228 | 33.964 | 37.452 | 49.125 | 1.00 | 0.00 | H |
| ATOM | 3529 | CD2 | LEU | 228 | 32.911 | 37.139 | 46.598 | 1.00 | 0.00 | C |
| ATOM | 3530 | HD21 | LEU | 228 | 32.649 | 38.188 | 46.525 | 1.00 | 0.00 | H |
| ATOM | 3531 | HD22 | LEU | 228 | 33.998 | 37.060 | 46.550 | 1.00 | 0.00 | H |
| ATOM | 3532 | HD23 | LEU | 228 | 32.489 | 36.606 | 45.751 | 1.00 | 0.00 | H |
| ATOM | 3533 | C | LEU | 228 | 33.151 | 32.799 | 47.122 | 1.00 | 0.00 | C |
| ATOM | 3534 | O | LEU | 228 | 34.022 | 32.772 | 46.304 | 1.00 | 0.00 | O |
| ATOM | 3535 | N | VAL | 229 | 33.036 | 31.786 | 48.026 | 1.00 | 0.00 | N |
| ATOM | 3536 | H | VAL | 229 | 32.328 | 31.868 | 48.744 | 1.00 | 0.00 | H |
| ATOM | 3537 | CA | VAL | 229 | 33.869 | 30.503 | 48.036 | 1.00 | 0.00 | C |
| ATOM | 3538 | HA | VAL | 229 | 34.882 | 30.727 | 47.699 | 1.00 | 0.00 | H |

| | | | | | | | | | | |
|---|---|---|---|---|---|---|---|---|---|---|
| ATOM | 3539 | CB | VAL | 229 | 33.933 | 29.876 | 49.442 | 1.00 | 0.00 | C |
| ATOM | 3540 | HB | VAL | 229 | 32.918 | 29.697 | 49.801 | 1.00 | 0.00 | H |
| ATOM | 3541 | CG1 | VAL | 229 | 34.671 | 28.547 | 49.399 | 1.00 | 0.00 | C |
| ATOM | 3542 | HG11 | VAL | 229 | 34.786 | 28.160 | 50.412 | 1.00 | 0.00 | H |
| ATOM | 3543 | HG12 | VAL | 229 | 34.111 | 27.812 | 48.824 | 1.00 | 0.00 | H |
| ATOM | 3544 | HG13 | VAL | 229 | 35.659 | 28.676 | 48.956 | 1.00 | 0.00 | H |
| ATOM | 3545 | CG2 | VAL | 229 | 34.609 | 30.838 | 50.407 | 1.00 | 0.00 | C |
| ATOM | 3546 | HG21 | VAL | 229 | 34.591 | 30.419 | 51.413 | 1.00 | 0.00 | H |
| ATOM | 3547 | HG22 | VAL | 229 | 35.638 | 31.011 | 50.096 | 1.00 | 0.00 | H |
| ATOM | 3548 | HG23 | VAL | 229 | 34.104 | 31.797 | 50.430 | 1.00 | 0.00 | H |
| ATOM | 3549 | C | VAL | 229 | 33.239 | 29.494 | 47.064 | 1.00 | 0.00 | C |
| ATOM | 3550 | O | VAL | 229 | 33.849 | 28.945 | 46.160 | 1.00 | 0.00 | O |
| ATOM | 3551 | N | GLU | 230 | 31.963 | 29.263 | 47.185 | 1.00 | 0.00 | N |
| ATOM | 3552 | H | GLU | 230 | 31.450 | 29.773 | 47.892 | 1.00 | 0.00 | H |
| ATOM | 3553 | CA | GLU | 230 | 31.229 | 28.226 | 46.486 | 1.00 | 0.00 | C |
| ATOM | 3554 | HA | GLU | 230 | 31.920 | 27.418 | 46.242 | 1.00 | 0.00 | H |
| ATOM | 3555 | CB | GLU | 230 | 30.209 | 27.653 | 47.472 | 1.00 | 0.00 | C |
| ATOM | 3556 | HB2 | GLU | 230 | 29.560 | 28.455 | 47.826 | 1.00 | 0.00 | H |
| ATOM | 3557 | HB3 | GLU | 230 | 29.589 | 26.932 | 46.938 | 1.00 | 0.00 | H |
| ATOM | 3558 | CG | GLU | 230 | 30.822 | 26.923 | 48.658 | 1.00 | 0.00 | C |
| ATOM | 3559 | HG2 | GLU | 230 | 31.466 | 26.127 | 48.282 | 1.00 | 0.00 | H |
| ATOM | 3560 | HG3 | GLU | 230 | 31.433 | 27.615 | 49.239 | 1.00 | 0.00 | H |
| ATOM | 3561 | CD | GLU | 230 | 29.791 | 26.318 | 49.570 | 1.00 | 0.00 | C |
| ATOM | 3562 | OE1 | GLU | 230 | 28.624 | 26.500 | 49.319 | 1.00 | 0.00 | O |
| ATOM | 3563 | OE2 | GLU | 230 | 30.171 | 25.673 | 50.519 | 1.00 | 0.00 | O |
| ATOM | 3564 | C | GLU | 230 | 30.493 | 28.594 | 45.140 | 1.00 | 0.00 | C |
| ATOM | 3565 | O | GLU | 230 | 29.840 | 27.765 | 44.467 | 1.00 | 0.00 | O |
| ATOM | 3566 | N | ALA | 231 | 30.608 | 29.831 | 44.705 | 1.00 | 0.00 | N |
| ATOM | 3567 | H | ALA | 231 | 31.108 | 30.491 | 45.284 | 1.00 | 0.00 | H |
| ATOM | 3568 | CA | ALA | 231 | 30.141 | 30.327 | 43.419 | 1.00 | 0.00 | C |

ATOM 3569 HA ALA 231 29.071 30.113 43.406 1.00 0.00 H
ATOM 3570 CB ALA 231 30.238 31.849 43.395 1.00 0.00 C
ATOM 3571 HB1 ALA 231 30.078 32.220 42.382 1.00 0.00 H
ATOM 3572 HB2 ALA 231 29.449 32.276 44.009 1.00 0.00 H
ATOM 3573 HB3 ALA 231 31.225 32.172 43.727 1.00 0.00 H
ATOM 3574 C ALA 231 30.649 29.727 42.125 1.00 0.00 C
ATOM 3575 O ALA 231 29.954 29.730 41.104 1.00 0.00 O
ATOM 3576 N PHE 232 31.883 29.198 42.106 1.00 0.00 N
ATOM 3577 H PHE 232 32.425 29.206 42.957 1.00 0.00 H
ATOM 3578 CA PHE 232 32.510 28.626 40.908 1.00 0.00 C
ATOM 3579 HA PHE 232 31.856 28.802 40.052 1.00 0.00 H
ATOM 3580 CB PHE 232 33.797 29.403 40.626 1.00 0.00 C
ATOM 3581 HB2 PHE 232 34.486 29.267 41.461 1.00 0.00 H
ATOM 3582 HB3 PHE 232 34.274 28.988 39.737 1.00 0.00 H
ATOM 3583 CG PHE 232 33.582 30.874 40.410 1.00 0.00 C
ATOM 3584 CD1 PHE 232 33.565 31.752 41.484 1.00 0.00 C
ATOM 3585 HD1 PHE 232 33.722 31.383 42.487 1.00 0.00 H
ATOM 3586 CE1 PHE 232 33.367 33.106 41.286 1.00 0.00 C
ATOM 3587 HE1 PHE 232 33.340 33.779 42.130 1.00 0.00 H
ATOM 3588 CZ PHE 232 33.184 33.597 40.011 1.00 0.00 C
ATOM 3589 HZ PHE 232 33.037 34.654 39.846 1.00 0.00 H
ATOM 3590 CE2 PHE 232 33.199 32.735 38.933 1.00 0.00 C
ATOM 3591 HE2 PHE 232 33.065 33.118 37.935 1.00 0.00 H
ATOM 3592 CD2 PHE 232 33.396 31.383 39.134 1.00 0.00 C
ATOM 3593 HD2 PHE 232 33.384 30.716 38.284 1.00 0.00 H
ATOM 3594 C PHE 232 32.849 27.117 40.910 1.00 0.00 C
ATOM 3595 O PHE 232 33.269 26.508 41.923 1.00 0.00 O
ATOM 3596 N GLN 233 32.693 26.515 39.735 1.00 0.00 N
ATOM 3597 H GLN 233 32.337 27.080 38.975 1.00 0.00 H
ATOM 3598 CA GLN 233 33.073 25.136 39.361 1.00 0.00 C

ATOM 3599 HA GLN 233 33.286 24.564 40.266 1.00 0.00 H

ATOM 3600 CB GLN 233 31.962 24.423 38.585 1.00 0.00 C

ATOM 3601 HB2 GLN 233 31.677 25.028 37.729 1.00 0.00 H

ATOM 3602 HB3 GLN 233 32.347 23.467 38.229 1.00 0.00 H

ATOM 3603 CG GLN 233 30.704 24.162 39.396 1.00 0.00 C

ATOM 3604 HG2 GLN 233 30.988 23.776 40.376 1.00 0.00 H

ATOM 3605 HG3 GLN 233 30.169 25.101 39.546 1.00 0.00 H

ATOM 3606 CD GLN 233 29.787 23.153 38.732 1.00 0.00 C

ATOM 3607 OE1 GLN 233 28.742 23.646 38.077 1.00 0.00 O

ATOM 3608 NE2 GLN 233 30.014 21.943 38.808 1.00 0.00 N

ATOM 3609 HE21 GLN 233 30.810 21.612 39.330 1.00 0.00 H

ATOM 3610 HE22 GLN 233 29.402 21.297 38.335 1.00 0.00 H

ATOM 3611 C GLN 233 34.312 25.206 38.532 1.00 0.00 C

ATOM 3612 O GLN 233 34.609 26.229 37.840 1.00 0.00 O

ATOM 3613 N ARG 234 34.986 24.090 38.521 1.00 0.00 N

ATOM 3614 H ARG 234 34.565 23.335 39.043 1.00 0.00 H

ATOM 3615 CA ARG 234 36.321 23.755 37.968 1.00 0.00 C

ATOM 3616 HA ARG 234 36.613 24.566 37.299 1.00 0.00 H

ATOM 3617 CB ARG 234 37.333 23.710 39.104 1.00 0.00 C

ATOM 3618 HB2 ARG 234 38.317 23.538 38.689 1.00 0.00 H

ATOM 3619 HB3 ARG 234 37.340 24.686 39.590 1.00 0.00 H

ATOM 3620 CG ARG 234 37.069 22.641 40.153 1.00 0.00 C

ATOM 3621 HG2 ARG 234 36.059 22.749 40.549 1.00 0.00 H

ATOM 3622 HG3 ARG 234 37.163 21.661 39.689 1.00 0.00 H

ATOM 3623 CD ARG 234 38.030 22.732 41.283 1.00 0.00 C

ATOM 3624 HD2 ARG 234 39.039 22.737 40.874 1.00 0.00 H

ATOM 3625 HD3 ARG 234 37.858 23.656 41.837 1.00 0.00 H

ATOM 3626 NE ARG 234 37.924 21.591 42.178 1.00 0.00 N

ATOM 3627 HE ARG 234 37.119 20.989 42.065 1.00 0.00 H

ATOM 3628 CZ ARG 234 38.803 21.303 43.158 1.00 0.00 C

| | | | | | | | | | | |
|---|---|---|---|---|---|---|---|---|---|---|
| ATOM | 3629 | NH1 | ARG | 234 | 39.844 | 22.080 | 43.355 | 1.00 | 0.00 | N |
| ATOM | 3630 | HH11 | ARG | 234 | 39.955 | 22.916 | 42.802 | 1.00 | 0.00 | H |
| ATOM | 3631 | HH12 | ARG | 234 | 40.476 | 21.881 | 44.114 | 1.00 | 0.00 | H |
| ATOM | 3632 | NH2 | ARG | 234 | 38.618 | 20.239 | 43.920 | 1.00 | 0.00 | N |
| ATOM | 3633 | HH21 | ARG | 234 | 39.277 | 20.014 | 44.652 | 1.00 | 0.00 | H |
| ATOM | 3634 | HH22 | ARG | 234 | 37.811 | 19.648 | 43.771 | 1.00 | 0.00 | H |
| ATOM | 3635 | C | ARG | 234 | 36.445 | 22.439 | 37.180 | 1.00 | 0.00 | C |
| ATOM | 3636 | O | ARG | 234 | 35.659 | 21.504 | 37.326 | 1.00 | 0.00 | O |
| ATOM | 3637 | N | ALA | 235 | 37.505 | 22.344 | 36.399 | 1.00 | 0.00 | N |
| ATOM | 3638 | H | ALA | 235 | 38.101 | 23.158 | 36.373 | 1.00 | 0.00 | H |
| ATOM | 3639 | CA | ALA | 235 | 37.934 | 21.286 | 35.552 | 1.00 | 0.00 | C |
| ATOM | 3640 | HA | ALA | 235 | 37.038 | 20.893 | 35.069 | 1.00 | 0.00 | H |
| ATOM | 3641 | CB | ALA | 235 | 38.798 | 21.812 | 34.455 | 1.00 | 0.00 | C |
| ATOM | 3642 | HB1 | ALA | 235 | 39.117 | 20.994 | 33.809 | 1.00 | 0.00 | H |
| ATOM | 3643 | HB2 | ALA | 235 | 38.234 | 22.513 | 33.845 | 1.00 | 0.00 | H |
| ATOM | 3644 | HB3 | ALA | 235 | 39.678 | 22.305 | 34.869 | 1.00 | 0.00 | H |
| ATOM | 3645 | C | ALA | 235 | 38.529 | 20.092 | 36.374 | 1.00 | 0.00 | C |
| ATOM | 3646 | O | ALA | 235 | 39.532 | 19.557 | 35.947 | 1.00 | 0.00 | O |
| ATOM | 3647 | N | ARG | 236 | 37.846 | 19.634 | 37.409 | 1.00 | 0.00 | N |
| ATOM | 3648 | H | ARG | 236 | 36.986 | 20.115 | 37.637 | 1.00 | 0.00 | H |
| ATOM | 3649 | CA | ARG | 236 | 38.171 | 18.488 | 38.284 | 1.00 | 0.00 | C |
| ATOM | 3650 | HA | ARG | 236 | 38.186 | 17.591 | 37.665 | 1.00 | 0.00 | H |
| ATOM | 3651 | CB | ARG | 236 | 39.557 | 18.683 | 38.881 | 1.00 | 0.00 | C |
| ATOM | 3652 | HB2 | ARG | 236 | 39.799 | 17.797 | 39.468 | 1.00 | 0.00 | H |
| ATOM | 3653 | HB3 | ARG | 236 | 40.302 | 18.725 | 38.090 | 1.00 | 0.00 | H |
| ATOM | 3654 | CG | ARG | 236 | 39.696 | 19.895 | 39.790 | 1.00 | 0.00 | C |
| ATOM | 3655 | HG2 | ARG | 236 | 39.479 | 20.810 | 39.237 | 1.00 | 0.00 | H |
| ATOM | 3656 | HG3 | ARG | 236 | 38.987 | 19.795 | 40.608 | 1.00 | 0.00 | H |
| ATOM | 3657 | CD | ARG | 236 | 41.058 | 19.988 | 40.375 | 1.00 | 0.00 | C |
| ATOM | 3658 | HD2 | ARG | 236 | 41.079 | 20.814 | 41.087 | 1.00 | 0.00 | H |

```
ATOM   3659  HD3 ARG   236      41.270  19.062  40.912  1.00  0.00           H
ATOM   3660  NE  ARG   236      42.070  20.226  39.358  1.00  0.00           N
ATOM   3661  HE  ARG   236      41.753  20.589  38.471  1.00  0.00           H
ATOM   3662  CZ  ARG   236      43.384  19.969  39.512  1.00  0.00           C
ATOM   3663  NH1 ARG   236      43.828  19.467  40.643  1.00  0.00           N
ATOM   3664 HH11 ARG   236      44.812  19.282  40.764  1.00  0.00           H
ATOM   3665 HH12 ARG   236      43.184  19.242  41.388  1.00  0.00           H
ATOM   3666  NH2 ARG   236      44.226  20.221  38.525  1.00  0.00           N
ATOM   3667 HH21 ARG   236      43.878  20.572  37.645  1.00  0.00           H
ATOM   3668 HH22 ARG   236      45.216  20.075  38.653  1.00  0.00           H
ATOM   3669  C   ARG   236      37.112  18.280  39.450  1.00  0.00           C
ATOM   3670  O   ARG   236      36.345  19.210  39.659  1.00  0.00           O
ATOM   3671  N   PRO   237      36.939  17.098  40.109  1.00  0.00           N
ATOM   3672  CD  PRO   237      37.739  15.916  39.854  1.00  0.00           C
ATOM   3673  HD2 PRO   237      38.700  15.996  40.363  1.00  0.00           H
ATOM   3674  HD3 PRO   237      37.869  15.725  38.789  1.00  0.00           H
ATOM   3675  CG  PRO   237      36.892  14.807  40.496  1.00  0.00           C
ATOM   3676  HG2 PRO   237      37.504  13.950  40.781  1.00  0.00           H
ATOM   3677  HG3 PRO   237      36.098  14.501  39.812  1.00  0.00           H
ATOM   3678  CB  PRO   237      36.302  15.491  41.695  1.00  0.00           C
ATOM   3679  HB2 PRO   237      37.062  15.562  42.476  1.00  0.00           H
ATOM   3680  HB3 PRO   237      35.424  14.962  42.066  1.00  0.00           H
ATOM   3681  CA  PRO   237      35.946  16.844  41.165  1.00  0.00           C
ATOM   3682  HA  PRO   237      34.971  16.774  40.678  1.00  0.00           H
ATOM   3683  C   PRO   237      35.849  17.808  42.334  1.00  0.00           C
ATOM   3684  O   PRO   237      36.788  18.147  42.980  1.00  0.00           O
ATOM   3685  N   GLY   238      34.626  18.185  42.646  1.00  0.00           N
ATOM   3686  H   GLY   238      33.843  17.863  42.096  1.00  0.00           H
ATOM   3687  CA  GLY   238      34.359  19.136  43.803  1.00  0.00           C
ATOM   3688  HA2 GLY   238      33.335  18.995  44.150  1.00  0.00           H
```

ATOM 3689 HA3 GLY 238 35.040 18.946 44.634 1.00 0.00 H

ATOM 3690 C GLY 238 34.534 20.577 43.303 1.00 0.00 C

ATOM 3691 O GLY 238 35.235 20.865 42.334 1.00 0.00 O

ATOM 3692 N ASN 239 34.079 21.479 44.147 1.00 0.00 N

ATOM 3693 H ASN 239 33.618 21.158 44.986 1.00 0.00 H

ATOM 3694 CA ASN 239 34.177 22.903 43.987 1.00 0.00 C

ATOM 3695 HA ASN 239 34.212 23.133 42.921 1.00 0.00 H

ATOM 3696 CB ASN 239 32.941 23.582 44.546 1.00 0.00 C

ATOM 3697 HB2 ASN 239 32.910 24.641 44.287 1.00 0.00 H

ATOM 3698 HB3 ASN 239 32.065 23.131 44.077 1.00 0.00 H

ATOM 3699 CG ASN 239 32.819 23.423 46.036 1.00 0.00 C

ATOM 3700 OD1 ASN 239 33.795 23.094 46.720 1.00 0.00 O

ATOM 3701 ND2 ASN 239 31.638 23.649 46.553 1.00 0.00 N

ATOM 3702 HD21 ASN 239 30.870 23.941 45.967 1.00 0.00 H

ATOM 3703 HD22 ASN 239 31.512 23.572 47.552 1.00 0.00 H

ATOM 3704 C ASN 239 35.381 23.487 44.602 1.00 0.00 C

ATOM 3705 O ASN 239 36.323 22.842 45.063 1.00 0.00 O

ATOM 3706 N HID 240 35.392 24.851 44.727 1.00 0.00 N

ATOM 3707 H HID 240 34.622 25.413 44.393 1.00 0.00 H

ATOM 3708 CA HID 240 36.597 25.525 45.257 1.00 0.00 C

ATOM 3709 HA HID 240 37.483 24.994 44.907 1.00 0.00 H

ATOM 3710 CB HID 240 36.677 26.950 44.700 1.00 0.00 C

ATOM 3711 HB2 HID 240 35.742 27.472 44.905 1.00 0.00 H

ATOM 3712 HB3 HID 240 37.477 27.500 45.197 1.00 0.00 H

ATOM 3713 CG HID 240 36.962 27.007 43.231 1.00 0.00 C

ATOM 3714 ND1 HID 240 35.976 26.868 42.277 1.00 0.00 N

ATOM 3715 HD1 HID 240 34.990 26.761 42.466 1.00 0.00 H

ATOM 3716 CE1 HID 240 36.516 26.960 41.074 1.00 0.00 C

ATOM 3717 HE1 HID 240 35.982 26.921 40.136 1.00 0.00 H

ATOM 3718 NE2 HID 240 37.815 27.153 41.215 1.00 0.00 N

ATOM 3719 CD2 HID 240 38.120 27.186 42.554 1.00 0.00 C

ATOM 3720 HD2 HID 240 39.098 27.329 42.986 1.00 0.00 H

ATOM 3721 C HID 240 36.688 25.594 46.789 1.00 0.00 C

ATOM 3722 O HID 240 37.692 26.163 47.290 1.00 0.00 O

ATOM 3723 N ASN 241 35.735 25.031 47.462 1.00 0.00 N

ATOM 3724 H ASN 241 34.990 24.567 46.965 1.00 0.00 H

ATOM 3725 CA ASN 241 35.678 25.008 48.969 1.00 0.00 C

ATOM 3726 HA ASN 241 36.047 25.959 49.357 1.00 0.00 H

ATOM 3727 CB ASN 241 34.275 24.778 49.499 1.00 0.00 C

ATOM 3728 HB2 ASN 241 33.614 25.525 49.062 1.00 0.00 H

ATOM 3729 HB3 ASN 241 33.915 23.795 49.200 1.00 0.00 H

ATOM 3730 CG ASN 241 34.200 24.873 50.998 1.00 0.00 C

ATOM 3731 OD1 ASN 241 35.197 24.658 51.696 1.00 0.00 O

ATOM 3732 ND2 ASN 241 33.037 25.192 51.505 1.00 0.00 N

ATOM 3733 HD21 ASN 241 32.234 25.355 50.912 1.00 0.00 H

ATOM 3734 HD22 ASN 241 32.939 25.244 52.507 1.00 0.00 H

ATOM 3735 C ASN 241 36.649 23.872 49.385 1.00 0.00 C

ATOM 3736 O ASN 241 36.369 22.660 49.377 1.00 0.00 O

ATOM 3737 N GLY 242 37.818 24.183 49.872 1.00 0.00 N

ATOM 3738 H GLY 242 38.043 25.161 49.998 1.00 0.00 H

ATOM 3739 CA GLY 242 38.893 23.198 50.116 1.00 0.00 C

ATOM 3740 HA2 GLY 242 39.108 23.167 51.183 1.00 0.00 H

ATOM 3741 HA3 GLY 242 38.611 22.196 49.794 1.00 0.00 H

ATOM 3742 C GLY 242 40.195 23.599 49.345 1.00 0.00 C

ATOM 3743 O GLY 242 41.219 22.927 49.467 1.00 0.00 O

ATOM 3744 N ASN 243 40.219 24.753 48.595 1.00 0.00 N

ATOM 3745 H ASN 243 39.387 25.327 48.559 1.00 0.00 H

ATOM 3746 CA ASN 243 41.338 25.207 47.861 1.00 0.00 C

ATOM 3747 HA ASN 243 41.758 24.358 47.321 1.00 0.00 H

ATOM 3748 CB ASN 243 40.867 26.217 46.831 1.00 0.00 C

```
ATOM   3749  HB2 ASN   243      41.720  26.505  46.216  1.00  0.00           H
ATOM   3750  HB3 ASN   243      40.123  25.762  46.176  1.00  0.00           H
ATOM   3751  CG  ASN   243      40.288  27.454  47.458  1.00  0.00           C
ATOM   3752  OD1 ASN   243      40.020  27.487  48.664  1.00  0.00           O
ATOM   3753  ND2 ASN   243      40.089  28.474  46.662  1.00  0.00           N
ATOM   3754 HD21 ASN   243      40.338  28.408  45.688  1.00  0.00           H
ATOM   3755 HD22 ASN   243      39.737  29.339  47.044  1.00  0.00           H
ATOM   3756  C   ASN   243      42.466  25.797  48.754  1.00  0.00           C
ATOM   3757  O   ASN   243      42.843  26.970  48.605  1.00  0.00           O
ATOM   3758  N   HIE   244      43.014  24.930  49.647  1.00  0.00           N
ATOM   3759  H   HIE   244      42.702  23.972  49.692  1.00  0.00           H
ATOM   3760  CA  HIE   244      44.003  25.353  50.596  1.00  0.00           C
ATOM   3761  HA  HIE   244      44.481  26.258  50.221  1.00  0.00           H
ATOM   3762  CB  HIE   244      43.294  25.721  51.903  1.00  0.00           C
ATOM   3763  HB2 HIE   244      43.868  26.484  52.425  1.00  0.00           H
ATOM   3764  HB3 HIE   244      42.316  26.142  51.673  1.00  0.00           H
ATOM   3765  CG  HIE   244      43.106  24.562  52.832  1.00  0.00           C
ATOM   3766  ND1 HIE   244      43.896  24.365  53.945  1.00  0.00           N
ATOM   3767  CE1 HIE   244      43.502  23.270  54.572  1.00  0.00           C
ATOM   3768  HE1 HIE   244      43.936  22.873  55.478  1.00  0.00           H
ATOM   3769  NE2 HIE   244      42.487  22.751  53.906  1.00  0.00           N
ATOM   3770  HE2 HIE   244      41.982  21.916  54.164  1.00  0.00           H
ATOM   3771  CD2 HIE   244      42.219  23.539  52.814  1.00  0.00           C
ATOM   3772  HD2 HIE   244      41.457  23.367  52.071  1.00  0.00           H
ATOM   3773  C   HIE   244      45.136  24.347  50.899  1.00  0.00           C
ATOM   3774  O   HIE   244      44.930  23.103  50.733  1.00  0.00           O
ATOM   3775  N   ARG   245      46.384  24.742  51.253  1.00  0.00           N
ATOM   3776  H   ARG   245      46.578  25.735  51.272  1.00  0.00           H
ATOM   3777  CA  ARG   245      47.352  23.907  51.868  1.00  0.00           C
ATOM   3778  HA  ARG   245      47.098  22.867  51.675  1.00  0.00           H
```

| | | | | | | | | | | |
|---|---|---|---|---|---|---|---|---|---|---|
| ATOM | 3779 | CB | ARG | 245 | 48.697 | 24.162 | 51.206 | 1.00 | 0.00 | C |
| ATOM | 3780 | HB2 | ARG | 245 | 48.588 | 23.996 | 50.132 | 1.00 | 0.00 | H |
| ATOM | 3781 | HB3 | ARG | 245 | 48.971 | 25.209 | 51.355 | 1.00 | 0.00 | H |
| ATOM | 3782 | CG | ARG | 245 | 49.835 | 23.288 | 51.710 | 1.00 | 0.00 | C |
| ATOM | 3783 | HG2 | ARG | 245 | 49.991 | 23.465 | 52.772 | 1.00 | 0.00 | H |
| ATOM | 3784 | HG3 | ARG | 245 | 49.569 | 22.241 | 51.566 | 1.00 | 0.00 | H |
| ATOM | 3785 | CD | ARG | 245 | 51.104 | 23.572 | 50.992 | 1.00 | 0.00 | C |
| ATOM | 3786 | HD2 | ARG | 245 | 50.956 | 23.402 | 49.930 | 1.00 | 0.00 | H |
| ATOM | 3787 | HD3 | ARG | 245 | 51.379 | 24.615 | 51.157 | 1.00 | 0.00 | H |
| ATOM | 3788 | NE | ARG | 245 | 52.185 | 22.709 | 51.442 | 1.00 | 0.00 | N |
| ATOM | 3789 | HE | ARG | 245 | 51.957 | 22.016 | 52.140 | 1.00 | 0.00 | H |
| ATOM | 3790 | CZ | ARG | 245 | 53.461 | 22.796 | 51.017 | 1.00 | 0.00 | C |
| ATOM | 3791 | NH1 | ARG | 245 | 53.799 | 23.711 | 50.136 | 1.00 | 0.00 | N |
| ATOM | 3792 | HH11 | ARG | 245 | 54.750 | 23.791 | 49.813 | 1.00 | 0.00 | H |
| ATOM | 3793 | HH12 | ARG | 245 | 53.098 | 24.360 | 49.808 | 1.00 | 0.00 | H |
| ATOM | 3794 | NH2 | ARG | 245 | 54.373 | 21.963 | 51.488 | 1.00 | 0.00 | N |
| ATOM | 3795 | HH21 | ARG | 245 | 54.098 | 21.218 | 52.110 | 1.00 | 0.00 | H |
| ATOM | 3796 | HH22 | ARG | 245 | 55.338 | 22.075 | 51.217 | 1.00 | 0.00 | H |
| ATOM | 3797 | C | ARG | 245 | 47.465 | 24.111 | 53.432 | 1.00 | 0.00 | C |
| ATOM | 3798 | O | ARG | 245 | 47.581 | 25.256 | 53.813 | 1.00 | 0.00 | O |
| ATOM | 3799 | N | PRO | 246 | 47.460 | 23.086 | 54.295 | 1.00 | 0.00 | N |
| ATOM | 3800 | CD | PRO | 246 | 47.246 | 21.662 | 53.952 | 1.00 | 0.00 | C |
| ATOM | 3801 | HD2 | PRO | 246 | 47.891 | 21.341 | 53.134 | 1.00 | 0.00 | H |
| ATOM | 3802 | HD3 | PRO | 246 | 46.196 | 21.498 | 53.708 | 1.00 | 0.00 | H |
| ATOM | 3803 | CG | PRO | 246 | 47.609 | 20.939 | 55.221 | 1.00 | 0.00 | C |
| ATOM | 3804 | HG2 | PRO | 246 | 48.687 | 20.769 | 55.249 | 1.00 | 0.00 | H |
| ATOM | 3805 | HG3 | PRO | 246 | 47.078 | 19.990 | 55.305 | 1.00 | 0.00 | H |
| ATOM | 3806 | CB | PRO | 246 | 47.222 | 21.883 | 56.308 | 1.00 | 0.00 | C |
| ATOM | 3807 | HB2 | PRO | 246 | 47.748 | 21.664 | 57.238 | 1.00 | 0.00 | H |
| ATOM | 3808 | HB3 | PRO | 246 | 46.144 | 21.831 | 56.467 | 1.00 | 0.00 | H |

| | | | | | | | | | | | |
|---|---|---|---|---|---|---|---|---|---|---|---|
| ATOM | 3809 | CA | PRO | 246 | 47.586 | 23.264 | 55.755 | 1.00 | 0.00 | C |
| ATOM | 3810 | HA | PRO | 246 | 46.886 | 24.018 | 56.120 | 1.00 | 0.00 | H |
| ATOM | 3811 | C | PRO | 246 | 49.073 | 23.675 | 56.088 | 1.00 | 0.00 | C |
| ATOM | 3812 | O | PRO | 246 | 50.028 | 23.361 | 55.400 | 1.00 | 0.00 | O |
| ATOM | 3813 | N | THR | 247 | 49.125 | 24.324 | 57.270 | 1.00 | 0.00 | N |
| ATOM | 3814 | H | THR | 247 | 48.273 | 24.527 | 57.772 | 1.00 | 0.00 | H |
| ATOM | 3815 | CA | THR | 247 | 50.392 | 24.721 | 57.880 | 1.00 | 0.00 | C |
| ATOM | 3816 | HA | THR | 247 | 50.957 | 25.260 | 57.127 | 1.00 | 0.00 | H |
| ATOM | 3817 | CB | THR | 247 | 50.111 | 25.684 | 59.049 | 1.00 | 0.00 | C |
| ATOM | 3818 | HB | THR | 247 | 51.047 | 25.911 | 59.560 | 1.00 | 0.00 | H |
| ATOM | 3819 | CG2 | THR | 247 | 49.507 | 26.983 | 58.537 | 1.00 | 0.00 | C |
| ATOM | 3820 | HG21 | THR | 247 | 50.159 | 27.424 | 57.786 | 1.00 | 0.00 | H |
| ATOM | 3821 | HG22 | THR | 247 | 48.519 | 26.805 | 58.114 | 1.00 | 0.00 | H |
| ATOM | 3822 | HG23 | THR | 247 | 49.416 | 27.686 | 59.365 | 1.00 | 0.00 | H |
| ATOM | 3823 | OG1 | THR | 247 | 49.201 | 25.068 | 59.970 | 1.00 | 0.00 | O |
| ATOM | 3824 | HG1 | THR | 247 | 49.586 | 24.244 | 60.278 | 1.00 | 0.00 | H |
| ATOM | 3825 | C | THR | 247 | 51.295 | 23.545 | 58.386 | 1.00 | 0.00 | C |
| ATOM | 3826 | O | THR | 247 | 50.795 | 22.430 | 58.601 | 1.00 | 0.00 | O |
| ATOM | 3827 | N | GLN | 248 | 52.624 | 23.713 | 58.237 | 1.00 | 0.00 | N |
| ATOM | 3828 | H | GLN | 248 | 52.941 | 24.620 | 57.922 | 1.00 | 0.00 | H |
| ATOM | 3829 | CA | GLN | 248 | 53.718 | 22.703 | 58.534 | 1.00 | 0.00 | C |
| ATOM | 3830 | HA | GLN | 248 | 53.247 | 21.730 | 58.662 | 1.00 | 0.00 | H |
| ATOM | 3831 | CB | GLN | 248 | 54.717 | 22.614 | 57.377 | 1.00 | 0.00 | C |
| ATOM | 3832 | HB2 | GLN | 248 | 55.246 | 23.565 | 57.300 | 1.00 | 0.00 | H |
| ATOM | 3833 | HB3 | GLN | 248 | 55.444 | 21.841 | 57.607 | 1.00 | 0.00 | H |
| ATOM | 3834 | CG | GLN | 248 | 54.084 | 22.309 | 56.030 | 1.00 | 0.00 | C |
| ATOM | 3835 | HG2 | GLN | 248 | 53.399 | 23.110 | 55.748 | 1.00 | 0.00 | H |
| ATOM | 3836 | HG3 | GLN | 248 | 54.865 | 22.260 | 55.274 | 1.00 | 0.00 | H |
| ATOM | 3837 | CD | GLN | 248 | 53.355 | 20.979 | 56.021 | 1.00 | 0.00 | C |
| ATOM | 3838 | OE1 | GLN | 248 | 52.061 | 21.016 | 55.726 | 1.00 | 0.00 | O |

```
ATOM   3839  NE2 GLN   248      53.949  19.928  56.277  1.00  0.00           N
ATOM   3840 HE21 GLN   248      54.937  19.939  56.476  1.00  0.00           H
ATOM   3841 HE22 GLN   248      53.438  19.058  56.246  1.00  0.00           H
ATOM   3842  C   GLN   248      54.459  23.106  59.857  1.00  0.00           C
ATOM   3843  O   GLN   248      54.541  24.322  60.160  1.00  0.00           O
ATOM   3844  N   PRO   249      55.207  22.224  60.554  1.00  0.00           N
ATOM   3845  CD  PRO   249      55.088  20.801  60.291  1.00  0.00           C
ATOM   3846  HD2 PRO   249      55.732  20.516  59.459  1.00  0.00           H
ATOM   3847  HD3 PRO   249      54.058  20.486  60.128  1.00  0.00           H
ATOM   3848  CG  PRO   249      55.604  20.209  61.576  1.00  0.00           C
ATOM   3849  HG2 PRO   249      56.060  19.232  61.411  1.00  0.00           H
ATOM   3850  HG3 PRO   249      54.785  20.126  62.293  1.00  0.00           H
ATOM   3851  CB  PRO   249      56.599  21.204  62.069  1.00  0.00           C
ATOM   3852  HB2 PRO   249      57.539  21.066  61.532  1.00  0.00           H
ATOM   3853  HB3 PRO   249      56.762  21.111  63.143  1.00  0.00           H
ATOM   3854  CA  PRO   249      55.983  22.560  61.710  1.00  0.00           C
ATOM   3855  HA  PRO   249      55.309  22.878  62.507  1.00  0.00           H
ATOM   3856  C   PRO   249      57.063  23.614  61.485  1.00  0.00           C
ATOM   3857  O   PRO   249      57.586  23.766  60.403  1.00  0.00           O
ATOM   3858  N   LEU   250      57.467  24.284  62.544  1.00  0.00           N
ATOM   3859  H   LEU   250      57.003  24.126  63.427  1.00  0.00           H
ATOM   3860  CA  LEU   250      58.546  25.272  62.521  1.00  0.00           C
ATOM   3861  HA  LEU   250      58.283  26.072  61.844  1.00  0.00           H
ATOM   3862  CB  LEU   250      58.719  25.872  63.922  1.00  0.00           C
ATOM   3863  HB2 LEU   250      58.828  25.053  64.635  1.00  0.00           H
ATOM   3864  HB3 LEU   250      59.651  26.439  63.942  1.00  0.00           H
ATOM   3865  CG  LEU   250      57.589  26.795  64.395  1.00  0.00           C
ATOM   3866  HG  LEU   250      56.640  26.258  64.359  1.00  0.00           H
ATOM   3867  CD1 LEU   250      57.845  27.215  65.836  1.00  0.00           C
ATOM   3868 HD11 LEU   250      57.035  27.859  66.181  1.00  0.00           H
```

| | | | | | | | | | | |
|---|---|---|---|---|---|---|---|---|---|---|
| ATOM | 3869 | HD12 | LEU | 250 | 57.880 | 26.335 | 66.479 | 1.00 | 0.00 | H |
| ATOM | 3870 | HD13 | LEU | 250 | 58.789 | 27.755 | 65.914 | 1.00 | 0.00 | H |
| ATOM | 3871 | CD2 | LEU | 250 | 57.505 | 28.007 | 63.479 | 1.00 | 0.00 | C |
| ATOM | 3872 | HD21 | LEU | 250 | 56.816 | 28.737 | 63.905 | 1.00 | 0.00 | H |
| ATOM | 3873 | HD22 | LEU | 250 | 58.476 | 28.476 | 63.354 | 1.00 | 0.00 | H |
| ATOM | 3874 | HD23 | LEU | 250 | 57.112 | 27.720 | 62.505 | 1.00 | 0.00 | H |
| ATOM | 3875 | C | LEU | 250 | 59.890 | 24.690 | 62.043 | 1.00 | 0.00 | C |
| ATOM | 3876 | O | LEU | 250 | 60.684 | 25.404 | 61.449 | 1.00 | 0.00 | O |
| ATOM | 3877 | N | ASN | 251 | 60.291 | 23.411 | 62.447 | 1.00 | 0.00 | N |
| ATOM | 3878 | H | ASN | 251 | 59.657 | 22.846 | 62.993 | 1.00 | 0.00 | H |
| ATOM | 3879 | CA | ASN | 251 | 61.567 | 22.819 | 62.062 | 1.00 | 0.00 | C |
| ATOM | 3880 | HA | ASN | 251 | 61.675 | 21.889 | 62.620 | 1.00 | 0.00 | H |
| ATOM | 3881 | CB | ASN | 251 | 61.557 | 22.503 | 60.578 | 1.00 | 0.00 | C |
| ATOM | 3882 | HB2 | ASN | 251 | 61.453 | 23.417 | 59.993 | 1.00 | 0.00 | H |
| ATOM | 3883 | HB3 | ASN | 251 | 62.490 | 22.017 | 60.290 | 1.00 | 0.00 | H |
| ATOM | 3884 | CG | ASN | 251 | 60.472 | 21.533 | 60.200 | 1.00 | 0.00 | C |
| ATOM | 3885 | OD1 | ASN | 251 | 59.737 | 21.853 | 59.166 | 1.00 | 0.00 | O |
| ATOM | 3886 | ND2 | ASN | 251 | 60.301 | 20.492 | 60.846 | 1.00 | 0.00 | N |
| ATOM | 3887 | HD21 | ASN | 251 | 60.913 | 20.267 | 61.614 | 1.00 | 0.00 | H |
| ATOM | 3888 | HD22 | ASN | 251 | 59.557 | 19.864 | 60.583 | 1.00 | 0.00 | H |
| ATOM | 3889 | C | ASN | 251 | 62.781 | 23.662 | 62.394 | 1.00 | 0.00 | C |
| ATOM | 3890 | O | ASN | 251 | 63.734 | 23.528 | 61.642 | 1.00 | 0.00 | O |
| ATOM | 3891 | N | GLY | 252 | 62.794 | 24.344 | 63.542 | 1.00 | 0.00 | N |
| ATOM | 3892 | H | GLY | 252 | 61.977 | 24.326 | 64.134 | 1.00 | 0.00 | H |
| ATOM | 3893 | CA | GLY | 252 | 63.901 | 25.236 | 63.932 | 1.00 | 0.00 | C |
| ATOM | 3894 | HA2 | GLY | 252 | 64.003 | 25.197 | 65.017 | 1.00 | 0.00 | H |
| ATOM | 3895 | HA3 | GLY | 252 | 64.832 | 24.881 | 63.489 | 1.00 | 0.00 | H |
| ATOM | 3896 | C | GLY | 252 | 63.685 | 26.720 | 63.515 | 1.00 | 0.00 | C |
| ATOM | 3897 | O | GLY | 252 | 64.500 | 27.484 | 63.902 | 1.00 | 0.00 | O |
| ATOM | 3898 | N | ARG | 253 | 62.527 | 27.013 | 62.815 | 1.00 | 0.00 | N |

| | | | | | | | | | | |
|---|---|---|---|---|---|---|---|---|---|---|
| ATOM | 3899 | H | ARG | 253 | 61.906 | 26.251 | 62.586 | 1.00 | 0.00 | H |
| ATOM | 3900 | CA | ARG | 253 | 62.133 | 28.354 | 62.355 | 1.00 | 0.00 | C |
| ATOM | 3901 | HA | ARG | 253 | 63.017 | 28.823 | 61.921 | 1.00 | 0.00 | H |
| ATOM | 3902 | CB | ARG | 253 | 61.088 | 28.221 | 61.257 | 1.00 | 0.00 | C |
| ATOM | 3903 | HB2 | ARG | 253 | 61.511 | 27.603 | 60.464 | 1.00 | 0.00 | H |
| ATOM | 3904 | HB3 | ARG | 253 | 60.212 | 27.741 | 61.689 | 1.00 | 0.00 | H |
| ATOM | 3905 | CG | ARG | 253 | 60.629 | 29.537 | 60.649 | 1.00 | 0.00 | C |
| ATOM | 3906 | HG2 | ARG | 253 | 60.030 | 30.090 | 61.374 | 1.00 | 0.00 | H |
| ATOM | 3907 | HG3 | ARG | 253 | 61.497 | 30.139 | 60.378 | 1.00 | 0.00 | H |
| ATOM | 3908 | CD | ARG | 253 | 59.805 | 29.322 | 59.432 | 1.00 | 0.00 | C |
| ATOM | 3909 | HD2 | ARG | 253 | 59.381 | 30.282 | 59.133 | 1.00 | 0.00 | H |
| ATOM | 3910 | HD3 | ARG | 253 | 60.440 | 28.990 | 58.614 | 1.00 | 0.00 | H |
| ATOM | 3911 | NE | ARG | 253 | 58.711 | 28.396 | 59.674 | 1.00 | 0.00 | N |
| ATOM | 3912 | HE | ARG | 253 | 57.923 | 28.743 | 60.198 | 1.00 | 0.00 | H |
| ATOM | 3913 | CZ | ARG | 253 | 58.687 | 27.114 | 59.260 | 1.00 | 0.00 | C |
| ATOM | 3914 | NH1 | ARG | 253 | 59.704 | 26.622 | 58.587 | 1.00 | 0.00 | N |
| ATOM | 3915 | HH11 | ARG | 253 | 60.508 | 27.204 | 58.410 | 1.00 | 0.00 | H |
| ATOM | 3916 | HH12 | ARG | 253 | 59.682 | 25.670 | 58.255 | 1.00 | 0.00 | H |
| ATOM | 3917 | NH2 | ARG | 253 | 57.643 | 26.351 | 59.531 | 1.00 | 0.00 | N |
| ATOM | 3918 | HH21 | ARG | 253 | 56.853 | 26.727 | 60.032 | 1.00 | 0.00 | H |
| ATOM | 3919 | HH22 | ARG | 253 | 57.644 | 25.379 | 59.255 | 1.00 | 0.00 | H |
| ATOM | 3920 | C | ARG | 253 | 61.580 | 29.302 | 63.407 | 1.00 | 0.00 | C |
| ATOM | 3921 | O | ARG | 253 | 60.724 | 28.972 | 64.220 | 1.00 | 0.00 | O |
| ATOM | 3922 | N | VAL | 254 | 61.913 | 30.605 | 63.423 | 1.00 | 0.00 | N |
| ATOM | 3923 | H | VAL | 254 | 62.570 | 30.929 | 62.729 | 1.00 | 0.00 | H |
| ATOM | 3924 | CA | VAL | 254 | 61.424 | 31.633 | 64.392 | 1.00 | 0.00 | C |
| ATOM | 3925 | HA | VAL | 254 | 60.784 | 31.170 | 65.143 | 1.00 | 0.00 | H |
| ATOM | 3926 | CB | VAL | 254 | 62.603 | 32.310 | 65.116 | 1.00 | 0.00 | C |
| ATOM | 3927 | HB | VAL | 254 | 63.273 | 32.753 | 64.377 | 1.00 | 0.00 | H |
| ATOM | 3928 | CG1 | VAL | 254 | 62.102 | 33.420 | 66.028 | 1.00 | 0.00 | C |

| | | | | | | | | | | |
|---|---|---|---|---|---|---|---|---|---|---|
| ATOM | 3929 | HG11 | VAL | 254 | 62.903 | 33.751 | 66.686 | 1.00 | 0.00 | H |
| ATOM | 3930 | HG12 | VAL | 254 | 61.780 | 34.277 | 65.439 | 1.00 | 0.00 | H |
| ATOM | 3931 | HG13 | VAL | 254 | 61.274 | 33.053 | 66.628 | 1.00 | 0.00 | H |
| ATOM | 3932 | CG2 | VAL | 254 | 63.389 | 31.275 | 65.906 | 1.00 | 0.00 | C |
| ATOM | 3933 | HG21 | VAL | 254 | 64.200 | 31.764 | 66.446 | 1.00 | 0.00 | H |
| ATOM | 3934 | HG22 | VAL | 254 | 62.737 | 30.775 | 66.624 | 1.00 | 0.00 | H |
| ATOM | 3935 | HG23 | VAL | 254 | 63.827 | 30.532 | 65.240 | 1.00 | 0.00 | H |
| ATOM | 3936 | C | VAL | 254 | 60.586 | 32.705 | 63.618 | 1.00 | 0.00 | C |
| ATOM | 3937 | O | VAL | 254 | 60.997 | 33.164 | 62.543 | 1.00 | 0.00 | O |
| ATOM | 3938 | N | VAL | 255 | 59.463 | 33.069 | 64.163 | 1.00 | 0.00 | N |
| ATOM | 3939 | H | VAL | 255 | 59.178 | 32.663 | 65.042 | 1.00 | 0.00 | H |
| ATOM | 3940 | CA | VAL | 255 | 58.662 | 34.139 | 63.608 | 1.00 | 0.00 | C |
| ATOM | 3941 | HA | VAL | 255 | 58.907 | 34.294 | 62.557 | 1.00 | 0.00 | H |
| ATOM | 3942 | CB | VAL | 255 | 57.180 | 33.723 | 63.662 | 1.00 | 0.00 | C |
| ATOM | 3943 | HB | VAL | 255 | 56.585 | 34.515 | 63.217 | 1.00 | 0.00 | H |
| ATOM | 3944 | CG1 | VAL | 255 | 56.956 | 32.447 | 62.865 | 1.00 | 0.00 | C |
| ATOM | 3945 | HG11 | VAL | 255 | 55.885 | 32.275 | 62.748 | 1.00 | 0.00 | H |
| ATOM | 3946 | HG12 | VAL | 255 | 57.411 | 32.534 | 61.881 | 1.00 | 0.00 | H |
| ATOM | 3947 | HG13 | VAL | 255 | 57.391 | 31.587 | 63.377 | 1.00 | 0.00 | H |
| ATOM | 3948 | CG2 | VAL | 255 | 56.746 | 33.540 | 65.108 | 1.00 | 0.00 | C |
| ATOM | 3949 | HG21 | VAL | 255 | 55.714 | 33.189 | 65.122 | 1.00 | 0.00 | H |
| ATOM | 3950 | HG22 | VAL | 255 | 57.351 | 32.797 | 65.621 | 1.00 | 0.00 | H |
| ATOM | 3951 | HG23 | VAL | 255 | 56.784 | 34.482 | 65.642 | 1.00 | 0.00 | H |
| ATOM | 3952 | C | VAL | 255 | 58.844 | 35.519 | 64.331 | 1.00 | 0.00 | C |
| ATOM | 3953 | O | VAL | 255 | 59.499 | 35.487 | 65.352 | 1.00 | 0.00 | O |
| ATOM | 3954 | N | TYR | 256 | 58.285 | 36.635 | 63.871 | 1.00 | 0.00 | N |
| ATOM | 3955 | H | TYR | 256 | 57.749 | 36.639 | 63.014 | 1.00 | 0.00 | H |
| ATOM | 3956 | CA | TYR | 256 | 58.536 | 37.913 | 64.563 | 1.00 | 0.00 | C |
| ATOM | 3957 | HA | TYR | 256 | 58.670 | 37.706 | 65.621 | 1.00 | 0.00 | H |
| ATOM | 3958 | CB | TYR | 256 | 59.839 | 38.521 | 64.040 | 1.00 | 0.00 | C |

ATOM 3959 HB2 TYR 256 60.063 39.425 64.603 1.00 0.00 H

ATOM 3960 HB3 TYR 256 60.659 37.824 64.217 1.00 0.00 H

ATOM 3961 CG TYR 256 59.798 38.883 62.572 1.00 0.00 C

ATOM 3962 CD1 TYR 256 59.346 40.135 62.179 1.00 0.00 C

ATOM 3963 HD1 TYR 256 58.993 40.842 62.908 1.00 0.00 H

ATOM 3964 CE1 TYR 256 59.308 40.466 60.838 1.00 0.00 C

ATOM 3965 HE1 TYR 256 58.969 41.445 60.532 1.00 0.00 H

ATOM 3966 CZ TYR 256 59.724 39.541 59.888 1.00 0.00 C

ATOM 3967 OH TYR 256 59.686 39.871 58.552 1.00 0.00 O

ATOM 3968 HH TYR 256 59.979 39.149 57.994 1.00 0.00 H

ATOM 3969 CE2 TYR 256 60.173 38.296 60.278 1.00 0.00 C

ATOM 3970 HE2 TYR 256 60.518 37.588 59.540 1.00 0.00 H

ATOM 3971 CD2 TYR 256 60.211 37.965 61.619 1.00 0.00 C

ATOM 3972 HD2 TYR 256 60.538 36.979 61.919 1.00 0.00 H

ATOM 3973 C TYR 256 57.416 38.962 64.460 1.00 0.00 C

ATOM 3974 O TYR 256 56.768 39.042 63.436 1.00 0.00 O

ATOM 3975 N HIE 257 57.356 39.913 65.479 1.00 0.00 N

ATOM 3976 H HIE 257 57.961 39.827 66.282 1.00 0.00 H

ATOM 3977 CA HIE 257 56.518 41.167 65.343 1.00 0.00 C

ATOM 3978 HA HIE 257 55.646 40.935 64.730 1.00 0.00 H

ATOM 3979 CB HIE 257 56.006 41.624 66.712 1.00 0.00 C

ATOM 3980 HB2 HIE 257 56.847 41.708 67.402 1.00 0.00 H

ATOM 3981 HB3 HIE 257 55.553 42.612 66.617 1.00 0.00 H

ATOM 3982 CG HIE 257 54.972 40.715 67.302 1.00 0.00 C

ATOM 3983 ND1 HIE 257 53.676 40.661 66.835 1.00 0.00 N

ATOM 3984 CE1 HIE 257 52.994 39.776 67.541 1.00 0.00 C

ATOM 3985 HE1 HIE 257 51.951 39.531 67.404 1.00 0.00 H

ATOM 3986 NE2 HIE 257 53.800 39.256 68.448 1.00 0.00 N

ATOM 3987 HE2 HIE 257 53.540 38.555 69.127 1.00 0.00 H

ATOM 3988 CD2 HIE 257 55.043 39.826 68.320 1.00 0.00 C

| | | | | | | | | | | |
|---|---|---|---|---|---|---|---|---|---|---|
| ATOM | 3989 | HD2 | HIE | 257 | 55.914 | 39.610 | 68.920 | 1.00 | 0.00 | H |
| ATOM | 3990 | C | HIE | 257 | 57.231 | 42.345 | 64.696 | 1.00 | 0.00 | C |
| ATOM | 3991 | O | HIE | 257 | 58.454 | 42.412 | 64.684 | 1.00 | 0.00 | O |
| ATOM | 3992 | N | PHE | 258 | 56.485 | 43.257 | 64.082 | 1.00 | 0.00 | N |
| ATOM | 3993 | H | PHE | 258 | 55.484 | 43.123 | 64.090 | 1.00 | 0.00 | H |
| ATOM | 3994 | CA | PHE | 258 | 56.964 | 44.530 | 63.511 | 1.00 | 0.00 | C |
| ATOM | 3995 | HA | PHE | 258 | 57.994 | 44.410 | 63.172 | 1.00 | 0.00 | H |
| ATOM | 3996 | CB | PHE | 258 | 56.115 | 44.916 | 62.298 | 1.00 | 0.00 | C |
| ATOM | 3997 | HB2 | PHE | 258 | 55.066 | 44.903 | 62.586 | 1.00 | 0.00 | H |
| ATOM | 3998 | HB3 | PHE | 258 | 56.356 | 45.940 | 62.008 | 1.00 | 0.00 | H |
| ATOM | 3999 | CG | PHE | 258 | 56.328 | 44.031 | 61.102 | 1.00 | 0.00 | C |
| ATOM | 4000 | CD1 | PHE | 258 | 55.618 | 42.849 | 60.961 | 1.00 | 0.00 | C |
| ATOM | 4001 | HD1 | PHE | 258 | 54.925 | 42.553 | 61.725 | 1.00 | 0.00 | H |
| ATOM | 4002 | CE1 | PHE | 258 | 55.814 | 42.034 | 59.862 | 1.00 | 0.00 | C |
| ATOM | 4003 | HE1 | PHE | 258 | 55.224 | 41.142 | 59.779 | 1.00 | 0.00 | H |
| ATOM | 4004 | CZ | PHE | 258 | 56.722 | 42.394 | 58.890 | 1.00 | 0.00 | C |
| ATOM | 4005 | HZ | PHE | 258 | 56.886 | 41.758 | 58.034 | 1.00 | 0.00 | H |
| ATOM | 4006 | CE2 | PHE | 258 | 57.437 | 43.569 | 59.017 | 1.00 | 0.00 | C |
| ATOM | 4007 | HE2 | PHE | 258 | 58.146 | 43.855 | 58.257 | 1.00 | 0.00 | H |
| ATOM | 4008 | CD2 | PHE | 258 | 57.240 | 44.380 | 60.117 | 1.00 | 0.00 | C |
| ATOM | 4009 | HD2 | PHE | 258 | 57.801 | 45.291 | 60.212 | 1.00 | 0.00 | H |
| ATOM | 4010 | C | PHE | 258 | 56.941 | 45.676 | 64.527 | 1.00 | 0.00 | C |
| ATOM | 4011 | O | PHE | 258 | 56.358 | 45.537 | 65.591 | 1.00 | 0.00 | O |
| ATOM | 4012 | N | VAL | 259 | 57.656 | 46.800 | 64.382 | 1.00 | 0.00 | N |
| ATOM | 4013 | H | VAL | 259 | 58.165 | 46.906 | 63.514 | 1.00 | 0.00 | H |
| ATOM | 4014 | CA | VAL | 259 | 57.780 | 47.929 | 65.287 | 1.00 | 0.00 | C |
| ATOM | 4015 | HA | VAL | 259 | 57.092 | 47.807 | 66.123 | 1.00 | 0.00 | H |
| ATOM | 4016 | CB | VAL | 259 | 59.213 | 48.003 | 65.847 | 1.00 | 0.00 | C |
| ATOM | 4017 | HB | VAL | 259 | 59.281 | 48.832 | 66.553 | 1.00 | 0.00 | H |
| ATOM | 4018 | CG1 | VAL | 259 | 59.557 | 46.725 | 66.599 | 1.00 | 0.00 | C |

ATOM 4019 HG11 VAL 259 60.549 46.821 67.039 1.00 0.00 H

ATOM 4020 HG12 VAL 259 58.822 46.561 67.386 1.00 0.00 H

ATOM 4021 HG13 VAL 259 59.556 45.863 65.934 1.00 0.00 H

ATOM 4022 CG2 VAL 259 60.199 48.245 64.715 1.00 0.00 C

ATOM 4023 HG21 VAL 259 61.194 48.371 65.140 1.00 0.00 H

ATOM 4024 HG22 VAL 259 60.221 47.400 64.029 1.00 0.00 H

ATOM 4025 HG23 VAL 259 59.962 49.151 64.163 1.00 0.00 H

ATOM 4026 C VAL 259 57.447 49.240 64.620 1.00 0.00 C

ATOM 4027 O VAL 259 57.387 49.302 63.401 1.00 0.00 O

ATOM 4028 N ASP 260 57.433 50.347 65.294 1.00 0.00 N

ATOM 4029 H ASP 260 57.566 50.315 66.293 1.00 0.00 H

ATOM 4030 CA ASP 260 57.027 51.674 64.704 1.00 0.00 C

ATOM 4031 HA ASP 260 56.154 51.525 64.068 1.00 0.00 H

ATOM 4032 CB ASP 260 56.656 52.664 65.811 1.00 0.00 C

ATOM 4033 HB2 ASP 260 57.485 52.753 66.516 1.00 0.00 H

ATOM 4034 HB3 ASP 260 56.461 53.645 65.373 1.00 0.00 H

ATOM 4035 CG ASP 260 55.393 52.266 66.563 1.00 0.00 C

ATOM 4036 OD1 ASP 260 54.465 51.819 65.931 1.00 0.00 O

ATOM 4037 OD2 ASP 260 55.368 52.413 67.761 1.00 0.00 O

ATOM 4038 C ASP 260 58.157 52.286 63.822 1.00 0.00 C

ATOM 4039 O ASP 260 58.660 53.339 64.257 1.00 0.00 O

ATOM 4040 N MET 261 58.476 51.676 62.706 1.00 0.00 N

ATOM 4041 H MET 261 57.987 50.839 62.421 1.00 0.00 H

ATOM 4042 CA MET 261 59.498 52.211 61.839 1.00 0.00 C

ATOM 4043 HA MET 261 59.895 53.142 62.243 1.00 0.00 H

ATOM 4044 CB MET 261 60.657 51.219 61.760 1.00 0.00 C

ATOM 4045 HB2 MET 261 60.258 50.214 61.606 1.00 0.00 H

ATOM 4046 HB3 MET 261 61.261 51.475 60.889 1.00 0.00 H

ATOM 4047 CG MET 261 61.571 51.220 62.977 1.00 0.00 C

ATOM 4048 HG2 MET 261 62.121 52.161 63.006 1.00 0.00 H

```
ATOM   4049  HG3 MET   261      60.967  51.151  63.881  1.00  0.00           H
ATOM   4050  SD  MET   261      62.743  49.849  62.968  1.00  0.00           S
ATOM   4051  CE  MET   261      63.596  50.144  61.422  1.00  0.00           C
ATOM   4052  HE1 MET   261      64.454  49.475  61.350  1.00  0.00           H
ATOM   4053  HE2 MET   261      62.924  49.945  60.588  1.00  0.00           H
ATOM   4054  HE3 MET   261      63.938  51.178  61.383  1.00  0.00           H
ATOM   4055  C   MET   261      58.997  52.549  60.434  1.00  0.00           C
ATOM   4056  O   MET   261      58.064  51.805  59.974  1.00  0.00           O
ATOM   4057  OXT MET   261      59.583  53.439  59.788  1.00  0.00           O
TER    4057      MET   261
HETATM 4058 ZN   ZN  A 262      47.057  32.618  47.950  1.00  0.00          ZN
END
```
